# Electronic Properties of Hexagonal Graphene Quantum Rings from TAO-DFT

**DOI:** 10.3390/nano12223943

**Published:** 2022-11-09

**Authors:** Chi-Chun Chen, Jeng-Da Chai

**Affiliations:** 1 Department of Physics, National Taiwan University, Taipei 10617, Taiwan; 2 Center for Theoretical Physics and Center for Quantum Science and Engineering, National Taiwan University, Taipei 10617, Taiwan; 3 Physics Division, National Center for Theoretical Sciences, Taipei 10617, Taiwan

**Keywords:** TAO-DFT, hexagonal graphene quantum rings, polyradical nature, electronic properties

## Abstract

The reliable prediction of electronic properties associated with graphene nanosystems can be challenging for conventional electronic structure methods, such as Kohn–Sham (KS) density functional theory (DFT), due to the presence of strong static correlation effects in these systems. To address this challenge, TAO (thermally assisted occupation) DFT has been recently proposed. In the present study, we employ TAO-DFT to predict the electronic properties of *n*-HGQRs (i.e., the hexagonal graphene quantum rings consisting of *n* aromatic rings fused together at each side). From TAO-DFT, the ground states of *n*-HGQRs are singlets for all the cases investigated (*n* = 3–15). As the system size increases, there should be a transition from the nonradical to polyradical nature of ground-state *n*-HGQR. The latter should be intimately related to the localization of active TAO-orbitals at the inner and outer edges of *n*-HGQR, which increases with increasing system size.

## 1. Introduction

Since the isolation and characterization of graphene by Geim and Novoselov in 2004 [1,2], graphene has received considerable research attention. Graphene has been found to exhibit interesting properties [3,4,5,6] (e.g., the properties of graphene can be changed by modulating as composite materials [7,8,9]) which can be useful for many technology applications, such as transistors [10,11], batteries [12,13,14], quantum computers [15,16], biocatalysis [17], memory devices and photothermal applications [18]. Graphene is a two-dimensional material constructed of a sequential honeycomb lattice of carbon atoms, each forming $sp^{2}$ hybridization bonding with three other carbon atoms. Leaving one delocalized free electron which forms a π-orbital, yielding fascinating properties, such as the quantum Hall effect [19,20,21] and thermal and electronic conductivity [22,23,24]. However, graphene has zero band gap, which can be an obstacle for some electronic applications. Many studies have shown that confining the size of graphene to the nanometer scale, such as graphene nanoribbons (GNRs) [25,26,27,28,29,30], graphene quantum dots (GQDs) [31,32,33,34,35,36,37,38], and graphene quantum rings (GQRs) [34,36,37,39,40,41,42,43], could open the band gap because of finite-size effects [30]. Nonetheless, the properties of graphene nanosystems with different edges, shapes, and sizes can be distinctly different, requiring comprehensive theoretical and experimental studies.

Due to the improvement in graphene fabrication, GQRs have attracted increasing attention in recent years. Note that GQRs are closely related to GQDs, and can be regarded as GQDs with additional inner edges. It is expected that the inter-edge coupling of states and enhanced finite-size effects in GQRs could yield novel properties. Indeed, some interesting properties of GQRs, such as the Aharonov–Bohm (AB) effects [42], have been theoretically predicted [39] and experimentally observed [34]. Further theoretical studies have focused on the AB effects on the persistent currents in GQRs [44] as well as the interplay of the AB effects and Klein tunneling in GQRs [45]. In addition, other theoretical studies have revealed the importance of edge and geometry effects on the energy spectra of GQRs in a magnetic field [36,43].

**Figure 1:**
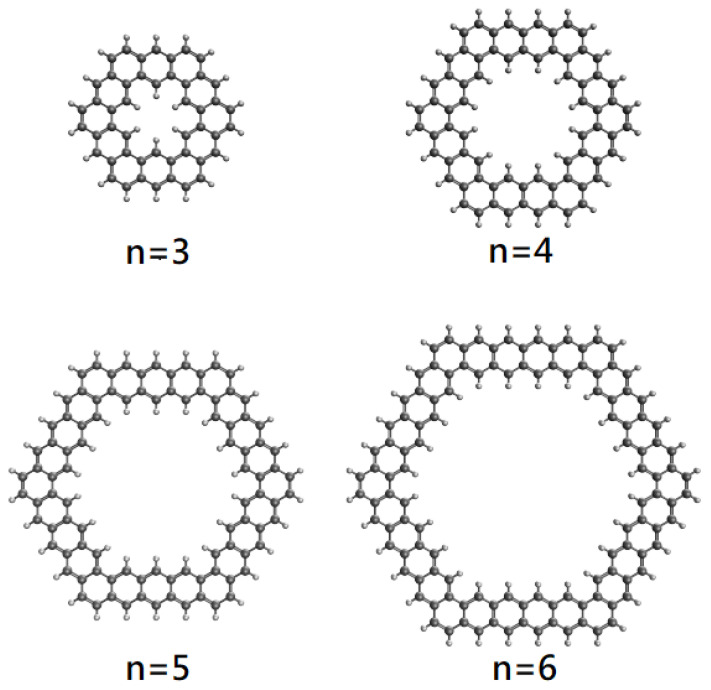
Geometry of *n*-HGQR, consisting of *n* aromatic rings fused together at each side.

Among various GQRs, in this study, we focus on the hexagonal GQRs, consisting of *n* aromatic rings fused together at each side, which are denoted as *n*-HGQRs. As shown in Figure 1, *n*-HGQR (with the chemical formula C${}_{24(n-1)}$H${}_{12(n-1)}$), which contains N=156(n−1) electrons, has six-fold rotational symmetry. To date, there have been scarce experimental studies on *n*-HGQR, which may be due to the fact that it remains very difficult to realize the precise *n*-HGQRs with smooth edges. On the theoretical side, there have been only few studies on the properties of energy spectra associated with *n*-HGQRs in the presence of a magnetic field [46,47].

Owing to the presence of strong static correlation in the larger *n*-HGQRs (which will be shown and discussed later), conventional electronic structure methods may not be appropriate. For example, Kohn–Sham (KS) density functional theory (DFT) [48,49] using the common exchange-correlation (XC) energy functionals (e.g., the LDA (local density approximation), GGA (generalized-gradient approximation), hybrid as well as double-hybrid XC functionals) have been commonly applied to predict the properties of electronic systems without strong static correlation (i.e., electronic systems with a single-reference (SR) character or SR systems), while KS-DFT with the common XC functionals can provide unreliable predictions of the properties of electronic systems with strong static correlation (i.e., electronic systems with a multi-reference (MR) character or MR systems). On the other hand, although high-level ab-initio MR electronic structure methods [50,51,52] are expected to take care of strong static correlation, accurate MR methods are known to be computationally expensive, and, thus, inapplicable to nanosystems, such as the larger *n*-HGQRs.

Recently, thermally assisted occupation (TAO) DFT, which is a DFT using fractional TAO-orbital occupation numbers (obeying the Fermi–Dirac (FD) distribution with a fictitious temperature θ) has been developed to describe the properties of nanosystems with strong static correlation [53]. Unlike finite-temperature DFT (FT-DFT) [54] (i.e., a DFT for studying the thermal equilibrium properties associated with systems at finite electronic temperatures; FT-DFT has been powered by the Mermin–Kohn–Sham (MKS) equations (see Section III of Ref. [49]), and the resulting MKS theory [49,54] is reduced to KS-DFT at zero electronic temperature (absolute zero)), TAO-DFT is a DFT for studying the ground-state properties associated with systems at absolute zero (even with a nonzero fictitious temperature θ, i.e., a parameter closely dependent on the strength of the static correlation of an electronic system at absolute zero). In the framework of TAO-DFT, the static correlation energy associated with the ground state of an electronic system is treated explicitly (though approximately) by the entropy contribution, and the corresponding natural orbital occupation numbers (NOONs) are approximately given by the TAO-orbital occupation numbers (TOONs) [53]. For the limit case of θ=0, TAO-DFT is reduced to KS-DFT. It is worth mentioning that TAO-DFT maintains a similar computational complexity as KS-DFT, while TAO-DFT can outperform KS-DFT for a diverse range of MR systems (even with the simplest LDA functional).

Moreover, a number of important extensions of TAO-DFT, such as the GGA [55] and hybrid [56,57] functionals in TAO-DFT, a simple model for defining the optimal system-independent θ in TAO-DFT [58], a self-consistent scheme for determining the optimal system-dependent θ in TAO-DFT [59], ab initio molecular dynamics based on TAO-DFT [60], and an approach for extracting excited-state properties within the TAO-DFT framework [61], have been recently developed. In addition, TAO-DFT has been successfully applied to predict the electronic properties [62,63,64,65,66,67,68,69,70,71,72,73,74,75], hydrogen storage properties [64,66,67], and spectroscopic properties [60,76,77] of various MR nanosystems in recent years.

As a result, in this work, we use TAO-DFT to explore the electronic properties (e.g., the singlet–triplet gaps, fundamental gaps and vertical electron affinities/ionization potentials, symmetrized von Neumann entropy as well as the occupation numbers and visualization of active TAO-orbitals) of *n*-HGQRs (*n* = 3–15). One of the objectives of this study is to address whether there is a transition from the nonradical to polyradical nature of ground-state *n*-HGQR with increasing system size, which has never been reported, although such a transition has been previously found in hexagonal GQDs [68] (i.e., closely related electronic systems). The significance of this work is to address whether unconventional electronic structure methods, such as TAO-DFT, are necessary for a reliable prediction of electronic properties of *n*-HGQRs.

## 2. Computational Details

We carried out all numerical calculations with the 6-31G(d) basis set on the software of Q-Chem 4.4 [78]. Geometry optimizations (see Supplementary Information (SI)) as well as single-point energy calculations were carried out using TAO-LDA, which is TAO-DFT [53] with the LDA XC and θ-dependent functionals, wherein θ=7 mhartree (i.e., an optimal system-independent θ value for TAO-LDA calculations on both SR and MR systems) [53] was adopted. For comparison purposes, we also present the results of KS-LDA (i.e., KS-DFT using the LDA XC functional or TAO-LDA with θ=0).

## 3. Results and Discussion

### 3.1. Singlet–Triplet Gap

To obtain the ground state of *n*-HGQR, we resorted to the singlet–triplet (ST) gap. First, we carried out KS-LDA and TAO-LDA calculations for the lowest spin-unrestricted triplet energy ($E_{\mathrm{UT}}$) and lowest spin-unrestricted singlet energy ($E_{\mathrm{US}}$) of *n*-HGQR at the respective optimized geometries. Subsequently, we computed the ST gap ($E_{\mathrm{ST}}$) of *n*-HGQR using

(1) $$E_{\mathrm{ST}}=E_{\mathrm{UT}}-E_{\mathrm{US}}.$$

**Figure 2:**
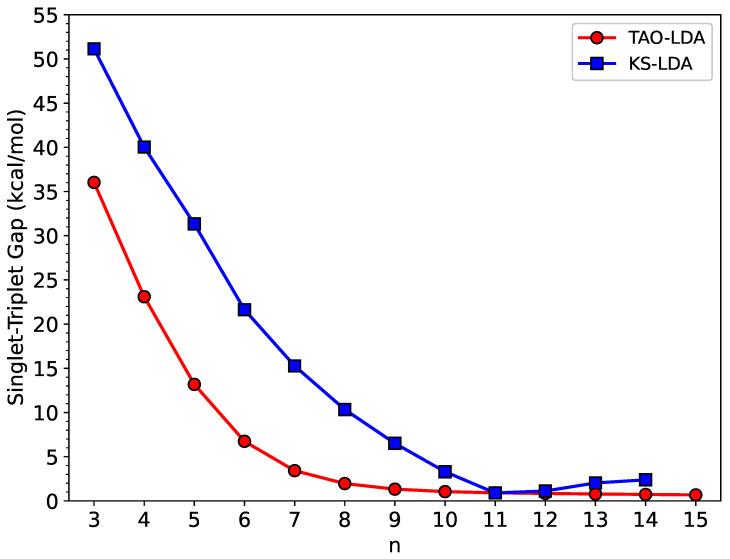
Singlet–triplet gap of *n*-HGQR calculated by spin-unrestricted KS-LDA and TAO-LDA.

As shown in Figure 2, TAO-LDA predicts that *n*-HGQRs (*n* = 3–15) (i.e., all the cases studied) should possess singlet ground states (see also Table S1 and Figure S1 (for additional information on the singlet–quintet gap of *n*-HGQR, calculated by spin-unrestricted TAO-LDA) in SI). Similarly, KS-LDA also predicts that *n*-HGQRs (*n* = 3–14) should have singlet ground states. However, the ST gap of TAO-LDA monotonically decreases with the *n*-HGQR size, while the ST gap of KS-LDA displays an unexpected increase beyond 11-HGQR.

To explore the possible reasons for discrepancies, we reveal the expectation values of the total spin-squared operator $\langle {\hat{S}}^{2}\rangle$ associated with the lowest singlet and lowest triplet states of *n*-HGQR, computed using spin-unrestricted KS-LDA (see Table 1). If the KS-LDA wavefunction has spin contamination (which is the artificial mixing of different spin-states) [68,71,79], the corresponding $\langle {\hat{S}}^{2}\rangle$ value will be larger than the exact $\langle {\hat{S}}^{2}\rangle$ value (i.e., 0 for the lowest singlet state and 2 for the lowest triplet state; e.q., refer to Section I in the SI of Ref. [79]). Consequently, the difference between the $\langle {\hat{S}}^{2}\rangle$ value of KS-LDA and the exact $\langle {\hat{S}}^{2}\rangle$ value is commonly used to assess the degree of spin contamination in the KS-LDA wavefunction. As shown, for the smaller *n*-HGQRs, the $\langle {\hat{S}}^{2}\rangle$ values of KS-LDA are rather close to the exact $\langle {\hat{S}}^{2}\rangle$ values, indicating that the lowest singlet and lowest triplet states of *n*-HGQR, obtained with spin-unrestricted KS-LDA, essentially have no spin contamination. Nevertheless, for the larger *n*-HGQRs (e.g., n≥11), the $\langle {\hat{S}}^{2}\rangle$ values of KS-LDA are much larger than the exact $\langle {\hat{S}}^{2}\rangle$ values, indicating that the lowest singlet and lowest triplet states of *n*-HGQR, obtained with spin-unrestricted KS-LDA, are highly spin-contaminated. Therefore, the larger *n*-HGQRs (e.g., n≥11) are expected to possess a pronounced MR character in the lowest singlet and lowest triplet states, and the unexpected increase in the ST gap of KS-LDA beyond 11-HGQR can be an artifact intimately correlated with spin contamination.

In addition, we also examine the consequences of spin contamination in spin-unrestricted KS-LDA/TAO-LDA calculations for the lowest singlet states of *n*-HGQRs. Owing to the constraint of spin symmetry in the lowest singlet state of *n*-HGQR, for the exact theory, the lowest singlet energy of *n*-HGQR computed using the spin-restricted formalism must be the same as that computed using the spin-unrestricted formalism [53,55,56]. Accordingly, we carried out KS-LDA and TAO-LDA calculations for the lowest spin-restricted singlet energy ($E_{\mathrm{RS}}$) and lowest spin-unrestricted singlet energy ($E_{\mathrm{US}}$) of *n*-HGQR at the corresponding optimized geometries. Subsequently, we computed the $E_{\mathrm{UR}}$ value of *n*-HGQR using

(2) $$E_{\mathrm{UR}}=E_{\mathrm{RS}}-E_{\mathrm{US}}.$$

As presented in Table 2, the $E_{\mathrm{UR}}$ values of the larger *n*-HGQRs (e.g., n≥11), calculated by KS-LDA, are very large (e.g., more than 1.6 kcal/mol), leading to unphysical spin-symmetry breaking effects in the respective spin-unrestricted KS-LDA calculations. By contrast, the $E_{\mathrm{UR}}$ values of *n*-HGQRs (*n* = 3–15) (i.e., all the cases examined), calculated by TAO-LDA, remain essentially zero, yielding essentially no unphysical spin-symmetry breaking effects in the respective spin-unrestricted TAO-LDA calculations.

In short, the ST gaps associated with the larger *n*-HGQRs (e.g., n≥11), calculated by KS-LDA, can be seriously affected by spin contamination, greatly degrading the accuracy of KS-LDA in predicting the ST gaps and possibly other electronic properties of the larger *n*-HGQRs (e.g., n≥11). As will be shown later, the larger *n*-HGQRs possess an increasing polyradical nature in the ground states (i.e., the lowest singlet states), wherein KS-LDA can provide unreliable electronic properties. Therefore, we only report the results of TAO-LDA hereafter.

### 3.2. Fundamental Gap and Vertical Electron Affinity/Ionization Potential

Another important electronic property is the fundamental gap, which is known to vanish for metals, but is positive for semiconductors and insulators [80]. Here, we employ spin-unrestricted TAO-LDA to calculate the fundamental gap ($E_{g}$) of ground-state *n*-HGQR (containing *N* electrons):

(3) $$E_{g}={\mathrm{IP}}_{v}-{\mathrm{EA}}_{v},$$

which is the difference between the ${\mathrm{IP}}_{v}$ (vertical ionization potential) and ${\mathrm{EA}}_{v}$ (vertical electron affinity) of ground-state *n*-HGQR. To obtain the ${\mathrm{IP}}_{v}$/${\mathrm{EA}}_{v}$ of ground-state *n*-HGQR, we perform spin-unrestricted TAO-LDA calculations with one electron removed from/added to neutral *n*-HGQR at the ground-state geometry, and compute the following energy difference [55,56]:

(4) $${\mathrm{IP}}_{v}=E_{N-1}-E_{N},$$

and

(5) $${\mathrm{EA}}_{v}=E_{N}-E_{N+1},$$

where $E_{N}$ refers to the total energy associated with the *N*-electron system.

**Figure 3:**
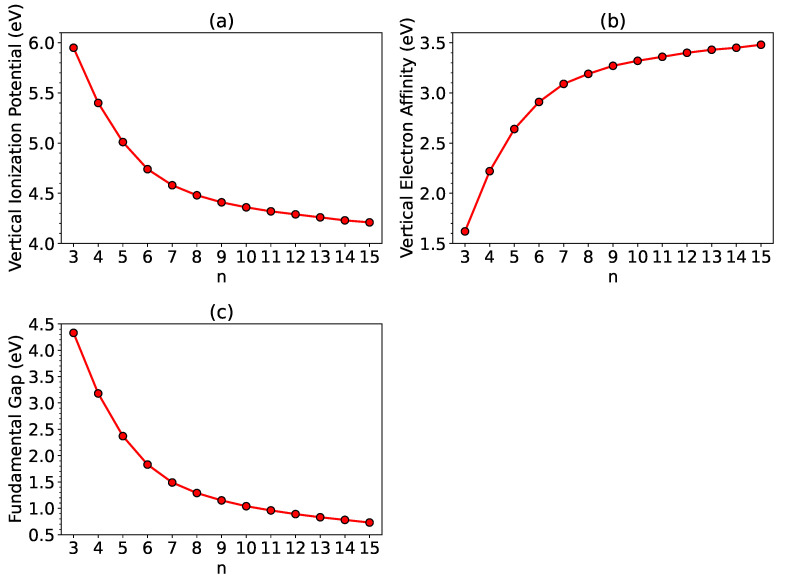
(**a**) Vertical ionization potential, (**b**) vertical electron affinity as well as (**c**) fundamental gap associated with ground-state *n*-HGQR, calculated by spin-unrestricted TAO-LDA.

As shown in Figure 3, with the increase in system size *n*, the $E_{g}$ of ground-state *n*-HGQR is monotonically decreasing, which is a consequence of the monotonically decreasing ${\mathrm{IP}}_{v}$ and monotonically increasing ${\mathrm{EA}}_{v}$ of ground-state *n*-HGQR (see, also, Table S2 in SI). Moreover, the fundamental gaps of ground-state *n*-HGQRs (*n* = 5–10) are in the range of 1–3 eV (i.e., for the ideal energy gaps), which shows great promise for the applications of ground-state *n*-HGQRs (*n* = 5–10) in nanophotonics.

### 3.3. Symmetrized von Neumann Entropy

To assess the strength of MR character associated with ground-state *n*-HGQR, we adopted spin-unrestricted TAO-LDA to calculate the $S_{\mathrm{vN}}$ (symmetrized von Neumann entropy) associated with ground-state *n*-HGQR [55,56]:

(6) $$S_{\mathrm{vN}}=-\frac{1}{2}\sum_{\sigma =↑,↓}\sum_{i=1}^{\infty }\left\{f_{i,\sigma }\;\ln\left(f_{i,\sigma }\right)+\left(1-f_{i,\sigma }\right)\;\ln\left(1-f_{i,\sigma }\right)\right\}.$$

Here, $f_{i,\sigma }$ (a value in the range of 0–1) is the occupation number associated with the ith TAO-orbital of σ-spin (i.e., up- or down-spin), which is intimately correlated with the respective natural spin-orbital occupation number [53,55,56]. For an SR system, the $f_{i,\sigma }$ values associated with all TAO-spin-orbitals must be very close to either 0 or 1, yielding a very small value of $S_{\mathrm{vN}}$. However, for an electronic system with pronounced MR character, the $f_{i,\sigma }$ values associated with the active TAO-spin-orbitals (i.e., the TAO-spin-orbitals with significant fractional occupations (e.g., 0.1–0.9)) can strongly deviate from the values of 0 and 1; hence, the corresponding value of $S_{\mathrm{vN}}$ can noticeably increase when the $f_{i,\sigma }$ values associated with the active TAO-spin-orbitals are closer to $\frac{1}{2}$, and/or the number of active TAO-spin-orbitals increases. As a consequence, the value of $S_{\mathrm{vN}}$ associated with ground-state *n*-HGQR can be adopted to assess the strength of MR character associated with ground-state *n*-HGQR. It is worth mentioning that the strength of MR character associated with ground-state *n*-HGQR may also be explored by other measures [81,82], due to the availability of TOONs (i.e., approximate NOONs) in TAO-DFT.

**Figure 4:**
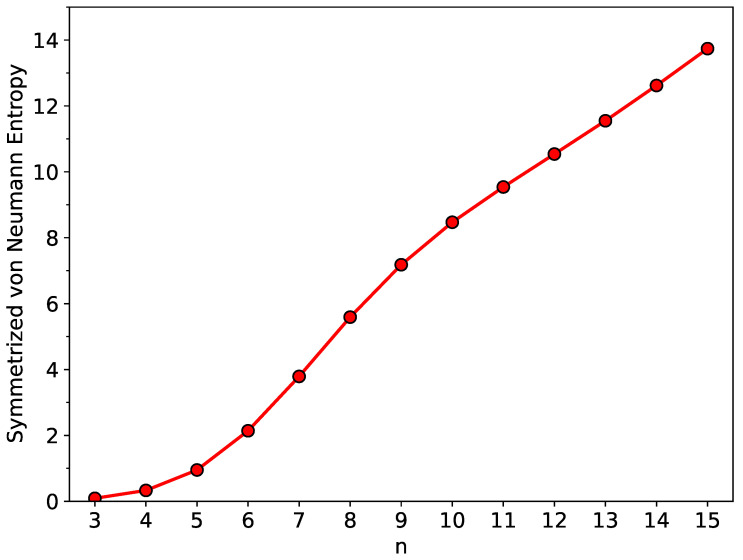
Symmetrized von Neumann entropy associated with ground-state *n*-HGQR, calculated by spin-unrestricted TAO-LDA.

As shown in Figure 4, the $S_{\mathrm{vN}}$ of ground-state *n*-HGQR, which remains very small for a small value of *n*, is monotonically increasing with the increase in *n*-HGQR size (see Table S2 in SI as well). This implies that the smaller ground-state *n*-HGQR should exhibit an SR character (i.e., nonradical nature), and the larger ground-state *n*-HGQR should possess an MR nature whose strength generally increases with increasing system size *n*.

Finally, we briefly comment on the fractional occupation number weighted density (FOD) method [83], which has been recently proposed for the real-space measure of strong static correlation effects at absolute zero. In the FOD method, the special density $\rho^{\mathrm{FOD}}$ is actually computed using an approximate TAO-DFT method [53] (i.e., TAO-DFT without the θ-dependent functional) at some fictitious temperature θ, instead of using an approximate MKS method [49,54] (i.e., the MKS theory with the XC free energy functional being approximated with the XC functional) at finite electronic temperature. This can be clearly seen from the distinctly different physical meanings of TAO-DFT [53] and the MKS theory [49,54] (which is reduced to KS-DFT at absolute zero). In other words, the fractional orbital occupation numbers adopted in the FOD method are approximate TOONs, and, hence, can be viewed as approximate NOONs. Accordingly, the $N^{\mathrm{FOD}}$ value (obtained from the integration of $\rho^{\mathrm{FOD}}$ over all space), which is similar to the aforementioned $S_{\mathrm{vN}}$ value, can also be used to measure the strength of the MR character associated with the ground state of an electronic system at absolute zero (even with a very large fictitious temperature θ), rather than that associated with an electronic system in thermal equilibrium at a finite electronic temperature.

### 3.4. Active TAO-Orbital Occupation Numbers

As mentioned previously, the NOONs associated with the ground state of an electronic system are approximately given by the TOONs in TAO-DFT [53,55,56]. To provide further insight into the increase in $S_{\mathrm{vN}}$ with increasing *n*-HGQR size and to understand the radical nature of the larger ground-state *n*-HGQR, we disclose the active TOONs of ground-state *n*-HGQR, obtained from spin-restricted TAO-LDA. For ground-state *n*-HGQR (containing *N* electrons), we denote the HOMO (highest occupied molecular orbital)/LUMO (lowest unoccupied molecular orbital) as the (N/2)th/(N/2+1)th TAO-orbital, and so forth. For brevity, we denote HOMO/LUMO as H/L. In addition, the TAO-orbitals with an orbital occupation number in the range of 0.2–1.8 (i.e., the TAO-spin-orbitals with an orbital occupation number in the range of 0.1–0.9) are viewed as the active TAO-orbitals.

**Figure 5:**
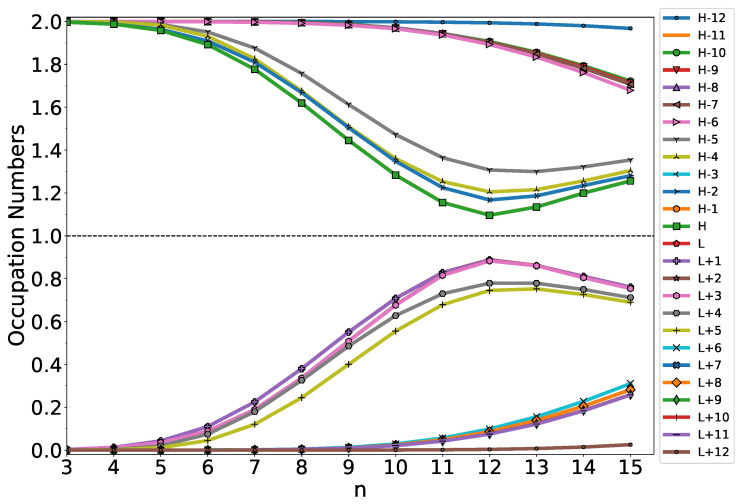
Active TAO-orbital occupation numbers (H − 12, H − 11, …, H − 1, H, L, L + 1, …, L + 11, and L + 12) of ground-state *n*-HGQR, calculated by spin-restricted TAO-LDA.

As shown in Figure 5, the active TOONs of ground-state *n*-HGQR display peculiar six-member patterns (i.e., the active TOONs are arranged to form groups of six members each), which can be related to the six-fold rotational symmetry of *n*-HGQR. When the *n*-HGQR size is small (e.g., n<7), all the TOONs are close to either 0 or 2. Therefore, the smaller ground-state *n*-HGQRs (e.g., n<7) should possess a nonradical nature, showing consistency with the investigation of the other properties, such as the larger $E_{\mathrm{ST}}$ and $E_{g}$ values and the smaller $S_{\mathrm{vN}}$ values, associated with the relatively stable *n*-HGQRs. Nevertheless, as the *n*-HGQR size increases, the active TOONs get closer to 1, and/or the number of active TAO-orbitals increases. Accordingly, the larger ground-state *n*-HGQRs (e.g., n≥7) should possess an increasingly polyradical nature, also showing consistency with the investigation of the other properties, such as the smaller $E_{\mathrm{ST}}$ and $E_{g}$ values and the larger $S_{\mathrm{vN}}$ values, associated with the relatively unstable *n*-HGQRs. As the system size grows, there is a transition from the nonradical to polyradical nature of ground-state *n*-HGQR, leading to the aforementioned increase in $S_{\mathrm{vN}}$.

### 3.5. Real-Space TAO-Orbital Representation

**Figure 6:**
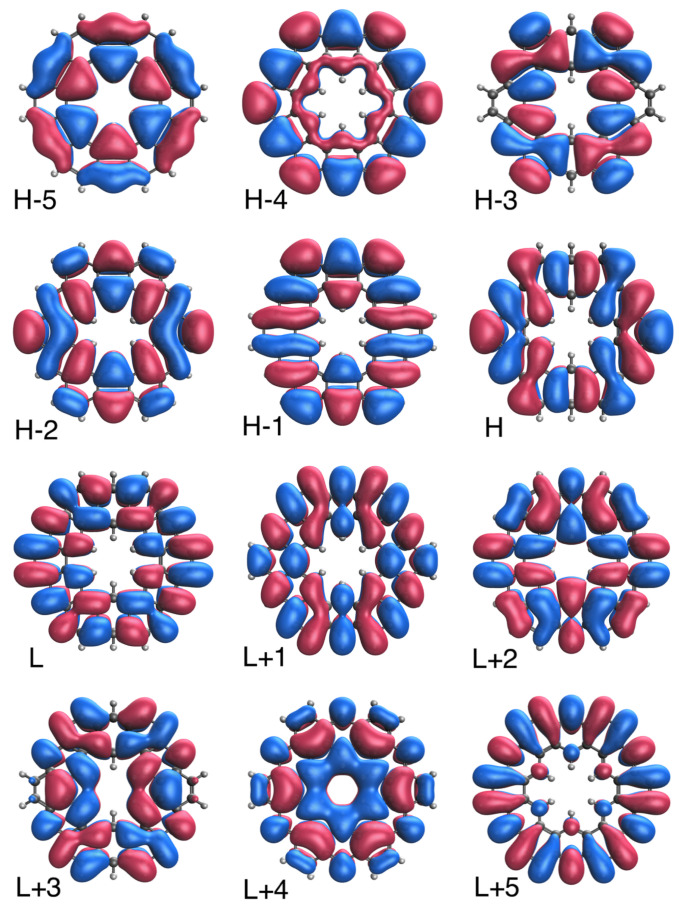
Real-space representation of active TAO-orbitals, such as H − 5 (2.000), H − 4 (2.000), H − 3 (1.998), H − 2 (1.998), H − 1 (1.996), H (1.996), L (0.003), L + 1 (0.003), L + 2 (0.002), L + 3 (0.002), L + 4 (0.000), and L + 5 (0.000), of ground-state 3-HGQR, obtained from spin-restricted TAO-LDA, at isovalue = 0.02 e/Å${}^{3}$. The TOONs are given in parentheses.

**Figure 7:**
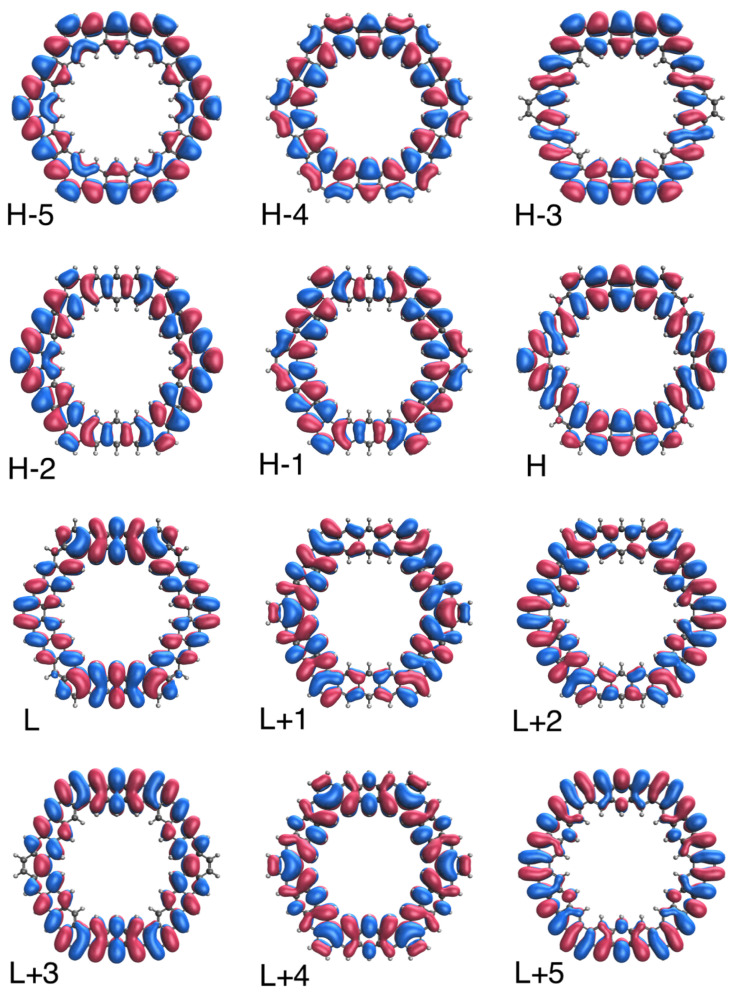
Real-space representation of active TAO-orbitals, such as H − 5 (1.987), H − 4 (1.983), H − 3 (1.964), H − 2 (1.964), H − 1 (1.958), H (1.958), L (0.043), L + 1 (0.043), L + 2 (0.035), L + 3 (0.034), L + 4 (0.020), and L + 5 (0.011), of ground-state 5-HGQR, obtained from spin-restricted TAO-LDA, at isovalue = 0.02 e/Å${}^{3}$. The TOONs are given in parentheses.

**Figure 8:**
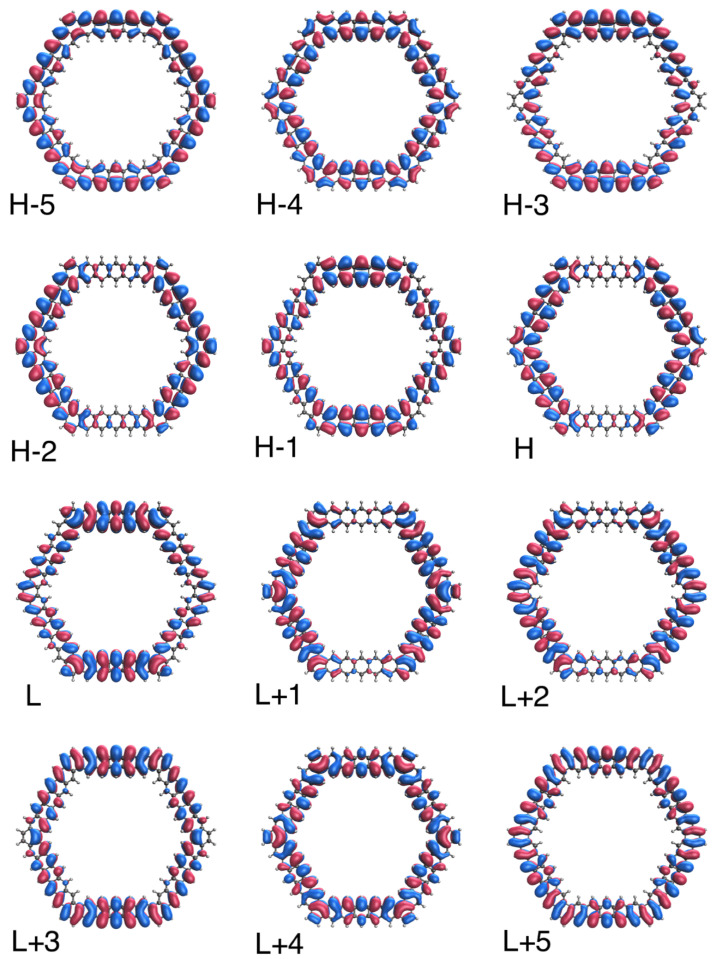
Real-space representation of active TAO-orbitals, such as H − 5 (1.876), H − 4 (1.826), H − 3 (1.810), H − 2 (1.810), H − 1 (1.777), H (1.777), L (0.225), L + 1 (0.225), L + 2 (0.190), L + 3 (0.189), L + 4 (0.179), and L + 5 (0.121), of ground-state 7-HGQR, obtained from spin-restricted TAO-LDA, at isovalue = 0.02 e/Å${}^{3}$. The TOONs are given in parentheses.

**Figure 9:**
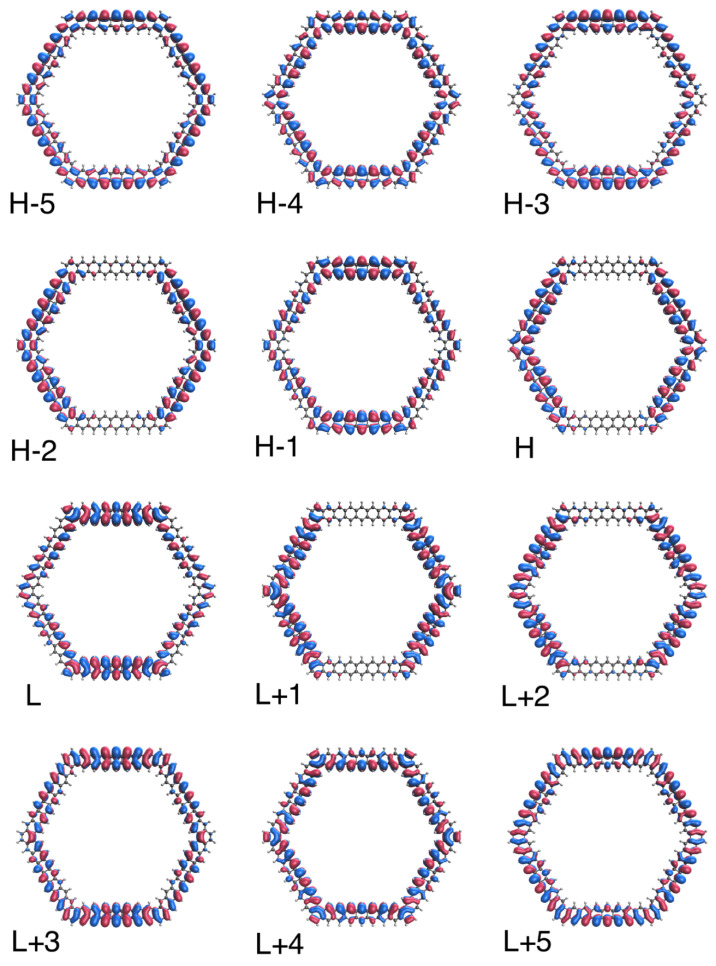
Real-space representation of active TAO-orbitals, such as H − 5 (1.613), H − 4 (1.510), H − 3 (1.504), H − 2 (1.504), H − 1 (1.446), H (1.445), L (0.551), L + 1 (0.551), L + 2 (0.509), L + 3 (0.508), L + 4 (0.486), and L + 5 (0.400) of ground-state 9-HGQR, obtained from spin-restricted TAO-LDA, at isovalue = 0.02 e/Å${}^{3}$. The TOONs are given in parentheses.

To further illustrate the transition from the nonradical to polyradical nature of ground-state *n*-HGQR in real space, we plot the visualization of active TAO-orbitals (H − 5, H − 4, …, H − 1, H, L, L + 1, …, L + 4, and L + 5) of ground-state *n*-HGQRs (*n* = 3, 5, 7, and 9), calculated by spin-restricted TAO-LDA. As shown in Figure 6, Figure 7, Figure 8 and Figure 9, when the *n*-HGQR size is small (e.g., n<7), the active TAO-orbitals are delocalized over the entire *n*-HGQR. Nonetheless, as the *n*-HGQR size increases, the active TAO-orbitals have an increasing tendency to localize at the inner and outer edges of *n*-HGQR. Consequently, the active TAO-orbitals of the larger ground-state *n*-HGQR (e.g., n≥7) are mainly localized at the two edges of *n*-HGQR.

Similar to the previous findings for other graphene nanosystems (e.g., linear acenes [62], zigzag GNRs [62], cyclacenes [65], Möbius cyclacenes [69], and hexagonal GQDs [68]), the increasing polyradical character associated with the larger ground-state *n*-HGQR (e.g., n≥7) should be intimately related to the localization of active TAO-orbitals at the inner and outer edges of *n*-HGQR, which increases as the system size *n* increases.

## 4. Conclusions

In conclusion, the MR character of graphene nanosystems has posed a great challenge to conventional electronic structure methods. The challenge has been greatly reduced by TAO-DFT. In this study, we explored the electronic properties of *n*-HGQRs (*n* = 3–15) obtained with TAO-LDA. Since the larger *n*-HGQRs were found to have pronounced MR character in the lowest singlet and lowest triplet states, KS-DFT using the common XC functionals can be unreliable for predicting the electronic properties of *n*-HGQRs. For example, the lowest singlet and triplet states of the larger *n*-HGQRs (e.g., n≥11), obtained with spin-unrestricted KS-LDA, were found to be highly spin-contaminated, leading to unphysical spin-symmetry breaking effects. Although accurate MR electronic structure methods are able to describe strong static correlation effects, these methods can be computationally intractable for the larger *n*-HGQRs. Consequently, it is reasonably justified that we employ TAO-LDA to study the electronic properties of *n*-HGQRs due to a good compromise between efficiency and accuracy.

From the TAO-LDA results, *n*-HGQRs (*n* = 3–15) are all ground-state singlets. With increasing *n*-HGQR size, the $E_{\mathrm{ST}}$, ${\mathrm{IP}}_{v}$, and $E_{g}$ values monotonically decrease, while the ${\mathrm{EA}}_{v}$ and $S_{\mathrm{vN}}$ values monotonically increase. With increasing system size, there is a transition from the nonradical to polyradical nature of ground-state *n*-HGQR. The increasing polyradical character associated with the larger ground-state *n*-HGQR (e.g., n≥7) should be intimately related to the localization of active TAO-orbitals at the inner and outer edges of *n*-HGQR, which increases with increasing system size.

As for possible future work, we plan to explore how the radical nature emerges from the evolution between *n*-HGQRs and hexagonal GQDs (i.e., the coronene series) [68] to see the trend forming hexagonal GQDs.

## Figures and Tables

**Table 1:** Expectation values of the total spin-squared operator $\langle {\hat{S}}^{2}\rangle$ associated with the lowest singlet and lowest triplet states of *n*-HGQR, calculated by spin-unrestricted KS-LDA. For the exact theory, the value of $\langle {\hat{S}}^{2}\rangle$ should be 0 for the lowest singlet state and two for the lowest triplet state.

*n*	Singlet	Triplet
3	0.0000	2.0047
4	0.0000	2.0040
5	0.0000	2.0028
6	0.0000	2.0054
7	0.0000	2.0068
8	0.0000	2.0101
9	0.0001	2.0271
10	0.0004	2.8843
11	4.1432	5.1379
12	5.1804	6.2166
13	4.8429	5.9705
14	3.8338	5.0410

**Table 2:** Difference $E_{\mathrm{UR}}$ (in kcal/mol) between the lowest spin-restricted singlet energy and lowest spin-unrestricted singlet energy of *n*-HGQR, calculated by KS-LDA and TAO-LDA.

*n*	KS-LDA	TAO-LDA
3	0.000	0.000
4	0.000	0.000
5	0.000	0.000
6	0.000	0.000
7	0.000	0.000
8	0.000	0.000
9	0.000	0.000
10	0.000	0.000
11	1.609	0.000
12	7.633	0.000
13	5.261	0.000
14	2.489	0.000
15		0.000

## Data Availability

The data that support the findings of this study are available from the authors upon reasonable request.

## Supplementary Materials

The following supporting information can be downloaded at: https://www.mdpi.com/article/10.3390/nano12223943/s1, Cartesian coordinates (in Å) for the lowest singlet states of *n*-HGQRs (*n* = 3–15), calculated by spin-unrestricted TAO-LDA; Cartesian coordinates (in Å) for the lowest triplet states of *n*-HGQRs (*n* = 3–15), calculated by spin-unrestricted TAO-LDA; Figure S1: Singlet–quintet gap of *n*-HGQR, calculated by spin-unrestricted TAO-LDA; Table S1: Singlet–triplet gap $E_{\mathrm{ST}}$ (in kcal/mol) of *n*-HGQR, calculated by spin-unrestricted KS-LDA and TAO-LDA; Table S2: Vertical ionization potential ${\mathrm{IP}}_{v}$ (in eV), vertical electron affinity ${\mathrm{EA}}_{v}$ (in eV), fundamental gap $E_{g}$ (in eV), and symmetrized von Neumann entropy $S_{\mathrm{vN}}$ of ground-state *n*-HGQR, calculated by spin-unrestricted TAO-LDA.
