# Supplementary material for: Electronic Properties of Hexagonal Graphene Quantum Rings from TAO-DFT
Source: Nanomaterials (Basel). 2022 Nov 9;12(22):3943. doi: 10.3390/nano12223943 (PMC9694783; doi:10.3390/nano12223943)
Supplement: Supplementary file 1 [file nanomaterials-12-03943-s001.zip › nanomaterials-2015409-supplementary.pdf]

Supplementary Information to: Electronic Properties of Hexagonal Graphene Quantum Rings from TAO-DFT

Chi-Chun Chen[1] and Jeng-Da Chai[1, 2, 3, *]

[1] *Department of Physics, National Taiwan University, Taipei 10617, Taiwan*
[2] *Center for Theoretical Physics and Center for Quantum Science and Engineering, National Taiwan University, Taipei 10617, Taiwan*
[3] *Physics Division, National Center for Theoretical Sciences, Taipei 10617, Taiwan*

Cartesian coordinates for the lowest singlet states of n-HGQRs (n = 3–15), calculated by spin-unrestricted TAO-LDA (S2 to S47).

Cartesian coordinates for the lowest triplet states of n-HGQRs (n = 3–15), calculated by spin-unrestricted TAO-LDA (S48 to S93).

FIG. S1. Singlet-quintet gap of n-HGQR, calculated by spin-unrestricted TAO-LDA (S94).

TABLE S1. Singlet-triplet gap of n-HGQR, calculated by spin-unrestricted KS-LDA and TAO-LDA (S94).

TABLE S2. Vertical ionization potential, vertical electron affinity, fundamental gap, and symmetrized von Neumann entropy of ground-state n-HGQR, calculated by spin-unrestricted TAO-LDA (S95).

* Author to whom correspondence should be addressed. Electronic mail: jdchai@phys.ntu.edu.tw

Cartesian coordinates (in Å) for the lowest singlet states of n-HGQRs (n = 3–15), calculated by spin-unrestricted TAO-LDA.

3-HGQR (singlet)

C	-6.14848528	-0.68184816	0.00000000
C	-6.14848532	0.68184808	0.00000000
C	-4.90417045	-2.83169560	0.00000000
C	-4.93177990	-1.43278832	0.00000000
C	-4.93177995	1.43278833	0.00000000
C	-4.90417056	2.83169561	0.00000000
C	-3.66496130	-4.98470372	0.00000000
C	-3.70680641	-3.55545297	0.00000000
C	-3.69499632	-0.72289751	0.00000000
C	-3.69499631	0.72289750	0.00000000
C	-3.70680663	3.55545296	0.00000000
C	-3.66496144	4.98470369	0.00000000
C	-2.48404937	-5.66666552	0.00000000
C	-2.47364017	-2.83939036	0.00000000
C	-2.51277813	-1.45128974	0.00000000
C	-2.51277820	1.45128979	0.00000000
C	-2.47364041	2.83939039	0.00000000
C	-2.48404945	5.66666556	0.00000000
C	-1.22533924	-4.98850315	0.00000000
C	-1.22170225	-3.56235090	0.00000000
C	-1.22170246	3.56235097	0.00000000
C	-1.22533922	4.98850324	0.00000000
C	-0.00000001	-5.66384240	0.00000000
C	0.00000000	-2.90237945	0.00000000
C	-0.00000002	2.90237935	0.00000000
C	-0.00000001	5.66384238	0.00000000
C	1.22533924	-4.98850315	0.00000000
C	1.22170226	-3.56235090	0.00000000
C	1.22170244	3.56235096	0.00000000
C	1.22533920	4.98850323	0.00000000
C	2.48404937	-5.66666553	0.00000000
C	2.47364018	-2.83939036	0.00000000
C	2.51277815	-1.45128975	0.00000000
C	2.51277820	1.45128976	0.00000000
C	2.47364040	2.83939036	0.00000000
C	2.48404943	5.66666554	0.00000000
C	3.66496131	-4.98470374	0.00000000
C	3.70680641	-3.55545298	0.00000000
C	3.69499634	-0.72289752	0.00000000
C	3.69499632	0.72289749	0.00000000
C	3.70680661	3.55545295	0.00000000
C	3.66496143	4.98470367	0.00000000
C	4.90417046	-2.83169562	0.00000000
C	4.93177992	-1.43278834	0.00000000
C	4.93177995	1.43278833	0.00000000
C	4.90417056	2.83169560	0.00000000
C	6.14848530	-0.68184815	0.00000000
C	6.14848532	0.68184808	0.00000000
H	-7.09736725	-1.23595024	0.00000000
H	-7.09736731	1.23595014	0.00000000
H	-5.85717467	-3.38178623	0.00000000
H	-5.85717483	3.38178615	0.00000000
H	-4.61926075	-5.52943853	0.00000000
H	-4.61926081	5.52943865	0.00000000
H	-2.47862582	-6.76546748	0.00000000
H	-2.47862599	6.76546754	0.00000000
H	-1.56467070	-0.90452921	0.00000000
H	-1.56467068	0.90452933	0.00000000
H	0.00000000	-6.76420128	0.00000000
H	-0.00000001	-1.80792276	0.00000000
H	-0.00000003	1.80792263	0.00000000
H	0.00000001	6.76420125	0.00000000
H	1.56467074	-0.90452920	0.00000000
H	1.56467070	0.90452929	0.00000000
H	2.47862582	-6.76546749	0.00000000
H	2.47862598	6.76546753	0.00000000
H	4.61926076	-5.52943854	0.00000000
H	4.61926079	5.52943863	0.00000000
H	5.85717468	-3.38178625	0.00000000
H	5.85717482	3.38178615	0.00000000
H	7.09736727	-1.23595023	0.00000000
H	7.09736731	1.23595014	0.00000000

4-HGQR (singlet)

C	-8.59735933	-0.67995805	0.00000000
C	-8.59735926	0.67995902	0.00000000
C	-7.38450940	-2.82689430	0.00000000
C	-7.38113439	-1.43602754	0.00000000
C	-7.38113435	1.43602829	0.00000000

C	-7.38450931	2.82689481	0.00000000
C	-6.14031201	-4.98223851	0.00000000
C	-6.19349387	-3.57611843	0.00000000
C	-6.13197078	-0.72570470	0.00000000
C	-6.13197079	0.72570545	0.00000000
C	-6.19349388	3.57611875	0.00000000
C	-6.14031195	4.98223879	0.00000000
C	-4.88749518	-7.10593705	0.00000000
C	-4.93404767	-5.67456817	0.00000000
C	-4.95125308	-2.85883836	0.00000000
C	-4.96068454	-1.45787206	0.00000000
C	-4.96068454	1.45787264	0.00000000
C	-4.95125316	2.85883879	0.00000000
C	-4.93404774	5.67456833	0.00000000
C	-4.88749534	7.10593697	0.00000000
C	-3.70990012	-7.78601699	0.00000000
C	-3.69433375	-4.94778840	0.00000000
C	-3.74261318	-3.56722204	0.00000000
C	-3.74261341	3.56722245	0.00000000
C	-3.69433409	4.94778857	0.00000000
C	-3.70990060	7.78601678	0.00000000
C	-2.44704249	-7.11077272	0.00000000
C	-2.43756522	-5.67363797	0.00000000
C	-2.43756581	5.67363801	0.00000000
C	-2.44704321	7.11077259	0.00000000
C	-1.24431808	-7.80924122	0.00000000
C	-1.21785796	-5.02537563	0.00000000
C	-1.21785881	5.02537568	0.00000000
C	-1.24431904	7.80924113	0.00000000
C	0.00000048	-7.15237183	0.00000000
C	0.00000047	-5.71775330	0.00000000
C	-0.00000047	5.71775332	0.00000000
C	-0.00000049	7.15237185	0.00000000
C	1.24431904	-7.80924110	0.00000000
C	1.21785881	-5.02537565	0.00000000
C	1.21785797	5.02537566	0.00000000
C	1.24431807	7.80924125	0.00000000
C	2.44704322	-7.11077257	0.00000000
C	2.43756580	-5.67363799	0.00000000
C	2.43756523	5.67363798	0.00000000
C	2.44704249	7.11077274	0.00000000
C	3.70990061	-7.78601677	0.00000000
C	3.69433409	-4.94778857	0.00000000
C	3.74261340	-3.56722245	0.00000000
C	3.74261319	3.56722204	0.00000000
C	3.69433375	4.94778840	0.00000000
C	3.70990012	7.78601699	0.00000000
C	4.88749536	-7.10593698	0.00000000
C	4.93404774	-5.67456834	0.00000000
C	4.95125316	-2.85883880	0.00000000
C	4.96068454	-1.45787265	0.00000000
C	4.96068453	1.45787205	0.00000000
C	4.95125309	2.85883835	0.00000000
C	4.93404767	5.67456816	0.00000000
C	4.88749517	7.10593703	0.00000000
C	6.14031196	-4.98223880	0.00000000
C	6.19349387	-3.57611876	0.00000000
C	6.13197079	-0.72570545	0.00000000
C	6.13197077	0.72570470	0.00000000
C	6.19349388	3.57611841	0.00000000
C	6.14031200	4.98223850	0.00000000
C	7.38450930	-2.82689482	0.00000000
C	7.38113435	-1.43602830	0.00000000
C	7.38113440	1.43602752	0.00000000
C	7.38450942	2.82689428	0.00000000
C	8.59735926	-0.67995903	0.00000000
C	8.59735934	0.67995804	0.00000000
H	-9.54558737	-1.23517423	0.00000000
H	-9.54558727	1.23517513	0.00000000
H	-8.34857090	-3.35688420	0.00000000
H	-8.34857072	3.35688480	0.00000000
H	-7.08119878	-5.55241172	0.00000000
H	-7.08119868	5.55241199	0.00000000
H	-5.84248661	-7.64943087	0.00000000
H	-5.84248675	7.64943073	0.00000000
H	-3.98588399	-0.95178880	0.00000000
H	-3.98588404	0.95178931	0.00000000
H	-3.70317434	-8.88480291	0.00000000
H	-3.70317484	8.88480267	0.00000000
H	-2.81692842	-2.97598936	0.00000000
H	-2.81692863	2.97598982	0.00000000
H	-1.26752800	-8.90913030	0.00000000
H	-1.16898452	-3.92814743	0.00000000

H	-1.16898528	3.92814749	0.00000000
H	-1.26752898	8.90913017	0.00000000
H	1.26752898	-8.90913015	0.00000000
H	1.16898529	-3.92814746	0.00000000
H	1.16898452	3.92814745	0.00000000
H	1.26752801	8.90913033	0.00000000
H	2.81692863	-2.97598981	0.00000000
H	2.81692842	2.97598936	0.00000000
H	3.70317483	-8.88480265	0.00000000
H	3.70317437	8.88480292	0.00000000
H	3.98588404	-0.95178933	0.00000000
H	3.98588398	0.95178879	0.00000000
H	5.84248676	-7.64943074	0.00000000
H	5.84248660	7.64943084	0.00000000
H	7.08119870	-5.55241198	0.00000000
H	7.08119876	5.55241171	0.00000000
H	8.34857071	-3.35688481	0.00000000
H	8.34857092	3.35688418	0.00000000
H	9.54558726	-1.23517515	0.00000000
H	9.54558738	1.23517422	0.00000000

5-HGQR (singlet)

C	-11.04713949	-0.67967855	0.00000000
C	-11.04713939	0.67967914	0.00000000
C	-9.84124389	-2.82215311	0.00000000
C	-9.83142003	-1.43571198	0.00000000
C	-9.83141982	1.43571235	0.00000000
C	-9.84124339	2.82215355	0.00000000
C	-8.63192208	-4.98378347	0.00000000
C	-8.65364324	-3.58391149	0.00000000
C	-8.57624459	-0.72663603	0.00000000
C	-8.57624448	0.72663617	0.00000000
C	-8.65364261	3.58391179	0.00000000
C	-8.63192124	4.98378370	0.00000000
C	-7.36422820	-7.11139688	0.00000000
C	-7.43022049	-5.70202062	0.00000000
C	-7.40102221	-2.86794294	0.00000000
C	-7.41007625	-1.46159190	0.00000000
C	-7.41007592	1.46159195	0.00000000
C	-7.40102166	2.86794311	0.00000000
C	-7.43021969	5.70202062	0.00000000
C	-7.36422737	7.11139670	0.00000000
C	-6.11166758	-9.22662573	0.00000000
C	-6.15848711	-7.79573435	0.00000000
C	-6.18405390	-4.97479348	0.00000000
C	-6.20453355	-3.58167311	0.00000000
C	-6.20453288	3.58167327	0.00000000
C	-6.18405321	4.97479356	0.00000000
C	-6.15848640	7.79573394	0.00000000
C	-6.11166694	9.22662519	0.00000000
C	-4.93453074	-9.90633113	0.00000000
C	-4.91693311	-7.06292515	0.00000000
C	-4.97043162	-5.68538830	0.00000000
C	-4.97043108	5.68538818	0.00000000
C	-4.91693257	7.06292474	0.00000000
C	-4.93453023	9.90633054	0.00000000
C	-3.67204209	-9.23136886	0.00000000
C	-3.65848973	-7.78962833	0.00000000
C	-3.65848930	7.78962783	0.00000000
C	-3.67204170	9.23136828	0.00000000
C	-2.47642571	-9.93324522	0.00000000
C	-2.43884385	-7.14729686	0.00000000
C	-2.43884356	7.14729644	0.00000000
C	-2.47642551	9.93324465	0.00000000
C	-1.22293236	-9.28571495	0.00000000
C	-1.21650750	-7.84275201	0.00000000
C	-1.21650729	7.84275156	0.00000000
C	-1.22293221	9.28571447	0.00000000
C	-0.00000003	-9.96710930	0.00000000
C	-0.00000006	-7.16365156	0.00000000
C	0.00000013	7.16365121	0.00000000
C	0.00000007	9.96710890	0.00000000
C	1.22293227	-9.28571491	0.00000000
C	1.21650739	-7.84275198	0.00000000
C	1.21650750	7.84275163	0.00000000
C	1.22293236	9.28571453	0.00000000
C	2.47642564	-9.93324515	0.00000000
C	2.43884372	-7.14729679	0.00000000
C	2.43884379	7.14729656	0.00000000
C	2.47642564	9.93324477	0.00000000
C	3.67204199	-9.23136876	0.00000000
C	3.65848961	-7.78962822	0.00000000
C	3.65848949	7.78962800	0.00000000
C	3.67204185	9.23136844	0.00000000

C	4.93453065	-9.90633101	0.00000000
C	4.91693297	-7.06292502	0.00000000
C	4.97043148	-5.68538816	0.00000000
C	4.97043122	5.68538838	0.00000000
C	4.91693275	7.06292493	0.00000000
C	4.93453038	9.90633072	0.00000000
C	6.11166748	-9.22662559	0.00000000
C	6.15848698	-7.79573421	0.00000000
C	6.18405376	-4.97479334	0.00000000
C	6.20453341	-3.58167296	0.00000000
C	6.20453295	3.58167345	0.00000000
C	6.18405333	4.97479374	0.00000000
C	6.15848658	7.79573413	0.00000000
C	6.11166709	9.22662537	0.00000000
C	7.36422807	-7.11139674	0.00000000
C	7.43022034	-5.70202048	0.00000000
C	7.40102209	-2.86794280	0.00000000
C	7.41007615	-1.46159176	0.00000000
C	7.41007590	1.46159211	0.00000000
C	7.40102170	2.86794327	0.00000000
C	7.43021982	5.70202078	0.00000000
C	7.36422754	7.11139687	0.00000000
C	8.63192194	-4.98378336	0.00000000
C	8.65364310	-3.58391137	0.00000000
C	8.57624451	-0.72663591	0.00000000
C	8.57624444	0.72663629	0.00000000
C	8.65364267	3.58391192	0.00000000
C	8.63192136	4.98378383	0.00000000
C	9.84124377	-2.82215301	0.00000000
C	9.83141993	-1.43571188	0.00000000
C	9.83141980	1.43571243	0.00000000
C	9.84124342	2.82215363	0.00000000
C	11.04713942	-0.67967849	0.00000000
C	11.04713936	0.67967920	0.00000000
H	-11.99493458	-1.23568456	0.00000000
H	-11.99493439	1.23568529	0.00000000
H	-10.80795392	-3.34744521	0.00000000
H	-10.80795331	3.34744580	0.00000000
H	-9.58436768	-5.53419846	0.00000000
H	-9.58436676	5.53419874	0.00000000
H	-8.30233310	-7.68626555	0.00000000
H	-8.30233223	7.68626539	0.00000000
H	-7.06711137	-9.76936679	0.00000000
H	-7.06711071	9.76936621	0.00000000
H	-6.43357620	-0.95839386	0.00000000
H	-6.43357596	0.95839377	0.00000000
H	-5.25188857	-3.03118569	0.00000000
H	-5.25188798	3.03118576	0.00000000
H	-4.92680005	-11.00513202	0.00000000
H	-4.92679953	11.00513139	0.00000000
H	-4.04650284	-5.09110099	0.00000000
H	-4.04650238	5.09110085	0.00000000
H	-2.50505656	-11.03307755	0.00000000
H	-2.38637661	-6.05004925	0.00000000
H	-2.38637633	6.05004890	0.00000000
H	-2.50505638	11.03307694	0.00000000
H	-0.00000001	-11.06713884	0.00000000
H	-0.00000008	-6.06343228	0.00000000
H	0.00000016	6.06343202	0.00000000
H	0.00000004	11.06713839	0.00000000
H	2.50505653	-11.03307748	0.00000000
H	2.38637644	-6.05004917	0.00000000
H	2.38637661	6.05004902	0.00000000
H	2.50505647	11.03307705	0.00000000
H	4.04650269	-5.09110086	0.00000000
H	4.04650251	5.09110107	0.00000000
H	4.92679998	-11.00513190	0.00000000
H	4.92679966	11.00513156	0.00000000
H	5.25188844	-3.03118554	0.00000000
H	5.25188803	3.03118598	0.00000000
H	6.43357611	-0.95839371	0.00000000
H	6.43357592	0.95839397	0.00000000
H	7.06711128	-9.76936663	0.00000000
H	7.06711086	9.76936641	0.00000000
H	8.30233298	-7.68626540	0.00000000
H	8.30233240	7.68626554	0.00000000
H	9.58436753	-5.53419835	0.00000000
H	9.58436690	5.53419884	0.00000000
H	10.80795379	-3.34744512	0.00000000
H	10.80795336	3.34744585	0.00000000
H	11.99493450	-1.23568454	0.00000000
H	11.99493437	1.23568533	0.00000000

6-HGQR (singlet)

C	-13.49724507	-0.67987993	0.00000000
C	-13.49724493	0.67988116	0.00000000
C	-12.29497003	-2.81979264	0.00000000
C	-12.28226189	-1.43467990	0.00000000
C	-12.28226158	1.43468085	0.00000000
C	-12.29496947	2.81979354	0.00000000
C	-11.09568159	-4.98131804	0.00000000
C	-11.10916821	-3.58538969	0.00000000
C	-11.02394811	-0.72648150	0.00000000
C	-11.02394795	0.72648219	0.00000000
C	-11.10916751	3.58539028	0.00000000
C	-11.09568070	4.98131855	0.00000000
C	-9.86147147	-7.11738630	0.00000000
C	-9.89720732	-5.71341967	0.00000000
C	-9.85185774	-2.87235649	0.00000000
C	-9.85960502	-1.46419943	0.00000000
C	-9.85960470	1.46419983	0.00000000
C	-9.85185717	2.87235684	0.00000000
C	-9.89720633	5.71341985	0.00000000
C	-9.86147034	7.11738638	0.00000000
C	-8.58841856	-9.23584067	0.00000000
C	-8.65888863	-7.82615086	0.00000000
C	-8.64137593	-4.98782581	0.00000000
C	-8.66051270	-3.58933337	0.00000000
C	-8.66051200	3.58933341	0.00000000
C	-8.64137503	4.98782577	0.00000000
C	-8.65888742	7.82615061	0.00000000
C	-8.58841728	9.23584031	0.00000000
C	-7.33603880	-11.34638678	0.00000000
C	-7.38227047	-9.91676653	0.00000000
C	-7.41310758	-7.09307960	0.00000000
C	-7.43935811	-5.70278063	0.00000000
C	-7.43935712	5.70278028	0.00000000
C	-7.41310646	7.09307917	0.00000000
C	-7.38226918	9.91676588	0.00000000
C	-7.33603742	11.34638608	0.00000000
C	-6.15849582	-12.02614535	0.00000000
C	-6.14001892	-9.18056339	0.00000000
C	-6.19710608	-7.80321729	0.00000000
C	-6.19710489	7.80321659	0.00000000
C	-6.14001773	9.18056258	0.00000000
C	-6.15849459	12.02614448	0.00000000
C	-4.89755442	-11.35101505	0.00000000
C	-4.88174463	-9.90697445	0.00000000
C	-4.88174350	9.90697352	0.00000000
C	-4.89755330	11.35101411	0.00000000
C	-3.70454377	-12.05469268	0.00000000
C	-3.66059360	-9.26759594	0.00000000
C	-3.66059267	9.26759492	0.00000000
C	-3.70454289	12.05469179	0.00000000
C	-2.44866211	-11.41062695	0.00000000
C	-2.43731144	-9.96507793	0.00000000
C	-2.43731068	9.96507696	0.00000000
C	-2.44866141	11.41062602	0.00000000
C	-1.23337786	-12.09730551	0.00000000
C	-1.22045789	-9.29218592	0.00000000
C	-1.22045732	9.29218493	0.00000000
C	-1.23337743	12.09730469	0.00000000
C	-0.00000071	-11.42580027	0.00000000
C	-0.00000062	-9.97525101	0.00000000
C	-0.00000031	9.97525016	0.00000000
C	-0.00000049	11.42579945	0.00000000
C	1.23337639	-12.09730573	0.00000000
C	1.22045677	-9.29218605	0.00000000
C	1.22045689	9.29218522	0.00000000
C	1.23337633	12.09730508	0.00000000
C	2.44866083	-11.41062737	0.00000000
C	2.43731035	-9.96507822	0.00000000
C	2.43731019	9.96507757	0.00000000
C	2.44866059	11.41062675	0.00000000
C	3.70454253	-12.05469337	0.00000000
C	3.66059276	-9.26759639	0.00000000
C	3.66059247	9.26759582	0.00000000
C	3.70454203	12.05469293	0.00000000
C	4.89755341	-11.35101584	0.00000000
C	4.88174387	-9.90697512	0.00000000
C	4.88174331	9.90697477	0.00000000
C	4.89755276	11.35101547	0.00000000
C	6.15849491	-12.02614630	0.00000000
C	6.14001837	-9.18056416	0.00000000
C	6.19710565	-7.80321780	0.00000000
C	6.19710503	7.80321775	0.00000000
C	6.14001777	9.18056399	0.00000000

C	6.15849412	12.02614605	0.00000000
C	7.33603810	-11.34638778	0.00000000
C	7.38227005	-9.91676738	0.00000000
C	7.41310737	-7.09308004	0.00000000
C	7.43935803	-5.70278082	0.00000000
C	7.43935752	5.70278113	0.00000000
C	7.41310679	7.09308025	0.00000000
C	7.38226933	9.91676739	0.00000000
C	7.33603721	11.34638774	0.00000000
C	8.58841830	-9.23584147	0.00000000
C	8.65888850	-7.82615154	0.00000000
C	8.64137605	-4.98782589	0.00000000
C	8.66051310	-3.58933323	0.00000000
C	8.66051273	3.58933387	0.00000000
C	8.64137560	4.98782646	0.00000000
C	8.65888786	7.82615191	0.00000000
C	8.58841760	9.23584175	0.00000000
C	9.86147154	-7.11738684	0.00000000
C	9.89720755	-5.71341997	0.00000000
C	9.85185840	-2.87235629	0.00000000
C	9.85960597	-1.46419912	0.00000000
C	9.85960581	1.46420003	0.00000000
C	9.85185810	2.87235716	0.00000000
C	9.89720703	5.71342068	0.00000000
C	9.86147096	7.11738746	0.00000000
C	11.09568204	-4.98131822	0.00000000
C	11.10916887	-3.58538965	0.00000000
C	11.02394923	-0.72648127	0.00000000
C	11.02394915	0.72648235	0.00000000
C	11.10916850	3.58539065	0.00000000
C	11.09568160	4.98131916	0.00000000
C	12.29497083	-2.81979259	0.00000000
C	12.28226286	-1.43467974	0.00000000
C	12.28226269	1.43468097	0.00000000
C	12.29497054	2.81979379	0.00000000
C	13.49724610	-0.67987991	0.00000000
C	13.49724602	0.67988131	0.00000000
H	-14.44473587	-1.23637480	0.00000000
H	-14.44473560	1.23637627	0.00000000
H	-13.26276810	-3.34311139	0.00000000
H	-13.26276745	3.34311249	0.00000000
H	-12.05144440	-5.52599823	0.00000000
H	-12.05144343	5.52599892	0.00000000
H	-10.81047170	-7.67376463	0.00000000
H	-10.81047051	7.67376488	0.00000000
H	-9.52527196	-9.81277332	0.00000000
H	-9.52527061	9.81277311	0.00000000
H	-8.88229912	-0.96227707	0.00000000
H	-8.88229889	0.96227729	0.00000000
H	-8.29171086	-11.88869067	0.00000000
H	-8.29170940	11.88869014	0.00000000
H	-7.70536874	-3.04339794	0.00000000
H	-7.70536812	3.04339781	0.00000000
H	-6.48956306	-5.14761833	0.00000000
H	-6.48956211	5.14761784	0.00000000
H	-6.15002955	-13.12492337	0.00000000
H	-6.15002836	13.12492253	0.00000000
H	-5.27400782	-7.20740043	0.00000000
H	-5.27400660	7.20739970	0.00000000
H	-3.73539553	-13.15448127	0.00000000
H	-3.60656866	-8.17029107	0.00000000
H	-3.60656779	8.17029000	0.00000000
H	-3.73539469	13.15448042	0.00000000
H	-1.24012992	-13.19733572	0.00000000
H	-1.21505452	-8.19207865	0.00000000
H	-1.21505391	8.19207761	0.00000000
H	-1.24012953	13.19733496	0.00000000
H	1.24012843	-13.19733597	0.00000000
H	1.21505368	-8.19207874	0.00000000
H	1.21505390	8.19207786	0.00000000
H	1.24012826	13.19733537	0.00000000
H	3.73539417	-13.15448204	0.00000000
H	3.60656804	-8.17029142	0.00000000
H	3.60656792	8.17029079	0.00000000
H	3.73539360	13.15448164	0.00000000
H	5.27400733	-7.20740084	0.00000000
H	5.27400667	7.20740077	0.00000000
H	6.15002852	-13.12492439	0.00000000
H	6.15002771	13.12492417	0.00000000
H	6.48956288	-5.14761849	0.00000000
H	6.48956240	5.14761869	0.00000000
H	7.70536906	-3.04339776	0.00000000
H	7.70536872	3.04339831	0.00000000

H	8.29171011	-11.88869187	0.00000000
H	8.29170911	11.88869205	0.00000000
H	8.88229998	-0.96227676	0.00000000
H	8.88229985	0.96227758	0.00000000
H	9.52527177	-9.81277416	0.00000000
H	9.52527099	9.81277459	0.00000000
H	10.81047190	-7.67376521	0.00000000
H	10.81047127	7.67376594	0.00000000
H	12.05144486	-5.52599867	0.00000000
H	12.05144436	5.52599972	0.00000000
H	13.26276884	-3.34311163	0.00000000
H	13.26276849	3.34311293	0.00000000
H	14.44473687	-1.23637492	0.00000000
H	14.44473672	1.23637646	0.00000000

7-HGQR (singlet)

C	-15.94896156	-0.68016314	0.00000000
C	-15.94896165	0.68016680	0.00000000
C	-14.74821230	-2.81859804	0.00000000
C	-14.73439156	-1.43324917	0.00000000
C	-14.73439189	1.43325293	0.00000000
C	-14.74821307	2.81860110	0.00000000
C	-13.55477788	-4.98024091	0.00000000
C	-13.56407941	-3.58509088	0.00000000
C	-13.47500100	-0.72597768	0.00000000
C	-13.47500124	0.72598192	0.00000000
C	-13.56408093	3.58509371	0.00000000
C	-13.55478032	4.98024258	0.00000000
C	-12.33075353	-7.11771023	0.00000000
C	-12.35845908	-5.71725052	0.00000000
C	-12.30436339	-2.87430028	0.00000000
C	-12.31079989	-1.46623584	0.00000000
C	-12.31080064	1.46623999	0.00000000
C	-12.30436509	2.87430381	0.00000000
C	-12.35846260	5.71725165	0.00000000
C	-12.33075817	7.11771003	0.00000000
C	-11.08957462	-9.24509533	0.00000000
C	-11.13104166	-7.84058643	0.00000000
C	-11.09830335	-4.99513703	0.00000000
C	-11.11506047	-3.59497145	0.00000000
C	-11.11506310	3.59497450	0.00000000
C	-11.09830714	4.99513900	0.00000000
C	-11.13104753	7.84058567	0.00000000
C	-11.08958162	9.24509347	0.00000000
C	-9.81297160	-11.35807788	0.00000000
C	-9.88536269	-9.94942838	0.00000000
C	-9.87632889	-7.10905491	0.00000000
C	-9.90146731	-5.71339228	0.00000000
C	-9.90147212	5.71339355	0.00000000
C	-9.87633497	7.10905499	0.00000000
C	-9.88537081	9.94942632	0.00000000
C	-9.81298074	11.35807532	0.00000000
C	-8.56080871	-13.46625781	0.00000000
C	-8.60592611	-12.03786629	0.00000000
C	-8.64052459	-9.21279916	0.00000000
C	-8.67142560	-7.82241096	0.00000000
C	-8.67143259	7.82241056	0.00000000
C	-8.64053269	9.21279786	0.00000000
C	-8.60593603	12.03786420	0.00000000
C	-8.56081935	13.46625586	0.00000000
C	-7.38269033	-14.14610795	0.00000000
C	-7.36408367	-11.29999221	0.00000000
C	-7.42376825	-9.92149249	0.00000000
C	-7.42377685	9.92149120	0.00000000
C	-7.36409316	11.29999078	0.00000000
C	-7.38270107	14.14610696	0.00000000
C	-6.12357705	-13.47024125	0.00000000
C	-6.10655305	-12.02571484	0.00000000
C	-6.10656263	12.02571403	0.00000000
C	-6.12358691	13.47024090	0.00000000
C	-4.93089558	-14.17490634	0.00000000
C	-4.88328925	-11.38764879	0.00000000
C	-4.88329750	11.38764822	0.00000000
C	-4.93090444	14.17490692	0.00000000
C	-3.67514003	-13.53265020	0.00000000
C	-3.66067057	-12.08614146	0.00000000
C	-3.66067769	12.08614112	0.00000000
C	-3.67514739	13.53265080	0.00000000
C	-2.46269187	-14.22260841	0.00000000
C	-2.44159048	-11.41689480	0.00000000
C	-2.44159565	11.41689431	0.00000000
C	-2.46269729	14.22260957	0.00000000
C	-1.22615625	-13.55546103	0.00000000
C	-1.22087383	-12.10290731	0.00000000

C	-1.22087707	12.10290709	0.00000000
C	-1.22615954	13.55546199	0.00000000
C	0.00000047	-14.23236250	0.00000000
C	0.00000031	-11.42618424	0.00000000
C	-0.00000070	11.42618380	0.00000000
C	-0.00000050	14.23236386	0.00000000
C	1.22615716	-13.55546097	0.00000000
C	1.22087457	-12.10290721	0.00000000
C	1.22087582	12.10290697	0.00000000
C	1.22615852	13.55546191	0.00000000
C	2.46269287	-14.22260817	0.00000000
C	2.44159137	-11.41689461	0.00000000
C	2.44159455	11.41689403	0.00000000
C	2.46269639	14.22260930	0.00000000
C	3.67514101	-13.53265001	0.00000000
C	3.66067160	-12.08614133	0.00000000
C	3.66067680	12.08614088	0.00000000
C	3.67514649	13.53265054	0.00000000
C	4.93089649	-14.17490606	0.00000000
C	4.88329024	-11.38764868	0.00000000
C	4.88329665	11.38764794	0.00000000
C	4.93090354	14.17490657	0.00000000
C	6.12357790	-13.47024101	0.00000000
C	6.10655395	-12.02571469	0.00000000
C	6.10656181	12.02571370	0.00000000
C	6.12358602	13.47024056	0.00000000
C	7.38269103	-14.14610776	0.00000000
C	7.36408443	-11.29999216	0.00000000
C	7.42376899	-9.92149267	0.00000000
C	7.42377612	9.92149088	0.00000000
C	7.36409233	11.29999047	0.00000000
C	7.38270013	14.14610671	0.00000000
C	8.56080932	-13.46625770	0.00000000
C	8.60592672	-12.03786623	0.00000000
C	8.64052523	-9.21279943	0.00000000
C	8.67142621	-7.82241133	0.00000000
C	8.67143204	7.82240990	0.00000000
C	8.64053203	9.21279744	0.00000000
C	8.60593519	12.03786393	0.00000000
C	8.56081846	13.46625563	0.00000000
C	9.81297216	-11.35807796	0.00000000
C	9.88536329	-9.94942856	0.00000000
C	9.87632936	-7.10905526	0.00000000
C	9.90146773	-5.71339269	0.00000000
C	9.90147179	5.71339234	0.00000000
C	9.87633452	7.10905406	0.00000000
C	9.88537025	9.94942591	0.00000000
C	9.81298006	11.35807504	0.00000000
C	11.08957512	-9.24509546	0.00000000
C	11.13104209	-7.84058663	0.00000000
C	11.09830374	-4.99513741	0.00000000
C	11.11506093	-3.59497180	0.00000000
C	11.11506318	3.59497268	0.00000000
C	11.09830702	4.99513750	0.00000000
C	11.13104718	7.84058472	0.00000000
C	11.08958120	9.24509278	0.00000000
C	12.33075386	-7.11771040	0.00000000
C	12.35845943	-5.71725079	0.00000000
C	12.30436385	-2.87430076	0.00000000
C	12.31080028	-1.46623660	0.00000000
C	12.31080096	1.46623823	0.00000000
C	12.30436537	2.87430193	0.00000000
C	12.35846257	5.71725016	0.00000000
C	12.33075799	7.11770880	0.00000000
C	13.55477803	-4.98024117	0.00000000
C	13.56407961	-3.58509138	0.00000000
C	13.47500107	-0.72597879	0.00000000
C	13.47500131	0.72598042	0.00000000
C	13.56408107	3.58509193	0.00000000
C	13.55478031	4.98024086	0.00000000
C	14.74821222	-2.81859865	0.00000000
C	14.73439138	-1.43325010	0.00000000
C	14.73439176	1.43325127	0.00000000
C	14.74821307	2.81859929	0.00000000
C	15.94896127	-0.68016434	0.00000000
C	15.94896140	0.68016533	0.00000000
H	-16.89622160	-1.23705748	0.00000000
H	-16.89622179	1.23706076	0.00000000
H	-15.71658380	-3.34087380	0.00000000
H	-15.71658465	3.34087625	0.00000000
H	-14.51221801	-5.52199270	0.00000000
H	-14.51222044	5.52199370	0.00000000
H	-13.28305940	-7.66843893	0.00000000

H	-13.28306395	7.66843816	0.00000000
H	-12.03671048	-9.80466412	0.00000000
H	-12.03671741	9.80466181	0.00000000
H	-11.33316619	-0.96479343	0.00000000
H	-11.33316695	0.96479766	0.00000000
H	-10.74909003	-11.93621518	0.00000000
H	-10.74909921	11.93621226	0.00000000
H	-10.15843237	-3.05162121	0.00000000
H	-10.15843502	3.05162462	0.00000000
H	-9.51664913	-14.00826849	0.00000000
H	-9.51665994	14.00826627	0.00000000
H	-8.94918470	-5.16272000	0.00000000
H	-8.94918944	5.16272182	0.00000000
H	-7.72330369	-7.26437522	0.00000000
H	-7.72331040	7.26437550	0.00000000
H	-7.37355492	-15.24488215	0.00000000
H	-7.37356607	15.24488145	0.00000000
H	-6.50105803	-9.32494269	0.00000000
H	-6.50106602	9.32494212	0.00000000
H	-4.96284854	-15.27467181	0.00000000
H	-4.82874788	-10.29029700	0.00000000
H	-4.82875535	10.29029620	0.00000000
H	-4.96285736	15.27467288	0.00000000
H	-2.47286046	-15.32262335	0.00000000
H	-2.43315851	-10.31679575	0.00000000
H	-2.43316334	10.31679475	0.00000000
H	-2.47286575	15.32262519	0.00000000
H	0.00000055	-15.33242452	0.00000000
H	0.00000032	-10.32616894	0.00000000
H	-0.00000069	10.32616789	0.00000000
H	-0.00000044	15.33242663	0.00000000
H	2.47286144	-15.32262308	0.00000000
H	2.43315931	-10.31679554	0.00000000
H	2.43316212	10.31679443	0.00000000
H	2.47286485	15.32262489	0.00000000
H	4.96284945	-15.27467148	0.00000000
H	4.82874873	-10.29029693	0.00000000
H	4.82875431	10.29029592	0.00000000
H	4.96285648	15.27467250	0.00000000
H	6.50105889	-9.32494281	0.00000000
H	6.50106542	9.32494164	0.00000000
H	7.37355554	-15.24488189	0.00000000
H	7.37356504	15.24488116	0.00000000
H	7.72330430	-7.26437573	0.00000000
H	7.72330982	7.26437490	0.00000000
H	8.94918518	-5.16272039	0.00000000
H	8.94918913	5.16272055	0.00000000
H	9.51664970	-14.00826832	0.00000000
H	9.51665905	14.00826601	0.00000000
H	10.15843287	-3.05162155	0.00000000
H	10.15843508	3.05162279	0.00000000
H	10.74909049	-11.93621527	0.00000000
H	10.74909851	11.93621198	0.00000000
H	11.33316655	-0.96479441	0.00000000
H	11.33316720	0.96479616	0.00000000
H	12.03671094	-9.80466416	0.00000000
H	12.03671701	9.80466106	0.00000000
H	13.28305964	-7.66843905	0.00000000
H	13.28306375	7.66843693	0.00000000
H	14.51221801	-5.52199297	0.00000000
H	14.51222033	5.52199206	0.00000000
H	15.71658362	-3.34087431	0.00000000
H	15.71658457	3.34087440	0.00000000
H	16.89622118	-1.23705865	0.00000000
H	16.89622145	1.23705926	0.00000000

8-HGQR (singlet)

C	-18.40280923	-0.68036238	0.00000000
C	-18.40280903	0.68037654	0.00000000
C	-17.20243740	-2.81792732	0.00000000
C	-17.18829051	-1.43177691	0.00000000
C	-17.18829025	1.43179072	0.00000000
C	-17.20243697	2.81794043	0.00000000
C	-16.01252498	-4.97984925	0.00000000
C	-16.01971974	-3.58396593	0.00000000
C	-15.92885013	-0.72544377	0.00000000
C	-15.92885006	0.72545768	0.00000000
C	-16.01971978	3.58397837	0.00000000
C	-16.01252530	4.97986042	0.00000000
C	-14.79503566	-7.11874851	0.00000000
C	-14.81858793	-5.71871350	0.00000000
C	-14.75904969	-2.87505493	0.00000000
C	-14.76414539	-1.46782490	0.00000000
C	-14.76414558	1.46783827	0.00000000

C	-14.75905028	2.87506767	0.00000000
C	-14.81858916	5.71872358	0.00000000
C	-14.79503740	7.11875693	0.00000000
C	-13.56361116	-9.24803844	0.00000000
C	-13.59758798	-7.84693277	0.00000000
C	-13.55599334	-4.99937706	0.00000000
C	-13.57042468	-3.59957667	0.00000000
C	-13.57042611	3.59958853	0.00000000
C	-13.55599535	4.99938765	0.00000000
C	-13.59759091	7.84693982	0.00000000
C	-13.56361474	9.24804374	0.00000000
C	-12.31719788	-11.36989480	0.00000000
C	-12.36190615	-9.96656405	0.00000000
C	-12.33876034	-7.11891742	0.00000000
C	-12.36140058	-5.72174800	0.00000000
C	-12.36140370	5.72175733	0.00000000
C	-12.33876416	7.11892508	0.00000000
C	-12.36191100	9.96656806	0.00000000
C	-12.31720330	11.36989732	0.00000000
C	-11.03801868	-13.47948921	0.00000000
C	-11.11107525	-12.07233184	0.00000000
C	-11.10865353	-9.23105278	0.00000000
C	-11.13905078	-7.83526787	0.00000000
C	-11.13905580	7.83527416	0.00000000
C	-11.10865925	9.23105736	0.00000000
C	-11.11108178	12.07233327	0.00000000
C	-11.03802552	13.47948958	0.00000000
C	-9.78605011	-15.58656813	0.00000000
C	-9.82996085	-14.15907114	0.00000000
C	-9.86761138	-11.33355562	0.00000000
C	-9.90264877	-9.94182224	0.00000000
C	-9.90265552	9.94182564	0.00000000
C	-9.86761863	11.33355753	0.00000000
C	-9.82996867	14.15907092	0.00000000
C	-9.78605839	15.58656735	0.00000000
C	-8.60746906	-16.26639220	0.00000000
C	-8.58907195	-13.42037796	0.00000000
C	-8.65070781	-12.04037602	0.00000000
C	-8.65071573	12.04037703	0.00000000
C	-8.58908039	13.42037789	0.00000000
C	-8.60747807	16.26639136	0.00000000
C	-7.34998525	-15.58946098	0.00000000
C	-7.33230827	-14.14534229	0.00000000
C	-7.33231725	14.14534210	0.00000000
C	-7.34999461	15.58946042	0.00000000
C	-6.15672016	-16.29467131	0.00000000
C	-6.10699579	-13.50777266	0.00000000
C	-6.10700526	13.50777276	0.00000000
C	-6.15673005	16.29467090	0.00000000
C	-4.90215241	-15.65328147	0.00000000
C	-4.88566871	-14.20688630	0.00000000
C	-4.88567860	14.20688641	0.00000000
C	-4.90216241	15.65328139	0.00000000
C	-3.69014752	-16.34540718	0.00000000
C	-3.66358112	-13.54011831	0.00000000
C	-3.66359117	13.54011858	0.00000000
C	-3.69015775	16.34540725	0.00000000
C	-2.45323651	-15.68122007	0.00000000
C	-2.44430024	-14.22794008	0.00000000
C	-2.44431042	14.22794033	0.00000000
C	-2.45324673	15.68122029	0.00000000
C	-1.22963063	-16.36160840	0.00000000
C	-1.22089647	-13.55530670	0.00000000
C	-1.22090662	13.55530699	0.00000000
C	-1.22964084	16.36160873	0.00000000
C	0.00000652	-15.68941673	0.00000000
C	0.00000639	-14.23505537	0.00000000
C	-0.00000368	14.23505567	0.00000000
C	-0.00000354	15.68941711	0.00000000
C	1.22964374	-16.36160810	0.00000000
C	1.22090906	-13.55530651	0.00000000
C	1.22089920	13.55530676	0.00000000
C	1.22963385	16.36160857	0.00000000
C	2.45324926	-15.68121952	0.00000000
C	2.44431275	-14.22793967	0.00000000
C	2.44430314	14.22793998	0.00000000
C	2.45323966	15.68122001	0.00000000
C	3.69016007	-16.34540625	0.00000000
C	3.66359311	-13.54011775	0.00000000
C	3.66358386	13.54011796	0.00000000
C	3.69015075	16.34540687	0.00000000
C	4.90216423	-15.65328022	0.00000000
C	4.88568025	-14.20688541	0.00000000

C	4.88567143	14.20688577	0.00000000
C	4.90215537	15.65328089	0.00000000
C	6.15673160	-16.29466952	0.00000000
C	6.10700639	-13.50777165	0.00000000
C	6.10699813	13.50777193	0.00000000
C	6.15672308	16.29467048	0.00000000
C	7.34999564	-15.58945891	0.00000000
C	7.33231791	-14.14534084	0.00000000
C	7.33231035	14.14534133	0.00000000
C	7.34998777	15.58945987	0.00000000
C	8.60747896	-16.26638961	0.00000000
C	8.58908063	-13.42037658	0.00000000
C	8.65071548	-12.04037617	0.00000000
C	8.65070887	12.04037522	0.00000000
C	8.58907361	13.42037679	0.00000000
C	8.60747160	16.26639073	0.00000000
C	9.78605884	-15.58656562	0.00000000
C	9.82996877	-14.15906942	0.00000000
C	9.86761800	-11.33355677	0.00000000
C	9.90265452	-9.94182522	0.00000000
C	9.90264889	9.94182172	0.00000000
C	9.86761201	11.33355475	0.00000000
C	9.82996250	14.15906965	0.00000000
C	9.78605222	15.58656649	0.00000000
C	11.03802522	-13.47948822	0.00000000
C	11.11108111	-12.07233223	0.00000000
C	11.10865792	-9.23105704	0.00000000
C	11.13905414	-7.83527410	0.00000000
C	11.13904999	7.83526763	0.00000000
C	11.10865324	9.23105223	0.00000000
C	11.11107593	12.07233059	0.00000000
C	11.03801988	13.47948769	0.00000000
C	12.31720234	-11.36989644	0.00000000
C	12.36190970	-9.96656749	0.00000000
C	12.33876224	-7.11892506	0.00000000
C	12.36140149	-5.72175743	0.00000000
C	12.36139902	5.72174791	0.00000000
C	12.33875920	7.11891714	0.00000000
C	12.36190594	9.96656313	0.00000000
C	12.31719817	11.36989357	0.00000000
C	13.56361317	-9.24804325	0.00000000
C	13.59758906	-7.84693958	0.00000000
C	13.55599301	-4.99938767	0.00000000
C	13.57042366	-3.59958859	0.00000000
C	13.57042262	3.59957651	0.00000000
C	13.55599155	4.99937681	0.00000000
C	13.59758696	7.84693212	0.00000000
C	13.56361058	9.24803750	0.00000000
C	14.79503532	-7.11875665	0.00000000
C	14.81858688	-5.71872346	0.00000000
C	14.75904777	-2.87506771	0.00000000
C	14.76414311	-1.46783841	0.00000000
C	14.76414306	1.46782477	0.00000000
C	14.75904747	2.87505466	0.00000000
C	14.81858624	5.71871298	0.00000000
C	14.79503431	7.11874779	0.00000000
C	16.01252286	-4.97986028	0.00000000
C	16.01971723	-3.58397836	0.00000000
C	15.92884748	-0.72545785	0.00000000
C	15.92884762	0.72544359	0.00000000
C	16.01971754	3.58396551	0.00000000
C	16.01252303	4.97984866	0.00000000
C	17.20243436	-2.81794052	0.00000000
C	17.18828762	-1.43179091	0.00000000
C	17.18828801	1.43177663	0.00000000
C	17.20243505	2.81792692	0.00000000
C	18.40280639	-0.68037682	0.00000000
C	18.40280666	0.68036210	0.00000000
H	-19.34974163	-1.23775330	0.00000000
H	-19.34974134	1.23776737	0.00000000
H	-18.17110927	-3.33965681	0.00000000
H	-18.17110866	3.33966960	0.00000000
H	-16.97107575	-5.51965111	0.00000000
H	-16.97107576	5.51966178	0.00000000
H	-15.74911491	-7.66642446	0.00000000
H	-15.74911615	7.66643244	0.00000000
H	-14.51393194	-9.80220853	0.00000000
H	-14.51393492	9.80221349	0.00000000
H	-13.78636526	-0.96660031	0.00000000
H	-13.78636579	0.96661322	0.00000000
H	-13.26301794	-11.93170118	0.00000000
H	-13.26302276	11.93170355	0.00000000
H	-12.61278563	-3.05797724	0.00000000

H	-12.61278754	3.05798902	0.00000000
H	-11.97367469	-14.05838324	0.00000000
H	-11.97368105	14.05838355	0.00000000
H	-11.40751274	-5.17384298	0.00000000
H	-11.40751641	5.17385248	0.00000000
H	-10.74207209	-16.12819473	0.00000000
H	-10.74208016	16.12819370	0.00000000
H	-10.18859452	-7.28142997	0.00000000
H	-10.18860003	7.28143664	0.00000000
H	-8.95572232	-9.38172786	0.00000000
H	-8.95572942	9.38173173	0.00000000
H	-8.59750063	-17.36512734	0.00000000
H	-8.59750966	17.36512631	0.00000000
H	-7.72826184	-11.44335822	0.00000000
H	-7.72826984	11.44335981	0.00000000
H	-6.18917272	-17.39442805	0.00000000
H	-6.05229446	-12.41039556	0.00000000
H	-6.05230365	12.41039595	0.00000000
H	-6.18918271	17.39442751	0.00000000
H	-3.70254054	-17.44540842	0.00000000
H	-3.65314774	-12.44001871	0.00000000
H	-3.65315757	12.44001911	0.00000000
H	-3.70255081	17.44540845	0.00000000
H	-1.23326209	-17.46167639	0.00000000
H	-1.21758898	-12.45528652	0.00000000
H	-1.21759898	12.45528682	0.00000000
H	-1.23327228	17.46167674	0.00000000
H	1.23327538	-17.46167606	0.00000000
H	1.21760131	-12.45528637	0.00000000
H	1.21759157	12.45528653	0.00000000
H	1.23326553	17.46167661	0.00000000
H	3.70255331	-17.44540739	0.00000000
H	3.65315935	-12.44001831	0.00000000
H	3.65315024	12.44001831	0.00000000
H	3.70254398	17.44540814	0.00000000
H	6.18918445	-17.39442608	0.00000000
H	6.05230478	-12.41039489	0.00000000
H	6.05229665	12.41039483	0.00000000
H	6.18917586	17.39442723	0.00000000
H	7.72826946	-11.44335925	0.00000000
H	7.72826267	11.44335782	0.00000000
H	8.59751077	-17.36512451	0.00000000
H	8.59750350	17.36512586	0.00000000
H	8.95572841	-9.38173144	0.00000000
H	8.95572235	9.38172758	0.00000000
H	10.18859835	-7.28143669	0.00000000
H	10.18859366	7.28142992	0.00000000
H	10.74208064	-16.12819179	0.00000000
H	10.74207430	16.12819284	0.00000000
H	11.40751412	-5.17385273	0.00000000
H	11.40751106	5.17384311	0.00000000
H	11.97368083	-14.05838202	0.00000000
H	11.97367604	14.05838147	0.00000000
H	12.61278499	-3.05798919	0.00000000
H	12.61278344	3.05797726	0.00000000
H	13.26302188	-11.93170253	0.00000000
H	13.26301838	11.93169970	0.00000000
H	13.78636329	-0.96661335	0.00000000
H	13.78636287	0.96660024	0.00000000
H	14.51393346	-9.80221287	0.00000000
H	14.51393151	9.80220737	0.00000000
H	15.74911420	-7.66643202	0.00000000
H	15.74911373	7.66642350	0.00000000
H	16.97107340	-5.51966159	0.00000000
H	16.97107393	5.51965035	0.00000000
H	18.17110605	-3.33966975	0.00000000
H	18.17110696	3.33965640	0.00000000
H	19.34973868	-1.23776774	0.00000000
H	19.34973910	1.23775301	0.00000000

9-HGQR (singlet)

C	-20.85842851	-0.68040719	0.00000000
C	-20.85842866	0.68040932	0.00000000
C	-19.65811684	-2.81745563	0.00000000
C	-19.64370151	-1.43066389	0.00000000
C	-19.64370184	1.43066628	0.00000000
C	-19.65811747	2.81745798	0.00000000
C	-18.47049883	-4.97974571	0.00000000
C	-18.47635392	-3.58282745	0.00000000
C	-18.38449792	-0.72522903	0.00000000
C	-18.38449809	0.72523172	0.00000000
C	-18.47635477	3.58283005	0.00000000
C	-18.47050000	4.97974824	0.00000000
C	-17.25739434	-7.11998568	0.00000000

C	-17.27857928	-5.71903694	0.00000000
C	-17.21542323	-2.87554524	0.00000000
C	-17.21932630	-1.46901154	0.00000000
C	-17.21932668	1.46901447	0.00000000
C	-17.21542397	2.87554813	0.00000000
C	-17.27858067	5.71903966	0.00000000
C	-17.25739603	7.11998829	0.00000000
C	-16.03250177	-9.25116924	0.00000000
C	-16.06250424	-7.85039698	0.00000000
C	-16.01486504	-5.00211560	0.00000000
C	-16.02698733	-3.60327468	0.00000000
C	-16.02698829	3.60327777	0.00000000
C	-16.01486636	5.00211860	0.00000000
C	-16.06250614	7.85039973	0.00000000
C	-16.03250389	9.25117186	0.00000000
C	-14.79523056	-11.37503584	0.00000000
C	-14.83299600	-9.97507847	0.00000000
C	-14.80125498	-7.12533227	0.00000000
C	-14.82120614	-5.72865461	0.00000000
C	-14.82120768	5.72865773	0.00000000
C	-14.80125684	7.12533526	0.00000000
C	-14.83299832	9.97508119	0.00000000
C	-14.79523305	11.37503846	0.00000000
C	-13.54468508	-13.49315897	0.00000000
C	-13.59125282	-12.09140314	0.00000000
C	-13.57585935	-9.24288762	0.00000000
C	-13.60385837	-7.84570545	0.00000000
C	-13.60386044	7.84570850	0.00000000
C	-13.57586167	9.24289052	0.00000000
C	-13.59125546	12.09140579	0.00000000
C	-13.54468776	13.49316153	0.00000000
C	-12.26341603	-15.60043947	0.00000000
C	-12.33675744	-14.19448344	0.00000000
C	-12.33982486	-11.35321340	0.00000000
C	-12.37490544	-9.95615126	0.00000000
C	-12.37490792	9.95615414	0.00000000
C	-12.33982752	11.35321614	0.00000000
C	-12.33676020	14.19448596	0.00000000
C	-12.26341871	15.60044193	0.00000000
C	-11.01143737	-17.70683628	0.00000000
C	-11.05449588	-16.27975420	0.00000000
C	-11.09481726	-13.45425838	0.00000000
C	-11.13322817	-12.06108263	0.00000000
C	-11.13323094	12.06108529	0.00000000
C	-11.09482010	13.45426091	0.00000000
C	-11.05449857	16.27975655	0.00000000
C	-11.01143987	17.70683859	0.00000000
C	-9.83266073	-18.38643522	0.00000000
C	-9.81469512	-15.54047689	0.00000000
C	-9.87770785	-14.15937090	0.00000000
C	-9.87771071	14.15937326	0.00000000
C	-9.81469793	15.54047915	0.00000000
C	-9.83266316	18.38643737	0.00000000
C	-8.57625410	-17.70843447	0.00000000
C	-8.55814441	-16.26495649	0.00000000
C	-8.55814718	16.26495855	0.00000000
C	-8.57625664	17.70843649	0.00000000
C	-7.38248602	-18.41401454	0.00000000
C	-7.33145627	-15.62750408	0.00000000
C	-7.33145922	15.62750590	0.00000000
C	-7.38248842	18.41401632	0.00000000
C	-6.12908656	-17.77293699	0.00000000
C	-6.11120596	-16.32713048	0.00000000
C	-6.11120881	16.32713202	0.00000000
C	-6.12908908	17.77293852	0.00000000
C	-4.91684795	-18.46666659	0.00000000
C	-4.88645294	-15.66204784	0.00000000
C	-4.88645600	15.66204904	0.00000000
C	-4.91685027	18.46666779	0.00000000
C	-3.68064021	-17.80435934	0.00000000
C	-3.66909041	-16.35131624	0.00000000
C	-3.66909329	16.35131709	0.00000000
C	-3.68064269	17.80436020	0.00000000
C	-2.45748939	-18.48728757	0.00000000
C	-2.44249794	-15.68159993	0.00000000
C	-2.44250103	15.68160038	0.00000000
C	-2.45749163	18.48728804	0.00000000
C	-1.22721564	-17.81845026	0.00000000
C	-1.22335400	-16.36346419	0.00000000
C	-1.22335686	16.36346425	0.00000000
C	-1.22721807	17.81845031	0.00000000
C	-0.00000061	-18.49408383	0.00000000
C	0.00000039	-15.68788326	0.00000000

C	-0.00000268	15.68788289	0.00000000
C	-0.00000278	18.49408346	0.00000000
C	1.22721493	-17.81845113	0.00000000
C	1.22335431	-16.36346501	0.00000000
C	1.22335151	16.36346423	0.00000000
C	1.22721256	17.81845035	0.00000000
C	2.45748836	-18.48728936	0.00000000
C	2.44249870	-15.68160157	0.00000000
C	2.44249574	15.68160039	0.00000000
C	2.45748622	18.48728820	0.00000000
C	3.68063977	-17.80436181	0.00000000
C	3.66909077	-16.35131857	0.00000000
C	3.66908807	16.35131702	0.00000000
C	3.68063745	17.80436027	0.00000000
C	4.91684733	-18.46666993	0.00000000
C	4.88645370	-15.66205086	0.00000000
C	4.88645087	15.66204897	0.00000000
C	4.91684520	18.46666804	0.00000000
C	6.12908660	-17.77294092	0.00000000
C	6.11120649	-16.32713415	0.00000000
C	6.11120389	16.32713195	0.00000000
C	6.12908431	17.77293873	0.00000000
C	7.38248600	-18.41401899	0.00000000
C	7.33145743	-15.62750825	0.00000000
C	7.33145476	15.62750581	0.00000000
C	7.38248383	18.41401658	0.00000000
C	8.57625463	-17.70843952	0.00000000
C	8.55814562	-16.26496143	0.00000000
C	8.55814311	16.26495878	0.00000000
C	8.57625233	17.70843689	0.00000000
C	9.83266115	-18.38644067	0.00000000
C	9.81469668	-15.54048195	0.00000000
C	9.87770973	-14.15937552	0.00000000
C	9.87770717	14.15937268	0.00000000
C	9.81469413	15.54047915	0.00000000
C	9.83265891	18.38643794	0.00000000
C	11.01143836	-17.70684195	0.00000000
C	11.05449747	-16.27975965	0.00000000
C	11.09481934	-13.45426258	0.00000000
C	11.13323045	-12.06108652	0.00000000
C	11.13322802	12.06108364	0.00000000
C	11.09481680	13.45425971	0.00000000
C	11.05449498	16.27975681	0.00000000
C	11.01143605	17.70683912	0.00000000
C	12.26341812	-15.60044446	0.00000000
C	12.33675961	-14.19448784	0.00000000
C	12.33982726	-11.35321676	0.00000000
C	12.37490802	-9.95615428	0.00000000
C	12.37490583	9.95615145	0.00000000
C	12.33982490	11.35321395	0.00000000
C	12.33675707	14.19448501	0.00000000
C	12.26341560	15.60044161	0.00000000
C	13.54468766	-13.49316282	0.00000000
C	13.59125543	-12.09140644	0.00000000
C	13.57586216	-9.24289027	0.00000000
C	13.60386138	-7.84570779	0.00000000
C	13.60385955	7.84570512	0.00000000
C	13.57586009	9.24288760	0.00000000
C	13.59125302	12.09140376	0.00000000
C	13.54468517	13.49316011	0.00000000
C	14.79523334	-11.37503873	0.00000000
C	14.83299886	-9.97508114	0.00000000
C	14.80125820	-7.12533452	0.00000000
C	14.82120968	-5.72865671	0.00000000
C	14.82120827	5.72865425	0.00000000
C	14.80125650	7.12533205	0.00000000
C	14.83299670	9.97507865	0.00000000
C	14.79523103	11.37503622	0.00000000
C	16.03250466	-9.25117180	0.00000000
C	16.06250733	-7.85039951	0.00000000
C	16.01486855	-5.00211774	0.00000000
C	16.02699116	-3.60327691	0.00000000
C	16.02699025	3.60327470	0.00000000
C	16.01486732	5.00211553	0.00000000
C	16.06250553	7.85039728	0.00000000
C	16.03250263	9.25116954	0.00000000
C	17.25739762	-7.11998827	0.00000000
C	17.27858273	-5.71903934	0.00000000
C	17.21542689	-2.87554749	0.00000000
C	17.21933017	-1.46901370	0.00000000
C	17.21932981	1.46901179	0.00000000
C	17.21542617	2.87554557	0.00000000
C	17.27858135	5.71903740	0.00000000

C	17.25739596	7.11998631	0.00000000
C	18.47050265	-4.97974815	0.00000000
C	18.47635781	-3.58282954	0.00000000
C	18.38450187	-0.72523117	0.00000000
C	18.38450169	0.72522954	0.00000000
C	18.47635693	3.58282791	0.00000000
C	18.47050144	4.97974651	0.00000000
C	19.65812085	-2.81745756	0.00000000
C	19.64370558	-1.43066570	0.00000000
C	19.64370523	1.43066438	0.00000000
C	19.65812016	2.81745624	0.00000000
C	20.85843231	-0.68040880	0.00000000
C	20.85843215	0.68040778	0.00000000
H	-21.80513493	-1.23819657	0.00000000
H	-21.80513520	1.23819847	0.00000000
H	-20.62697730	-3.33885668	0.00000000
H	-20.62697804	3.33885877	0.00000000
H	-19.42985743	-5.51814957	0.00000000
H	-19.42985870	5.51815184	0.00000000
H	-18.21276588	-7.66545296	0.00000000
H	-18.21276763	7.66545535	0.00000000
H	-16.98459005	-9.80235327	0.00000000
H	-16.98459219	9.80235573	0.00000000
H	-16.24146385	-0.96788239	0.00000000
H	-16.24146413	0.96788552	0.00000000
H	-15.74404343	-11.93183005	0.00000000
H	-15.74404592	11.93183256	0.00000000
H	-15.06859178	-3.06296436	0.00000000
H	-15.06859266	3.06296765	0.00000000
H	-14.48949858	-14.05669227	0.00000000
H	-14.48950122	14.05669482	0.00000000
H	-13.86615148	-5.18272316	0.00000000
H	-13.86615297	5.18272646	0.00000000
H	-13.19872642	-16.17990829	0.00000000
H	-13.19872903	16.17991081	0.00000000
H	-12.65180322	-7.29457166	0.00000000
H	-12.65180527	7.29457487	0.00000000
H	-11.96759402	-18.24823757	0.00000000
H	-11.96759642	18.24824001	0.00000000
H	-11.42581787	-9.39990909	0.00000000
H	-11.42582035	9.39991209	0.00000000
H	-10.18723903	-11.49935781	0.00000000
H	-10.18724182	11.49936052	0.00000000
H	-9.82195870	-19.48516949	0.00000000
H	-9.82196100	19.48517162	0.00000000
H	-8.95546134	-13.56198873	0.00000000
H	-8.95546427	13.56199106	0.00000000
H	-7.41514621	-19.51377500	0.00000000
H	-7.27680245	-14.53009379	0.00000000
H	-7.27680558	14.53009563	0.00000000
H	-7.41514837	19.51377678	0.00000000
H	-4.93079056	-19.56666813	0.00000000
H	-4.87459608	-14.56193655	0.00000000
H	-4.87459945	14.56193777	0.00000000
H	-4.93079256	19.56666932	0.00000000
H	-2.46371396	-19.58736763	0.00000000
H	-2.43686224	-14.58155710	0.00000000
H	-2.43686568	14.58155756	0.00000000
H	-2.46371582	19.58736809	0.00000000
H	-0.00000092	-19.59418448	0.00000000
H	0.00000081	-14.58783317	0.00000000
H	-0.00000270	14.58783280	0.00000000
H	-0.00000273	19.59418412	0.00000000
H	2.46371232	-19.58736949	0.00000000
H	2.43686372	-14.58155872	0.00000000
H	2.43686042	14.58155755	0.00000000
H	2.46371054	19.58736833	0.00000000
H	4.93078930	-19.56667154	0.00000000
H	4.87459757	-14.56193951	0.00000000
H	4.87459445	14.56193763	0.00000000
H	4.93078748	19.56666966	0.00000000
H	7.41514550	-19.51377950	0.00000000
H	7.27680399	-14.53009784	0.00000000
H	7.27680115	14.53009540	0.00000000
H	7.41514354	19.51377709	0.00000000
H	8.95546301	-13.56199354	0.00000000
H	8.95546042	13.56199073	0.00000000
H	9.82195879	-19.48517495	0.00000000
H	9.82195663	19.48517223	0.00000000
H	10.18724123	-11.49936182	0.00000000
H	10.18723884	11.49935885	0.00000000
H	11.42582045	-9.39991215	0.00000000
H	11.42581833	9.39990919	0.00000000

H	11.96759501	-18.24824346	0.00000000
H	11.96759277	18.24824052	0.00000000
H	12.65180630	-7.29457395	0.00000000
H	12.65180455	7.29457110	0.00000000
H	13.19872873	-16.17991322	0.00000000
H	13.19872624	16.17991035	0.00000000
H	13.86615514	-5.18272516	0.00000000
H	13.86615384	5.18272249	0.00000000
H	14.48950134	-14.05669612	0.00000000
H	14.48949882	14.05669349	0.00000000
H	15.06859579	-3.06296636	0.00000000
H	15.06859500	3.06296393	0.00000000
H	15.74404627	-11.93183301	0.00000000
H	15.74404390	11.93183063	0.00000000
H	16.24146779	-0.96788431	0.00000000
H	16.24146754	0.96788215	0.00000000
H	16.98459298	-9.80235587	0.00000000
H	16.98459086	9.80235380	0.00000000
H	18.21276926	-7.66545556	0.00000000
H	18.21276750	7.66545382	0.00000000
H	19.42986133	-5.51815217	0.00000000
H	19.42986000	5.51815078	0.00000000
H	20.62698122	-3.33885897	0.00000000
H	20.62698039	3.33885790	0.00000000
H	21.80513880	-1.23819812	0.00000000
H	21.80513849	1.23819734	0.00000000

10-HGQR (singlet)

C	-23.31536584	-0.68034118	0.00000000
C	-23.31536585	0.68034077	0.00000000
C	-22.11517649	-2.81698465	0.00000000
C	-22.10034997	-1.42995804	0.00000000
C	-22.10034994	1.42995759	0.00000000
C	-22.11517642	2.81698421	0.00000000
C	-20.92931340	-4.97962252	0.00000000
C	-20.93399192	-3.58204656	0.00000000
C	-20.84124199	-0.72524635	0.00000000
C	-20.84124198	0.72524586	0.00000000
C	-20.93399182	3.58204612	0.00000000
C	-20.92931328	4.97962211	0.00000000
C	-19.71936896	-7.12104588	0.00000000
C	-19.73883302	-5.71907703	0.00000000
C	-19.67291777	-2.87608440	0.00000000
C	-19.67592087	-1.46983187	0.00000000
C	-19.67592084	1.46983136	0.00000000
C	-19.67291767	2.87608391	0.00000000
C	-19.73883285	5.71907660	0.00000000
C	-19.71936875	7.12104550	0.00000000
C	-18.49904860	-9.25395943	0.00000000
C	-18.52663769	-7.85228642	0.00000000
C	-18.47454768	-5.00417689	0.00000000
C	-18.48469562	-3.60606317	0.00000000
C	-18.48469547	3.60606267	0.00000000
C	-18.47454749	5.00417642	0.00000000
C	-18.52663744	7.85228605	0.00000000
C	-18.49904832	9.25395913	0.00000000
C	-17.26807629	-11.37986791	0.00000000
C	-17.30212545	-9.98021191	0.00000000
C	-17.26410949	-7.12979896	0.00000000
C	-17.28138599	-5.73411022	0.00000000
C	-17.28138575	5.73410976	0.00000000
C	-17.26410923	7.12979856	0.00000000
C	-17.30212512	9.98021163	0.00000000
C	-17.26807593	11.37986770	0.00000000
C	-16.02602345	-13.49990486	0.00000000
C	-16.06620769	-12.10144648	0.00000000
C	-16.04257578	-9.25088812	0.00000000
C	-16.06778964	-7.85428632	0.00000000
C	-16.06778933	7.85428596	0.00000000
C	-16.04257544	9.25088782	0.00000000
C	-16.06620729	12.10144630	0.00000000
C	-16.02602302	13.49990474	0.00000000
C	-14.77203081	-15.61524960	0.00000000
C	-14.81998426	-14.21469738	0.00000000
C	-14.81103115	-11.36622677	0.00000000
C	-14.84388741	-9.96792050	0.00000000
C	-14.84388701	9.96792024	0.00000000
C	-14.81103071	11.36622658	0.00000000
C	-14.81998380	14.21469730	0.00000000
C	-14.77203032	15.61524956	0.00000000
C	-13.48889590	-17.72064319	0.00000000
C	-13.56268453	-16.31538390	0.00000000
C	-13.57033582	-13.47446711	0.00000000
C	-13.60925427	-12.07613788	0.00000000

C	-13.60925379	12.07613772	0.00000000
C	-13.57033532	13.47446700	0.00000000
C	-13.56268401	16.31538389	0.00000000
C	-13.48889538	17.72064320	0.00000000
C	-12.23670892	-19.82632943	0.00000000
C	-12.27937056	-18.39933320	0.00000000
C	-12.32208096	-15.57392816	0.00000000
C	-12.36309258	-14.17980994	0.00000000
C	-12.36309206	14.17980986	0.00000000
C	-12.32208043	15.57392812	0.00000000
C	-12.27937002	18.39933323	0.00000000
C	-12.23670838	19.82632946	0.00000000
C	-11.05789554	-20.50556312	0.00000000
C	-11.04045567	-17.65939809	0.00000000
C	-11.10452989	-16.27776008	0.00000000
C	-11.10452934	16.27776007	0.00000000
C	-11.04045510	17.65939812	0.00000000
C	-11.05789500	20.50556316	0.00000000
C	-9.80209536	-19.82664392	0.00000000
C	-9.78368151	-18.38359370	0.00000000
C	-9.78368094	18.38359373	0.00000000
C	-9.80209479	19.82664396	0.00000000
C	-8.60821648	-20.53247459	0.00000000
C	-8.55631002	-17.74619262	0.00000000
C	-8.55630944	17.74619264	0.00000000
C	-8.60821591	20.53247461	0.00000000
C	-7.35550707	-19.89151814	0.00000000
C	-7.33660347	-18.44624424	0.00000000
C	-7.33660290	18.44624427	0.00000000
C	-7.35550650	19.89151817	0.00000000
C	-6.14324485	-20.58647732	0.00000000
C	-6.11004698	-17.78234476	0.00000000
C	-6.11004642	17.78234479	0.00000000
C	-6.14324428	20.58647733	0.00000000
C	-4.90769961	-19.92537152	0.00000000
C	-4.89420680	-18.47284169	0.00000000
C	-4.89420626	18.47284172	0.00000000
C	-4.90769906	19.92537154	0.00000000
C	-3.68454979	-20.61028086	0.00000000
C	-3.66490316	-17.80522581	0.00000000
C	-3.66490266	17.80522583	0.00000000
C	-3.68454927	20.61028086	0.00000000
C	-2.45467294	-19.94369886	0.00000000
C	-2.44791259	-18.48889101	0.00000000
C	-2.44791213	18.48889103	0.00000000
C	-2.45467247	19.94369884	0.00000000
C	-1.22798274	-20.62196850	0.00000000
C	-1.22144833	-17.81638595	0.00000000
C	-1.22144791	17.81638595	0.00000000
C	-1.22798231	20.62196845	0.00000000
C	-0.00001169	-19.94982987	0.00000000
C	-0.00000935	-18.49418317	0.00000000
C	-0.00000897	18.49418317	0.00000000
C	-0.00001132	19.94982984	0.00000000
C	1.22795687	-20.62197248	0.00000000
C	1.22143176	-17.81639025	0.00000000
C	1.22143208	17.81639025	0.00000000
C	1.22795719	20.62197242	0.00000000
C	2.45464877	-19.94370708	0.00000000
C	2.44789319	-18.48889962	0.00000000
C	2.44789346	18.48889961	0.00000000
C	2.45464905	19.94370704	0.00000000
C	3.68452246	-20.61029299	0.00000000
C	3.66488525	-17.80523865	0.00000000
C	3.66488548	17.80523865	0.00000000
C	3.68452269	20.61029295	0.00000000
C	4.90767364	-19.92538808	0.00000000
C	4.89418567	-18.47285875	0.00000000
C	4.89418585	18.47285874	0.00000000
C	4.90767382	19.92538805	0.00000000
C	6.14321573	-20.58649799	0.00000000
C	6.11002720	-17.78236593	0.00000000
C	6.11002734	17.78236592	0.00000000
C	6.14321587	20.58649794	0.00000000
C	7.35547970	-19.89154325	0.00000000
C	7.33658073	-18.44626946	0.00000000
C	7.33658083	18.44626944	0.00000000
C	7.35547979	19.89154322	0.00000000
C	8.60818673	-20.53250403	0.00000000
C	8.55628934	-17.74622164	0.00000000
C	8.55628941	17.74622162	0.00000000
C	8.60818680	20.53250397	0.00000000
C	9.80206809	-19.82667756	0.00000000

C	9.78365904	-18.38362690	0.00000000
C	9.78365906	18.38362689	0.00000000
C	9.80206814	19.82667753	0.00000000
C	11.05786690	-20.50560055	0.00000000
C	11.04043593	-17.65943476	0.00000000
C	11.10451440	-16.27779527	0.00000000
C	11.10451442	16.27779532	0.00000000
C	11.04043593	17.65943477	0.00000000
C	11.05786693	20.50560055	0.00000000
C	12.23668334	-19.82637031	0.00000000
C	12.27935002	-18.39937322	0.00000000
C	12.32206825	-15.57396494	0.00000000
C	12.36308342	-14.17984425	0.00000000
C	12.36308339	14.17984434	0.00000000
C	12.32206822	15.57396499	0.00000000
C	12.27935000	18.39937324	0.00000000
C	12.23668334	19.82637031	0.00000000
C	13.48887892	-17.72068543	0.00000000
C	13.56267161	-16.31542436	0.00000000
C	13.57033020	-13.47450206	0.00000000
C	13.60925220	-12.07616944	0.00000000
C	13.60925212	12.07616956	0.00000000
C	13.57033014	13.47450214	0.00000000
C	13.56267156	16.31542439	0.00000000
C	13.48887888	17.72068544	0.00000000
C	14.77202148	-15.61529086	0.00000000
C	14.81997917	-14.21473598	0.00000000
C	14.81103338	-11.36625820	0.00000000
C	14.84389316	-9.96794767	0.00000000
C	14.84389307	9.96794788	0.00000000
C	14.81103328	11.36625837	0.00000000
C	14.81997909	14.21473606	0.00000000
C	14.77202141	15.61529091	0.00000000
C	16.02602225	-13.49994351	0.00000000
C	16.06621096	-12.10148175	0.00000000
C	16.04258643	-9.25091462	0.00000000
C	16.06780393	-7.85430795	0.00000000
C	16.06780383	7.85430823	0.00000000
C	16.04258631	9.25091487	0.00000000
C	16.06621083	12.10148192	0.00000000
C	16.02602216	13.49994365	0.00000000
C	17.26808379	-11.37990251	0.00000000
C	17.30213750	-9.98024248	0.00000000
C	17.26412895	-7.12981946	0.00000000
C	17.28140906	-5.73412575	0.00000000
C	17.28140897	5.73412610	0.00000000
C	17.26412885	7.12981980	0.00000000
C	17.30213735	9.98024274	0.00000000
C	17.26808365	11.37990273	0.00000000
C	18.49906520	-9.25398867	0.00000000
C	18.52665877	-7.85231108	0.00000000
C	18.47457573	-5.00419099	0.00000000
C	18.48472686	-3.60607277	0.00000000
C	18.48472682	3.60607321	0.00000000
C	18.47457564	5.00419140	0.00000000
C	18.52665863	7.85231143	0.00000000
C	18.49906504	9.25398897	0.00000000
C	19.71939470	-7.12106877	0.00000000
C	19.73886285	-5.71909505	0.00000000
C	19.67295373	-2.87609250	0.00000000
C	19.67595987	-1.46983583	0.00000000
C	19.67595985	1.46983632	0.00000000
C	19.67295369	2.87609297	0.00000000
C	19.73886273	5.71909549	0.00000000
C	19.71939457	7.12106918	0.00000000
C	20.92934787	-4.97963890	0.00000000
C	20.93403015	-3.58205797	0.00000000
C	20.84128518	-0.72524747	0.00000000
C	20.84128517	0.72524801	0.00000000
C	20.93403007	3.58205847	0.00000000
C	20.92934774	4.97963938	0.00000000
C	22.11521920	-2.81699467	0.00000000
C	22.10039486	-1.42996302	0.00000000
C	22.10039484	1.42996358	0.00000000
C	22.11521914	2.81699521	0.00000000
C	23.31541291	-0.68034284	0.00000000
C	23.31541290	0.68034342	0.00000000
H	-24.26191071	-1.23842340	0.00000000
H	-24.26191070	1.23842301	0.00000000
H	-23.08418650	-3.33810670	0.00000000
H	-23.08418642	3.33810630	0.00000000
H	-21.88926893	-5.51697498	0.00000000
H	-21.88926881	5.51697460	0.00000000

H	-20.67568622	-7.66487987	0.00000000
H	-20.67568602	7.66487951	0.00000000
H	-19.45245617	-9.80289882	0.00000000
H	-19.45245591	9.80289855	0.00000000
H	-18.69798706	-0.96879670	0.00000000
H	-18.69798704	0.96879617	0.00000000
H	-18.21857928	-11.93382053	0.00000000
H	-18.21857894	11.93382033	0.00000000
H	-17.52570643	-3.06678520	0.00000000
H	-17.52570628	3.06678468	0.00000000
H	-16.97363720	-14.05876287	0.00000000
H	-16.97363679	14.05876276	0.00000000
H	-16.32541831	-5.18973946	0.00000000
H	-16.32541805	5.18973899	0.00000000
H	-15.71601567	-16.18018305	0.00000000
H	-15.71601520	16.18018301	0.00000000
H	-15.11453448	-7.30517961	0.00000000
H	-15.11453414	7.30517924	0.00000000
H	-14.42386811	-18.30065560	0.00000000
H	-14.42386760	18.30065561	0.00000000
H	-13.89328888	-9.41422656	0.00000000
H	-13.89328846	9.41422627	0.00000000
H	-13.19292558	-20.36764372	0.00000000
H	-13.19292504	20.36764374	0.00000000
H	-12.66131863	-11.51789440	0.00000000
H	-12.66131813	11.51789422	0.00000000
H	-11.41791551	-13.61669601	0.00000000
H	-11.41791497	13.61669592	0.00000000
H	-11.04654977	-21.60430054	0.00000000
H	-11.04654924	21.60430058	0.00000000
H	-10.18250188	-15.67999852	0.00000000
H	-10.18250134	15.67999850	0.00000000
H	-8.64098289	-21.63223252	0.00000000
H	-8.50175151	-16.64875424	0.00000000
H	-8.50175093	16.64875426	0.00000000
H	-8.64098232	21.63223254	0.00000000
H	-6.15828730	-21.68647265	0.00000000
H	-6.09711140	-16.68223196	0.00000000
H	-6.09711085	16.68223199	0.00000000
H	-6.15828673	21.68647265	0.00000000
H	-3.69264658	-21.71036344	0.00000000
H	-3.65747501	-16.70517152	0.00000000
H	-3.65747453	16.70517155	0.00000000
H	-3.69264604	21.71036343	0.00000000
H	-1.23067812	-21.72208729	0.00000000
H	-1.21900076	-16.71631091	0.00000000
H	-1.21900037	16.71631092	0.00000000
H	-1.23067768	21.72208723	0.00000000
H	1.23064818	-21.72209120	0.00000000
H	1.21898786	-16.71631521	0.00000000
H	1.21898818	16.71631523	0.00000000
H	1.23064850	21.72209112	0.00000000
H	3.69261542	-21.71037543	0.00000000
H	3.65746087	-16.70518433	0.00000000
H	3.65746110	16.70518435	0.00000000
H	3.69261566	21.71037536	0.00000000
H	6.15825469	-21.68649328	0.00000000
H	6.09709577	-16.68225288	0.00000000
H	6.09709590	16.68225289	0.00000000
H	6.15825484	21.68649322	0.00000000
H	8.64094986	-21.63226220	0.00000000
H	8.50173513	-16.64878245	0.00000000
H	8.50173521	16.64878244	0.00000000
H	8.64094993	21.63226214	0.00000000
H	10.18248772	-15.68002996	0.00000000
H	10.18248774	15.68003002	0.00000000
H	11.04651853	-21.60433836	0.00000000
H	11.04651854	21.60433835	0.00000000
H	11.41790661	-13.61672680	0.00000000
H	11.41790659	13.61672690	0.00000000
H	12.66131626	-11.51792227	0.00000000
H	12.66131619	11.51792240	0.00000000
H	13.19289900	-20.36768803	0.00000000
H	13.19289900	20.36768800	0.00000000
H	13.89329376	-9.41425003	0.00000000
H	13.89329369	9.41425023	0.00000000
H	14.42385077	-18.30070054	0.00000000
H	14.42385072	18.30070054	0.00000000
H	15.11454739	-7.30519783	0.00000000
H	15.11454732	7.30519808	0.00000000
H	15.71600658	-16.18022679	0.00000000
H	15.71600649	16.18022685	0.00000000
H	16.32543968	-5.18975214	0.00000000

H	16.32543962	5.18975248	0.00000000
H	16.97363656	-14.05880416	0.00000000
H	16.97363645	14.05880430	0.00000000
H	17.52573599	-3.06679220	0.00000000
H	17.52573598	3.06679261	0.00000000
H	18.21858770	-11.93385784	0.00000000
H	18.21858755	11.93385807	0.00000000
H	18.69802406	-0.96879891	0.00000000
H	18.69802405	0.96879938	0.00000000
H	19.45247390	-9.80293095	0.00000000
H	19.45247372	9.80293125	0.00000000
H	20.67571314	-7.66490570	0.00000000
H	20.67571298	7.66490613	0.00000000
H	21.88930444	-5.51699436	0.00000000
H	21.88930430	5.51699485	0.00000000
H	23.08423039	-3.33811908	0.00000000
H	23.08423031	3.33811965	0.00000000
H	24.26196000	-1.23842529	0.00000000
H	24.26195998	1.23842588	0.00000000

11-HGQR (singlet)

C	-25.77372936	-0.68024104	0.00000000
C	-25.77372941	0.68023886	0.00000000
C	-24.57379467	-2.81648649	0.00000000
C	-24.55848190	-1.42951197	0.00000000
C	-24.55848199	1.42950989	0.00000000
C	-24.57379485	2.81648446	0.00000000
C	-23.38949637	-4.97945723	0.00000000
C	-23.39304137	-3.58171067	0.00000000
C	-23.29921207	-0.72530073	0.00000000
C	-23.29921211	0.72529870	0.00000000
C	-23.39304155	3.58170876	0.00000000
C	-23.38949659	4.97945543	0.00000000
C	-22.18203115	-7.12187990	0.00000000
C	-22.19997025	-5.71934034	0.00000000
C	-22.13164661	-2.87673955	0.00000000
C	-22.13403540	-1.47045860	0.00000000
C	-22.13403546	1.47045667	0.00000000
C	-22.13164670	2.87673767	0.00000000
C	-22.19997043	5.71933870	0.00000000
C	-22.18203135	7.12187840	0.00000000
C	-20.96511747	-9.25635591	0.00000000
C	-20.99088395	-7.85373745	0.00000000
C	-20.93515678	-5.00603044	0.00000000
C	-20.94383360	-3.60820013	0.00000000
C	-20.94383367	3.60819839	0.00000000
C	-20.93515686	5.00602880	0.00000000
C	-20.99088407	7.85373613	0.00000000
C	-20.96511760	9.25635476	0.00000000
C	-19.73864898	-11.38420391	0.00000000
C	-19.77042690	-9.98366990	0.00000000
C	-19.72748963	-7.13337440	0.00000000
C	-19.74253115	-5.73840062	0.00000000
C	-19.74253117	5.73839915	0.00000000
C	-19.72748964	7.13337309	0.00000000
C	-19.77042692	9.98366895	0.00000000
C	-19.73864900	11.38420312	0.00000000
C	-18.50252513	-13.50631366	0.00000000
C	-18.53937475	-12.10811270	0.00000000
C	-18.50941736	-9.25684985	0.00000000
C	-18.53187001	-7.86127413	0.00000000
C	-18.53186993	7.86127300	0.00000000
C	-18.50941727	9.25684889	0.00000000
C	-18.53937467	12.10811208	0.00000000
C	-18.50252506	13.50631319	0.00000000
C	-17.25635624	-15.62341178	0.00000000
C	-17.29853195	-14.22606483	0.00000000
C	-17.28171033	-11.37550339	0.00000000
C	-17.31186567	-9.97788797	0.00000000
C	-17.31186550	9.97788719	0.00000000
C	-17.28171016	11.37550278	0.00000000
C	-17.29853181	14.22606450	0.00000000
C	-17.25635612	15.62341158	0.00000000
C	-15.99934987	-17.73643489	0.00000000
C	-16.04872378	-16.33654064	0.00000000
C	-16.04515558	-13.48836376	0.00000000
C	-16.08211260	-12.08896395	0.00000000
C	-16.08211236	12.08896350	0.00000000
C	-16.04515535	13.48836344	0.00000000
C	-16.04872360	16.33654055	0.00000000
C	-15.99934972	17.73643488	0.00000000
C	-14.71438642	-19.84005378	0.00000000
C	-14.78900908	-18.43513533	0.00000000
C	-14.80053058	-15.59448273	0.00000000

C	-14.84251939	-14.19540482	0.00000000
C	-14.84251912	14.19540465	0.00000000
C	-14.80053033	15.59448265	0.00000000
C	-14.78900890	18.43513541	0.00000000
C	-14.71438626	19.84005392	0.00000000
C	-13.46184007	-21.94486795	0.00000000
C	-13.50444680	-20.51786613	0.00000000
C	-13.54939660	-17.69241924	0.00000000
C	-13.59248623	-16.29791688	0.00000000
C	-13.59248596	16.29791691	0.00000000
C	-13.54939636	17.69241935	0.00000000
C	-13.50444662	20.51786633	0.00000000
C	-13.46183993	21.94486817	0.00000000
C	-12.28302252	-22.62365427	0.00000000
C	-12.26612451	-19.77711756	0.00000000
C	-12.33125343	-18.39532634	0.00000000
C	-12.33125320	18.39532654	0.00000000
C	-12.26612428	19.77711781	0.00000000
C	-12.28302237	22.62365456	0.00000000
C	-11.02763129	-21.94396772	0.00000000
C	-11.00903902	-20.50101252	0.00000000
C	-11.00903878	20.50101284	0.00000000
C	-11.02763110	21.94396804	0.00000000
C	-9.83390396	-22.64991955	0.00000000
C	-9.78136558	-19.86375270	0.00000000
C	-9.78136527	19.86375307	0.00000000
C	-9.83390377	22.64991991	0.00000000
C	-8.58143304	-22.00914610	0.00000000
C	-8.56176697	-20.56409021	0.00000000
C	-8.56176667	20.56409065	0.00000000
C	-8.58143279	22.00914653	0.00000000
C	-7.36951959	-22.70499594	0.00000000
C	-7.33420484	-19.90110516	0.00000000
C	-7.33420449	19.90110565	0.00000000
C	-7.36951934	22.70499640	0.00000000
C	-6.13420204	-22.04475302	0.00000000
C	-6.11922429	-20.59256268	0.00000000
C	-6.11922394	20.59256321	0.00000000
C	-6.13420174	22.04475354	0.00000000
C	-4.91131152	-22.73118604	0.00000000
C	-4.88809508	-19.92654473	0.00000000
C	-4.88809469	19.92654530	0.00000000
C	-4.91131120	22.73118658	0.00000000
C	-3.68176537	-22.06620171	0.00000000
C	-3.67277206	-20.61170858	0.00000000
C	-3.67277165	20.61170917	0.00000000
C	-3.68176498	22.06620230	0.00000000
C	-2.45522825	-22.74655021	0.00000000
C	-2.44365689	-19.94154030	0.00000000
C	-2.44365643	19.94154090	0.00000000
C	-2.45522781	22.74655079	0.00000000
C	-1.22747255	-22.07690048	0.00000000
C	-1.22447521	-20.62124284	0.00000000
C	-1.22447470	20.62124344	0.00000000
C	-1.22747204	22.07690109	0.00000000
C	-0.00001842	-22.75160543	0.00000000
C	-0.00001373	-19.94658880	0.00000000
C	-0.00001315	19.94658938	0.00000000
C	-0.00001781	22.75160603	0.00000000
C	1.22743811	-22.07690459	0.00000000
C	1.22444565	-20.62124689	0.00000000
C	1.22444633	20.62124745	0.00000000
C	1.22743881	22.07690518	0.00000000
C	2.45519200	-22.74655848	0.00000000
C	2.44362982	-19.94154845	0.00000000
C	2.44363059	19.94154897	0.00000000
C	2.45519285	22.74655905	0.00000000
C	3.68173200	-22.06621393	0.00000000
C	3.67274334	-20.61172060	0.00000000
C	3.67274422	20.61172108	0.00000000
C	3.68173294	22.06621447	0.00000000
C	4.91127697	-22.73120229	0.00000000
C	4.88806927	-19.92656069	0.00000000
C	4.88807025	19.92656113	0.00000000
C	4.91127808	22.73120279	0.00000000
C	6.13417069	-22.04477304	0.00000000
C	6.11919730	-20.59258235	0.00000000
C	6.11919842	20.59258272	0.00000000
C	6.13417188	22.04477347	0.00000000
C	7.36948749	-22.70501977	0.00000000
C	7.33418105	-19.90112851	0.00000000
C	7.33418222	19.90112882	0.00000000
C	7.36948881	22.70502013	0.00000000

C	8.58140428	-22.00917347	0.00000000
C	8.56174243	-20.56411709	0.00000000
C	8.56174372	20.56411733	0.00000000
C	8.58140565	22.00917374	0.00000000
C	9.83387462	-22.64995068	0.00000000
C	9.78134412	-19.86378294	0.00000000
C	9.78134543	19.86378312	0.00000000
C	9.83387610	22.64995088	0.00000000
C	11.02760548	-21.94400177	0.00000000
C	11.00901762	-20.50104568	0.00000000
C	11.00901905	20.50104577	0.00000000
C	11.02760697	21.94400187	0.00000000
C	12.28299599	-22.62369183	0.00000000
C	12.26610621	-19.77715343	0.00000000
C	12.33123992	-18.39536047	0.00000000
C	12.33124130	18.39536048	0.00000000
C	12.26610762	19.77715344	0.00000000
C	12.28299755	22.62369185	0.00000000
C	13.46181685	-21.94490776	0.00000000
C	13.50442782	-20.51790499	0.00000000
C	13.54938591	-17.69245481	0.00000000
C	13.59247970	-16.29795022	0.00000000
C	13.59248097	16.29795022	0.00000000
C	13.54938724	17.69245477	0.00000000
C	13.50442928	20.51790492	0.00000000
C	13.46181839	21.94490770	0.00000000
C	14.71437060	-19.84009430	0.00000000
C	14.78899759	-18.43517431	0.00000000
C	14.80052729	-15.59451676	0.00000000
C	14.84252033	-14.19543628	0.00000000
C	14.84252147	14.19543635	0.00000000
C	14.80052850	15.59451676	0.00000000
C	14.78899893	18.43517421	0.00000000
C	14.71437201	19.84009419	0.00000000
C	15.99934141	-17.73647472	0.00000000
C	16.04871990	-16.33657823	0.00000000
C	16.04515992	-13.48839509	0.00000000
C	16.08212118	-12.08899235	0.00000000
C	16.08212216	12.08899256	0.00000000
C	16.04516098	13.48839519	0.00000000
C	16.04872110	16.33657816	0.00000000
C	15.99934267	17.73647460	0.00000000
C	17.25635565	-15.62344957	0.00000000
C	17.29853601	-14.22609959	0.00000000
C	17.28172225	-11.37553084	0.00000000
C	17.31188162	-9.97791213	0.00000000
C	17.31188241	9.97791254	0.00000000
C	17.28172312	11.37553111	0.00000000
C	17.29853703	14.22609962	0.00000000
C	17.25635676	15.62344951	0.00000000
C	18.50253272	-13.50634775	0.00000000
C	18.53938675	-12.10814314	0.00000000
C	18.50943643	-9.25687239	0.00000000
C	18.53189271	-7.86129308	0.00000000
C	18.53189329	7.86129374	0.00000000
C	18.50943711	9.25687289	0.00000000
C	18.53938757	12.10814333	0.00000000
C	18.50253362	13.50634782	0.00000000
C	19.73866458	-11.38423277	0.00000000
C	19.77044648	-9.98369485	0.00000000
C	19.72751526	-7.13339133	0.00000000
C	19.74255981	-5.73841384	0.00000000
C	19.74256019	5.73841472	0.00000000
C	19.72751572	7.13339207	0.00000000
C	19.77044708	9.98369527	0.00000000
C	19.73866526	11.38423303	0.00000000
C	20.96514049	-9.25637854	0.00000000
C	20.99091042	-7.85375626	0.00000000
C	20.93518826	-5.00604150	0.00000000
C	20.94386747	-3.60820776	0.00000000
C	20.94386767	3.60820879	0.00000000
C	20.93518852	5.00604243	0.00000000
C	20.99091081	7.85375692	0.00000000
C	20.96514094	9.25637905	0.00000000
C	22.18206062	-7.12189607	0.00000000
C	22.20000269	-5.71935303	0.00000000
C	22.13168311	-2.87674504	0.00000000
C	22.13407368	-1.47046140	0.00000000
C	22.13407373	1.47046251	0.00000000
C	22.13168322	2.87674611	0.00000000
C	22.20000290	5.71935390	0.00000000
C	22.18206089	7.12189681	0.00000000
C	23.38953126	-4.97946744	0.00000000

C	23.39307877	-3.58171774	0.00000000
C	23.29925217	-0.72530036	0.00000000
C	23.29925218	0.72530148	0.00000000
C	23.39307885	3.58171875	0.00000000
C	23.38953137	4.97946836	0.00000000
C	24.57383419	-2.81649163	0.00000000
C	24.55852276	-1.42951378	0.00000000
C	24.55852277	1.42951487	0.00000000
C	24.57383422	2.81649267	0.00000000
C	25.77377155	-0.68024108	0.00000000
C	25.77377155	0.68024216	0.00000000
H	-26.72004199	-1.23866056	0.00000000
H	-26.72004208	1.23865834	0.00000000
H	-25.54296434	-3.33730556	0.00000000
H	-25.54296456	3.33730351	0.00000000
H	-24.34989764	-5.51600877	0.00000000
H	-24.34989791	5.51600697	0.00000000
H	-23.13907225	-7.66445375	0.00000000
H	-23.13907252	7.66445224	0.00000000
H	-21.91952942	-9.80358085	0.00000000
H	-21.91952962	9.80357970	0.00000000
H	-21.15595444	-0.96967639	0.00000000
H	-21.15595444	0.96967455	0.00000000
H	-20.69045206	-11.93596823	0.00000000
H	-20.69045216	11.93596743	0.00000000
H	-19.98434183	-3.06980953	0.00000000
H	-19.98434183	3.06980784	0.00000000
H	-19.45172917	-14.06252096	0.00000000
H	-19.45172917	14.06252045	0.00000000
H	-18.78582295	-5.19531278	0.00000000
H	-18.78582289	5.19531134	0.00000000
H	-18.20296515	-16.18398284	0.00000000
H	-18.20296508	16.18398259	0.00000000
H	-17.57764388	-7.31381954	0.00000000
H	-17.57764374	7.31381841	0.00000000
H	-16.94262714	-18.30254392	0.00000000
H	-16.94262703	18.30254387	0.00000000
H	-16.36009236	-9.42616706	0.00000000
H	-16.36009213	9.42616628	0.00000000
H	-15.64898223	-20.42066545	0.00000000
H	-15.64898212	20.42066555	0.00000000
H	-15.13277526	-11.53307365	0.00000000
H	-15.13277497	11.53307319	0.00000000
H	-14.41809254	-22.48605713	0.00000000
H	-14.41809244	22.48605731	0.00000000
H	-13.89559989	-13.63542065	0.00000000
H	-13.89559958	13.63542049	0.00000000
H	-12.64806274	-15.73353364	0.00000000
H	-12.64806244	15.73353369	0.00000000
H	-12.27092264	-23.72235267	0.00000000
H	-12.27092252	23.72235296	0.00000000
H	-11.40957102	-17.79700150	0.00000000
H	-11.40957079	17.79700169	0.00000000
H	-9.86676444	-23.74967082	0.00000000
H	-9.72675406	-18.76629978	0.00000000
H	-9.72675369	18.76630016	0.00000000
H	-9.86676429	23.74967117	0.00000000
H	-7.38532328	-23.80497710	0.00000000
H	-7.32040697	-18.80099981	0.00000000
H	-7.32040658	18.80100031	0.00000000
H	-7.38532308	23.80497756	0.00000000
H	-4.92079293	-23.83126301	0.00000000
H	-4.87925512	-18.82649204	0.00000000
H	-4.87925470	18.82649262	0.00000000
H	-4.92079263	23.83126355	0.00000000
H	-2.45994155	-23.84667706	0.00000000
H	-2.43926365	-18.84145388	0.00000000
H	-2.43926316	18.84145447	0.00000000
H	-2.45994110	23.84667765	0.00000000
H	-0.00002023	-23.85174977	0.00000000
H	-0.00001197	-18.84648768	0.00000000
H	-0.00001137	18.84648824	0.00000000
H	-0.00001958	23.85175039	0.00000000
H	2.45990176	-23.84668547	0.00000000
H	2.43924015	-18.84146198	0.00000000
H	2.43924091	18.84146246	0.00000000
H	2.45990266	23.84668607	0.00000000
H	4.92075500	-23.83127951	0.00000000
H	4.87923290	-18.82650784	0.00000000
H	4.87923384	18.82650824	0.00000000
H	4.92075616	23.83128005	0.00000000
H	7.38528795	-23.80500127	0.00000000
H	7.32038655	-18.80102286	0.00000000

H	7.32038765	18.80102313	0.00000000
H	7.38528933	23.80500167	0.00000000
H	9.86673200	-23.74970240	0.00000000
H	9.72673512	-18.76632955	0.00000000
H	9.72673631	18.76632972	0.00000000
H	9.86673353	23.74970262	0.00000000
H	11.40955922	-17.79703208	0.00000000
H	11.40956056	17.79703212	0.00000000
H	12.27089309	-23.72239060	0.00000000
H	12.27089471	23.72239064	0.00000000
H	12.64805697	-15.73356415	0.00000000
H	12.64805822	15.73356420	0.00000000
H	13.89560122	-13.63544959	0.00000000
H	13.89560235	13.63544972	0.00000000
H	14.41806840	-22.48609957	0.00000000
H	14.41806996	22.48609947	0.00000000
H	15.13278393	-11.53309986	0.00000000
H	15.13278491	11.53310014	0.00000000
H	15.64896560	-20.42070837	0.00000000
H	15.64896703	20.42070821	0.00000000
H	16.36010822	-9.42618927	0.00000000
H	16.36010903	9.42618975	0.00000000
H	16.94261803	-18.30258620	0.00000000
H	16.94261930	18.30258605	0.00000000
H	17.57766634	-7.31383666	0.00000000
H	17.57766696	7.31383737	0.00000000
H	18.20296413	-16.18402308	0.00000000
H	18.20296522	16.18402299	0.00000000
H	18.78585117	-5.19532440	0.00000000
H	18.78585158	5.19532531	0.00000000
H	19.45173657	-14.06255740	0.00000000
H	19.45173744	14.06255743	0.00000000
H	19.98437498	-3.06981608	0.00000000
H	19.98437521	3.06981713	0.00000000
H	20.69046776	-11.93599913	0.00000000
H	20.69046840	11.93599935	0.00000000
H	21.15599140	-0.96967973	0.00000000
H	21.15599147	0.96968082	0.00000000
H	21.91955280	-9.80360511	0.00000000
H	21.91955321	9.80360556	0.00000000
H	23.13910219	-7.66447123	0.00000000
H	23.13910243	7.66447190	0.00000000
H	24.34993299	-5.51602016	0.00000000
H	24.34993309	5.51602101	0.00000000
H	25.54300435	-3.33731174	0.00000000
H	25.54300437	3.33731272	0.00000000
H	26.72008486	-1.23866118	0.00000000
H	26.72008487	1.23866223	0.00000000

12-HGQR (singlet)

C	-28.23372305	-0.68015806	0.00000000
C	-28.23372347	0.68014645	0.00000000
C	-27.03410550	-2.81603812	0.00000000
C	-27.01830760	-1.42925313	0.00000000
C	-27.01830841	1.42924215	0.00000000
C	-27.03410710	2.81602728	0.00000000
C	-25.85114554	-4.97922560	0.00000000
C	-25.85362906	-3.58159961	0.00000000
C	-25.75877588	-0.72538569	0.00000000
C	-25.75877628	0.72537526	0.00000000
C	-25.85363087	3.58158936	0.00000000
C	-25.85114807	4.97921564	0.00000000
C	-24.64571248	-7.12239134	0.00000000
C	-24.66221483	-5.71971034	0.00000000
C	-24.59185800	-2.87736527	0.00000000
C	-24.59383863	-1.47090297	0.00000000
C	-24.59383932	1.47089310	0.00000000
C	-24.59185932	2.87735547	0.00000000
C	-24.66221738	5.71970100	0.00000000
C	-24.64571563	7.12238247	0.00000000
C	-23.43149120	-9.25811983	0.00000000
C	-23.45559542	-7.85494267	0.00000000
C	-23.39689266	-5.00762265	0.00000000
C	-23.40451008	-3.60975101	0.00000000
C	-23.40451149	3.60974176	0.00000000
C	-23.39689467	5.00761363	0.00000000
C	-23.45559837	7.85493447	0.00000000
C	-23.43149454	9.25811224	0.00000000
C	-22.20842478	-11.38764819	0.00000000
C	-22.23839531	-9.98616403	0.00000000
C	-22.19153149	-7.13628356	0.00000000
C	-22.20484501	-5.74158620	0.00000000
C	-22.20484694	5.74157774	0.00000000
C	-22.19153389	7.13627553	0.00000000

C	-22.23839828	9.98615719	0.00000000
C	-22.20842786	11.38764208	0.00000000
C	-20.97660491	-13.51169684	0.00000000
C	-21.01134187	-12.11259817	0.00000000
C	-20.97645125	-9.26146967	0.00000000
C	-20.99652652	-7.86661704	0.00000000
C	-20.99652870	7.86660963	0.00000000
C	-20.97645371	9.26146287	0.00000000
C	-21.01134448	12.11259286	0.00000000
C	-20.97660735	13.51169231	0.00000000
C	-19.73597070	-15.63075367	0.00000000
C	-19.77510601	-14.23362338	0.00000000
C	-19.75222016	-11.38237623	0.00000000
C	-19.77959571	-9.98581720	0.00000000
C	-19.77959785	9.98581112	0.00000000
C	-19.75222234	11.38237089	0.00000000
C	-19.77510798	14.23361966	0.00000000
C	-19.73597229	15.63075071	0.00000000
C	-18.48617983	-17.74529504	0.00000000
C	-18.53022467	-16.34854601	0.00000000
C	-18.51930951	-13.49829735	0.00000000
C	-18.55367345	-12.09964108	0.00000000
C	-18.55367528	12.09963654	0.00000000
C	-18.51931116	13.49829358	0.00000000
C	-18.53022585	16.34854382	0.00000000
C	-18.48618052	17.74529355	0.00000000
C	-17.22648122	-19.85617487	0.00000000
C	-17.27732781	-18.45654725	0.00000000
C	-17.27842928	-15.60866790	0.00000000
C	-17.31868825	-14.20859410	0.00000000
C	-17.31868956	14.20859117	0.00000000
C	-17.27843025	15.60866566	0.00000000
C	-17.27732817	18.45654644	0.00000000
C	-17.22648109	19.85617464	0.00000000
C	-15.93979215	-21.95807997	0.00000000
C	-16.01539497	-20.55328760	0.00000000
C	-16.03032521	-17.71278928	0.00000000
C	-16.07476328	-16.31342314	0.00000000
C	-16.07476393	16.31342169	0.00000000
C	-16.03032545	17.71278842	0.00000000
C	-16.01539456	20.55328792	0.00000000
C	-15.93979135	21.95808066	0.00000000
C	-14.68672987	-24.06191893	0.00000000
C	-14.72952352	-22.63488379	0.00000000
C	-14.77656539	-19.80932160	0.00000000
C	-14.82136212	-18.41476328	0.00000000
C	-14.82136209	18.41476308	0.00000000
C	-14.77656494	19.80932190	0.00000000
C	-14.72952242	22.63488490	0.00000000
C	-14.68672872	24.06192027	0.00000000
C	-13.50785642	-24.74021697	0.00000000
C	-13.49164428	-21.89324993	0.00000000
C	-13.55776262	-20.51149619	0.00000000
C	-13.55776194	20.51149693	0.00000000
C	-13.49164293	21.89325120	0.00000000
C	-13.50785484	24.74021867	0.00000000
C	-12.25279073	-24.05984419	0.00000000
C	-12.23416454	-22.61681584	0.00000000
C	-12.23416281	22.61681744	0.00000000
C	-12.25278891	24.05984594	0.00000000
C	-11.05930595	-24.76580008	0.00000000
C	-11.00644455	-21.97962315	0.00000000
C	-11.00644207	21.97962516	0.00000000
C	-11.05930379	24.76580202	0.00000000
C	-9.80687201	-24.12511282	0.00000000
C	-9.78668819	-22.68009604	0.00000000
C	-9.78668545	22.68009821	0.00000000
C	-9.80686942	24.12511500	0.00000000
C	-8.59549956	-24.82160472	0.00000000
C	-8.55865129	-22.01775396	0.00000000
C	-8.55864805	22.01775631	0.00000000
C	-8.59549664	24.82160704	0.00000000
C	-7.36010500	-24.16195864	0.00000000
C	-7.34401823	-22.70993790	0.00000000
C	-7.34401463	22.70994035	0.00000000
C	-7.36010156	24.16196112	0.00000000
C	-6.13777813	-24.84958596	0.00000000
C	-6.11183581	-22.04509263	0.00000000
C	-6.11183176	22.04509517	0.00000000
C	-6.13777431	24.84958857	0.00000000
C	-4.90824295	-24.18571897	0.00000000
C	-4.89752570	-22.73149847	0.00000000
C	-4.89752126	22.73150111	0.00000000

C	-4.90823861	24.18572165	0.00000000
C	-3.68208898	-24.86777345	0.00000000
C	-3.66658776	-22.06306217	0.00000000
C	-3.66658289	22.06306483	0.00000000
C	-3.68208423	24.86777624	0.00000000
C	-2.45454066	-24.19985430	0.00000000
C	-2.44919014	-22.74445112	0.00000000
C	-2.44918488	22.74445386	0.00000000
C	-2.45453543	24.19985707	0.00000000
C	-1.22727859	-24.87670765	0.00000000
C	-1.22212056	-22.07215818	0.00000000
C	-1.22211491	22.07216089	0.00000000
C	-1.22727293	24.87671048	0.00000000
C	0.00000136	-24.20452865	0.00000000
C	0.00000122	-22.74880286	0.00000000
C	0.00000724	22.74880560	0.00000000
C	0.00000742	24.20453139	0.00000000
C	1.22728126	-24.87670725	0.00000000
C	1.22212270	-22.07215811	0.00000000
C	1.22212900	22.07216078	0.00000000
C	1.22728773	24.87670996	0.00000000
C	2.45454294	-24.19985364	0.00000000
C	2.44919214	-22.74445085	0.00000000
C	2.44919875	22.74445346	0.00000000
C	2.45454966	24.19985619	0.00000000
C	3.68209081	-24.86777232	0.00000000
C	3.66658910	-22.06306193	0.00000000
C	3.66659585	22.06306444	0.00000000
C	3.68209788	24.86777473	0.00000000
C	4.90824405	-24.18571775	0.00000000
C	4.89752655	-22.73149790	0.00000000
C	4.89753353	22.73150025	0.00000000
C	4.90825124	24.18571998	0.00000000
C	6.13777859	-24.84958429	0.00000000
C	6.11183582	-22.04509211	0.00000000
C	6.11184281	22.04509434	0.00000000
C	6.13778606	24.84958626	0.00000000
C	7.36010460	-24.16195699	0.00000000
C	7.34401760	-22.70993696	0.00000000
C	7.34402475	22.70993892	0.00000000
C	7.36011204	24.16195876	0.00000000
C	8.59549858	-24.82160265	0.00000000
C	8.55864986	-22.01775304	0.00000000
C	8.55865692	22.01775487	0.00000000
C	8.59550629	24.82160402	0.00000000
C	9.80687017	-24.12511075	0.00000000
C	9.78668616	-22.68009464	0.00000000
C	9.78669338	22.68009602	0.00000000
C	9.80687772	24.12511186	0.00000000
C	11.05930372	-24.76579753	0.00000000
C	11.00644181	-21.97962162	0.00000000
C	11.00644877	21.97962279	0.00000000
C	11.05931146	24.76579803	0.00000000
C	12.25278788	-24.05984179	0.00000000
C	12.23416123	-22.61681395	0.00000000
C	12.23416849	22.61681441	0.00000000
C	12.25279540	24.05984205	0.00000000
C	13.50785308	-24.74021454	0.00000000
C	13.49164061	-21.89324816	0.00000000
C	13.55775877	-20.51149502	0.00000000
C	13.55776638	20.51149535	0.00000000
C	13.49164785	21.89324833	0.00000000
C	13.50786050	24.74021454	0.00000000
C	14.68672613	-24.06191670	0.00000000
C	14.72951939	-22.63488200	0.00000000
C	14.77656125	-19.80932086	0.00000000
C	14.82135777	-18.41476309	0.00000000
C	14.82136540	18.41476384	0.00000000
C	14.77656865	19.80932126	0.00000000
C	14.72952642	22.63488192	0.00000000
C	14.68673345	24.06191643	0.00000000
C	15.93978765	-21.95807854	0.00000000
C	16.01539047	-20.55328660	0.00000000
C	16.03032052	-17.71278946	0.00000000
C	16.07475828	-16.31342375	0.00000000
C	16.07476564	16.31342495	0.00000000
C	16.03032777	17.71279020	0.00000000
C	16.01539742	20.55328668	0.00000000
C	15.93979436	21.95807834	0.00000000
C	17.22647644	-19.85617438	0.00000000
C	17.27732284	-18.45654719	0.00000000
C	17.27842398	-15.60866877	0.00000000
C	17.31868263	-14.20859513	0.00000000

C	17.31868950	14.20859700	0.00000000
C	17.27843084	15.60867009	0.00000000
C	17.27732957	18.45654760	0.00000000
C	17.22648298	19.85617447	0.00000000
C	18.48617461	-17.74529537	0.00000000
C	18.53021922	-16.34854669	0.00000000
C	18.51930376	-13.49829840	0.00000000
C	18.55366746	-12.09964185	0.00000000
C	18.55367351	12.09964457	0.00000000
C	18.51931001	13.49830050	0.00000000
C	18.53022553	16.34854761	0.00000000
C	18.48618083	17.74529581	0.00000000
C	19.73596524	-15.63075456	0.00000000
C	19.77510038	-14.23362437	0.00000000
C	19.75221431	-11.38237666	0.00000000
C	19.77958978	-9.98581689	0.00000000
C	19.77959478	9.98582061	0.00000000
C	19.75221973	11.38237974	0.00000000
C	19.77510610	14.23362607	0.00000000
C	19.73597097	15.63075563	0.00000000
C	20.97659958	-13.51169772	0.00000000
C	21.01133650	-12.11259875	0.00000000
C	20.97644576	-9.26146866	0.00000000
C	20.99652108	-7.86661497	0.00000000
C	20.99652489	7.86661972	0.00000000
C	20.97645018	9.26147279	0.00000000
C	21.01134146	12.11260141	0.00000000
C	20.97660473	13.51169970	0.00000000
C	22.20842003	-11.38764825	0.00000000
C	22.23839063	-9.98616334	0.00000000
C	22.19152672	-7.13628057	0.00000000
C	22.20484037	-5.74158207	0.00000000
C	22.20484297	5.74158773	0.00000000
C	22.19153002	7.13628569	0.00000000
C	22.23839471	9.98616705	0.00000000
C	22.20842448	11.38765129	0.00000000
C	23.43148732	-9.25811829	0.00000000
C	23.45559164	-7.85494008	0.00000000
C	23.39688877	-5.00761757	0.00000000
C	23.40450633	-3.60974498	0.00000000
C	23.40450782	3.60975125	0.00000000
C	23.39689094	5.00762347	0.00000000
C	23.45559476	7.85494477	0.00000000
C	23.43149095	9.25812242	0.00000000
C	24.64570950	-7.12238779	0.00000000
C	24.66221193	-5.71970566	0.00000000
C	24.59185500	-2.87735849	0.00000000
C	24.59383583	-1.47089549	0.00000000
C	24.59383637	1.47090199	0.00000000
C	24.59185612	2.87736480	0.00000000
C	24.66221408	5.71971113	0.00000000
C	24.64571222	7.12239282	0.00000000
C	25.85114330	-4.97922014	0.00000000
C	25.85362692	-3.58159315	0.00000000
C	25.75877369	-0.72537749	0.00000000
C	25.75877394	0.72538384	0.00000000
C	25.85362815	3.58159904	0.00000000
C	25.85114510	4.97922573	0.00000000
C	27.03410395	-2.81603121	0.00000000
C	27.01830587	-1.42924545	0.00000000
C	27.01830634	1.42925140	0.00000000
C	27.03410487	2.81603700	0.00000000
C	28.23372152	-0.68014961	0.00000000
C	28.23372173	0.68015530	0.00000000
H	-29.17986123	-1.23881430	0.00000000
H	-29.17986196	1.23880224	0.00000000
H	-28.00341129	-3.33657304	0.00000000
H	-28.00341317	3.33656184	0.00000000
H	-26.81189585	-5.51511948	0.00000000
H	-26.81189868	5.51510927	0.00000000
H	-25.60330897	-7.66396243	0.00000000
H	-25.60331243	7.66395339	0.00000000
H	-24.38666628	-9.80400363	0.00000000
H	-24.38666997	9.80399592	0.00000000
H	-23.61564644	-0.97032708	0.00000000
H	-23.61564685	0.97031774	0.00000000
H	-23.16121640	-11.93771512	0.00000000
H	-23.16121982	11.93770891	0.00000000
H	-22.44463104	-3.07207357	0.00000000
H	-22.44463209	3.07206475	0.00000000
H	-21.92703649	-14.06583076	0.00000000
H	-21.92703923	14.06582617	0.00000000
H	-21.24755710	-5.19953720	0.00000000

H	-21.24755866	5.19952905	0.00000000
H	-20.68404149	-16.18888233	0.00000000
H	-20.68404331	16.18887937	0.00000000
H	-20.04153026	-7.32051061	0.00000000
H	-20.04153212	7.32050336	0.00000000
H	-19.43188725	-18.30736458	0.00000000
H	-19.43188808	18.30736312	0.00000000
H	-18.82687128	-9.43572580	0.00000000
H	-18.82687316	9.43571973	0.00000000
H	-18.16909371	-20.42335985	0.00000000
H	-18.16909365	20.42335966	0.00000000
H	-17.60323106	-11.54561525	0.00000000
H	-17.60323269	11.54561064	0.00000000
H	-16.87398663	-22.53930951	0.00000000
H	-16.87398594	22.53931016	0.00000000
H	-16.37048607	-13.65077174	0.00000000
H	-16.37048724	13.65076872	0.00000000
H	-15.64293775	-24.60312492	0.00000000
H	-15.64293678	24.60312605	0.00000000
H	-15.12876319	-15.75190096	0.00000000
H	-15.12876375	15.75189943	0.00000000
H	-13.87763293	-17.84923932	0.00000000
H	-13.87763287	17.84923904	0.00000000
H	-13.49506618	-25.83887686	0.00000000
H	-13.49506450	25.83887859	0.00000000
H	-12.63643130	-19.91261704	0.00000000
H	-12.63643087	19.91261740	0.00000000
H	-11.09218699	-25.86553670	0.00000000
H	-10.95188716	-20.88215925	0.00000000
H	-10.95188422	20.88216131	0.00000000
H	-11.09218495	25.86553865	0.00000000
H	-8.61185328	-25.92156249	0.00000000
H	-8.54423409	-20.91766585	0.00000000
H	-8.54423076	20.91766821	0.00000000
H	-8.61185053	25.92156479	0.00000000
H	-6.14830047	-25.94964316	0.00000000
H	-6.10191127	-20.94505534	0.00000000
H	-6.10190719	20.94505792	0.00000000
H	-6.14829677	25.94964573	0.00000000
H	-3.68833891	-25.96788902	0.00000000
H	-3.66064731	-20.96298293	0.00000000
H	-3.66064244	20.96298566	0.00000000
H	-3.68833424	25.96789174	0.00000000
H	-1.22934863	-25.97685565	0.00000000
H	-1.22012901	-20.97204997	0.00000000
H	-1.22012341	20.97205279	0.00000000
H	-1.22934299	25.97685839	0.00000000
H	1.22935104	-25.97685510	0.00000000
H	1.22013047	-20.97205006	0.00000000
H	1.22013665	20.97205287	0.00000000
H	1.22935754	25.97685768	0.00000000
H	3.68834065	-25.96788749	0.00000000
H	3.66064814	-20.96298310	0.00000000
H	3.66065473	20.96298580	0.00000000
H	3.68834782	25.96788974	0.00000000
H	6.14830104	-25.94964097	0.00000000
H	6.10191102	-20.94505536	0.00000000
H	6.10191781	20.94505782	0.00000000
H	6.14830870	25.94964276	0.00000000
H	8.61185263	-25.92155990	0.00000000
H	8.54423252	-20.91766549	0.00000000
H	8.54423929	20.91766759	0.00000000
H	8.61186064	25.92156106	0.00000000
H	11.09218502	-25.86553372	0.00000000
H	10.95188437	-20.88215827	0.00000000
H	10.95189068	20.88215980	0.00000000
H	11.09219302	25.86553402	0.00000000
H	12.63642790	-19.91261612	0.00000000
H	12.63643591	19.91261649	0.00000000
H	13.49506276	-25.83887406	0.00000000
H	13.49507006	25.83887387	0.00000000
H	13.87762900	-17.84923931	0.00000000
H	13.87763691	17.84924037	0.00000000
H	15.12875857	-15.75190162	0.00000000
H	15.12876627	15.75190318	0.00000000
H	15.64293385	-24.60312230	0.00000000
H	15.64294117	24.60312153	0.00000000
H	16.37048076	-13.65077256	0.00000000
H	16.37048803	13.65077474	0.00000000
H	16.87398182	-22.53930794	0.00000000
H	16.87398826	22.53930758	0.00000000
H	17.60322517	-11.54561557	0.00000000
H	17.60323165	11.54561854	0.00000000

H	18.16908859	-20.42335920	0.00000000
H	18.16909470	20.42335923	0.00000000
H	18.82686512	-9.43572492	0.00000000
H	18.82687052	9.43572885	0.00000000
H	19.43188189	-18.30736456	0.00000000
H	19.43188767	18.30736483	0.00000000
H	20.04152428	-7.32050803	0.00000000
H	20.04152838	7.32051300	0.00000000
H	20.68403615	-16.18888278	0.00000000
H	20.68404146	16.18888357	0.00000000
H	21.24755170	-5.19953275	0.00000000
H	21.24755446	5.19953862	0.00000000
H	21.92703161	-14.06583121	0.00000000
H	21.92703640	14.06583279	0.00000000
H	22.44462651	-3.07206730	0.00000000
H	22.44462805	3.07207378	0.00000000
H	23.16121235	-11.93771493	0.00000000
H	23.16121652	11.93771754	0.00000000
H	23.61564288	-0.97031957	0.00000000
H	23.61564341	0.97032618	0.00000000
H	24.38666322	-9.80400214	0.00000000
H	24.38666666	9.80400581	0.00000000
H	25.60330675	-7.66395917	0.00000000
H	25.60330938	7.66396377	0.00000000
H	26.81189419	-5.51511453	0.00000000
H	26.81189601	5.51511968	0.00000000
H	28.00341024	-3.33656645	0.00000000
H	28.00341125	3.33657186	0.00000000
H	29.17986033	-1.23880575	0.00000000
H	29.17986072	1.23881107	0.00000000

13-HGQR (singlet)

C	-30.69565596	-0.68010721	0.00000000
C	-30.69565681	0.68006099	0.00000000
C	-29.49638371	-2.81569843	0.00000000
C	-29.48014375	-1.42911842	0.00000000
C	-29.48014560	1.42907391	0.00000000
C	-29.49638674	2.81565455	0.00000000
C	-28.31456376	-4.97902478	0.00000000
C	-28.31608795	-3.58161011	0.00000000
C	-28.22034806	-0.72551243	0.00000000
C	-28.22034892	0.72546944	0.00000000
C	-28.31609143	3.58156828	0.00000000
C	-28.31456786	4.97898406	0.00000000
C	-27.11085888	-7.12275432	0.00000000
C	-27.12603696	-5.72017210	0.00000000
C	-27.05396796	-2.87796420	0.00000000
C	-27.05564428	-1.47128409	0.00000000
C	-27.05564577	1.47124283	0.00000000
C	-27.05397061	2.87792361	0.00000000
C	-27.12604103	5.72013423	0.00000000
C	-27.11086294	7.12271819	0.00000000
C	-25.89887810	-9.25945325	0.00000000
C	-25.92140212	-7.85610019	0.00000000
C	-25.86023824	-5.00904872	0.00000000
C	-25.86705926	-3.61098692	0.00000000
C	-25.86706219	3.61094874	0.00000000
C	-25.86024162	5.00901200	0.00000000
C	-25.92140562	7.85606777	0.00000000
C	-25.89888098	9.25942336	0.00000000
C	-24.67855145	-11.39035825	0.00000000
C	-24.70682231	-9.98827948	0.00000000
C	-24.65676243	-7.13881011	0.00000000
C	-24.66876477	-5.74407276	0.00000000
C	-24.66876799	5.74403906	0.00000000
C	-24.65676530	7.13877878	0.00000000
C	-24.70682419	9.98825415	0.00000000
C	-24.67855216	11.39033623	0.00000000
C	-23.45003418	-13.51611783	0.00000000
C	-23.48302593	-12.11603876	0.00000000
C	-23.44419573	-9.26535418	0.00000000
C	-23.46237642	-7.87078118	0.00000000
C	-23.46237867	7.87075369	0.00000000
C	-23.44419695	9.26532988	0.00000000
C	-23.48302533	12.11602184	0.00000000
C	-23.45003208	13.51610466	0.00000000
C	-22.21345818	-15.63705679	0.00000000
C	-22.25063854	-14.23900124	0.00000000
C	-22.22301301	-11.38792200	0.00000000
C	-22.24793929	-9.99209213	0.00000000
C	-22.24793945	9.99207248	0.00000000
C	-22.22301172	11.38790605	0.00000000
C	-22.25063507	14.23899320	0.00000000
C	-22.21345317	15.63705242	0.00000000

C	-20.96880873	-17.75341907	0.00000000
C	-21.01006515	-16.35685616	0.00000000
C	-20.99347946	-13.50595389	0.00000000
C	-21.02509298	-12.10836285	0.00000000
C	-21.02509038	12.10835202	0.00000000
C	-20.99347535	13.50594680	0.00000000
C	-21.01005901	16.35685640	0.00000000
C	-20.96880130	17.75342248	0.00000000
C	-19.71577241	-19.86559338	0.00000000
C	-19.76165218	-18.46907475	0.00000000
C	-19.75598981	-15.61915559	0.00000000
C	-19.79378868	-14.21983698	0.00000000
C	-19.79378330	14.21983493	0.00000000
C	-19.75598315	15.61915680	0.00000000
C	-19.76164395	18.46908189	0.00000000
C	-19.71576324	19.86560303	0.00000000
C	-18.45360285	-21.97444608	0.00000000
C	-18.50593564	-20.57489149	0.00000000
C	-18.51122954	-17.72721559	0.00000000
C	-18.55417150	-16.32687171	0.00000000
C	-18.55416382	16.32687729	0.00000000
C	-18.51122092	17.72722365	0.00000000
C	-18.50592587	20.57490394	0.00000000
C	-18.45359236	21.97446041	0.00000000
C	-17.16527870	-24.07471704	0.00000000
C	-17.24187345	-22.66996447	0.00000000
C	-17.25994945	-19.82953915	0.00000000
C	-17.30643831	-18.43014976	0.00000000
C	-17.30642905	18.43016123	0.00000000
C	-17.25993946	19.82955228	0.00000000
C	-17.24186239	22.66998072	0.00000000
C	-17.16526699	24.07473450	0.00000000
C	-15.91155309	-26.17755127	0.00000000
C	-15.95469246	-24.75049972	0.00000000
C	-16.00374286	-21.92479705	0.00000000
C	-16.05004314	-20.53030580	0.00000000
C	-16.05003277	20.53032126	0.00000000
C	-16.00373172	21.92481360	0.00000000
C	-15.95468014	24.75051867	0.00000000
C	-15.91154051	26.17757040	0.00000000
C	-14.73256662	-26.85534033	0.00000000
C	-14.71721525	-24.00797202	0.00000000
C	-14.78427768	-22.62630461	0.00000000
C	-14.78426624	22.62632249	0.00000000
C	-14.71720311	24.00799063	0.00000000
C	-14.73255285	26.85535955	0.00000000
C	-13.47780877	-26.17434021	0.00000000
C	-13.45926317	-24.73119621	0.00000000
C	-13.45925051	24.73121473	0.00000000
C	-13.47779555	26.17435848	0.00000000
C	-12.28452961	-26.88021008	0.00000000
C	-12.23160709	-24.09396545	0.00000000
C	-12.23159454	24.09398372	0.00000000
C	-12.28451566	26.88022730	0.00000000
C	-11.03209668	-26.23948368	0.00000000
C	-11.01158193	-24.79445356	0.00000000
C	-11.01156897	24.79447100	0.00000000
C	-11.03208306	26.23950048	0.00000000
C	-9.82126694	-26.93644471	0.00000000
C	-9.78332572	-24.13254933	0.00000000
C	-9.78331335	24.13256669	0.00000000
C	-9.82125334	26.93646078	0.00000000
C	-8.58566525	-26.27721826	0.00000000
C	-8.56875459	-24.82526524	0.00000000
C	-8.56874223	24.82528163	0.00000000
C	-8.58565239	26.27723394	0.00000000
C	-7.36402823	-26.96578445	0.00000000
C	-7.33599653	-24.16128576	0.00000000
C	-7.33598495	24.16130225	0.00000000
C	-7.36401575	26.96579962	0.00000000
C	-6.13424849	-26.30272531	0.00000000
C	-6.12219206	-24.84869148	0.00000000
C	-6.12218090	24.84870716	0.00000000
C	-6.13423701	26.30274027	0.00000000
C	-4.90873887	-26.98618197	0.00000000
C	-4.89014171	-24.18155339	0.00000000
C	-4.89013135	24.18156938	0.00000000
C	-4.90872807	26.98619664	0.00000000
C	-3.68109258	-26.31949832	0.00000000
C	-3.67387632	-24.86439038	0.00000000
C	-3.67386678	24.86440572	0.00000000
C	-3.68108289	26.31951294	0.00000000
C	-2.45424208	-26.99818176	0.00000000

C	-2.44496719	-24.19386998	0.00000000
C	-2.44495862	24.19388576	0.00000000
C	-2.45423334	26.99819625	0.00000000
C	-1.22710849	-26.32777438	0.00000000
C	-1.22470618	-24.87233075	0.00000000
C	-1.22469871	24.87234601	0.00000000
C	-1.22710096	26.32778884	0.00000000
C	-0.00000553	-27.00214369	0.00000000
C	-0.00000590	-24.19800284	0.00000000
C	0.00000040	24.19801860	0.00000000
C	0.00000082	27.00215807	0.00000000
C	1.22709739	-26.32777406	0.00000000
C	1.22469470	-24.87233043	0.00000000
C	1.22469980	24.87234568	0.00000000
C	1.22710250	26.32778846	0.00000000
C	2.45423137	-26.99818113	0.00000000
C	2.44495578	-24.19386935	0.00000000
C	2.44495962	24.19388515	0.00000000
C	2.45423516	26.99819545	0.00000000
C	3.68108213	-26.31949741	0.00000000
C	3.67386553	-24.86438942	0.00000000
C	3.67386823	24.86440472	0.00000000
C	3.68108476	26.31951183	0.00000000
C	4.90872911	-26.98618075	0.00000000
C	4.89013132	-24.18155218	0.00000000
C	4.89013290	24.18156814	0.00000000
C	4.90873036	26.98619509	0.00000000
C	6.13423918	-26.30272385	0.00000000
C	6.12218247	-24.84868998	0.00000000
C	6.12218294	24.84870553	0.00000000
C	6.13423943	26.30273848	0.00000000
C	7.36401973	-26.96578275	0.00000000
C	7.33598754	-24.16128402	0.00000000
C	7.33598719	24.16130043	0.00000000
C	7.36401865	26.96579743	0.00000000
C	8.58565728	-26.27721638	0.00000000
C	8.56874640	-24.82526327	0.00000000
C	8.56874493	24.82527943	0.00000000
C	8.58565540	26.27723156	0.00000000
C	9.82125974	-26.93644264	0.00000000
C	9.78331818	-24.13254718	0.00000000
C	9.78331624	24.13256432	0.00000000
C	9.82125681	26.93645803	0.00000000
C	11.03209003	-26.23948149	0.00000000
C	11.01157518	-24.79445125	0.00000000
C	11.01157227	24.79446826	0.00000000
C	11.03208660	26.23949756	0.00000000
C	12.28452359	-26.88020779	0.00000000
C	12.23160092	-24.09396296	0.00000000
C	12.23159795	24.09398079	0.00000000
C	12.28451954	26.88022399	0.00000000
C	13.47780337	-26.17433778	0.00000000
C	13.45925783	-24.73119353	0.00000000
C	13.45925431	24.73121139	0.00000000
C	13.47779955	26.17435507	0.00000000
C	14.73256150	-26.85533801	0.00000000
C	14.71721034	-24.00796904	0.00000000
C	14.78427324	-22.62630095	0.00000000
C	14.78427048	22.62631889	0.00000000
C	14.71720703	24.00798710	0.00000000
C	14.73255682	26.85535611	0.00000000
C	15.91154854	-26.17754857	0.00000000
C	15.95468787	-24.75049671	0.00000000
C	16.00373884	-21.92479281	0.00000000
C	16.05003952	-20.53030083	0.00000000
C	16.05003738	20.53031759	0.00000000
C	16.00373603	21.92480993	0.00000000
C	15.95468397	24.75051508	0.00000000
C	15.91154458	26.17756676	0.00000000
C	17.16527457	-24.07471347	0.00000000
C	17.24186964	-22.66996032	0.00000000
C	17.25994636	-19.82953348	0.00000000
C	17.30643570	-18.43014329	0.00000000
C	17.30643397	18.43015759	0.00000000
C	17.25994409	19.82954857	0.00000000
C	17.24186646	22.66997695	0.00000000
C	17.16527079	24.07473074	0.00000000
C	18.45359948	-21.97444136	0.00000000
C	18.50593273	-20.57488602	0.00000000
C	18.51122754	-17.72720840	0.00000000
C	18.55417002	-16.32686365	0.00000000
C	18.55416896	16.32687394	0.00000000
C	18.51122579	17.72722011	0.00000000

C	18.50593016	20.57490017	0.00000000
C	18.45359638	21.97445664	0.00000000
C	19.71577006	-19.86558731	0.00000000
C	19.76165038	-18.46906783	0.00000000
C	19.75598897	-15.61914692	0.00000000
C	19.79378832	-14.21982748	0.00000000
C	19.79378843	14.21983227	0.00000000
C	19.75598812	15.61915378	0.00000000
C	19.76164838	18.46907831	0.00000000
C	19.71576743	19.86559931	0.00000000
C	20.96880758	-17.75341156	0.00000000
C	21.01006453	-16.35684783	0.00000000
C	20.99347967	-13.50594399	0.00000000
C	21.02509353	-12.10835229	0.00000000
C	21.02509521	12.10835059	0.00000000
C	20.99348018	13.50594477	0.00000000
C	21.01006350	16.35685335	0.00000000
C	20.96880561	17.75341909	0.00000000
C	22.21345822	-15.63704794	0.00000000
C	22.25063901	-14.23899172	0.00000000
C	22.22301402	-11.38791133	0.00000000
C	22.24794047	-9.99208105	0.00000000
C	22.24794364	9.99207276	0.00000000
C	22.22301612	11.38790552	0.00000000
C	22.25063941	14.23899117	0.00000000
C	22.21345746	15.63704979	0.00000000
C	23.45003525	-13.51610799	0.00000000
C	23.48302722	-12.11602847	0.00000000
C	23.44419721	-9.26534325	0.00000000
C	23.46237789	-7.87077012	0.00000000
C	23.46238201	7.87075597	0.00000000
C	23.44420065	9.26533121	0.00000000
C	23.48302926	12.11602132	0.00000000
C	23.45003612	13.51610331	0.00000000
C	24.67855320	-11.39034789	0.00000000
C	24.70682409	-9.98826889	0.00000000
C	24.65676408	-7.13879936	0.00000000
C	24.66876625	-5.74406213	0.00000000
C	24.66877043	5.74404333	0.00000000
C	24.65676816	7.13878212	0.00000000
C	24.70682750	9.98825551	0.00000000
C	24.67855571	11.39033664	0.00000000
C	25.89888015	-9.25944283	0.00000000
C	25.92140403	-7.85608972	0.00000000
C	25.86023981	-5.00903845	0.00000000
C	25.86706060	-3.61097692	0.00000000
C	25.86706386	3.61095475	0.00000000
C	25.86024366	5.00901722	0.00000000
C	25.92140819	7.85607114	0.00000000
C	25.89888386	9.25942580	0.00000000
C	27.11086091	-7.12274417	0.00000000
C	27.12603875	-5.72016202	0.00000000
C	27.05396929	-2.87795463	0.00000000
C	27.05564544	-1.47127485	0.00000000
C	27.05564690	1.47125021	0.00000000
C	27.05397197	2.87793043	0.00000000
C	27.12604287	5.72013948	0.00000000
C	27.11086510	7.12272264	0.00000000
C	28.31456554	-4.97901510	0.00000000
C	28.31608945	-3.58160059	0.00000000
C	28.22034915	-0.72550378	0.00000000
C	28.22034993	0.72547766	0.00000000
C	28.31609270	3.58157511	0.00000000
C	28.31456937	4.97899024	0.00000000
C	29.49638517	-2.81568931	0.00000000
C	29.48014486	-1.42910963	0.00000000
C	29.48014651	1.42908204	0.00000000
C	29.49638783	2.81566209	0.00000000
C	30.69565688	-0.68009836	0.00000000
C	30.69565759	0.68006927	0.00000000
H	-31.64174736	-1.23887227	0.00000000
H	-31.64174899	1.23882450	0.00000000
H	-30.46581749	-3.33600910	0.00000000
H	-30.46582100	3.33596376	0.00000000
H	-29.27563773	-5.51435321	0.00000000
H	-29.27564221	5.51431076	0.00000000
H	-28.06892067	-7.66351529	0.00000000
H	-28.06892491	7.66347727	0.00000000
H	-26.85467133	-9.80427230	0.00000000
H	-26.85467416	9.80424048	0.00000000
H	-26.07734679	-0.97086131	0.00000000
H	-26.07734819	0.97082049	0.00000000
H	-25.63212855	-11.93909212	0.00000000

H	-25.63212893	11.93906834	0.00000000
H	-24.90685321	-3.07387466	0.00000000
H	-24.90685624	3.07383705	0.00000000
H	-24.40143853	-14.06862734	0.00000000
H	-24.40143583	14.06861271	0.00000000
H	-23.71098860	-5.20286667	0.00000000
H	-23.71099229	5.20283342	0.00000000
H	-23.16270329	-16.19324877	0.00000000
H	-23.16269748	16.19324329	0.00000000
H	-22.50673499	-7.32578029	0.00000000
H	-22.50673792	7.32575338	0.00000000
H	-21.91588495	-18.31324994	0.00000000
H	-21.91587661	18.31325256	0.00000000
H	-21.29441823	-9.44334801	0.00000000
H	-21.29441912	9.44332925	0.00000000
H	-20.66067315	-20.42902918	0.00000000
H	-20.66066308	20.42903831	0.00000000
H	-20.07371585	-11.55589233	0.00000000
H	-20.07371387	11.55588278	0.00000000
H	-19.39559443	-22.54267471	0.00000000
H	-19.39558311	22.54268875	0.00000000
H	-18.84453933	-13.66373470	0.00000000
H	-18.84453441	13.66373424	0.00000000
H	-18.09909964	-24.65655699	0.00000000
H	-18.09908752	24.65657380	0.00000000
H	-17.60698492	-15.76730251	0.00000000
H	-17.60697760	15.76730980	0.00000000
H	-16.86765628	-26.71897031	0.00000000
H	-16.86764340	26.71898866	0.00000000
H	-16.36126148	-17.86722286	0.00000000
H	-16.36125257	17.86723586	0.00000000
H	-15.10693345	-19.96373033	0.00000000
H	-15.10692358	19.96374686	0.00000000
H	-14.71921322	-27.95400755	0.00000000
H	-14.71919784	27.95402615	0.00000000
H	-13.86326938	-22.02688008	0.00000000
H	-13.86325844	22.02689881	0.00000000
H	-12.31732824	-27.97995551	0.00000000
H	-12.17721640	-22.99646657	0.00000000
H	-12.17720450	22.99648562	0.00000000
H	-12.31731326	27.97997217	0.00000000
H	-9.83803949	-28.03640317	0.00000000
H	-9.76848841	-23.03245660	0.00000000
H	-9.76847657	23.03247485	0.00000000
H	-9.83802532	28.03641867	0.00000000
H	-7.37534456	-28.06583964	0.00000000
H	-7.32523751	-23.06124566	0.00000000
H	-7.32522622	23.06126310	0.00000000
H	-7.37533162	28.06585426	0.00000000
H	-4.91617602	-28.08629828	0.00000000
H	-4.88296501	-23.08146889	0.00000000
H	-4.88295475	23.08148584	0.00000000
H	-4.91616495	28.08631245	0.00000000
H	-2.45792006	-28.09833936	0.00000000
H	-2.44134930	-23.09374795	0.00000000
H	-2.44134076	23.09376472	0.00000000
H	-2.45791123	28.09835338	0.00000000
H	-0.00000549	-28.10231626	0.00000000
H	-0.00000615	-23.09786645	0.00000000
H	0.00000010	23.09788322	0.00000000
H	0.00000082	28.10233018	0.00000000
H	2.45790947	-28.09833867	0.00000000
H	2.44133745	-23.09374737	0.00000000
H	2.44134121	23.09376423	0.00000000
H	2.45791313	28.09835246	0.00000000
H	4.91616643	-28.08629697	0.00000000
H	4.88295432	-23.08146776	0.00000000
H	4.88295588	23.08148484	0.00000000
H	4.91616741	28.08631069	0.00000000
H	7.37533627	-28.06583783	0.00000000
H	7.32522835	-23.06124401	0.00000000
H	7.32522820	23.06126157	0.00000000
H	7.37533476	28.06585181	0.00000000
H	9.83803251	-28.03640102	0.00000000
H	9.76848075	-23.03245453	0.00000000
H	9.76847923	23.03247279	0.00000000
H	9.83802909	28.03641565	0.00000000
H	12.31732225	-27.97995318	0.00000000
H	12.17720993	-22.99646417	0.00000000
H	12.17720751	22.99648305	0.00000000
H	12.31731728	27.97996864	0.00000000
H	13.86326515	-22.02687619	0.00000000
H	13.86326308	22.02689522	0.00000000

H	14.71920798	-27.95400521	0.00000000
H	14.71920163	27.95402249	0.00000000
H	15.10692988	-19.96372527	0.00000000
H	15.10692853	19.96374335	0.00000000
H	16.36125890	-17.86721624	0.00000000
H	16.36125787	17.86723237	0.00000000
H	16.86765185	-26.71896740	0.00000000
H	16.86764743	26.71898463	0.00000000
H	17.60698348	-15.76729416	0.00000000
H	17.60698320	15.76730645	0.00000000
H	18.09909558	-24.65655334	0.00000000
H	18.09909108	24.65656991	0.00000000
H	18.84453897	-13.66372477	0.00000000
H	18.84454003	13.66373144	0.00000000
H	19.39559106	-22.54267004	0.00000000
H	19.39558672	22.54268498	0.00000000
H	20.07371633	-11.55588127	0.00000000
H	20.07371914	11.55588111	0.00000000
H	20.66067084	-20.42902317	0.00000000
H	20.66066686	20.42903453	0.00000000
H	21.29441923	-9.44333648	0.00000000
H	21.29442364	9.44332926	0.00000000
H	21.91588391	-18.31324249	0.00000000
H	21.91588053	18.31324906	0.00000000
H	22.50673617	-7.32576892	0.00000000
H	22.50674144	7.32575544	0.00000000
H	23.16270357	-16.19323998	0.00000000
H	23.16270148	16.19324047	0.00000000
H	23.71098974	-5.20285587	0.00000000
H	23.71099478	5.20283752	0.00000000
H	24.40143992	-14.06861758	0.00000000
H	24.40143968	14.06861116	0.00000000
H	24.90685426	-3.07386455	0.00000000
H	24.90685789	3.07384285	0.00000000
H	25.63213065	-11.93908192	0.00000000
H	25.63213237	11.93906862	0.00000000
H	26.07734781	-0.97085179	0.00000000
H	26.07734936	0.97082735	0.00000000
H	26.85467371	-9.80426213	0.00000000
H	26.85467699	9.80424290	0.00000000
H	28.06892296	-7.66350545	0.00000000
H	28.06892705	7.66348179	0.00000000
H	29.27563970	-5.51434385	0.00000000
H	29.27564373	5.51431706	0.00000000
H	30.46581916	-3.33600007	0.00000000
H	30.46582218	3.33597130	0.00000000
H	31.64174858	-1.23886321	0.00000000
H	31.64175001	1.23883259	0.00000000

14-HGQR (singlet)

C	-33.15994342	-0.67991698	0.00000000
C	-33.15994303	0.68016623	0.00000000
C	-31.96096519	-2.81526986	0.00000000
C	-31.94437816	-1.42884071	0.00000000
C	-31.94437786	1.42908938	0.00000000
C	-31.96096459	2.81551636	0.00000000
C	-30.78008617	-4.97869134	0.00000000
C	-30.78082432	-3.58146649	0.00000000
C	-30.68436059	-0.72543577	0.00000000
C	-30.68436061	0.72568536	0.00000000
C	-30.78082543	3.58171105	0.00000000
C	-30.78008810	4.97893149	0.00000000
C	-29.57782565	-7.12285185	0.00000000
C	-29.59187436	-5.72044632	0.00000000
C	-29.51839443	-2.87828090	0.00000000
C	-29.51983281	-1.47143497	0.00000000
C	-29.51983423	1.47168342	0.00000000
C	-29.51839754	2.87852707	0.00000000
C	-29.59187957	5.72068218	0.00000000
C	-29.57783199	7.12308073	0.00000000
C	-28.36772239	-9.26030423	0.00000000
C	-28.38883339	-7.85701127	0.00000000
C	-28.32563258	-5.01007537	0.00000000
C	-28.33185169	-3.61181306	0.00000000
C	-28.33185856	3.61205653	0.00000000
C	-28.32564158	5.01031351	0.00000000
C	-28.38884484	7.85723288	0.00000000
C	-28.36773541	9.26051592	0.00000000
C	-27.14966898	-11.39231319	0.00000000
C	-27.17635053	-9.99002197	0.00000000
C	-27.12364996	-7.14079381	0.00000000
C	-27.13468090	-5.74587774	0.00000000
C	-27.13469543	5.74611070	0.00000000
C	-27.12366718	7.14101813	0.00000000

C	-27.17637066	9.99022348	0.00000000
C	-27.14969127	11.39250181	0.00000000
C	-25.92380869	-13.51952097	0.00000000
C	-25.95516966	-12.11881516	0.00000000
C	-25.91311220	-9.26850775	0.00000000
C	-25.92987686	-7.87389643	0.00000000
C	-25.92990150	7.87411295	0.00000000
C	-25.91314004	9.26871240	0.00000000
C	-25.95520092	12.11899107	0.00000000
C	-25.92384284	13.51968134	0.00000000
C	-24.69034340	-15.64218063	0.00000000
C	-24.72592019	-14.24311571	0.00000000
C	-24.69447506	-11.39244446	0.00000000
C	-24.71747450	-9.99689058	0.00000000
C	-24.71751148	9.99708458	0.00000000
C	-24.69451567	11.39262385	0.00000000
C	-24.72596483	14.24326161	0.00000000
C	-24.69039173	15.64230905	0.00000000
C	-23.44945390	-17.76037162	0.00000000
C	-23.48896404	-16.36285622	0.00000000
C	-23.46790840	-13.51208585	0.00000000
C	-23.49711573	-12.11522012	0.00000000
C	-23.49716683	12.11538629	0.00000000
C	-23.46796348	13.51223548	0.00000000
C	-23.48902395	16.36296940	0.00000000
C	-23.44951828	17.76046632	0.00000000
C	-22.20115405	-19.87424632	0.00000000
C	-22.24451845	-18.47788935	0.00000000
C	-22.23360127	-15.62728229	0.00000000
C	-22.26876715	-14.22902647	0.00000000
C	-22.26883355	14.22916106	0.00000000
C	-22.23367198	15.62739944	0.00000000
C	-22.24459497	18.47796919	0.00000000
C	-22.20123570	19.87430793	0.00000000
C	-20.94514046	-21.98420310	0.00000000
C	-20.99284039	-20.58773653	0.00000000
C	-20.99192766	-17.73799324	0.00000000
C	-21.03259942	-16.33840358	0.00000000
C	-21.03268170	16.33850490	0.00000000
C	-20.99201455	17.73807733	0.00000000
C	-20.99293404	20.58778481	0.00000000
C	-20.94523951	21.98423509	0.00000000
C	-19.68065925	-24.09115475	0.00000000
C	-19.73444997	-22.69162374	0.00000000
C	-19.74358580	-19.84401241	0.00000000
C	-19.78884087	-18.44364634	0.00000000
C	-19.78893892	18.44371510	0.00000000
C	-19.74368864	19.84406522	0.00000000
C	-19.73455997	22.69164474	0.00000000
C	-19.68077364	24.09116297	0.00000000
C	-18.39074561	-26.18989015	0.00000000
C	-18.46832235	-24.78518841	0.00000000
C	-18.48934752	-21.94475447	0.00000000
C	-18.53768513	-20.54543690	0.00000000
C	-18.53779796	20.54547630	0.00000000
C	-18.48946504	21.94477997	0.00000000
C	-18.46844558	24.78518759	0.00000000
C	-18.39087123	26.18988023	0.00000000
C	-17.13625551	-28.29174242	0.00000000
C	-17.17981248	-26.86471380	0.00000000
C	-17.23083119	-24.03886297	0.00000000
C	-17.27855352	-22.64443782	0.00000000
C	-17.27867929	22.64445293	0.00000000
C	-17.23096048	24.03886620	0.00000000
C	-17.17994623	26.86469830	0.00000000
C	-17.13639298	28.29172130	0.00000000
C	-15.95708171	-28.96900269	0.00000000
C	-15.94273452	-26.12130769	0.00000000
C	-16.01075064	-24.73968855	0.00000000
C	-16.01088590	24.73968383	0.00000000
C	-15.94287422	26.12129321	0.00000000
C	-15.95722641	28.96897952	0.00000000
C	-14.70268458	-28.28735753	0.00000000
C	-14.68434836	-26.84410147	0.00000000
C	-14.68449341	26.84408534	0.00000000
C	-14.70283379	28.28733673	0.00000000
C	-13.50948928	-28.99304032	0.00000000
C	-13.45677109	-26.20672431	0.00000000
C	-13.45692312	26.20671086	0.00000000
C	-13.50964552	28.99301927	0.00000000
C	-12.25712562	-28.35217294	0.00000000
C	-12.23646098	-26.90709872	0.00000000
C	-12.23661984	26.90708535	0.00000000

C	-12.25728578	28.35215547	0.00000000
C	-11.04671115	-29.04942108	0.00000000
C	-11.00808274	-26.24546429	0.00000000
C	-11.00824663	26.24545351	0.00000000
C	-11.04687746	29.04940427	0.00000000
C	-9.81093147	-28.39046730	0.00000000
C	-9.79344468	-26.93850604	0.00000000
C	-9.79361387	26.93849488	0.00000000
C	-9.81110162	28.39045306	0.00000000
C	-8.58993505	-29.07972263	0.00000000
C	-8.56035569	-26.27516653	0.00000000
C	-8.56052844	26.27515685	0.00000000
C	-8.59010974	29.07970869	0.00000000
C	-7.35982521	-28.41727037	0.00000000
C	-7.34674878	-26.96330542	0.00000000
C	-7.34692521	26.96329544	0.00000000
C	-7.36000266	28.41725848	0.00000000
C	-6.13506322	-29.10182371	0.00000000
C	-6.11405147	-26.29716849	0.00000000
C	-6.11422977	26.29715944	0.00000000
C	-6.13524331	29.10181253	0.00000000
C	-4.90707838	-28.43608166	0.00000000
C	-4.89838991	-26.98115615	0.00000000
C	-4.89856991	26.98114691	0.00000000
C	-4.90725926	28.43607191	0.00000000
C	-3.68089850	-29.11626266	0.00000000
C	-3.66833207	-26.31201198	0.00000000
C	-3.66851206	26.31200317	0.00000000
C	-3.68107982	29.11625406	0.00000000
C	-2.45360309	-28.44717091	0.00000000
C	-2.44926749	-26.99201866	0.00000000
C	-2.44944706	26.99200983	0.00000000
C	-2.45378318	28.44716292	0.00000000
C	-1.22690877	-29.12340310	0.00000000
C	-1.22272569	-26.31948436	0.00000000
C	-1.22290355	26.31947544	0.00000000
C	-1.22708724	29.12339633	0.00000000
C	0.00008103	-28.45083189	0.00000000
C	0.00008197	-26.99566755	0.00000000
C	-0.00009382	26.99565884	0.00000000
C	-0.00009486	28.45082491	0.00000000
C	1.22706986	-29.12340439	0.00000000
C	1.22289028	-26.31948606	0.00000000
C	1.22271716	26.31947692	0.00000000
C	1.22689681	29.12339838	0.00000000
C	2.45376461	-28.44717363	0.00000000
C	2.44943083	-26.99202184	0.00000000
C	2.44926047	26.99201305	0.00000000
C	2.45359448	28.44716682	0.00000000
C	3.68105853	-29.11626646	0.00000000
C	3.66849540	-26.31201701	0.00000000
C	3.66832781	26.31200765	0.00000000
C	3.68089142	29.11626024	0.00000000
C	4.90723821	-28.43608692	0.00000000
C	4.89855141	-26.98116234	0.00000000
C	4.89838645	26.98115346	0.00000000
C	4.90707362	28.43607978	0.00000000
C	6.13522103	-29.10182973	0.00000000
C	6.11421218	-26.29717661	0.00000000
C	6.11404973	26.29716709	0.00000000
C	6.13505892	29.10182293	0.00000000
C	7.35998206	-28.41727780	0.00000000
C	7.34690706	-26.96331429	0.00000000
C	7.34674687	26.96330553	0.00000000
C	7.35982200	28.41727049	0.00000000
C	8.59008936	-29.07973041	0.00000000
C	8.56051227	-26.27517732	0.00000000
C	8.56035458	26.27516798	0.00000000
C	8.58993112	29.07972351	0.00000000
C	9.81108393	-28.39047631	0.00000000
C	9.79359816	-26.93851704	0.00000000
C	9.79344255	26.93850890	0.00000000
C	9.81092772	28.39046957	0.00000000
C	11.04686072	-29.04942976	0.00000000
C	11.00823359	-26.24547711	0.00000000
C	11.00808073	26.24546860	0.00000000
C	11.04670605	29.04942414	0.00000000
C	12.25727208	-28.35218231	0.00000000
C	12.23660792	-26.90711087	0.00000000
C	12.23645743	26.90710410	0.00000000
C	12.25712039	28.35217748	0.00000000
C	13.50963270	-28.99304810	0.00000000
C	13.45691411	-26.20673781	0.00000000

C	13.45676735	26.20673104	0.00000000
C	13.50948285	28.99304587	0.00000000
C	14.70282369	-28.28736649	0.00000000
C	14.68448585	-26.84411398	0.00000000
C	14.68434300	26.84410916	0.00000000
C	14.70267837	28.28736462	0.00000000
C	15.95721675	-28.96901092	0.00000000
C	15.94286853	-26.12132252	0.00000000
C	16.01088256	-24.73971112	0.00000000
C	16.01074534	24.73969805	0.00000000
C	15.94272911	26.12131641	0.00000000
C	15.95707455	28.96901069	0.00000000
C	17.13638571	-28.29175280	0.00000000
C	17.17994091	-26.86472865	0.00000000
C	17.23095887	-24.03889260	0.00000000
C	17.27867989	-22.64447736	0.00000000
C	17.27854833	22.64444840	0.00000000
C	17.23082572	24.03887322	0.00000000
C	17.17980629	26.86472315	0.00000000
C	17.13624863	28.29175142	0.00000000
C	18.39086779	-26.18990989	0.00000000
C	18.46844404	-24.78521550	0.00000000
C	18.48946703	-21.94480276	0.00000000
C	18.53780202	-20.54549736	0.00000000
C	18.53768075	20.54544808	0.00000000
C	18.48934260	21.94476581	0.00000000
C	18.46831650	24.78519861	0.00000000
C	18.39073935	26.18990004	0.00000000
C	19.68077399	-24.09118941	0.00000000
C	19.73456221	-22.69166859	0.00000000
C	19.74369353	-19.84408381	0.00000000
C	19.78894517	-18.44373204	0.00000000
C	19.78883883	18.44365796	0.00000000
C	19.74358235	19.84402453	0.00000000
C	19.73444451	22.69163531	0.00000000
C	19.68065309	24.09116565	0.00000000
C	20.94524371	-21.98425677	0.00000000
C	20.99293960	-20.58780347	0.00000000
C	20.99202110	-17.73809125	0.00000000
C	21.03268870	-16.33851724	0.00000000
C	21.03260118	16.33841564	0.00000000
C	20.99192703	17.73800593	0.00000000
C	20.99283601	20.58774967	0.00000000
C	20.94513466	21.98421553	0.00000000
C	22.20124265	-19.87432368	0.00000000
C	22.24460246	-18.47798221	0.00000000
C	22.23367894	-15.62740849	0.00000000
C	22.26884033	-14.22916846	0.00000000
C	22.26877347	14.22903930	0.00000000
C	22.23360460	15.62729551	0.00000000
C	22.24451657	18.47790382	0.00000000
C	22.20114987	19.87426052	0.00000000
C	23.44952638	-17.76047606	0.00000000
C	23.48903199	-16.36297698	0.00000000
C	23.46797019	-13.51223950	0.00000000
C	23.49717313	-12.11538850	0.00000000
C	23.49712636	12.11523434	0.00000000
C	23.46791598	13.51209995	0.00000000
C	23.48896583	16.36287154	0.00000000
C	23.44945283	17.76038717	0.00000000
C	24.69039981	-15.64231323	0.00000000
C	24.72597264	-14.24326406	0.00000000
C	24.69452213	-11.39262282	0.00000000
C	24.71751766	-9.99708166	0.00000000
C	24.71748838	9.99690667	0.00000000
C	24.69448632	11.39245993	0.00000000
C	24.72592599	14.24313170	0.00000000
C	24.69034628	15.64219700	0.00000000
C	25.92385046	-13.51968050	0.00000000
C	25.95520837	-12.11898874	0.00000000
C	25.91314667	-9.26870672	0.00000000
C	25.92990818	-7.87410547	0.00000000
C	25.92989257	7.87391435	0.00000000
C	25.91312600	9.26852480	0.00000000
C	25.95517903	12.11883196	0.00000000
C	25.92381544	13.51953799	0.00000000
C	27.14969851	-11.39249652	0.00000000
C	27.17637800	-9.99021693	0.00000000
C	27.12367451	-7.14100859	0.00000000
C	27.13470323	-5.74609962	0.00000000
C	27.13469701	5.74589690	0.00000000
C	27.12366498	7.14081216	0.00000000
C	27.17636255	9.99003965	0.00000000

C	27.14967892	11.39233088	0.00000000
C	28.36774261	-9.26050697	0.00000000
C	28.38885250	-7.85722295	0.00000000
C	28.32565012	-5.01030112	0.00000000
C	28.33186796	-3.61204296	0.00000000
C	28.33186697	3.61183254	0.00000000
C	28.32564760	5.01009433	0.00000000
C	28.38884701	7.85702952	0.00000000
C	28.36773464	9.26032240	0.00000000
C	29.57783963	-7.12306907	0.00000000
C	29.59188802	-5.72066984	0.00000000
C	29.51840762	-2.87851275	0.00000000
C	29.51984523	-1.47166845	0.00000000
C	29.51984612	1.47145358	0.00000000
C	29.51840842	2.87829937	0.00000000
C	29.59188850	5.72046448	0.00000000
C	29.57783920	7.12286996	0.00000000
C	30.78009661	-4.97891801	0.00000000
C	30.78083493	-3.58169714	0.00000000
C	30.68437192	-0.72566956	0.00000000
C	30.68437271	0.72545227	0.00000000
C	30.78083794	3.58148375	0.00000000
C	30.78009997	4.97870863	0.00000000
C	31.96097407	-2.81550184	0.00000000
C	31.94438844	-1.42907446	0.00000000
C	31.94439060	1.42885620	0.00000000
C	31.96097808	2.81528568	0.00000000
C	33.15995406	-0.68015222	0.00000000
C	33.15995535	0.67993174	0.00000000
H	-34.10601033	-1.23875218	0.00000000
H	-34.10600980	1.23900064	0.00000000
H	-32.93050652	-3.33539648	0.00000000
H	-32.93050532	3.33564124	0.00000000
H	-31.74142953	-5.51355350	0.00000000
H	-31.74143006	5.51379113	0.00000000
H	-30.53627067	-7.66294820	0.00000000
H	-30.53627437	7.66317430	0.00000000
H	-29.32401843	-9.80425656	0.00000000
H	-29.32402748	9.80446526	0.00000000
H	-28.54145388	-0.97112410	0.00000000
H	-28.54145663	0.97137100	0.00000000
H	-28.10387501	-11.93997478	0.00000000
H	-28.10389202	11.94016035	0.00000000
H	-27.37138065	-3.07515413	0.00000000
H	-27.37139008	3.07539721	0.00000000
H	-26.87598191	-14.07073904	0.00000000
H	-26.87600968	14.07089638	0.00000000
H	-26.17650663	-5.20535577	0.00000000
H	-26.17652514	5.20558890	0.00000000
H	-25.64051613	-16.19683905	0.00000000
H	-25.64055735	16.19696442	0.00000000
H	-24.97370708	-7.32980099	0.00000000
H	-24.97373696	7.33001828	0.00000000
H	-24.39763084	-18.31840371	0.00000000
H	-24.39768786	18.31849536	0.00000000
H	-23.76329613	-9.44926300	0.00000000
H	-23.76333938	9.44945835	0.00000000
H	-23.14732189	-20.43562647	0.00000000
H	-23.14739638	20.43568519	0.00000000
H	-22.54495888	-11.56405563	0.00000000
H	-22.54501690	11.56422371	0.00000000
H	-21.88929330	-22.54890527	0.00000000
H	-21.88938579	22.54893489	0.00000000
H	-21.31862952	-13.67438780	0.00000000
H	-21.31870306	13.67452479	0.00000000
H	-20.62206304	-24.66037310	0.00000000
H	-20.62217154	24.66038016	0.00000000
H	-20.08444244	-15.78041369	0.00000000
H	-20.08453159	15.78051786	0.00000000
H	-19.32419418	-26.77234085	0.00000000
H	-19.32431530	26.77232995	0.00000000
H	-18.84258331	-17.88248567	0.00000000
H	-18.84268749	17.88255772	0.00000000
H	-18.09221234	-28.83344797	0.00000000
H	-18.09234749	28.83342396	0.00000000
H	-17.59326596	-19.98121888	0.00000000
H	-17.59338380	19.98126196	0.00000000
H	-16.33602294	-22.07687865	0.00000000
H	-16.33615266	22.07689737	0.00000000
H	-15.94316257	-30.06767651	0.00000000
H	-15.94330725	30.06765065	0.00000000
H	-15.09006639	-24.13972145	0.00000000
H	-15.09020348	24.13972148	0.00000000

H	-13.54211869	-30.09279770	0.00000000
H	-13.40263749	-25.10918836	0.00000000
H	-13.40278884	25.10917852	0.00000000
H	-13.54227650	30.09277424	0.00000000
H	-11.06375215	-30.14938252	0.00000000
H	-10.99299244	-25.14536500	0.00000000
H	-10.99315455	25.14535712	0.00000000
H	-11.06391958	30.14936356	0.00000000
H	-8.60184074	-30.17977670	0.00000000
H	-8.54898295	-25.17512348	0.00000000
H	-8.54915412	25.17511592	0.00000000
H	-8.60201557	30.17976105	0.00000000
H	-6.14342035	-30.20193851	0.00000000
H	-6.10590835	-25.19708188	0.00000000
H	-6.10608507	25.19707415	0.00000000
H	-6.14360008	30.20192632	0.00000000
H	-3.68584030	-30.21642261	0.00000000
H	-3.66339760	-25.21188428	0.00000000
H	-3.66357611	25.21187596	0.00000000
H	-3.68602106	30.21641380	0.00000000
H	-1.22854336	-30.22358748	0.00000000
H	-1.22107269	-25.21933299	0.00000000
H	-1.22124950	25.21932380	0.00000000
H	-1.22872134	30.22358121	0.00000000
H	1.22870266	-30.22358858	0.00000000
H	1.22123803	-25.21933494	0.00000000
H	1.22106535	25.21932497	0.00000000
H	1.22852987	30.22358352	0.00000000
H	3.68599863	-30.21642582	0.00000000
H	3.66356162	-25.21189006	0.00000000
H	3.66339409	25.21187953	0.00000000
H	3.68583153	30.21642074	0.00000000
H	6.14357667	-30.20194357	0.00000000
H	6.10606960	-25.19709127	0.00000000
H	6.10590729	25.19708032	0.00000000
H	6.14341437	30.20193791	0.00000000
H	8.60199390	-30.17978317	0.00000000
H	8.54913984	-25.17513610	0.00000000
H	8.54898261	25.17512502	0.00000000
H	8.60183533	30.17977743	0.00000000
H	11.06390120	-30.14938956	0.00000000
H	10.99314316	-25.14538026	0.00000000
H	10.99299128	25.14536959	0.00000000
H	11.06374596	30.14938529	0.00000000
H	13.54226187	-30.09280355	0.00000000
H	13.40278057	-25.10920494	0.00000000
H	13.40263527	25.10919539	0.00000000
H	13.54211132	30.09280297	0.00000000
H	15.09020036	-24.13974756	0.00000000
H	15.09006144	24.13973117	0.00000000
H	15.94329614	-30.06768244	0.00000000
H	15.94315435	30.06768416	0.00000000
H	16.33615311	-22.07692143	0.00000000
H	16.33601837	22.07688883	0.00000000
H	17.59338762	-19.98128320	0.00000000
H	17.59326220	19.98122944	0.00000000
H	18.09234009	-28.83345655	0.00000000
H	18.09220492	28.83345716	0.00000000
H	18.84269338	-17.88257540	0.00000000
H	18.84258203	17.88249638	0.00000000
H	19.32431197	-26.77236033	0.00000000
H	19.32418724	26.77235093	0.00000000
H	20.08453817	-15.78053126	0.00000000
H	20.08444514	15.78042475	0.00000000
H	20.62217218	-24.66040728	0.00000000
H	20.62205594	24.66038430	0.00000000
H	21.31870941	-13.67453331	0.00000000
H	21.31863698	13.67439978	0.00000000
H	21.88939035	-22.54895720	0.00000000
H	21.88928642	22.54891803	0.00000000
H	22.54502280	-11.56422694	0.00000000
H	22.54497085	11.56406925	0.00000000
H	23.14740374	-20.43570116	0.00000000
H	23.14731648	20.43564132	0.00000000
H	23.76334525	-9.44945624	0.00000000
H	23.76331148	9.44927875	0.00000000
H	24.39769639	-18.31850469	0.00000000
H	24.39762838	18.31842034	0.00000000
H	24.97374350	-7.33001132	0.00000000
H	24.97372423	7.32981884	0.00000000
H	25.64056585	-16.19696768	0.00000000
H	25.64051748	16.19685678	0.00000000
H	26.17653302	-5.20557807	0.00000000

H	26.17652396	5.20537516	0.00000000
H	26.87601761	-14.07089441	0.00000000
H	26.87598711	14.07075733	0.00000000
H	27.37139976	-3.07538381	0.00000000
H	27.37139675	3.07517420	0.00000000
H	28.10389938	-11.94015402	0.00000000
H	28.10388359	11.93999328	0.00000000
H	28.54146777	-0.97135707	0.00000000
H	28.54146733	0.97114421	0.00000000
H	29.32403457	-9.80445553	0.00000000
H	29.32402965	9.80427489	0.00000000
H	30.53628169	-7.66316217	0.00000000
H	30.53628359	7.66296586	0.00000000
H	31.74143813	-5.51377741	0.00000000
H	31.74144312	5.51356981	0.00000000
H	32.93051413	-3.33562703	0.00000000
H	32.93051928	3.33541139	0.00000000
H	34.10601973	-1.23898767	0.00000000
H	34.10602189	1.23876668	0.00000000

15-HGQR (singlet)

C	-35.62692609	-0.68000782	0.00000000
C	-35.62692399	0.68003541	0.00000000
C	-34.42814864	-2.81513536	0.00000000
C	-34.41131948	-1.42879695	0.00000000
C	-34.41131533	1.42882104	0.00000000
C	-34.42814008	2.81515914	0.00000000
C	-33.24805688	-4.97863817	0.00000000
C	-33.24819383	-3.58155961	0.00000000
C	-33.15112065	-0.72554071	0.00000000
C	-33.15111839	0.72556152	0.00000000
C	-33.24818334	3.58157975	0.00000000
C	-33.24804244	4.97865750	0.00000000
C	-32.04699532	-7.12312379	0.00000000
C	-32.06013685	-5.72089641	0.00000000
C	-31.98547558	-2.87872529	0.00000000
C	-31.98672264	-1.47179486	0.00000000
C	-31.98671837	1.47181213	0.00000000
C	-31.98546757	2.87874232	0.00000000
C	-32.06012111	5.72091230	0.00000000
C	-32.04697600	7.12313870	0.00000000
C	-30.83848175	-9.26115557	0.00000000
C	-30.85840773	-7.85803133	0.00000000
C	-30.79348201	-5.01111578	0.00000000
C	-30.79924120	-3.61272002	0.00000000
C	-30.79923183	3.61273372	0.00000000
C	-30.79346903	5.01112903	0.00000000
C	-30.85838750	7.85804325	0.00000000
C	-30.83845843	9.26116685	0.00000000
C	-29.62236481	-11.39401366	0.00000000
C	-29.64764178	-9.99178130	0.00000000
C	-29.59269449	-7.14264927	0.00000000
C	-29.60300008	-5.74756820	0.00000000
C	-29.60298579	5.74757829	0.00000000
C	-29.59267681	7.14265899	0.00000000
C	-29.64761762	9.99179042	0.00000000
C	-29.62233795	11.39402296	0.00000000
C	-28.39875040	-13.52236321	0.00000000
C	-28.42859257	-12.12145343	0.00000000
C	-28.38379423	-9.27136798	0.00000000
C	-28.39951830	-7.87661353	0.00000000
C	-28.39949924	7.87662072	0.00000000
C	-28.38377199	9.27137516	0.00000000
C	-28.42856455	12.12146152	0.00000000
C	-28.39871994	13.52237240	0.00000000
C	-27.16783067	-15.64645213	0.00000000
C	-27.20190532	-14.24677824	0.00000000
C	-27.16724792	-11.39646851	0.00000000
C	-27.18881949	-10.00091413	0.00000000
C	-27.18879553	10.00091963	0.00000000
C	-27.16722097	11.39647464	0.00000000
C	-27.20187323	14.24678700	0.00000000
C	-27.16779634	15.64646248	0.00000000
C	-25.92985675	-17.76629262	0.00000000
C	-25.96794597	-16.36779137	0.00000000
C	-25.94320349	-13.51741663	0.00000000
C	-25.97053208	-12.12086720	0.00000000
C	-25.97050300	12.12087254	0.00000000
C	-25.94317169	13.51742330	0.00000000
C	-25.96790995	16.36780145	0.00000000
C	-25.92981897	17.76630380	0.00000000
C	-24.68503650	-19.88187681	0.00000000
C	-24.72688837	-18.48459244	0.00000000
C	-24.71175258	-15.63410537	0.00000000

C	-24.74462045	-14.23661554	0.00000000
C	-24.74458629	14.23662204	0.00000000
C	-24.71171635	15.63411345	0.00000000
C	-24.72684955	18.48460275	0.00000000
C	-24.68499696	19.88188676	0.00000000
C	-23.43339325	-21.99336819	0.00000000
C	-23.47886071	-20.59708664	0.00000000
C	-23.47306276	-17.74664308	0.00000000
C	-23.51126786	-16.34815434	0.00000000
C	-23.51122966	16.34816225	0.00000000
C	-23.47302368	17.74665194	0.00000000
C	-23.47882138	20.59709473	0.00000000
C	-23.43335459	21.99337429	0.00000000
C	-22.17461996	-24.10121122	0.00000000
C	-22.22410709	-22.70478734	0.00000000
C	-22.22752252	-19.85509863	0.00000000
C	-22.27072942	-18.45552131	0.00000000
C	-22.27068939	18.45552925	0.00000000
C	-22.22748309	19.85510616	0.00000000
C	-22.22406970	22.70479090	0.00000000
C	-22.17458425	24.10121208	0.00000000
C	-20.90796729	-26.20636497	0.00000000
C	-20.96316659	-24.80689878	0.00000000
C	-20.97585573	-21.95927845	0.00000000
C	-21.02323561	-20.55901590	0.00000000
C	-21.02319650	20.55902143	0.00000000
C	-20.97581829	21.95928221	0.00000000
C	-20.96313222	24.80689703	0.00000000
C	-20.90793467	26.20636106	0.00000000
C	-19.61647824	-28.30363505	0.00000000
C	-19.69505769	-26.89904798	0.00000000
C	-19.71885408	-24.05857245	0.00000000
C	-19.76895368	-22.65935049	0.00000000
C	-19.76891751	22.65935146	0.00000000
C	-19.71881962	24.05857096	0.00000000
C	-19.69502562	26.89904166	0.00000000
C	-19.61644727	28.30362758	0.00000000
C	-18.36118112	-30.40453599	0.00000000
C	-18.40517811	-28.97756949	0.00000000
C	-18.45815222	-26.15159404	0.00000000
C	-18.50727577	-24.75721382	0.00000000
C	-18.50724282	24.75720927	0.00000000
C	-18.45812031	26.15158684	0.00000000
C	-18.40514721	28.97755913	0.00000000
C	-18.36114802	30.40452539	0.00000000
C	-17.18175961	-31.08124203	0.00000000
C	-17.16850192	-28.23330313	0.00000000
C	-17.23752261	-26.85168576	0.00000000
C	-17.23749170	26.85167583	0.00000000
C	-17.16847188	28.23329103	0.00000000
C	-17.18172775	31.08122895	0.00000000
C	-15.92779654	-30.39889555	0.00000000
C	-15.90979502	-28.95553524	0.00000000
C	-15.90976557	28.95552117	0.00000000
C	-15.92776549	30.39888160	0.00000000
C	-14.73455757	-31.10428701	0.00000000
C	-14.68227545	-28.31793973	0.00000000
C	-14.68224942	28.31792316	0.00000000
C	-14.73452784	31.10427217	0.00000000
C	-13.48235016	-30.46323302	0.00000000
C	-13.46169990	-29.01807667	0.00000000
C	-13.46167513	29.01805841	0.00000000
C	-13.48232339	30.46321551	0.00000000
C	-12.27218999	-31.16058139	0.00000000
C	-12.23323474	-28.35658664	0.00000000
C	-12.23321340	28.35656569	0.00000000
C	-12.27216424	31.16056291	0.00000000
C	-11.03633201	-30.50178033	0.00000000
C	-11.01848149	-29.04975083	0.00000000
C	-11.01846151	29.04972861	0.00000000
C	-11.03630970	30.50175940	0.00000000
C	-9.81582910	-31.19148234	0.00000000
C	-9.78516542	-28.38688826	0.00000000
C	-9.78514818	28.38686307	0.00000000
C	-9.81580784	31.19146067	0.00000000
C	-8.58543206	-30.52950374	0.00000000
C	-8.57160382	-29.07549383	0.00000000
C	-8.57158735	29.07546789	0.00000000
C	-8.58541379	30.52947975	0.00000000
C	-7.36135472	-31.21485346	0.00000000
C	-7.33849990	-28.41015425	0.00000000
C	-7.33848573	28.41012544	0.00000000
C	-7.36133731	31.21482923	0.00000000

C	-6.13295204	-30.54987969	0.00000000
C	-6.12311711	-29.09497412	0.00000000
C	-6.12310330	29.09494507	0.00000000
C	-6.13293685	30.54985304	0.00000000
C	-4.90753587	-31.23121382	0.00000000
C	-4.89236020	-28.42694715	0.00000000
C	-4.89234828	28.42691567	0.00000000
C	-4.90752148	31.23118745	0.00000000
C	-3.67983538	-30.56318516	0.00000000
C	-3.67395198	-29.10816019	0.00000000
C	-3.67394033	29.10812893	0.00000000
C	-3.67982264	30.56315644	0.00000000
C	-2.45380397	-31.24092103	0.00000000
C	-2.44623433	-28.43707659	0.00000000
C	-2.44622416	28.43704327	0.00000000
C	-2.45379210	31.24089295	0.00000000
C	-1.22660592	-30.56974657	0.00000000
C	-1.22464781	-29.11481597	0.00000000
C	-1.22463803	29.11478332	0.00000000
C	-1.22659548	30.56971644	0.00000000
C	0.00000279	-31.24414101	0.00000000
C	0.00000281	-28.44045923	0.00000000
C	0.00001150	28.44042489	0.00000000
C	0.00001225	31.24411182	0.00000000
C	1.22661133	-30.56974653	0.00000000
C	1.22465323	-29.11481604	0.00000000
C	1.22466120	29.11478274	0.00000000
C	1.22661941	30.56971568	0.00000000
C	2.45380891	-31.24092092	0.00000000
C	2.44623928	-28.43707674	0.00000000
C	2.44624640	28.43704222	0.00000000
C	2.45381582	31.24089134	0.00000000
C	3.67983953	-30.56318504	0.00000000
C	3.67395614	-29.10816037	0.00000000
C	3.67396202	29.10812720	0.00000000
C	3.67984509	30.56315419	0.00000000
C	4.90753901	-31.23121362	0.00000000
C	4.89236333	-28.42694742	0.00000000
C	4.89236839	28.42691355	0.00000000
C	4.90754305	31.23118431	0.00000000
C	6.13295401	-30.54987952	0.00000000
C	6.12311906	-29.09497435	0.00000000
C	6.12312235	29.09494216	0.00000000
C	6.13295664	30.54984937	0.00000000
C	7.36135537	-31.21485321	0.00000000
C	7.33850050	-28.41015456	0.00000000
C	7.33850279	28.41012220	0.00000000
C	7.36135579	31.21482468	0.00000000
C	8.58543141	-30.52950352	0.00000000
C	8.57160313	-29.07549406	0.00000000
C	8.57160312	29.07546378	0.00000000
C	8.58543026	30.52947478	0.00000000
C	9.81582713	-31.19148205	0.00000000
C	9.78516336	-28.38688853	0.00000000
C	9.78516186	28.38685865	0.00000000
C	9.81582289	31.19145485	0.00000000
C	11.03632884	-30.50178008	0.00000000
C	11.01847827	-29.04975099	0.00000000
C	11.01847388	29.04972331	0.00000000
C	11.03632276	30.50175321	0.00000000
C	12.27218577	-31.16058105	0.00000000
C	12.23323038	-28.35658681	0.00000000
C	12.23322386	28.35656011	0.00000000
C	12.27217605	31.16055588	0.00000000
C	13.48234495	-30.46323268	0.00000000
C	13.46169460	-29.01807670	0.00000000
C	13.46168438	29.01805189	0.00000000
C	13.48233330	30.46320809	0.00000000
C	14.73455167	-31.10428651	0.00000000
C	14.68226935	-28.31793973	0.00000000
C	14.68225700	28.31791630	0.00000000
C	14.73453681	31.10426378	0.00000000
C	15.92778990	-30.39889515	0.00000000
C	15.90978821	-28.95553516	0.00000000
C	15.90977188	28.95551356	0.00000000
C	15.92777275	30.39887313	0.00000000
C	17.18175259	-31.08124164	0.00000000
C	17.16849478	-28.23330322	0.00000000
C	17.23751530	-26.85168624	0.00000000
C	17.23749633	26.85166985	0.00000000
C	17.16847718	28.23328358	0.00000000
C	17.18173417	31.08122001	0.00000000
C	18.36117369	-30.40453590	0.00000000

C	18.40517066	-28.97756967	0.00000000
C	18.45814474	-26.15159480	0.00000000
C	18.50726825	-24.75721484	0.00000000
C	18.50724618	24.75720595	0.00000000
C	18.45812405	26.15158191	0.00000000
C	18.40515160	28.97755148	0.00000000
C	18.36115310	30.40451679	0.00000000
C	19.61647060	-28.30363554	0.00000000
C	19.69505005	-26.89904871	0.00000000
C	19.71884645	-24.05857358	0.00000000
C	19.76894600	-22.65935173	0.00000000
C	19.76891925	22.65935109	0.00000000
C	19.71882184	24.05856884	0.00000000
C	19.69502849	26.89903618	0.00000000
C	19.61645045	28.30362079	0.00000000
C	20.90795955	-26.20636592	0.00000000
C	20.96315885	-24.80689986	0.00000000
C	20.97584801	-21.95927965	0.00000000
C	21.02322787	-20.55901703	0.00000000
C	21.02319644	20.55902432	0.00000000
C	20.97581871	21.95928326	0.00000000
C	20.96313346	24.80689432	0.00000000
C	20.90793641	26.20635677	0.00000000
C	22.17461218	-24.10121239	0.00000000
C	22.22409934	-22.70478852	0.00000000
C	22.22751486	-19.85509954	0.00000000
C	22.27072178	-18.45552189	0.00000000
C	22.27068749	18.45553542	0.00000000
C	22.22748165	19.85511053	0.00000000
C	22.22406903	22.70479132	0.00000000
C	22.17458419	24.10121066	0.00000000
C	23.43338559	-21.99336931	0.00000000
C	23.47885316	-20.59708758	0.00000000
C	23.47305535	-17.74664325	0.00000000
C	23.51126051	-16.34815388	0.00000000
C	23.51122613	16.34817132	0.00000000
C	23.47302052	17.74665947	0.00000000
C	23.47881883	20.59709853	0.00000000
C	23.43335257	21.99337614	0.00000000
C	24.68502922	-19.88187752	0.00000000
C	24.72688126	-18.48459273	0.00000000
C	24.71174566	-15.63410431	0.00000000
C	24.74461362	-14.23661356	0.00000000
C	24.74458152	14.23663337	0.00000000
C	24.71171180	15.63412365	0.00000000
C	24.72684535	18.48460982	0.00000000
C	24.68499314	19.88189201	0.00000000
C	25.92985015	-17.76629249	0.00000000
C	25.96793958	-16.36779056	0.00000000
C	25.94319729	-13.51741389	0.00000000
C	25.97052600	-12.12086331	0.00000000
C	25.97049750	12.12088538	0.00000000
C	25.94316622	13.51743546	0.00000000
C	25.96790454	16.36781136	0.00000000
C	25.92981374	17.76631220	0.00000000
C	27.16782500	-15.64645068	0.00000000
C	27.20189986	-14.24677586	0.00000000
C	27.16724261	-11.39646375	0.00000000
C	27.18881431	-10.00090810	0.00000000
C	27.18878986	10.00093326	0.00000000
C	27.16721514	11.39648802	0.00000000
C	27.20186717	14.24679908	0.00000000
C	27.16779024	15.64647349	0.00000000
C	28.39874584	-13.52236001	0.00000000
C	28.42858819	-12.12144910	0.00000000
C	28.38378991	-9.27136104	0.00000000
C	28.39951410	-7.87660534	0.00000000
C	28.39949388	7.87663459	0.00000000
C	28.38376632	9.27138911	0.00000000
C	28.42855838	12.12147503	0.00000000
C	28.39871354	13.52238530	0.00000000
C	29.62236139	-11.39400840	0.00000000
C	29.64763847	-9.99177480	0.00000000
C	29.59269114	-7.14264022	0.00000000
C	29.60299680	-5.74755806	0.00000000
C	29.60298114	5.74759208	0.00000000
C	29.59267173	7.14267306	0.00000000
C	29.64761181	9.99180469	0.00000000
C	29.62233178	11.39403701	0.00000000
C	30.83847937	-9.26114812	0.00000000
C	30.85840536	-7.85802268	0.00000000
C	30.79347948	-5.01110488	0.00000000
C	30.79923869	-3.61270829	0.00000000

C	30.79922813	3.61274732	0.00000000
C	30.79346483	5.01114299	0.00000000
C	30.85838242	7.85805778	0.00000000
C	30.83845287	9.26118146	0.00000000
C	32.04699373	-7.12311426	0.00000000
C	32.06013520	-5.72088582	0.00000000
C	31.98547372	-2.87871298	0.00000000
C	31.98672083	-1.47178196	0.00000000
C	31.98671589	1.47182553	0.00000000
C	31.98546448	2.87875611	0.00000000
C	32.06011698	5.72092682	0.00000000
C	32.04697133	7.12315347	0.00000000
C	33.24805583	-4.97862693	0.00000000
C	33.24819273	-3.58154751	0.00000000
C	33.15111930	-0.72552731	0.00000000
C	33.15111664	0.72557515	0.00000000
C	33.24818038	3.58159419	0.00000000
C	33.24803882	4.97867228	0.00000000
C	34.42814802	-2.81512282	0.00000000
C	34.41131859	-1.42878374	0.00000000
C	34.41131361	1.42883541	0.00000000
C	34.42813778	2.81517393	0.00000000
C	35.62692528	-0.67999386	0.00000000
C	35.62692274	0.68004975	0.00000000
H	-36.57287028	-1.23898534	0.00000000
H	-36.57286662	1.23901518	0.00000000
H	-35.39776236	-3.33508859	0.00000000
H	-35.39775212	3.33511451	0.00000000
H	-34.20958234	-5.51312889	0.00000000
H	-34.20956607	5.51314985	0.00000000
H	-33.00573805	-7.66264921	0.00000000
H	-33.00571670	7.66266579	0.00000000
H	-31.79517722	-9.80436172	0.00000000
H	-31.79515187	9.80437486	0.00000000
H	-31.00828459	-0.97161543	0.00000000
H	-31.00828242	0.97162895	0.00000000
H	-30.57706932	-11.94076352	0.00000000
H	-30.57704075	11.94077499	0.00000000
H	-29.83857103	-3.07645456	0.00000000
H	-29.83856389	3.07646552	0.00000000
H	-29.35152295	-14.07250780	0.00000000
H	-29.35149131	14.07251934	0.00000000
H	-28.64452869	-5.20761239	0.00000000
H	-28.64451652	5.20762039	0.00000000
H	-28.11871477	-16.19986532	0.00000000
H	-28.11867993	16.19987781	0.00000000
H	-27.44294724	-7.33326470	0.00000000
H	-27.44292976	7.33327054	0.00000000
H	-26.87887605	-18.32288387	0.00000000
H	-26.87883832	18.32289664	0.00000000
H	-26.23413255	-9.45421377	0.00000000
H	-26.23410943	9.45421848	0.00000000
H	-25.63219443	-20.44159265	0.00000000
H	-25.63215531	20.44160355	0.00000000
H	-25.01775819	-11.57080523	0.00000000
H	-25.01772918	11.57080998	0.00000000
H	-24.37868149	-22.55618294	0.00000000
H	-24.37864340	22.55618960	0.00000000
H	-23.79376225	-13.68323671	0.00000000
H	-23.79372761	13.68324248	0.00000000
H	-23.11803995	-24.66709284	0.00000000
H	-23.11800486	24.66709438	0.00000000
H	-22.56230051	-15.79155059	0.00000000
H	-22.56226155	15.79155753	0.00000000
H	-21.84879747	-26.77648593	0.00000000
H	-21.84876556	26.77648300	0.00000000
H	-21.32359972	-17.89582928	0.00000000
H	-21.32355885	17.89583619	0.00000000
H	-20.54951955	-28.88670006	0.00000000
H	-20.54948877	28.88669451	0.00000000
H	-20.07785679	-19.99641646	0.00000000
H	-20.07781682	19.99642111	0.00000000
H	-19.31697790	-30.94645166	0.00000000
H	-19.31694431	30.94644445	0.00000000
H	-18.82526385	-22.09395216	0.00000000
H	-18.82522680	22.09395244	0.00000000
H	-17.56532895	-24.18872314	0.00000000
H	-17.56529499	24.18871807	0.00000000
H	-17.16712751	-32.17987118	0.00000000
H	-17.16709597	32.17985952	0.00000000
H	-16.31722062	-26.25114392	0.00000000
H	-16.31718773	26.25113431	0.00000000
H	-14.76695607	-32.20403242	0.00000000

H	-14.62841529	-27.22039598	0.00000000
H	-14.62839133	27.22037750	0.00000000
H	-14.76692518	32.20401932	0.00000000
H	-12.28933090	-32.26051937	0.00000000
H	-12.21801557	-27.25650718	0.00000000
H	-12.21799507	27.25648395	0.00000000
H	-12.28930263	32.26050314	0.00000000
H	-9.82815456	-32.29151110	0.00000000
H	-9.77337474	-27.28686799	0.00000000
H	-9.77335803	27.28683996	0.00000000
H	-9.82813080	32.29149209	0.00000000
H	-7.37042896	-32.31494183	0.00000000
H	-7.32963647	-27.31009170	0.00000000
H	-7.32962299	27.31005966	0.00000000
H	-7.37040948	32.31492067	0.00000000
H	-4.91348870	-32.33134909	0.00000000
H	-4.88639405	-27.32684204	0.00000000
H	-4.88638290	27.32680712	0.00000000
H	-4.91347305	32.33132604	0.00000000
H	-2.45674904	-32.34108616	0.00000000
H	-2.44323448	-27.33694253	0.00000000
H	-2.44322496	27.33690572	0.00000000
H	-2.45673657	32.34106147	0.00000000
H	0.00000279	-32.34431616	0.00000000
H	0.00000281	-27.34031526	0.00000000
H	0.00001110	27.34027745	0.00000000
H	0.00001246	32.34429031	0.00000000
H	2.45675399	-32.34108598	0.00000000
H	2.44323945	-27.33694276	0.00000000
H	2.44324643	27.33690488	0.00000000
H	2.45676073	32.34105966	0.00000000
H	4.91349183	-32.33134876	0.00000000
H	4.88639716	-27.32684244	0.00000000
H	4.88640227	27.32680539	0.00000000
H	4.91349507	32.33132254	0.00000000
H	7.37042959	-32.31494141	0.00000000
H	7.32963701	-27.31009217	0.00000000
H	7.32963938	27.31005692	0.00000000
H	7.37042844	32.31491566	0.00000000
H	9.82815259	-32.29151064	0.00000000
H	9.77337260	-27.28686844	0.00000000
H	9.77337111	27.28683612	0.00000000
H	9.82814637	32.29148577	0.00000000
H	12.28932674	-32.26051887	0.00000000
H	12.21801116	-27.25650752	0.00000000
H	12.21800501	27.25647897	0.00000000
H	12.28931505	32.26049561	0.00000000
H	14.76695029	-32.20403179	0.00000000
H	14.62840921	-27.22039613	0.00000000
H	14.62839859	27.22037126	0.00000000
H	14.76693473	32.20401045	0.00000000
H	16.31721336	-26.25114459	0.00000000
H	16.31719268	26.25112903	0.00000000
H	17.16712048	-32.17987069	0.00000000
H	17.16710254	32.17985013	0.00000000
H	17.56532154	-24.18872425	0.00000000
H	17.56529891	24.18871529	0.00000000
H	18.82525626	-22.09395346	0.00000000
H	18.82522922	22.09395259	0.00000000
H	19.31697032	-30.94645162	0.00000000
H	19.31694910	30.94643535	0.00000000
H	20.07784913	-19.99641754	0.00000000
H	20.07781754	19.99642442	0.00000000
H	20.54951176	-28.88670060	0.00000000
H	20.54949136	28.88668756	0.00000000
H	21.32359208	-17.89582966	0.00000000
H	21.32355777	17.89584260	0.00000000
H	21.84878961	-26.77648689	0.00000000
H	21.84876655	26.77647855	0.00000000
H	22.56229305	-15.79154978	0.00000000
H	22.56225879	15.79156665	0.00000000
H	23.11803210	-24.66709396	0.00000000
H	23.11800402	24.66709264	0.00000000
H	23.79375515	-13.68323427	0.00000000
H	23.79372348	13.68325368	0.00000000
H	24.37867386	-22.55618395	0.00000000
H	24.37864061	22.55619104	0.00000000
H	25.01775165	-11.57080085	0.00000000
H	25.01772415	11.57082253	0.00000000
H	25.63218735	-20.44159323	0.00000000
H	25.63215079	20.44160833	0.00000000
H	26.23412674	-9.45420731	0.00000000
H	26.23410404	9.45423171	0.00000000

H	26.87886984	-18.32288362	0.00000000
H	26.87883254	18.32290460	0.00000000
H	27.44294230	-7.33325621	0.00000000
H	27.44292452	7.33328395	0.00000000
H	28.11870969	-16.19986382	0.00000000
H	28.11867346	16.19988847	0.00000000
H	28.64452465	-5.20760211	0.00000000
H	28.64451186	5.20763367	0.00000000
H	29.35151911	-14.07250467	0.00000000
H	29.35148471	14.07253204	0.00000000
H	29.83856785	-3.07644276	0.00000000
H	29.83856013	3.07647853	0.00000000
H	30.57706668	-11.94075846	0.00000000
H	30.57703450	11.94078903	0.00000000
H	31.00828223	-0.97160251	0.00000000
H	31.00827984	0.97164157	0.00000000
H	31.79517557	-9.80435459	0.00000000
H	31.79514632	9.80438964	0.00000000
H	33.00573710	-7.66264008	0.00000000
H	33.00571205	7.66268089	0.00000000
H	34.20958175	-5.51311808	0.00000000
H	34.20956246	5.51316513	0.00000000
H	35.39776216	-3.33507627	0.00000000
H	35.39774986	3.33512976	0.00000000
H	36.57287001	-1.23897119	0.00000000
H	36.57286559	1.23902970	0.00000000

Cartesian coordinates (in Å) for the lowest triplet states of n-HGQRs (n = 3–15), calculated by spin-unrestricted TAO-LDA.

```
3-HGQR (triplet)
  C     -6.14982306     -0.68663278      0.00000000
  C     -6.14982308      0.68663274      0.00000000
  C     -4.90915205     -2.83459886      0.00000000
  C     -4.93905696     -1.43288232      0.00000000
  C     -4.93905703      1.43288239      0.00000000
  C     -4.90915218      2.83459893      0.00000000
  C     -3.66975112     -4.98350923      0.00000000
  C     -3.71052289     -3.56172195      0.00000000
  C     -3.69813986     -0.72017989      0.00000000
  C     -3.69813989      0.72018007      0.00000000
  C     -3.71052301      3.56172203      0.00000000
  C     -3.66975124      4.98350938      0.00000000
  C     -2.48057993     -5.67023351      0.00000000
  C     -2.47285770     -2.84346777      0.00000000
  C     -2.51470237     -1.45238588      0.00000000
  C     -2.51470249      1.45238606      0.00000000
  C     -2.47285782      2.84346785      0.00000000
  C     -2.48057998      5.67023365      0.00000000
  C     -1.22889468     -4.99484708      0.00000000
  C     -1.22563148     -3.56369226      0.00000000
  C     -1.22563164      3.56369229      0.00000000
  C     -1.22889472      4.99484713      0.00000000
  C      0.00000001     -5.66959022      0.00000000
  C      0.00000008     -2.90457827      0.00000000
  C     -0.00000011      2.90457829      0.00000000
  C      0.00000001      5.66959026      0.00000000
  C      1.22889472     -4.99484708      0.00000000
  C      1.22563164     -3.56369225      0.00000000
  C      1.22563148      3.56369225      0.00000000
  C      1.22889469      4.99484707      0.00000000
  C      2.48057997     -5.67023361      0.00000000
  C      2.47285783     -2.84346782      0.00000000
  C      2.51470250     -1.45238603      0.00000000
  C      2.51470239      1.45238588      0.00000000
  C      2.47285770      2.84346776      0.00000000
  C      2.48057993      5.67023347      0.00000000
  C      3.66975123     -4.98350935      0.00000000
  C      3.71052300     -3.56172201      0.00000000
  C      3.69813991     -0.72018007      0.00000000
  C      3.69813988      0.72017989      0.00000000
  C      3.71052289      3.56172193      0.00000000
  C      3.66975113      4.98350921      0.00000000
  C      4.90915218     -2.83459893      0.00000000
  C      4.93905703     -1.43288241      0.00000000
  C      4.93905697      1.43288231      0.00000000
  C      4.90915205      2.83459883      0.00000000
  C      6.14982309     -0.68663278      0.00000000
  C      6.14982305      0.68663273      0.00000000
  H     -7.09975392     -1.23864117      0.00000000
  H     -7.09975401      1.23864108      0.00000000
  H     -5.86205336     -3.38464067      0.00000000
  H     -5.86205351      3.38464073      0.00000000
  H     -4.62276553     -5.53019395      0.00000000
  H     -4.62276566      5.53019415      0.00000000
  H     -2.47748406     -6.76889749      0.00000000
  H     -2.47748410      6.76889765      0.00000000
  H     -1.56673530     -0.90567733      0.00000000
  H     -1.56673543      0.90567751      0.00000000
  H      0.00000003     -6.76983452      0.00000000
  H      0.00000007     -1.81027039      0.00000000
  H     -0.00000014      1.81027041      0.00000000
  H      0.00000002      6.76983455      0.00000000
  H      1.56673544     -0.90567747      0.00000000
  H      1.56673533      0.90567732      0.00000000
  H      2.47748407     -6.76889761      0.00000000
  H      2.47748408      6.76889745      0.00000000
  H      4.62276564     -5.53019413      0.00000000
  H      4.62276554      5.53019393      0.00000000
  H      5.86205351     -3.38464074      0.00000000
  H      5.86205334      3.38464065      0.00000000
  H      7.09975400     -1.23864110      0.00000000
  H      7.09975393      1.23864111      0.00000000

4-HGQR (triplet)
  C     -8.59866514     -0.68298084      0.00000000
  C     -8.59866502      0.68298185      0.00000000
  C     -7.38612152     -2.82945633      0.00000000
  C     -7.38613360     -1.43387689      0.00000000
  C     -7.38613336      1.43387768      0.00000000
```

C	-7.38612115	2.82945705	0.00000000
C	-6.14332343	-4.98237197	0.00000000
C	-6.19595715	-3.57754371	0.00000000
C	-6.13710463	-0.72274582	0.00000000
C	-6.13710453	0.72274644	0.00000000
C	-6.19595681	3.57754423	0.00000000
C	-6.14332286	4.98237231	0.00000000
C	-4.89074652	-7.10558005	0.00000000
C	-4.93468765	-5.67998096	0.00000000
C	-4.95166390	-2.85907458	0.00000000
C	-4.96241364	-1.45895401	0.00000000
C	-4.96241351	1.45895448	0.00000000
C	-4.95166373	2.85907498	0.00000000
C	-4.93468711	5.67998099	0.00000000
C	-4.89074590	7.10557988	0.00000000
C	-3.70792793	-7.78866469	0.00000000
C	-3.69434172	-4.95372022	0.00000000
C	-3.74441569	-3.56817408	0.00000000
C	-3.74441550	3.56817419	0.00000000
C	-3.69434140	4.95372018	0.00000000
C	-3.70792737	7.78866430	0.00000000
C	-2.45139211	-7.11401920	0.00000000
C	-2.44269801	-5.67658548	0.00000000
C	-2.44269775	5.67658520	0.00000000
C	-2.45139181	7.11401876	0.00000000
C	-1.24291558	-7.81190652	0.00000000
C	-1.21778702	-5.02739645	0.00000000
C	-1.21778702	5.02739610	0.00000000
C	-1.24291545	7.81190593	0.00000000
C	-0.00000003	-7.15521051	0.00000000
C	0.00000002	-5.71821612	0.00000000
C	-0.00000014	5.71821568	0.00000000
C	-0.00000016	7.15520991	0.00000000
C	1.24291545	-7.81190665	0.00000000
C	1.21778712	-5.02739654	0.00000000
C	1.21778676	5.02739611	0.00000000
C	1.24291516	7.81190592	0.00000000
C	2.45139204	-7.11401942	0.00000000
C	2.44269806	-5.67658568	0.00000000
C	2.44269753	5.67658520	0.00000000
C	2.45139156	7.11401878	0.00000000
C	3.70792785	-7.78866495	0.00000000
C	3.69434182	-4.95372050	0.00000000
C	3.74441588	-3.56817433	0.00000000
C	3.74441534	3.56817413	0.00000000
C	3.69434121	4.95372017	0.00000000
C	3.70792713	7.78866440	0.00000000
C	4.89074650	-7.10558037	0.00000000
C	4.93468775	-5.67998128	0.00000000
C	4.95166416	-2.85907488	0.00000000
C	4.96241383	-1.45895425	0.00000000
C	4.96241354	1.45895434	0.00000000
C	4.95166360	2.85907488	0.00000000
C	4.93468694	5.67998103	0.00000000
C	4.89074570	7.10557999	0.00000000
C	6.14332361	-4.98237235	0.00000000
C	6.19595744	-3.57754407	0.00000000
C	6.13710481	-0.72274596	0.00000000
C	6.13710467	0.72274635	0.00000000
C	6.19595670	3.57754417	0.00000000
C	6.14332275	4.98237232	0.00000000
C	7.38612179	-2.82945660	0.00000000
C	7.38613380	-1.43387709	0.00000000
C	7.38613353	1.43387764	0.00000000
C	7.38612118	2.82945705	0.00000000
C	8.59866537	-0.68298097	0.00000000
C	8.59866523	0.68298177	0.00000000
H	-9.54729826	-1.23736424	0.00000000
H	-9.54729804	1.23736537	0.00000000
H	-8.34992773	-3.35993023	0.00000000
H	-8.34992740	3.35993075	0.00000000
H	-7.08449508	-5.55209214	0.00000000
H	-7.08449435	5.55209262	0.00000000
H	-5.84520779	-7.64986444	0.00000000
H	-5.84520703	7.64986435	0.00000000
H	-3.98812979	-0.95213024	0.00000000
H	-3.98812973	0.95213060	0.00000000
H	-3.70212902	-8.88738533	0.00000000
H	-3.70212841	8.88738484	0.00000000
H	-2.81835423	-2.97774407	0.00000000
H	-2.81835422	2.97774402	0.00000000
H	-1.26559938	-8.91181611	0.00000000
H	-1.16978014	-3.93024373	0.00000000

H	-1.16978023	3.93024345	0.00000000
H	-1.26559922	8.91181541	0.00000000
H	1.26559921	-8.91181624	0.00000000
H	1.16978033	-3.93024380	0.00000000
H	1.16978000	3.93024346	0.00000000
H	1.26559890	8.91181540	0.00000000
H	2.81835446	-2.97774421	0.00000000
H	2.81835404	2.97774397	0.00000000
H	3.70212895	-8.88738560	0.00000000
H	3.70212809	8.88738496	0.00000000
H	3.98812993	-0.95213053	0.00000000
H	3.98812981	0.95213035	0.00000000
H	5.84520773	-7.64986487	0.00000000
H	5.84520686	7.64986444	0.00000000
H	7.08449523	-5.55209263	0.00000000
H	7.08449428	5.55209261	0.00000000
H	8.34992818	-3.35993023	0.00000000
H	8.34992729	3.35993109	0.00000000
H	9.54729853	-1.23736437	0.00000000
H	9.54729828	1.23736531	0.00000000

5-HGQR (triplet)

C	-11.04918206	-0.68123796	0.00000000
C	-11.04918183	0.68124209	0.00000000
C	-9.84194142	-2.82392429	0.00000000
C	-9.83495372	-1.43351249	0.00000000
C	-9.83495343	1.43351628	0.00000000
C	-9.84194092	2.82392756	0.00000000
C	-8.63250445	-4.98413267	0.00000000
C	-8.65554199	-3.58296191	0.00000000
C	-8.58151756	-0.72449356	0.00000000
C	-8.58151749	0.72449737	0.00000000
C	-8.65554187	3.58296465	0.00000000
C	-8.63250443	4.98413460	0.00000000
C	-7.36611072	-7.11115910	0.00000000
C	-7.43035168	-5.70417246	0.00000000
C	-7.40262791	-2.86650220	0.00000000
C	-7.41194139	-1.46234007	0.00000000
C	-7.41194145	1.46234347	0.00000000
C	-7.40262826	2.86650515	0.00000000
C	-7.43035225	5.70417372	0.00000000
C	-7.36611141	7.11115953	0.00000000
C	-6.11403953	-9.22766459	0.00000000
C	-6.15836087	-7.79993419	0.00000000
C	-6.18360747	-4.97694444	0.00000000
C	-6.20565733	-3.58234811	0.00000000
C	-6.20565817	3.58235052	0.00000000
C	-6.18360862	4.97694610	0.00000000
C	-6.15836228	7.79993419	0.00000000
C	-6.11404134	9.22766399	0.00000000
C	-4.93420596	-9.90892385	0.00000000
C	-4.91772340	-7.06861376	0.00000000
C	-4.97201395	-5.68667509	0.00000000
C	-4.97201559	5.68667606	0.00000000
C	-4.91772540	7.06861398	0.00000000
C	-4.93420834	9.90892318	0.00000000
C	-3.67571165	-9.23337488	0.00000000
C	-3.66298874	-7.79316255	0.00000000
C	-3.66299129	7.79316258	0.00000000
C	-3.67571449	9.23337441	0.00000000
C	-2.47525601	-9.93478258	0.00000000
C	-2.43914027	-7.14932694	0.00000000
C	-2.43914326	7.14932722	0.00000000
C	-2.47525937	9.93478218	0.00000000
C	-1.22471004	-9.28693335	0.00000000
C	-1.21856977	-7.84346837	0.00000000
C	-1.21857320	7.84346865	0.00000000
C	-1.22471357	9.28693334	0.00000000
C	0.00000264	-9.96782498	0.00000000
C	0.00000217	-7.16499915	0.00000000
C	-0.00000138	7.16499955	0.00000000
C	-0.00000116	9.96782514	0.00000000
C	1.22471494	-9.28693275	0.00000000
C	1.21857422	-7.84346797	0.00000000
C	1.21857062	7.84346840	0.00000000
C	1.22471124	9.28693323	0.00000000
C	2.47526094	-9.93478126	0.00000000
C	2.43914411	-7.14932627	0.00000000
C	2.43914077	7.14932666	0.00000000
C	2.47525727	9.93478201	0.00000000
C	3.67571581	-9.23337308	0.00000000
C	3.66299220	-7.79316134	0.00000000
C	3.66298929	7.79316185	0.00000000
C	3.67571257	9.23337404	0.00000000

C	4.93420970	-9.90892150	0.00000000
C	4.91772595	-7.06861251	0.00000000
C	4.97201563	-5.68667490	0.00000000
C	4.97201407	5.68667427	0.00000000
C	4.91772372	7.06861281	0.00000000
C	4.93420689	9.90892272	0.00000000
C	6.11404232	-9.22766201	0.00000000
C	6.15836273	-7.79993241	0.00000000
C	6.18360836	-4.97694505	0.00000000
C	6.20565758	-3.58234989	0.00000000
C	6.20565697	3.58234733	0.00000000
C	6.18360734	4.97694351	0.00000000
C	6.15836121	7.79993299	0.00000000
C	6.11404020	9.22766327	0.00000000
C	7.36611137	-7.11115774	0.00000000
C	7.43035181	-5.70417234	0.00000000
C	7.40262734	-2.86650479	0.00000000
C	7.41194030	-1.46234351	0.00000000
C	7.41194044	1.46233939	0.00000000
C	7.40262725	2.86650130	0.00000000
C	7.43035154	5.70417123	0.00000000
C	7.36611083	7.11115776	0.00000000
C	8.63250354	-4.98413347	0.00000000
C	8.65554066	-3.58296403	0.00000000
C	8.58151608	-0.72449776	0.00000000
C	8.58151628	0.72449290	0.00000000
C	8.65554128	3.58296073	0.00000000
C	8.63250407	4.98413134	0.00000000
C	9.84193932	-2.82392724	0.00000000
C	9.83495178	-1.43351645	0.00000000
C	9.83495237	1.43351145	0.00000000
C	9.84194039	2.82392299	0.00000000
C	11.04918006	-0.68124270	0.00000000
C	11.04918046	0.68123703	0.00000000
H	-11.99698028	-1.23710314	0.00000000
H	-11.99697992	1.23710723	0.00000000
H	-10.80828945	-3.34987642	0.00000000
H	-10.80828872	3.34987946	0.00000000
H	-9.58493978	-5.53453504	0.00000000
H	-9.58493941	5.53453674	0.00000000
H	-8.30459919	-7.68539542	0.00000000
H	-8.30459951	7.68539567	0.00000000
H	-7.06935274	-9.77049929	0.00000000
H	-7.06935432	9.77049846	0.00000000
H	-6.43603344	-0.95827768	0.00000000
H	-6.43603373	0.95828077	0.00000000
H	-5.25302134	-3.03188146	0.00000000
H	-5.25302247	3.03188392	0.00000000
H	-4.92660609	-11.00765700	0.00000000
H	-4.92660849	11.00765611	0.00000000
H	-4.04763335	-5.09332673	0.00000000
H	-4.04763522	5.09332796	0.00000000
H	-2.50315363	-11.03463062	0.00000000
H	-2.38770235	-6.05215890	0.00000000
H	-2.38770503	6.05215945	0.00000000
H	-2.50315708	11.03463005	0.00000000
H	0.00000281	-11.06783824	0.00000000
H	0.00000184	-6.06479855	0.00000000
H	-0.00000141	6.06479899	0.00000000
H	-0.00000112	11.06783831	0.00000000
H	2.50315890	-11.03462916	0.00000000
H	2.38770554	-6.05215855	0.00000000
H	2.38770249	6.05215874	0.00000000
H	2.50315523	11.03462993	0.00000000
H	4.04763512	-5.09332717	0.00000000
H	4.04763350	5.09332612	0.00000000
H	4.92661005	-11.00765441	0.00000000
H	4.92660728	11.00765577	0.00000000
H	5.25302198	-3.03188351	0.00000000
H	5.25302100	3.03188095	0.00000000
H	6.43603278	-0.95828085	0.00000000
H	6.43603255	0.95827727	0.00000000
H	7.06935533	-9.77049616	0.00000000
H	7.06935346	9.77049773	0.00000000
H	8.30459937	-7.68539365	0.00000000
H	8.30459931	7.68539386	0.00000000
H	9.58493836	-5.53453539	0.00000000
H	9.58493940	5.53453348	0.00000000
H	10.80828693	-3.34987900	0.00000000
H	10.80828840	3.34987489	0.00000000
H	11.99697791	-1.23710786	0.00000000
H	11.99697858	1.23710210	0.00000000

6-HGQR (triplet)

C	-13.49915371	-0.68054052	0.00000000
C	-13.49915430	0.68053414	0.00000000
C	-12.29551731	-2.82075520	0.00000000
C	-12.28454162	-1.43326805	0.00000000
C	-12.28454278	1.43326271	0.00000000
C	-12.29551957	2.82075012	0.00000000
C	-11.09572552	-4.98172126	0.00000000
C	-11.11051380	-3.58412449	0.00000000
C	-11.02777254	-0.72534202	0.00000000
C	-11.02777311	0.72533753	0.00000000
C	-11.11051643	3.58412057	0.00000000
C	-11.09572902	4.98171784	0.00000000
C	-9.86183726	-7.11728529	0.00000000
C	-9.89764688	-5.71371231	0.00000000
C	-9.85362122	-2.87099411	0.00000000
C	-9.86123121	-1.46461697	0.00000000
C	-9.86123223	1.46461352	0.00000000
C	-9.85362310	2.87099090	0.00000000
C	-9.89765054	5.71371015	0.00000000
C	-9.86184169	7.11728377	0.00000000
C	-8.58954588	-9.23592878	0.00000000
C	-8.65847811	-7.82803515	0.00000000
C	-8.64167618	-4.98805232	0.00000000
C	-8.66133626	-3.58983949	0.00000000
C	-8.66133833	3.58983741	0.00000000
C	-8.64167902	4.98805073	0.00000000
C	-8.65848257	7.82803496	0.00000000
C	-8.58955107	9.23592931	0.00000000
C	-7.33759536	-11.34781631	0.00000000
C	-7.38221787	-9.91954802	0.00000000
C	-7.41281000	-7.09539273	0.00000000
C	-7.44017796	-5.70334616	0.00000000
C	-7.44018085	5.70334574	0.00000000
C	-7.41281358	7.09539296	0.00000000
C	-7.38222289	9.91954972	0.00000000
C	-7.33760062	11.34781867	0.00000000
C	-6.15892190	-12.02823624	0.00000000
C	-6.14096749	-9.18456517	0.00000000
C	-6.19828939	-7.80453453	0.00000000
C	-6.19829294	7.80453596	0.00000000
C	-6.14097154	9.18456737	0.00000000
C	-6.15892683	12.02823944	0.00000000
C	-4.89995108	-11.35240499	0.00000000
C	-4.88467688	-9.90983485	0.00000000
C	-4.88468068	9.90983783	0.00000000
C	-4.89995499	11.35240863	0.00000000
C	-3.70403041	-12.05577873	0.00000000
C	-3.66108000	-9.26933825	0.00000000
C	-3.66108278	9.26934130	0.00000000
C	-3.70403356	12.05578296	0.00000000
C	-2.45046498	-11.41129613	0.00000000
C	-2.43941125	-9.96605775	0.00000000
C	-2.43941324	9.96606109	0.00000000
C	-2.45046714	11.41130025	0.00000000
C	-1.23308364	-12.09767152	0.00000000
C	-1.22048004	-9.29327728	0.00000000
C	-1.22048103	9.29328043	0.00000000
C	-1.23308475	12.09767591	0.00000000
C	-0.00000030	-11.42644439	0.00000000
C	-0.00000020	-9.97574044	0.00000000
C	-0.00000025	9.97574383	0.00000000
C	-0.00000032	11.42644857	0.00000000
C	1.23308297	-12.09767167	0.00000000
C	1.22047972	-9.29327746	0.00000000
C	1.22048060	9.29328057	0.00000000
C	1.23308404	12.09767602	0.00000000
C	2.45046441	-11.41129643	0.00000000
C	2.43941086	-9.96605806	0.00000000
C	2.43941277	9.96606136	0.00000000
C	2.45046653	11.41130050	0.00000000
C	3.70402981	-12.05577915	0.00000000
C	3.66107971	-9.26933872	0.00000000
C	3.66108238	9.26934170	0.00000000
C	3.70403293	12.05578329	0.00000000
C	4.89995061	-11.35240554	0.00000000
C	4.88467657	-9.90983539	0.00000000
C	4.88468031	9.90983824	0.00000000
C	4.89995447	11.35240906	0.00000000
C	6.15892137	-12.02823695	0.00000000
C	6.14096733	-9.18456579	0.00000000
C	6.19828947	-7.80453514	0.00000000
C	6.19829300	7.80453644	0.00000000
C	6.14097133	9.18456786	0.00000000

C	6.15892625	12.02824001	0.00000000
C	7.33759493	-11.34781713	0.00000000
C	7.38221764	-9.91954879	0.00000000
C	7.41281018	-7.09539338	0.00000000
C	7.44017833	-5.70334678	0.00000000
C	7.44018123	5.70334629	0.00000000
C	7.41281375	7.09539353	0.00000000
C	7.38222260	9.91955036	0.00000000
C	7.33760013	11.34781935	0.00000000
C	8.58954574	-9.23592960	0.00000000
C	8.65847822	-7.82803589	0.00000000
C	8.64167659	-4.98805289	0.00000000
C	8.66133683	-3.58984000	0.00000000
C	8.66133891	3.58983790	0.00000000
C	8.64167944	4.98805125	0.00000000
C	8.65848265	7.82803562	0.00000000
C	8.58955087	9.23593003	0.00000000
C	9.86183743	-7.11728600	0.00000000
C	9.89764724	-5.71371291	0.00000000
C	9.85362178	-2.87099451	0.00000000
C	9.86123181	-1.46461732	0.00000000
C	9.86123282	1.46461386	0.00000000
C	9.85362367	2.87099129	0.00000000
C	9.89765090	5.71371072	0.00000000
C	9.86184183	7.11728443	0.00000000
C	11.09572593	-4.98172172	0.00000000
C	11.11051434	-3.58412488	0.00000000
C	11.02777304	-0.72534211	0.00000000
C	11.02777361	0.72533764	0.00000000
C	11.11051695	3.58412094	0.00000000
C	11.09572942	4.98171828	0.00000000
C	12.29551782	-2.82075536	0.00000000
C	12.28454217	-1.43326813	0.00000000
C	12.28454332	1.43326281	0.00000000
C	12.29552007	2.82075030	0.00000000
C	13.49915430	-0.68054052	0.00000000
C	13.49915488	0.68053419	0.00000000
H	-14.44654259	-1.23711700	0.00000000
H	-14.44654365	1.23710998	0.00000000
H	-13.26303824	-3.34457574	0.00000000
H	-13.26304101	3.34457011	0.00000000
H	-12.05141847	-5.52650349	0.00000000
H	-12.05142256	5.52649961	0.00000000
H	-10.81090174	-7.67353317	0.00000000
H	-10.81090683	7.67353120	0.00000000
H	-9.52669275	-9.81237519	0.00000000
H	-9.52669859	9.81237535	0.00000000
H	-8.88433152	-0.96208725	0.00000000
H	-8.88433202	0.96208466	0.00000000
H	-8.29328267	-11.89000057	0.00000000
H	-8.29328838	11.89000286	0.00000000
H	-7.70626907	-3.04379682	0.00000000
H	-7.70627049	3.04379540	0.00000000
H	-6.49031278	-5.14833086	0.00000000
H	-6.49031495	5.14833093	0.00000000
H	-6.15034806	-13.12696696	0.00000000
H	-6.15035349	13.12697059	0.00000000
H	-5.27486393	-7.20937720	0.00000000
H	-5.27486674	7.20937890	0.00000000
H	-3.73432771	-13.15557777	0.00000000
H	-3.60777071	-8.17208148	0.00000000
H	-3.60777311	8.17208403	0.00000000
H	-3.73433095	13.15558251	0.00000000
H	-1.23970632	-13.19769200	0.00000000
H	-1.21522023	-8.19318287	0.00000000
H	-1.21522115	8.19318543	0.00000000
H	-1.23970739	13.19769697	0.00000000
H	1.23970550	-13.19769215	0.00000000
H	1.21522000	-8.19318305	0.00000000
H	1.21522080	8.19318559	0.00000000
H	1.23970656	13.19769709	0.00000000
H	3.73432696	-13.15557818	0.00000000
H	3.60777044	-8.17208199	0.00000000
H	3.60777269	8.17208447	0.00000000
H	3.73433020	13.15558283	0.00000000
H	5.27486409	-7.20937776	0.00000000
H	5.27486692	7.20937931	0.00000000
H	6.15034737	-13.12696767	0.00000000
H	6.15035276	13.12697115	0.00000000
H	6.49031320	-5.14833148	0.00000000
H	6.49031539	5.14833148	0.00000000
H	7.70626970	-3.04379734	0.00000000
H	7.70627115	3.04379589	0.00000000

```
  H      8.29328216    -11.89000151      0.00000000
  H      8.29328779     11.89000366      0.00000000
  H      8.88433213     -0.96208775      0.00000000
  H      8.88433264      0.96208516      0.00000000
  H      9.52669249     -9.81237614      0.00000000
  H      9.52669826      9.81237622      0.00000000
  H     10.81090181     -7.67353395      0.00000000
  H     10.81090686      7.67353195      0.00000000
  H     12.05141885     -5.52650391      0.00000000
  H     12.05142292      5.52650001      0.00000000
  H     13.26303876     -3.34457581      0.00000000
  H     13.26304151      3.34457021      0.00000000
  H     14.44654315     -1.23711700      0.00000000
  H     14.44654420      1.23711003      0.00000000

7-HGQR (triplet)
  C    -15.95006779     -0.68039520      0.00000000
  C    -15.95006832      0.68038863      0.00000000
  C    -14.74842494     -2.81899230      0.00000000
  C    -14.73551676     -1.43254159      0.00000000
  C    -14.73551787      1.43253594      0.00000000
  C    -14.74842695      2.81898681      0.00000000
  C    -13.55456381     -4.98044631      0.00000000
  C    -13.56468124     -3.58422959      0.00000000
  C    -13.47699018     -0.72543942      0.00000000
  C    -13.47699069      0.72543457      0.00000000
  C    -13.56468360      3.58422507      0.00000000
  C    -13.55456692      4.98044211      0.00000000
  C    -12.33058067     -7.11767638      0.00000000
  C    -12.35864429     -5.71690654      0.00000000
  C    -12.30536878     -2.87344169      0.00000000
  C    -12.31169903     -1.46634117      0.00000000
  C    -12.31169996      1.46633712      0.00000000
  C    -12.30537053      2.87343783      0.00000000
  C    -12.35864758      5.71690341      0.00000000
  C    -12.33058452      7.11767370      0.00000000
  C    -11.08965079     -9.24494981      0.00000000
  C    -11.13080263     -7.84105832      0.00000000
  C    -11.09857757     -4.99478635      0.00000000
  C    -11.11540685     -3.59518738      0.00000000
  C    -11.11540888      3.59518442      0.00000000
  C    -11.09858023      4.99478374      0.00000000
  C    -11.13080653      7.84105669      0.00000000
  C    -11.08965509      9.24494873      0.00000000
  C     -9.81346673    -11.35824889      0.00000000
  C     -9.88494288     -9.95055743      0.00000000
  C     -9.87610862     -7.10964055      0.00000000
  C     -9.90165814     -5.71361792      0.00000000
  C     -9.90166095      5.71361624      0.00000000
  C     -9.87611195      7.10963932      0.00000000
  C     -9.88494716      9.95055729      0.00000000
  C     -9.81347118     11.35824932      0.00000000
  C     -8.56163148    -13.46730655      0.00000000
  C     -8.60593627    -12.03939910      0.00000000
  C     -8.64029247     -9.21430474      0.00000000
  C     -8.67175338     -7.82281076      0.00000000
  C     -8.67175674      7.82281047      0.00000000
  C     -8.64029619      9.21430492      0.00000000
  C     -8.60594060     12.03940039      0.00000000
  C     -8.56163623     13.46730830      0.00000000
  C     -7.38312636    -14.14739610      0.00000000
  C     -7.36465971    -11.30221228      0.00000000
  C     -7.42433173     -9.92244721      0.00000000
  C     -7.42433539      9.92244827      0.00000000
  C     -7.36466346     11.30221375      0.00000000
  C     -7.38313097     14.14739844      0.00000000
  C     -6.12482495    -13.47109559      0.00000000
  C     -6.10808195    -12.02739979      0.00000000
  C     -6.10808524     12.02740203      0.00000000
  C     -6.12482880     13.47109825      0.00000000
  C     -4.93074078    -14.17553277      0.00000000
  C     -4.88371528    -11.38872330      0.00000000
  C     -4.88371782     11.38872605      0.00000000
  C     -4.93074419     14.17553580      0.00000000
  C     -3.67625608    -13.53299512      0.00000000
  C     -3.66198978    -12.08683625      0.00000000
  C     -3.66199170     12.08683940      0.00000000
  C     -3.67625838     13.53299862      0.00000000
  C     -2.46247212    -14.22277297      0.00000000
  C     -2.44166049    -11.41754766      0.00000000
  C     -2.44166154     11.41755117      0.00000000
  C     -2.46247381     14.22277671      0.00000000
  C     -1.22661199    -13.55568648      0.00000000
  C     -1.22139527    -12.10320515      0.00000000
```

C	-1.22139554	12.10320886	0.00000000
C	-1.22661251	13.55569054	0.00000000
C	0.00000660	-14.23241217	0.00000000
C	0.00000383	-11.42667738	0.00000000
C	0.00000447	11.42668130	0.00000000
C	0.00000696	14.23241631	0.00000000
C	1.22662374	-13.55568393	0.00000000
C	1.22140422	-12.10320273	0.00000000
C	1.22140563	12.10320667	0.00000000
C	1.22662512	13.55568823	0.00000000
C	2.46248500	-14.22276783	0.00000000
C	2.44166789	-11.41754294	0.00000000
C	2.44167017	11.41754689	0.00000000
C	2.46248743	14.22277204	0.00000000
C	3.67626717	-13.53298749	0.00000000
C	3.66199813	-12.08682901	0.00000000
C	3.66200115	12.08683281	0.00000000
C	3.67627035	13.53299164	0.00000000
C	4.93075264	-14.17552242	0.00000000
C	4.88372176	-11.38871387	0.00000000
C	4.88372545	11.38871744	0.00000000
C	4.93075686	14.17552631	0.00000000
C	6.12483472	-13.47108306	0.00000000
C	6.10808888	-12.02738792	0.00000000
C	6.10809321	12.02739109	0.00000000
C	6.12483951	13.47108666	0.00000000
C	7.38313644	-14.14738130	0.00000000
C	7.36466471	-11.30219871	0.00000000
C	7.42433430	-9.92243482	0.00000000
C	7.42433881	9.92243694	0.00000000
C	7.36466945	11.30220115	0.00000000
C	7.38314202	14.14738458	0.00000000
C	8.56163951	-13.46729004	0.00000000
C	8.60594132	-12.03938349	0.00000000
C	8.64029326	-9.21429207	0.00000000
C	8.67175212	-7.82279987	0.00000000
C	8.67175604	7.82280059	0.00000000
C	8.64029772	9.21429320	0.00000000
C	8.60594666	12.03938569	0.00000000
C	8.56164525	13.46729271	0.00000000
C	9.81346953	-11.35823251	0.00000000
C	9.88494336	-9.95054257	0.00000000
C	9.87610531	-7.10962996	0.00000000
C	9.90165285	-5.71360943	0.00000000
C	9.90165599	5.71360866	0.00000000
C	9.87610908	7.10962960	0.00000000
C	9.88494840	9.95054326	0.00000000
C	9.81347491	11.35823375	0.00000000
C	11.08964902	-9.24493516	0.00000000
C	11.13079857	-7.84104573	0.00000000
C	11.09857016	-4.99477865	0.00000000
C	11.11539778	-3.59518182	0.00000000
C	11.11539997	3.59517969	0.00000000
C	11.09857308	4.99477685	0.00000000
C	11.13080299	7.84104484	0.00000000
C	11.08965399	9.24493478	0.00000000
C	12.33057431	-7.11766456	0.00000000
C	12.35863589	-5.71689707	0.00000000
C	12.30535765	-2.87343705	0.00000000
C	12.31168648	-1.46633850	0.00000000
C	12.31168748	1.46633526	0.00000000
C	12.30535950	2.87343399	0.00000000
C	12.35863945	5.71689462	0.00000000
C	12.33057856	7.11766252	0.00000000
C	13.55455328	-4.98043788	0.00000000
C	13.56466894	-3.58422346	0.00000000
C	13.47697602	-0.72543812	0.00000000
C	13.47697653	0.72543408	0.00000000
C	13.56467141	3.58421966	0.00000000
C	13.55455657	4.98043432	0.00000000
C	14.74841073	-2.81898717	0.00000000
C	14.73550163	-1.43253868	0.00000000
C	14.73550275	1.43253380	0.00000000
C	14.74841279	2.81898239	0.00000000
C	15.95005158	-0.68039392	0.00000000
C	15.95005212	0.68038810	0.00000000
H	-16.89716124	-1.23746664	0.00000000
H	-16.89716225	1.23745932	0.00000000
H	-15.71656592	-3.34168775	0.00000000
H	-15.71656838	3.34168163	0.00000000
H	-14.51185387	-5.52244828	0.00000000
H	-14.51185748	5.52244351	0.00000000
H	-13.28289360	-7.66838649	0.00000000

H	-13.28289791	7.66838338	0.00000000
H	-12.03694320	-9.80423888	0.00000000
H	-12.03694787	9.80423753	0.00000000
H	-11.33421768	-0.96468163	0.00000000
H	-11.33421835	0.96467804	0.00000000
H	-10.74983991	-11.93596827	0.00000000
H	-10.74984474	11.93596842	0.00000000
H	-10.15876670	-3.05187433	0.00000000
H	-10.15876837	3.05187177	0.00000000
H	-9.51754113	-14.00908672	0.00000000
H	-9.51754634	14.00908798	0.00000000
H	-8.94937319	-5.16296721	0.00000000
H	-8.94937562	5.16296583	0.00000000
H	-7.72363967	-7.26477731	0.00000000
H	-7.72364268	7.26477719	0.00000000
H	-7.37377284	-15.24611457	0.00000000
H	-7.37377770	15.24611706	0.00000000
H	-6.50150172	-9.32614918	0.00000000
H	-6.50150518	9.32615022	0.00000000
H	-4.96223307	-15.27530841	0.00000000
H	-4.82940813	-10.29139520	0.00000000
H	-4.82941035	10.29139781	0.00000000
H	-4.96223692	15.27531160	0.00000000
H	-2.47234342	-15.32278349	0.00000000
H	-2.43319748	-10.31745684	0.00000000
H	-2.43319835	10.31746015	0.00000000
H	-2.47234555	15.32278738	0.00000000
H	0.00000767	-15.33247139	0.00000000
H	0.00000265	-10.32667097	0.00000000
H	0.00000340	10.32667470	0.00000000
H	0.00000793	15.33247571	0.00000000
H	2.47235849	-15.32277818	0.00000000
H	2.43320255	-10.31745242	0.00000000
H	2.43320484	10.31745619	0.00000000
H	2.47236115	15.32278256	0.00000000
H	4.96224703	-15.27529771	0.00000000
H	4.82941240	-10.29138644	0.00000000
H	4.82941595	10.29138990	0.00000000
H	4.96225153	15.27530179	0.00000000
H	6.50150390	-9.32613878	0.00000000
H	6.50150814	9.32614102	0.00000000
H	7.37378462	-15.24609936	0.00000000
H	7.37379047	15.24610281	0.00000000
H	7.72363863	-7.26476817	0.00000000
H	7.72364217	7.26476920	0.00000000
H	8.94936851	-5.16296027	0.00000000
H	8.94937124	5.16295988	0.00000000
H	9.51754951	-14.00906822	0.00000000
H	9.51755573	14.00907043	0.00000000
H	10.15875850	-3.05187006	0.00000000
H	10.15876031	3.05186837	0.00000000
H	10.74984259	-11.93595037	0.00000000
H	10.74984843	11.93595120	0.00000000
H	11.33420628	-0.96467961	0.00000000
H	11.33420702	0.96467685	0.00000000
H	12.03694094	-9.80422280	0.00000000
H	12.03694633	9.80422200	0.00000000
H	13.28288659	-7.66837323	0.00000000
H	13.28289131	7.66837064	0.00000000
H	14.51184267	-5.52243843	0.00000000
H	14.51184643	5.52243425	0.00000000
H	15.71655099	-3.34168149	0.00000000
H	15.71655348	3.34167606	0.00000000
H	16.89714401	-1.23746499	0.00000000
H	16.89714503	1.23745842	0.00000000

8-HGQR (triplet)

C	-18.40344192	-0.68044817	0.00000000
C	-18.40344352	0.68042353	0.00000000
C	-17.20262494	-2.81810410	0.00000000
C	-17.18886885	-1.43148513	0.00000000
C	-17.18887197	1.43146323	0.00000000
C	-17.20263074	2.81808312	0.00000000
C	-16.01245815	-4.97998923	0.00000000
C	-16.02006848	-3.58355002	0.00000000
C	-15.92984798	-0.72525436	0.00000000
C	-15.92984938	0.72523455	0.00000000
C	-16.02007490	3.58353218	0.00000000
C	-16.01246653	4.97997315	0.00000000
C	-14.79492112	-7.11881217	0.00000000
C	-14.81875805	-5.71844106	0.00000000
C	-14.75965066	-2.87467108	0.00000000
C	-14.76467219	-1.46787853	0.00000000
C	-14.76467456	1.46786132	0.00000000

C	-14.75965493	2.87465483	0.00000000
C	-14.81876628	5.71842869	0.00000000
C	-14.79493067	7.11880222	0.00000000
C	-13.56356889	-9.24803059	0.00000000
C	-13.59757369	-7.84700663	0.00000000
C	-13.55628765	-4.99912927	0.00000000
C	-13.57067651	-3.59971858	0.00000000
C	-13.57068093	3.59970546	0.00000000
C	-13.55629344	4.99911800	0.00000000
C	-13.59758253	7.84700049	0.00000000
C	-13.56357848	9.24802713	0.00000000
C	-12.31728718	-11.36991842	0.00000000
C	-12.36173015	-9.96699786	0.00000000
C	-12.33877907	-7.11902679	0.00000000
C	-12.36153075	-5.72191675	0.00000000
C	-12.36153618	5.72190898	0.00000000
C	-12.33878543	7.11902137	0.00000000
C	-12.36173880	9.96699785	0.00000000
C	-12.31729624	11.36992087	0.00000000
C	-11.03829721	-13.47973949	0.00000000
C	-11.11090426	-12.07301568	0.00000000
C	-11.10854751	-9.23160512	0.00000000
C	-11.13919221	-7.83547092	0.00000000
C	-11.13919793	7.83546900	0.00000000
C	-11.10855390	9.23160555	0.00000000
C	-11.11091245	12.07302092	0.00000000
C	-11.03830555	13.47974670	0.00000000
C	-9.78648859	-15.58726343	0.00000000
C	-9.83003820	-14.15990844	0.00000000
C	-9.86758319	-11.33446220	0.00000000
C	-9.90287339	-9.94216584	0.00000000
C	-9.90287913	9.94216941	0.00000000
C	-9.86758952	11.33446779	0.00000000
C	-9.83004567	14.15991767	0.00000000
C	-9.78649652	15.58727387	0.00000000
C	-8.60779848	-16.26716389	0.00000000
C	-8.58943904	-13.42154425	0.00000000
C	-8.65103167	-12.04101470	0.00000000
C	-8.65103752	12.04102290	0.00000000
C	-8.58944499	13.42155382	0.00000000
C	-8.60780580	16.26717533	0.00000000
C	-7.35060636	-15.59001393	0.00000000
C	-7.33304322	-14.14630958	0.00000000
C	-7.33304816	14.14632064	0.00000000
C	-7.35061222	15.59002584	0.00000000
C	-6.15674691	-16.29513044	0.00000000
C	-6.10728402	-13.50846390	0.00000000
C	-6.10728783	13.50847579	0.00000000
C	-6.15675207	16.29514266	0.00000000
C	-4.90276579	-15.65359288	0.00000000
C	-4.88637920	-14.20743225	0.00000000
C	-4.88638220	14.20744489	0.00000000
C	-4.90276935	15.65360584	0.00000000
C	-3.69007024	-16.34563521	0.00000000
C	-3.66366707	-13.54062228	0.00000000
C	-3.66366924	13.54063559	0.00000000
C	-3.69007340	16.34564829	0.00000000
C	-2.45363330	-15.68144370	0.00000000
C	-2.44474452	-14.22828282	0.00000000
C	-2.44474598	14.22829645	0.00000000
C	-2.45363511	15.68145737	0.00000000
C	-1.22958532	-16.36174314	0.00000000
C	-1.22090668	-13.55570497	0.00000000
C	-1.22090754	13.55571900	0.00000000
C	-1.22958652	16.36175665	0.00000000
C	0.00000704	-15.68963175	0.00000000
C	0.00000577	-14.23531585	0.00000000
C	0.00000554	14.23532980	0.00000000
C	0.00000681	15.68964562	0.00000000
C	1.22960054	-16.36174089	0.00000000
C	1.22091697	-13.55570283	0.00000000
C	1.22091736	13.55571687	0.00000000
C	1.22960128	16.36175438	0.00000000
C	2.45364711	-15.68143922	0.00000000
C	2.44475582	-14.22827850	0.00000000
C	2.44475682	14.22829211	0.00000000
C	2.45364848	15.68145285	0.00000000
C	3.69008492	-16.34562845	0.00000000
C	3.66367691	-13.54061592	0.00000000
C	3.66367856	13.54062920	0.00000000
C	3.69008770	16.34564143	0.00000000
C	4.90277881	-15.65358397	0.00000000
C	4.88638979	-14.20742370	0.00000000

C	4.88639230	14.20743617	0.00000000
C	4.90278200	15.65359672	0.00000000
C	6.15676052	-16.29511925	0.00000000
C	6.10729296	-13.50845344	0.00000000
C	6.10729609	13.50846504	0.00000000
C	6.15676540	16.29513106	0.00000000
C	7.35061810	-15.59000088	0.00000000
C	7.33305241	-14.14629716	0.00000000
C	7.33305680	14.14630755	0.00000000
C	7.35062363	15.59001216	0.00000000
C	8.60781054	-16.26714876	0.00000000
C	8.58944630	-13.42153033	0.00000000
C	8.65103629	-12.04100182	0.00000000
C	8.65104163	12.04100925	0.00000000
C	8.58945167	13.42153900	0.00000000
C	8.60781762	16.26715935	0.00000000
C	9.78649880	-15.58724671	0.00000000
C	9.83004563	-14.15989251	0.00000000
C	9.86758610	-11.33444878	0.00000000
C	9.90287405	-9.94215398	0.00000000
C	9.90287908	9.94215687	0.00000000
C	9.86759176	11.33445355	0.00000000
C	9.83005254	14.15990071	0.00000000
C	9.78650635	15.58725611	0.00000000
C	11.03830254	-13.47972263	0.00000000
C	11.11090713	-12.07300010	0.00000000
C	11.10854620	-9.23159334	0.00000000
C	11.13918868	-7.83546109	0.00000000
C	11.13919351	7.83545843	0.00000000
C	11.10855169	9.23159293	0.00000000
C	11.11091456	12.07300428	0.00000000
C	11.03831018	13.47972869	0.00000000
C	12.31728793	-11.36990274	0.00000000
C	12.36172841	-9.96698399	0.00000000
C	12.33877341	-7.11901752	0.00000000
C	12.36152301	-5.72190963	0.00000000
C	12.36152757	5.72190108	0.00000000
C	12.33877876	7.11901126	0.00000000
C	12.36173605	9.96698294	0.00000000
C	12.31729604	11.36990402	0.00000000
C	13.56356490	-9.24801716	0.00000000
C	13.59756732	-7.84699537	0.00000000
C	13.55627780	-4.99912291	0.00000000
C	13.57066486	-3.59971428	0.00000000
C	13.57066864	3.59970045	0.00000000
C	13.55628268	4.99911090	0.00000000
C	13.59757498	7.84698830	0.00000000
C	13.56357327	9.24801260	0.00000000
C	14.79491253	-7.11880174	0.00000000
C	14.81874735	-5.71843289	0.00000000
C	14.75963701	-2.87466750	0.00000000
C	14.76465700	-1.46787678	0.00000000
C	14.76465906	1.46785909	0.00000000
C	14.75964066	2.87465074	0.00000000
C	14.81875445	5.71841983	0.00000000
C	14.79492077	7.11879093	0.00000000
C	16.01244542	-4.97998196	0.00000000
C	16.02005390	-3.58354488	0.00000000
C	15.92983121	-0.72525365	0.00000000
C	15.92983240	0.72523365	0.00000000
C	16.02005944	3.58352671	0.00000000
C	16.01245262	4.97996540	0.00000000
C	17.20260851	-2.81809975	0.00000000
C	17.18885133	-1.43148279	0.00000000
C	17.18885400	1.43146095	0.00000000
C	17.20261348	2.81807869	0.00000000
C	18.40342326	-0.68044713	0.00000000
C	18.40342462	0.68042286	0.00000000
H	-19.35028905	-1.23791508	0.00000000
H	-19.35029199	1.23788858	0.00000000
H	-18.17118693	-3.34001556	0.00000000
H	-18.17119414	3.33999318	0.00000000
H	-16.97091059	-5.51994140	0.00000000
H	-16.97092068	5.51992422	0.00000000
H	-15.74896796	-7.66652659	0.00000000
H	-15.74897938	7.66651581	0.00000000
H	-14.51391974	-9.80213125	0.00000000
H	-14.51393113	9.80212717	0.00000000
H	-13.78697473	-0.96654408	0.00000000
H	-13.78697605	0.96652867	0.00000000
H	-13.26319883	-11.93154688	0.00000000
H	-13.26320942	11.93154881	0.00000000
H	-12.61303113	-3.05815314	0.00000000

H	-12.61303408	3.05814125	0.00000000
H	-11.97406109	-14.05843944	0.00000000
H	-11.97407076	14.05844605	0.00000000
H	-11.40763998	-5.17403905	0.00000000
H	-11.40764385	5.17403204	0.00000000
H	-10.74253340	-16.12877831	0.00000000
H	-10.74254248	16.12878787	0.00000000
H	-10.18874523	-7.28163945	0.00000000
H	-10.18874949	7.28163791	0.00000000
H	-8.95596194	-9.38206946	0.00000000
H	-8.95596653	9.38207310	0.00000000
H	-8.59773727	-17.36586282	0.00000000
H	-8.59774502	17.36587456	0.00000000
H	-7.72852670	-11.44413245	0.00000000
H	-7.72853205	11.44414030	0.00000000
H	-6.18900058	-17.39488265	0.00000000
H	-6.05269912	-12.41110455	0.00000000
H	-6.05270276	12.41111605	0.00000000
H	-6.18900642	17.39489502	0.00000000
H	-3.70228263	-17.44562670	0.00000000
H	-3.65320268	-12.44053442	0.00000000
H	-3.65320475	12.44054755	0.00000000
H	-3.70228658	17.44563975	0.00000000
H	-1.23315641	-17.46180278	0.00000000
H	-1.21758489	-12.45569579	0.00000000
H	-1.21758577	12.45570982	0.00000000
H	-1.23315787	17.46181617	0.00000000
H	1.23317357	-17.46180045	0.00000000
H	1.21759313	-12.45569375	0.00000000
H	1.21759350	12.45570781	0.00000000
H	1.23317454	17.46181384	0.00000000
H	3.70229922	-17.44561975	0.00000000
H	3.65321050	-12.44052836	0.00000000
H	3.65321198	12.44054154	0.00000000
H	3.70230284	17.44563269	0.00000000
H	6.18901602	-17.39487110	0.00000000
H	6.05270635	-12.41109462	0.00000000
H	6.05270902	12.41110603	0.00000000
H	6.18902168	17.39488308	0.00000000
H	7.72853073	-11.44412156	0.00000000
H	7.72853572	11.44412877	0.00000000
H	8.59775091	-17.36584730	0.00000000
H	8.59775862	17.36585821	0.00000000
H	8.95596269	-9.38205932	0.00000000
H	8.95596659	9.38206253	0.00000000
H	10.18874209	-7.28163130	0.00000000
H	10.18874555	7.28162914	0.00000000
H	10.74254399	-16.12875970	0.00000000
H	10.74255272	16.12876812	0.00000000
H	11.40763287	-5.17403347	0.00000000
H	11.40763602	5.17402563	0.00000000
H	11.97406647	-14.05842098	0.00000000
H	11.97407542	14.05842634	0.00000000
H	12.61302024	-3.05815021	0.00000000
H	12.61302274	3.05813740	0.00000000
H	13.26319930	-11.93152966	0.00000000
H	13.26320886	11.93153030	0.00000000
H	13.78696052	-0.96654324	0.00000000
H	13.78696169	0.96652704	0.00000000
H	14.51391528	-9.80211629	0.00000000
H	14.51392529	9.80211097	0.00000000
H	15.74895879	-7.66651468	0.00000000
H	15.74896869	7.66650297	0.00000000
H	16.97089728	-5.51993273	0.00000000
H	16.97090599	5.51991509	0.00000000
H	18.17116987	-3.34001008	0.00000000
H	18.17117610	3.33998772	0.00000000
H	19.35026950	-1.23791354	0.00000000
H	19.35027206	1.23788763	0.00000000

9-HGQR (triplet)

C	-20.85876726	-0.68043739	0.00000000
C	-20.85876968	0.68042382	0.00000000
C	-19.65822576	-2.81750239	0.00000000
C	-19.64399309	-1.43050991	0.00000000
C	-19.64399847	1.43050067	0.00000000
C	-19.65823645	2.81749204	0.00000000
C	-18.47049095	-4.97980757	0.00000000
C	-18.47656603	-3.58263155	0.00000000
C	-18.38497025	-0.72511151	0.00000000
C	-18.38497318	0.72510753	0.00000000
C	-18.47658063	3.58262482	0.00000000
C	-18.47051154	4.97979883	0.00000000
C	-17.25732763	-7.12003066	0.00000000

C	-17.27869942	-5.71887134	0.00000000
C	-17.21573583	-2.87535272	0.00000000
C	-17.21961119	-1.46902363	0.00000000
C	-17.21961764	1.46902360	0.00000000
C	-17.21574875	2.87535161	0.00000000
C	-17.27872447	5.71886545	0.00000000
C	-17.25735897	7.12002208	0.00000000
C	-16.03244561	-9.25120108	0.00000000
C	-16.06254616	-7.85037635	0.00000000
C	-16.01504626	-5.00196001	0.00000000
C	-16.02714680	-3.60333668	0.00000000
C	-16.02716404	3.60333882	0.00000000
C	-16.01507017	5.00196003	0.00000000
C	-16.06258239	7.85037012	0.00000000
C	-16.03248827	9.25119201	0.00000000
C	-14.79521870	-11.37507585	0.00000000
C	-14.83295601	-9.97524561	0.00000000
C	-14.80130434	-7.12531571	0.00000000
C	-14.82128985	-5.72875568	0.00000000
C	-14.82131847	5.72875810	0.00000000
C	-14.80133969	7.12531539	0.00000000
C	-14.83300354	9.97523908	0.00000000
C	-14.79527250	11.37506715	0.00000000
C	-13.54474853	-13.49326585	0.00000000
C	-13.59116400	-12.09173389	0.00000000
C	-13.57581579	-9.24307759	0.00000000
C	-13.60392313	-7.84583605	0.00000000
C	-13.60396314	7.84583778	0.00000000
C	-13.57586227	9.24307668	0.00000000
C	-13.59122210	12.09172851	0.00000000
C	-13.54481232	13.49325968	0.00000000
C	-12.26354461	-15.60067530	0.00000000
C	-12.33671740	-14.19492826	0.00000000
C	-12.33976503	-11.35360872	0.00000000
C	-12.37499311	-9.95633474	0.00000000
C	-12.37504362	9.95633612	0.00000000
C	-12.33982146	11.35360836	0.00000000
C	-12.33678447	14.19492660	0.00000000
C	-12.26361675	15.60067464	0.00000000
C	-11.01167223	-17.70728862	0.00000000
C	-11.05454755	-16.28025841	0.00000000
C	-11.09482207	-13.45480281	0.00000000
C	-11.13335550	-12.06136839	0.00000000
C	-11.13341485	12.06137114	0.00000000
C	-11.09488651	13.45480534	0.00000000
C	-11.05462142	16.28026347	0.00000000
C	-11.01174978	17.70729613	0.00000000
C	-9.83285964	-18.38691641	0.00000000
C	-9.81486045	-15.54113114	0.00000000
C	-9.87788056	-14.15979877	0.00000000
C	-9.87794647	14.15980569	0.00000000
C	-9.81493037	15.54113982	0.00000000
C	-9.83293714	18.38692964	0.00000000
C	-8.57658159	-17.70879801	0.00000000
C	-8.55852676	-16.26549408	0.00000000
C	-8.55859631	16.26550756	0.00000000
C	-8.57665346	17.70881487	0.00000000
C	-7.38255600	-18.41432931	0.00000000
C	-7.33163082	-15.62795543	0.00000000
C	-7.33169426	15.62797064	0.00000000
C	-7.38262421	18.41435108	0.00000000
C	-6.12941392	-17.77323058	0.00000000
C	-6.11157898	-16.32751523	0.00000000
C	-6.11163766	16.32753272	0.00000000
C	-6.12947477	17.77325337	0.00000000
C	-4.91684497	-18.46689786	0.00000000
C	-4.88653486	-15.66242743	0.00000000
C	-4.88658540	15.66244456	0.00000000
C	-4.91689880	18.46692424	0.00000000
C	-3.68090162	-17.80458826	0.00000000
C	-3.66937598	-16.35160577	0.00000000
C	-3.66941865	16.35162529	0.00000000
C	-3.68094556	17.80461471	0.00000000
C	-2.45747459	-18.48745236	0.00000000
C	-2.44252309	-15.68192762	0.00000000
C	-2.44255556	15.68194608	0.00000000
C	-2.45750879	18.48748204	0.00000000
C	-1.22732062	-17.81867056	0.00000000
C	-1.22346623	-16.36369632	0.00000000
C	-1.22348832	16.36371696	0.00000000
C	-1.22734316	17.81869919	0.00000000
C	-0.00000949	-18.49423459	0.00000000
C	-0.00000534	-15.68819291	0.00000000

C	-0.00001625	15.68821177	0.00000000
C	-0.00002044	18.49426559	0.00000000
C	1.22730375	-17.81867437	0.00000000
C	1.22345361	-16.36369995	0.00000000
C	1.22345392	16.36372059	0.00000000
C	1.22730452	17.81870306	0.00000000
C	2.45745597	-18.48746003	0.00000000
C	2.44251284	-15.68193475	0.00000000
C	2.44252362	15.68195314	0.00000000
C	2.45746856	18.48748983	0.00000000
C	3.68088547	-17.80459968	0.00000000
C	3.66936413	-16.35161672	0.00000000
C	3.66938541	16.35163614	0.00000000
C	3.68090812	17.80462623	0.00000000
C	4.91682728	-18.46691333	0.00000000
C	4.88652569	-15.66244181	0.00000000
C	4.88655516	15.66245866	0.00000000
C	4.91686027	18.46693980	0.00000000
C	6.12939915	-17.77324985	0.00000000
C	6.11156850	-16.32753367	0.00000000
C	6.11160670	16.32755082	0.00000000
C	6.12943961	17.77327264	0.00000000
C	7.38253983	-18.41435305	0.00000000
C	7.33162329	-15.62797734	0.00000000
C	7.33166689	15.62799190	0.00000000
C	7.38258836	18.41437474	0.00000000
C	8.57656852	-17.70882511	0.00000000
C	8.55851826	-16.26552026	0.00000000
C	8.55856910	16.26553276	0.00000000
C	8.57662141	17.70884171	0.00000000
C	9.83284585	-18.38694687	0.00000000
C	9.81485441	-15.54116056	0.00000000
C	9.87787805	-14.15982671	0.00000000
C	9.87792790	14.15983057	0.00000000
C	9.81490696	15.54116761	0.00000000
C	9.83290506	18.38696026	0.00000000
C	11.01166136	-17.70732179	0.00000000
C	11.05454105	-16.28029062	0.00000000
C	11.09482196	-13.45483238	0.00000000
C	11.13335837	-12.06139599	0.00000000
C	11.13340422	12.06139225	0.00000000
C	11.09487165	13.45483053	0.00000000
C	11.05459846	16.28029483	0.00000000
C	11.01172156	17.70732942	0.00000000
C	12.26354130	-15.60070963	0.00000000
C	12.33671715	-14.19496132	0.00000000
C	12.33977078	-11.35363769	0.00000000
C	12.37500190	-9.95636132	0.00000000
C	12.37504204	9.95635151	0.00000000
C	12.33981541	11.35362879	0.00000000
C	12.33677051	14.19495631	0.00000000
C	12.26359837	15.60070742	0.00000000
C	13.54475148	-13.49330006	0.00000000
C	13.59117012	-12.09176634	0.00000000
C	13.57582782	-9.24310500	0.00000000
C	13.60393793	-7.84586080	0.00000000
C	13.60397099	7.84584580	0.00000000
C	13.57586583	9.24309034	0.00000000
C	13.59121778	12.09175359	0.00000000
C	13.54480321	13.49328909	0.00000000
C	14.79522833	-11.37510913	0.00000000
C	14.83296886	-9.97527651	0.00000000
C	14.80132259	-7.12534080	0.00000000
C	14.82131061	-5.72877805	0.00000000
C	14.82133544	5.72875834	0.00000000
C	14.80135282	7.12532114	0.00000000
C	14.83300956	9.97525734	0.00000000
C	14.79527367	11.37509086	0.00000000
C	16.03246205	-9.25123233	0.00000000
C	16.06256556	-7.85040476	0.00000000
C	16.01507046	-5.00198231	0.00000000
C	16.02717322	-3.60335639	0.00000000
C	16.02718906	3.60333210	0.00000000
C	16.01509207	5.00195798	0.00000000
C	16.06259833	7.85038013	0.00000000
C	16.03249990	9.25120791	0.00000000
C	17.25735040	-7.12005907	0.00000000
C	17.27872492	-5.71889672	0.00000000
C	17.21576579	-2.87537226	0.00000000
C	17.21964349	-1.46904065	0.00000000
C	17.21964984	1.46901154	0.00000000
C	17.21577824	2.87534315	0.00000000
C	17.27874911	5.71886713	0.00000000

C	17.25738004	7.12002932	0.00000000
C	18.47051961	-4.97983298	0.00000000
C	18.47659751	-3.58265387	0.00000000
C	18.38500577	-0.72512740	0.00000000
C	18.38500885	0.72509359	0.00000000
C	18.47661272	3.58261942	0.00000000
C	18.47054053	4.97979824	0.00000000
C	19.65826052	-2.81752484	0.00000000
C	19.64402947	-1.43052914	0.00000000
C	19.64403565	1.43048986	0.00000000
C	19.65827237	2.81748532	0.00000000
C	20.85880547	-0.68045439	0.00000000
C	20.85880834	0.68040989	0.00000000
H	-21.80536026	-1.23830684	0.00000000
H	-21.80536470	1.23828933	0.00000000
H	-20.62702882	-3.33895781	0.00000000
H	-20.62704116	3.33894295	0.00000000
H	-19.42975951	-5.51831573	0.00000000
H	-19.42978142	5.51830226	0.00000000
H	-18.21265146	-7.66553681	0.00000000
H	-18.21268375	7.66552358	0.00000000
H	-16.98452074	-9.80236459	0.00000000
H	-16.98456421	9.80235115	0.00000000
H	-16.24179473	-0.96787605	0.00000000
H	-16.24179967	0.96787941	0.00000000
H	-15.74405252	-11.93179096	0.00000000
H	-15.74410731	11.93177837	0.00000000
H	-15.06875255	-3.06307642	0.00000000
H	-15.06876853	3.06308244	0.00000000
H	-14.48961495	-14.05665716	0.00000000
H	-14.48968020	14.05664771	0.00000000
H	-13.86623969	-5.18286640	0.00000000
H	-13.86626722	5.18287297	0.00000000
H	-13.19887640	-16.18006393	0.00000000
H	-13.19895071	16.18006055	0.00000000
H	-12.65188349	-7.29472458	0.00000000
H	-12.65192223	7.29473059	0.00000000
H	-11.96783794	-18.24855826	0.00000000
H	-11.96791793	18.24856383	0.00000000
H	-11.42593307	-9.40009470	0.00000000
H	-11.42598173	9.40010024	0.00000000
H	-10.18740180	-11.49963465	0.00000000
H	-10.18745840	11.49964111	0.00000000
H	-9.82204789	-19.48559197	0.00000000
H	-9.82212851	19.48560753	0.00000000
H	-8.95564023	-13.56246697	0.00000000
H	-8.95570224	13.56247673	0.00000000
H	-7.41515598	-19.51406622	0.00000000
H	-7.27698781	-14.53057671	0.00000000
H	-7.27704759	14.53058952	0.00000000
H	-7.41522544	19.51409134	0.00000000
H	-4.93065462	-19.56687222	0.00000000
H	-4.87463283	-14.56234366	0.00000000
H	-4.87468122	14.56235659	0.00000000
H	-4.93070895	19.56690324	0.00000000
H	-2.46363509	-19.58750892	0.00000000
H	-2.43685966	-14.58191146	0.00000000
H	-2.43689099	14.58192456	0.00000000
H	-2.46366961	19.58754429	0.00000000
H	-0.00001111	-19.59431166	0.00000000
H	-0.00000353	-14.58816927	0.00000000
H	-0.00001439	14.58818229	0.00000000
H	-0.00002212	19.59434879	0.00000000
H	2.46361341	-19.58751683	0.00000000
H	2.43685324	-14.58191821	0.00000000
H	2.43686277	14.58193112	0.00000000
H	2.46362626	19.58755242	0.00000000
H	4.93063369	-19.56688816	0.00000000
H	4.87462758	-14.56235729	0.00000000
H	4.87465470	14.56236964	0.00000000
H	4.93066721	19.56691947	0.00000000
H	7.41513672	-19.51409064	0.00000000
H	7.27698443	-14.53059734	0.00000000
H	7.27702362	14.53060908	0.00000000
H	7.41518649	19.51411598	0.00000000
H	8.95563850	-13.56249121	0.00000000
H	8.95568431	13.56249709	0.00000000
H	9.82203206	-19.48562315	0.00000000
H	9.82209396	19.48563927	0.00000000
H	10.18740470	-11.49965868	0.00000000
H	10.18744709	11.49965803	0.00000000
H	11.42594137	-9.40011770	0.00000000
H	11.42597872	9.40011144	0.00000000

H	11.96782642	-18.24859464	0.00000000
H	11.96788944	18.24860087	0.00000000
H	12.65189718	-7.29474617	0.00000000
H	12.65192802	7.29473480	0.00000000
H	13.19887319	-16.18010055	0.00000000
H	13.19893297	16.18009637	0.00000000
H	13.86625882	-5.18288615	0.00000000
H	13.86628180	5.18287001	0.00000000
H	14.48961873	-14.05669337	0.00000000
H	14.48967263	14.05668010	0.00000000
H	15.06877715	-3.06309375	0.00000000
H	15.06879121	3.06307300	0.00000000
H	15.74406318	-11.93182666	0.00000000
H	15.74411054	11.93180540	0.00000000
H	16.24182476	-0.96789122	0.00000000
H	16.24182953	0.96786540	0.00000000
H	16.98453832	-9.80239858	0.00000000
H	16.98457820	9.80237048	0.00000000
H	18.21267532	-7.66556818	0.00000000
H	18.21270712	7.66553416	0.00000000
H	19.42978902	-5.51834437	0.00000000
H	19.42981228	5.51830498	0.00000000
H	20.62706472	-3.33898264	0.00000000
H	20.62707889	3.33893851	0.00000000
H	21.80540065	-1.23832430	0.00000000
H	21.80540608	1.23827533	0.00000000

10-HGQR (triplet)

C	-23.31581926	-0.68034954	0.00000000
C	-23.31581908	0.68034154	0.00000000
C	-22.11553066	-2.81698568	0.00000000
C	-22.10079064	-1.42989202	0.00000000
C	-22.10079024	1.42988370	0.00000000
C	-22.11552988	2.81697743	0.00000000
C	-20.92962948	-4.97966692	0.00000000
C	-20.93441148	-3.58202012	0.00000000
C	-20.84171917	-0.72517251	0.00000000
C	-20.84171896	0.72516377	0.00000000
C	-20.93441045	3.58201164	0.00000000
C	-20.92962810	4.97965860	0.00000000
C	-19.71962703	-7.12111801	0.00000000
C	-19.73919275	-5.71908244	0.00000000
C	-19.67333904	-2.87603784	0.00000000
C	-19.67636256	-1.46984223	0.00000000
C	-19.67636208	1.46983322	0.00000000
C	-19.67333811	2.87602895	0.00000000
C	-19.73919114	5.71907408	0.00000000
C	-19.71962513	7.12110992	0.00000000
C	-18.49927018	-9.25407549	0.00000000
C	-18.52694825	-7.85236477	0.00000000
C	-18.47490911	-5.00417107	0.00000000
C	-18.48509354	-3.60611506	0.00000000
C	-18.48509223	3.60610603	0.00000000
C	-18.47490744	5.00416235	0.00000000
C	-18.52694613	7.85235688	0.00000000
C	-18.49926786	9.25406800	0.00000000
C	-17.26827141	-11.38003149	0.00000000
C	-17.30238234	-9.98038115	0.00000000
C	-17.26440552	-7.12986141	0.00000000
C	-17.28173505	-5.73421409	0.00000000
C	-17.28173295	5.73420546	0.00000000
C	-17.26440317	7.12985325	0.00000000
C	-17.30237977	9.98037409	0.00000000
C	-17.26826875	11.38002492	0.00000000
C	-16.02620121	-13.50012137	0.00000000
C	-16.06641523	-12.10170803	0.00000000
C	-16.04280979	-9.25104293	0.00000000
C	-16.06809545	-7.85444465	0.00000000
C	-16.06809265	7.85443682	0.00000000
C	-16.04280683	9.25103568	0.00000000
C	-16.06641233	12.10170207	0.00000000
C	-16.02619832	13.50011593	0.00000000
C	-14.77221476	-15.61552877	0.00000000
C	-14.82015655	-14.21503695	0.00000000
C	-14.81121901	-11.36648215	0.00000000
C	-14.84416067	-9.96813797	0.00000000
C	-14.84415730	9.96813129	0.00000000
C	-14.81121561	11.36647610	0.00000000
C	-14.82015341	14.21503217	0.00000000
C	-14.77221176	15.61552448	0.00000000
C	-13.48905881	-17.72097433	0.00000000
C	-13.56286050	-16.31578785	0.00000000
C	-13.57050061	-13.47481260	0.00000000
C	-13.60950507	-12.07641968	0.00000000

C	-13.60950134	12.07641432	0.00000000
C	-13.57049695	13.47480783	0.00000000
C	-13.56285730	16.31578423	0.00000000
C	-13.48905589	17.72097115	0.00000000
C	-12.23692010	-19.82673530	0.00000000
C	-12.27951947	-18.39976788	0.00000000
C	-12.32224210	-15.57434322	0.00000000
C	-12.36332542	-14.18015767	0.00000000
C	-12.36332154	14.18015361	0.00000000
C	-12.32223840	15.57433968	0.00000000
C	-12.27951634	18.39976528	0.00000000
C	-12.23691718	19.82673306	0.00000000
C	-11.05809702	-20.50596733	0.00000000
C	-11.04061658	-17.65986734	0.00000000
C	-11.10474782	-16.27815960	0.00000000
C	-11.10474399	16.27815675	0.00000000
C	-11.04061288	17.65986493	0.00000000
C	-11.05809395	20.50596562	0.00000000
C	-9.80235788	-19.82699859	0.00000000
C	-9.78397890	-18.38397646	0.00000000
C	-9.78397502	18.38397459	0.00000000
C	-9.80235425	19.82699715	0.00000000
C	-8.60838001	-20.53278384	0.00000000
C	-8.55652263	-17.74658416	0.00000000
C	-8.55651809	17.74658258	0.00000000
C	-8.60837607	20.53278296	0.00000000
C	-7.35573132	-19.89186925	0.00000000
C	-7.33685509	-18.44658902	0.00000000
C	-7.33685016	18.44658774	0.00000000
C	-7.35572671	19.89186847	0.00000000
C	-6.14335592	-20.58676624	0.00000000
C	-6.11020872	-17.78271137	0.00000000
C	-6.11020313	17.78271024	0.00000000
C	-6.14335081	20.58676592	0.00000000
C	-4.90787433	-19.92567410	0.00000000
C	-4.89439880	-18.47314192	0.00000000
C	-4.89439267	18.47314107	0.00000000
C	-4.90786840	19.92567380	0.00000000
C	-3.68461489	-20.61052043	0.00000000
C	-3.66500037	-17.80556303	0.00000000
C	-3.66499356	17.80556226	0.00000000
C	-3.68460834	20.61052050	0.00000000
C	-2.45476666	-19.94398264	0.00000000
C	-2.44801710	-18.48915569	0.00000000
C	-2.44800964	18.48915512	0.00000000
C	-2.45475927	19.94398262	0.00000000
C	-1.22799890	-20.62218841	0.00000000
C	-1.22147916	-17.81670199	0.00000000
C	-1.22147107	17.81670140	0.00000000
C	-1.22799085	20.62218860	0.00000000
C	-0.00000484	-19.95010435	0.00000000
C	-0.00000530	-18.49443143	0.00000000
C	0.00000340	18.49443098	0.00000000
C	0.00000389	19.95010436	0.00000000
C	1.22798977	-20.62218783	0.00000000
C	1.22146826	-17.81670117	0.00000000
C	1.22147750	17.81670064	0.00000000
C	1.22799909	20.62218788	0.00000000
C	2.45475755	-19.94398137	0.00000000
C	2.44800707	-18.48915408	0.00000000
C	2.44801673	18.48915363	0.00000000
C	2.45476729	19.94398124	0.00000000
C	3.68460690	-20.61051858	0.00000000
C	3.66499053	-17.80556055	0.00000000
C	3.66500056	17.80556002	0.00000000
C	3.68461702	20.61051839	0.00000000
C	4.90786672	-19.92567141	0.00000000
C	4.89439023	-18.47313866	0.00000000
C	4.89440046	18.47313819	0.00000000
C	4.90787699	19.92567109	0.00000000
C	6.14334963	-20.58676301	0.00000000
C	6.11020058	-17.78270723	0.00000000
C	6.11021097	17.78270671	0.00000000
C	6.14336005	20.58676257	0.00000000
C	7.35572554	-19.89186517	0.00000000
C	7.33684832	-18.44658421	0.00000000
C	7.33685870	18.44658372	0.00000000
C	7.35573590	19.89186466	0.00000000
C	8.60837534	-20.53277940	0.00000000
C	8.55651634	-17.74657852	0.00000000
C	8.55652665	17.74657805	0.00000000
C	8.60838566	20.53277872	0.00000000
C	9.80235358	-19.82699320	0.00000000

C	9.78397394	-18.38397030	0.00000000
C	9.78398403	18.38396980	0.00000000
C	9.80236369	19.82699252	0.00000000
C	11.05809389	-20.50596115	0.00000000
C	11.04061179	-17.65986021	0.00000000
C	11.10474268	-16.27815137	0.00000000
C	11.10475239	16.27815162	0.00000000
C	11.04062163	17.65985989	0.00000000
C	11.05810375	20.50596037	0.00000000
C	12.23691715	-19.82672813	0.00000000
C	12.27951596	-18.39976001	0.00000000
C	12.32223713	-15.57433374	0.00000000
C	12.36331991	-14.18014701	0.00000000
C	12.36332920	14.18014890	0.00000000
C	12.32224653	15.57433468	0.00000000
C	12.27952534	18.39975966	0.00000000
C	12.23692669	19.82672742	0.00000000
C	13.48905576	-17.72096549	0.00000000
C	13.56285665	-16.31577816	0.00000000
C	13.57049555	-13.47480088	0.00000000
C	13.60949967	-12.07640676	0.00000000
C	13.60950804	12.07641085	0.00000000
C	13.57050423	13.47480370	0.00000000
C	13.56286541	16.31577890	0.00000000
C	13.48906459	17.72096551	0.00000000
C	14.77221135	-15.61551793	0.00000000
C	14.82015262	-14.21502514	0.00000000
C	14.81121429	-11.36646838	0.00000000
C	14.84415562	-9.96812317	0.00000000
C	14.84416264	9.96812993	0.00000000
C	14.81122176	11.36647365	0.00000000
C	14.82016043	14.21502765	0.00000000
C	14.77221943	15.61551940	0.00000000
C	16.02619779	-13.50010859	0.00000000
C	16.06641149	-12.10169430	0.00000000
C	16.04280547	-9.25102773	0.00000000
C	16.06809083	-7.85442885	0.00000000
C	16.06809615	7.85443824	0.00000000
C	16.04281139	9.25103563	0.00000000
C	16.06641796	12.10169911	0.00000000
C	16.02620472	13.50011207	0.00000000
C	17.26826809	-11.38001702	0.00000000
C	17.30237868	-9.98036608	0.00000000
C	17.26440150	-7.12984574	0.00000000
C	17.28173075	-5.73419838	0.00000000
C	17.28173428	5.73420990	0.00000000
C	17.26440571	7.12985606	0.00000000
C	17.30238362	9.98037342	0.00000000
C	17.26827357	11.38002293	0.00000000
C	18.49926677	-9.25406012	0.00000000
C	18.52694445	-7.85234931	0.00000000
C	18.47490524	-5.00415592	0.00000000
C	18.48508952	-3.60610035	0.00000000
C	18.48509142	3.60611322	0.00000000
C	18.47490780	5.00416800	0.00000000
C	18.52694789	7.85235899	0.00000000
C	18.49927075	9.25406850	0.00000000
C	19.71962329	-7.12110282	0.00000000
C	19.73918867	-5.71906760	0.00000000
C	19.67333535	-2.87602411	0.00000000
C	19.67635899	-1.46982929	0.00000000
C	19.67635955	1.46984265	0.00000000
C	19.67333651	2.87603711	0.00000000
C	19.73919076	5.71907904	0.00000000
C	19.71962591	7.12111329	0.00000000
C	20.92962542	-4.97965301	0.00000000
C	20.93440735	-3.58200670	0.00000000
C	20.84171596	-0.72516075	0.00000000
C	20.84171615	0.72517402	0.00000000
C	20.93440834	3.58201915	0.00000000
C	20.92962688	4.97966484	0.00000000
C	22.11552661	-2.81697365	0.00000000
C	22.10078658	-1.42988065	0.00000000
C	22.10078685	1.42989347	0.00000000
C	22.11552717	2.81698616	0.00000000
C	23.31581488	-0.68033891	0.00000000
C	23.31581496	0.68035156	0.00000000
H	-24.26223842	-1.23851118	0.00000000
H	-24.26223808	1.23850348	0.00000000
H	-23.08450218	-3.33812010	0.00000000
H	-23.08450129	3.33811218	0.00000000
H	-21.88951140	-5.51708566	0.00000000
H	-21.88950997	5.51707763	0.00000000

H	-20.67589857	-7.66497930	0.00000000
H	-20.67589673	7.66497145	0.00000000
H	-19.45264794	-9.80301627	0.00000000
H	-19.45264580	9.80300895	0.00000000
H	-18.69843025	-0.96887454	0.00000000
H	-18.69842981	0.96886534	0.00000000
H	-18.21876209	-11.93395505	0.00000000
H	-18.21875973	11.93394857	0.00000000
H	-17.52610688	-3.06689302	0.00000000
H	-17.52610549	3.06688379	0.00000000
H	-16.97381688	-14.05892397	0.00000000
H	-16.97381436	14.05891853	0.00000000
H	-16.32577850	-5.18988373	0.00000000
H	-16.32577618	5.18987493	0.00000000
H	-15.71621954	-16.18036628	0.00000000
H	-15.71621695	16.18036189	0.00000000
H	-15.11485929	-7.30536293	0.00000000
H	-15.11485615	7.30535494	0.00000000
H	-14.42401967	-18.30095252	0.00000000
H	-14.42401714	18.30094922	0.00000000
H	-13.89359231	-9.41444998	0.00000000
H	-13.89358854	9.41444318	0.00000000
H	-13.19313549	-20.36791259	0.00000000
H	-13.19313289	20.36791028	0.00000000
H	-12.66160926	-11.51816777	0.00000000
H	-12.66160512	11.51816233	0.00000000
H	-11.41819852	-13.61701880	0.00000000
H	-11.41819424	13.61701473	0.00000000
H	-11.04662119	-21.60463434	0.00000000
H	-11.04661844	21.60463292	0.00000000
H	-10.18278484	-15.68035740	0.00000000
H	-10.18278065	15.68035453	0.00000000
H	-8.64111957	-21.63251422	0.00000000
H	-8.50189199	-16.64918123	0.00000000
H	-8.50188710	16.64917936	0.00000000
H	-8.64111583	21.63251368	0.00000000
H	-6.15830026	-21.68673023	0.00000000
H	-6.09722835	-16.68262973	0.00000000
H	-6.09722254	16.68262829	0.00000000
H	-6.15829530	21.68673032	0.00000000
H	-3.69265903	-21.71057655	0.00000000
H	-3.65754844	-16.70553905	0.00000000
H	-3.65754151	16.70553794	0.00000000
H	-3.69265261	21.71057705	0.00000000
H	-1.23067342	-21.72228174	0.00000000
H	-1.21902365	-16.71665611	0.00000000
H	-1.21901556	16.71665521	0.00000000
H	-1.23066547	21.72228232	0.00000000
H	1.23066494	-21.72228129	0.00000000
H	1.21901218	-16.71665519	0.00000000
H	1.21902134	16.71665441	0.00000000
H	1.23067420	21.72228163	0.00000000
H	3.69265180	-21.71057504	0.00000000
H	3.65753804	-16.70553627	0.00000000
H	3.65754794	16.70553561	0.00000000
H	3.69266187	21.71057499	0.00000000
H	6.15829475	-21.68672744	0.00000000
H	6.09721981	-16.68262515	0.00000000
H	6.09723007	16.68262465	0.00000000
H	6.15830515	21.68672702	0.00000000
H	8.64111565	-21.63251022	0.00000000
H	8.50188545	-16.64917505	0.00000000
H	8.50189561	16.64917479	0.00000000
H	8.64112596	21.63250946	0.00000000
H	10.18277922	-15.68034872	0.00000000
H	10.18278910	15.68034931	0.00000000
H	11.04661928	-21.60462862	0.00000000
H	11.04662904	21.60462768	0.00000000
H	11.41819233	-13.61700781	0.00000000
H	11.41820206	13.61701007	0.00000000
H	12.66160319	-11.51815439	0.00000000
H	12.66161218	11.51815896	0.00000000
H	13.19313325	-20.36790518	0.00000000
H	13.19314253	20.36790432	0.00000000
H	13.89358659	-9.41443484	0.00000000
H	13.89359434	9.41444207	0.00000000
H	14.42401754	-18.30094332	0.00000000
H	14.42402587	18.30094330	0.00000000
H	15.11485408	-7.30534698	0.00000000
H	15.11486012	7.30535680	0.00000000
H	15.71621722	-16.18035508	0.00000000
H	15.71622462	16.18035645	0.00000000
H	16.32577380	-5.18986804	0.00000000

H	16.32577790	5.18987992	0.00000000
H	16.97381442	-14.05891115	0.00000000
H	16.97382062	14.05891435	0.00000000
H	17.52610284	-3.06687816	0.00000000
H	17.52610508	3.06689127	0.00000000
H	18.21875954	-11.93394071	0.00000000
H	18.21876431	11.93394619	0.00000000
H	18.69842698	-0.96886115	0.00000000
H	18.69842764	0.96887454	0.00000000
H	19.45264498	-9.80300116	0.00000000
H	19.45264838	9.80300899	0.00000000
H	20.67589497	-7.66496447	0.00000000
H	20.67589721	7.66497433	0.00000000
H	21.88950716	-5.51707229	0.00000000
H	21.88950845	5.51708351	0.00000000
H	23.08449790	-3.33810837	0.00000000
H	23.08449843	3.33812041	0.00000000
H	24.26223413	-1.23850016	0.00000000
H	24.26223431	1.23851250	0.00000000

11-HGQR (triplet)

C	-25.77372936	-0.68024104	0.00000000
C	-25.77372941	0.68023886	0.00000000
C	-24.57379467	-2.81648649	0.00000000
C	-24.55848190	-1.42951197	0.00000000
C	-24.55848199	1.42950989	0.00000000
C	-24.57379485	2.81648446	0.00000000
C	-23.38949637	-4.97945723	0.00000000
C	-23.39304137	-3.58171067	0.00000000
C	-23.29921207	-0.72530073	0.00000000
C	-23.29921211	0.72529870	0.00000000
C	-23.39304155	3.58170876	0.00000000
C	-23.38949659	4.97945543	0.00000000
C	-22.18203115	-7.12187990	0.00000000
C	-22.19997025	-5.71934034	0.00000000
C	-22.13164661	-2.87673955	0.00000000
C	-22.13403540	-1.47045860	0.00000000
C	-22.13403546	1.47045667	0.00000000
C	-22.13164670	2.87673767	0.00000000
C	-22.19997043	5.71933870	0.00000000
C	-22.18203135	7.12187840	0.00000000
C	-20.96511747	-9.25635591	0.00000000
C	-20.99088395	-7.85373745	0.00000000
C	-20.93515678	-5.00603044	0.00000000
C	-20.94383360	-3.60820013	0.00000000
C	-20.94383367	3.60819839	0.00000000
C	-20.93515686	5.00602880	0.00000000
C	-20.99088407	7.85373613	0.00000000
C	-20.96511760	9.25635476	0.00000000
C	-19.73864898	-11.38420391	0.00000000
C	-19.77042690	-9.98366990	0.00000000
C	-19.72748963	-7.13337440	0.00000000
C	-19.74253115	-5.73840062	0.00000000
C	-19.74253117	5.73839915	0.00000000
C	-19.72748964	7.13337309	0.00000000
C	-19.77042692	9.98366895	0.00000000
C	-19.73864900	11.38420312	0.00000000
C	-18.50252513	-13.50631366	0.00000000
C	-18.53937475	-12.10811270	0.00000000
C	-18.50941736	-9.25684985	0.00000000
C	-18.53187001	-7.86127413	0.00000000
C	-18.53186993	7.86127300	0.00000000
C	-18.50941727	9.25684889	0.00000000
C	-18.53937467	12.10811208	0.00000000
C	-18.50252506	13.50631319	0.00000000
C	-17.25635624	-15.62341178	0.00000000
C	-17.29853195	-14.22606483	0.00000000
C	-17.28171033	-11.37550339	0.00000000
C	-17.31186567	-9.97788797	0.00000000
C	-17.31186550	9.97788719	0.00000000
C	-17.28171016	11.37550278	0.00000000
C	-17.29853181	14.22606450	0.00000000
C	-17.25635612	15.62341158	0.00000000
C	-15.99934987	-17.73643489	0.00000000
C	-16.04872378	-16.33654064	0.00000000
C	-16.04515558	-13.48836376	0.00000000
C	-16.08211260	-12.08896395	0.00000000
C	-16.08211236	12.08896350	0.00000000
C	-16.04515535	13.48836344	0.00000000
C	-16.04872360	16.33654055	0.00000000
C	-15.99934972	17.73643488	0.00000000
C	-14.71438642	-19.84005378	0.00000000
C	-14.78900908	-18.43513533	0.00000000
C	-14.80053058	-15.59448273	0.00000000

C	-14.84251939	-14.19540482	0.00000000
C	-14.84251912	14.19540465	0.00000000
C	-14.80053033	15.59448265	0.00000000
C	-14.78900890	18.43513541	0.00000000
C	-14.71438626	19.84005392	0.00000000
C	-13.46184007	-21.94486795	0.00000000
C	-13.50444680	-20.51786613	0.00000000
C	-13.54939660	-17.69241924	0.00000000
C	-13.59248623	-16.29791688	0.00000000
C	-13.59248596	16.29791691	0.00000000
C	-13.54939636	17.69241935	0.00000000
C	-13.50444662	20.51786633	0.00000000
C	-13.46183993	21.94486817	0.00000000
C	-12.28302252	-22.62365427	0.00000000
C	-12.26612451	-19.77711756	0.00000000
C	-12.33125343	-18.39532634	0.00000000
C	-12.33125320	18.39532654	0.00000000
C	-12.26612428	19.77711781	0.00000000
C	-12.28302237	22.62365456	0.00000000
C	-11.02763129	-21.94396772	0.00000000
C	-11.00903902	-20.50101252	0.00000000
C	-11.00903878	20.50101284	0.00000000
C	-11.02763110	21.94396804	0.00000000
C	-9.83390396	-22.64991955	0.00000000
C	-9.78136558	-19.86375270	0.00000000
C	-9.78136527	19.86375307	0.00000000
C	-9.83390377	22.64991991	0.00000000
C	-8.58143304	-22.00914610	0.00000000
C	-8.56176697	-20.56409021	0.00000000
C	-8.56176667	20.56409065	0.00000000
C	-8.58143279	22.00914653	0.00000000
C	-7.36951959	-22.70499594	0.00000000
C	-7.33420484	-19.90110516	0.00000000
C	-7.33420449	19.90110565	0.00000000
C	-7.36951934	22.70499640	0.00000000
C	-6.13420204	-22.04475302	0.00000000
C	-6.11922429	-20.59256268	0.00000000
C	-6.11922394	20.59256321	0.00000000
C	-6.13420174	22.04475354	0.00000000
C	-4.91131152	-22.73118604	0.00000000
C	-4.88809508	-19.92654473	0.00000000
C	-4.88809469	19.92654530	0.00000000
C	-4.91131120	22.73118658	0.00000000
C	-3.68176537	-22.06620171	0.00000000
C	-3.67277206	-20.61170858	0.00000000
C	-3.67277165	20.61170917	0.00000000
C	-3.68176498	22.06620230	0.00000000
C	-2.45522825	-22.74655021	0.00000000
C	-2.44365689	-19.94154030	0.00000000
C	-2.44365643	19.94154090	0.00000000
C	-2.45522781	22.74655079	0.00000000
C	-1.22747255	-22.07690048	0.00000000
C	-1.22447521	-20.62124284	0.00000000
C	-1.22447470	20.62124344	0.00000000
C	-1.22747204	22.07690109	0.00000000
C	-0.00001842	-22.75160543	0.00000000
C	-0.00001373	-19.94658880	0.00000000
C	-0.00001315	19.94658938	0.00000000
C	-0.00001781	22.75160603	0.00000000
C	1.22743811	-22.07690459	0.00000000
C	1.22444565	-20.62124689	0.00000000
C	1.22444633	20.62124745	0.00000000
C	1.22743881	22.07690518	0.00000000
C	2.45519200	-22.74655848	0.00000000
C	2.44362982	-19.94154845	0.00000000
C	2.44363059	19.94154897	0.00000000
C	2.45519285	22.74655905	0.00000000
C	3.68173200	-22.06621393	0.00000000
C	3.67274334	-20.61172060	0.00000000
C	3.67274422	20.61172108	0.00000000
C	3.68173294	22.06621447	0.00000000
C	4.91127697	-22.73120229	0.00000000
C	4.88806927	-19.92656069	0.00000000
C	4.88807025	19.92656113	0.00000000
C	4.91127808	22.73120279	0.00000000
C	6.13417069	-22.04477304	0.00000000
C	6.11919730	-20.59258235	0.00000000
C	6.11919842	20.59258272	0.00000000
C	6.13417188	22.04477347	0.00000000
C	7.36948749	-22.70501977	0.00000000
C	7.33418105	-19.90112851	0.00000000
C	7.33418222	19.90112882	0.00000000
C	7.36948881	22.70502013	0.00000000

C	8.58140428	-22.00917347	0.00000000
C	8.56174243	-20.56411709	0.00000000
C	8.56174372	20.56411733	0.00000000
C	8.58140565	22.00917374	0.00000000
C	9.83387462	-22.64995068	0.00000000
C	9.78134412	-19.86378294	0.00000000
C	9.78134543	19.86378312	0.00000000
C	9.83387610	22.64995088	0.00000000
C	11.02760548	-21.94400177	0.00000000
C	11.00901762	-20.50104568	0.00000000
C	11.00901905	20.50104577	0.00000000
C	11.02760697	21.94400187	0.00000000
C	12.28299599	-22.62369183	0.00000000
C	12.26610621	-19.77715343	0.00000000
C	12.33123992	-18.39536047	0.00000000
C	12.33124130	18.39536048	0.00000000
C	12.26610762	19.77715344	0.00000000
C	12.28299755	22.62369185	0.00000000
C	13.46181685	-21.94490776	0.00000000
C	13.50442782	-20.51790499	0.00000000
C	13.54938591	-17.69245481	0.00000000
C	13.59247970	-16.29795022	0.00000000
C	13.59248097	16.29795022	0.00000000
C	13.54938724	17.69245477	0.00000000
C	13.50442928	20.51790492	0.00000000
C	13.46181839	21.94490770	0.00000000
C	14.71437060	-19.84009430	0.00000000
C	14.78899759	-18.43517431	0.00000000
C	14.80052729	-15.59451676	0.00000000
C	14.84252033	-14.19543628	0.00000000
C	14.84252147	14.19543635	0.00000000
C	14.80052850	15.59451676	0.00000000
C	14.78899893	18.43517421	0.00000000
C	14.71437201	19.84009419	0.00000000
C	15.99934141	-17.73647472	0.00000000
C	16.04871990	-16.33657823	0.00000000
C	16.04515992	-13.48839509	0.00000000
C	16.08212118	-12.08899235	0.00000000
C	16.08212216	12.08899256	0.00000000
C	16.04516098	13.48839519	0.00000000
C	16.04872110	16.33657816	0.00000000
C	15.99934267	17.73647460	0.00000000
C	17.25635565	-15.62344957	0.00000000
C	17.29853601	-14.22609959	0.00000000
C	17.28172225	-11.37553084	0.00000000
C	17.31188162	-9.97791213	0.00000000
C	17.31188241	9.97791254	0.00000000
C	17.28172312	11.37553111	0.00000000
C	17.29853703	14.22609962	0.00000000
C	17.25635676	15.62344951	0.00000000
C	18.50253272	-13.50634775	0.00000000
C	18.53938675	-12.10814314	0.00000000
C	18.50943643	-9.25687239	0.00000000
C	18.53189271	-7.86129308	0.00000000
C	18.53189329	7.86129374	0.00000000
C	18.50943711	9.25687289	0.00000000
C	18.53938757	12.10814333	0.00000000
C	18.50253362	13.50634782	0.00000000
C	19.73866458	-11.38423277	0.00000000
C	19.77044648	-9.98369485	0.00000000
C	19.72751526	-7.13339133	0.00000000
C	19.74255981	-5.73841384	0.00000000
C	19.74256019	5.73841472	0.00000000
C	19.72751572	7.13339207	0.00000000
C	19.77044708	9.98369527	0.00000000
C	19.73866526	11.38423303	0.00000000
C	20.96514049	-9.25637854	0.00000000
C	20.99091042	-7.85375626	0.00000000
C	20.93518826	-5.00604150	0.00000000
C	20.94386747	-3.60820776	0.00000000
C	20.94386767	3.60820879	0.00000000
C	20.93518852	5.00604243	0.00000000
C	20.99091081	7.85375692	0.00000000
C	20.96514094	9.25637905	0.00000000
C	22.18206062	-7.12189607	0.00000000
C	22.20000269	-5.71935303	0.00000000
C	22.13168311	-2.87674504	0.00000000
C	22.13407368	-1.47046140	0.00000000
C	22.13407373	1.47046251	0.00000000
C	22.13168322	2.87674611	0.00000000
C	22.20000290	5.71935390	0.00000000
C	22.18206089	7.12189681	0.00000000
C	23.38953126	-4.97946744	0.00000000

C	23.39307877	-3.58171774	0.00000000
C	23.29925217	-0.72530036	0.00000000
C	23.29925218	0.72530148	0.00000000
C	23.39307885	3.58171875	0.00000000
C	23.38953137	4.97946836	0.00000000
C	24.57383419	-2.81649163	0.00000000
C	24.55852276	-1.42951378	0.00000000
C	24.55852277	1.42951487	0.00000000
C	24.57383422	2.81649267	0.00000000
C	25.77377155	-0.68024108	0.00000000
C	25.77377155	0.68024216	0.00000000
H	-26.72004199	-1.23866056	0.00000000
H	-26.72004208	1.23865834	0.00000000
H	-25.54296434	-3.33730556	0.00000000
H	-25.54296456	3.33730351	0.00000000
H	-24.34989764	-5.51600877	0.00000000
H	-24.34989791	5.51600697	0.00000000
H	-23.13907225	-7.66445375	0.00000000
H	-23.13907252	7.66445224	0.00000000
H	-21.91952942	-9.80358085	0.00000000
H	-21.91952962	9.80357970	0.00000000
H	-21.15595444	-0.96967639	0.00000000
H	-21.15595444	0.96967455	0.00000000
H	-20.69045206	-11.93596823	0.00000000
H	-20.69045216	11.93596743	0.00000000
H	-19.98434183	-3.06980953	0.00000000
H	-19.98434183	3.06980784	0.00000000
H	-19.45172917	-14.06252096	0.00000000
H	-19.45172917	14.06252045	0.00000000
H	-18.78582295	-5.19531278	0.00000000
H	-18.78582289	5.19531134	0.00000000
H	-18.20296515	-16.18398284	0.00000000
H	-18.20296508	16.18398259	0.00000000
H	-17.57764388	-7.31381954	0.00000000
H	-17.57764374	7.31381841	0.00000000
H	-16.94262714	-18.30254392	0.00000000
H	-16.94262703	18.30254387	0.00000000
H	-16.36009236	-9.42616706	0.00000000
H	-16.36009213	9.42616628	0.00000000
H	-15.64898223	-20.42066545	0.00000000
H	-15.64898212	20.42066555	0.00000000
H	-15.13277526	-11.53307365	0.00000000
H	-15.13277497	11.53307319	0.00000000
H	-14.41809254	-22.48605713	0.00000000
H	-14.41809244	22.48605731	0.00000000
H	-13.89559989	-13.63542065	0.00000000
H	-13.89559958	13.63542049	0.00000000
H	-12.64806274	-15.73353364	0.00000000
H	-12.64806244	15.73353369	0.00000000
H	-12.27092264	-23.72235267	0.00000000
H	-12.27092252	23.72235296	0.00000000
H	-11.40957102	-17.79700150	0.00000000
H	-11.40957079	17.79700169	0.00000000
H	-9.86676444	-23.74967082	0.00000000
H	-9.72675406	-18.76629978	0.00000000
H	-9.72675369	18.76630016	0.00000000
H	-9.86676429	23.74967117	0.00000000
H	-7.38532328	-23.80497710	0.00000000
H	-7.32040697	-18.80099981	0.00000000
H	-7.32040658	18.80100031	0.00000000
H	-7.38532308	23.80497756	0.00000000
H	-4.92079293	-23.83126301	0.00000000
H	-4.87925512	-18.82649204	0.00000000
H	-4.87925470	18.82649262	0.00000000
H	-4.92079263	23.83126355	0.00000000
H	-2.45994155	-23.84667706	0.00000000
H	-2.43926365	-18.84145388	0.00000000
H	-2.43926316	18.84145447	0.00000000
H	-2.45994110	23.84667765	0.00000000
H	-0.00002023	-23.85174977	0.00000000
H	-0.00001197	-18.84648768	0.00000000
H	-0.00001137	18.84648824	0.00000000
H	-0.00001958	23.85175039	0.00000000
H	2.45990176	-23.84668547	0.00000000
H	2.43924015	-18.84146198	0.00000000
H	2.43924091	18.84146246	0.00000000
H	2.45990266	23.84668607	0.00000000
H	4.92075500	-23.83127951	0.00000000
H	4.87923290	-18.82650784	0.00000000
H	4.87923384	18.82650824	0.00000000
H	4.92075616	23.83128005	0.00000000
H	7.38528795	-23.80500127	0.00000000
H	7.32038655	-18.80102286	0.00000000

H	7.32038765	18.80102313	0.00000000
H	7.38528933	23.80500167	0.00000000
H	9.86673200	-23.74970240	0.00000000
H	9.72673512	-18.76632955	0.00000000
H	9.72673631	18.76632972	0.00000000
H	9.86673353	23.74970262	0.00000000
H	11.40955922	-17.79703208	0.00000000
H	11.40956056	17.79703212	0.00000000
H	12.27089309	-23.72239060	0.00000000
H	12.27089471	23.72239064	0.00000000
H	12.64805697	-15.73356415	0.00000000
H	12.64805822	15.73356420	0.00000000
H	13.89560122	-13.63544959	0.00000000
H	13.89560235	13.63544972	0.00000000
H	14.41806840	-22.48609957	0.00000000
H	14.41806996	22.48609947	0.00000000
H	15.13278393	-11.53309986	0.00000000
H	15.13278491	11.53310014	0.00000000
H	15.64896560	-20.42070837	0.00000000
H	15.64896703	20.42070821	0.00000000
H	16.36010822	-9.42618927	0.00000000
H	16.36010903	9.42618975	0.00000000
H	16.94261803	-18.30258620	0.00000000
H	16.94261930	18.30258605	0.00000000
H	17.57766634	-7.31383666	0.00000000
H	17.57766696	7.31383737	0.00000000
H	18.20296413	-16.18402308	0.00000000
H	18.20296522	16.18402299	0.00000000
H	18.78585117	-5.19532440	0.00000000
H	18.78585158	5.19532531	0.00000000
H	19.45173657	-14.06255740	0.00000000
H	19.45173744	14.06255743	0.00000000
H	19.98437498	-3.06981608	0.00000000
H	19.98437521	3.06981713	0.00000000
H	20.69046776	-11.93599913	0.00000000
H	20.69046840	11.93599935	0.00000000
H	21.15599140	-0.96967973	0.00000000
H	21.15599147	0.96968082	0.00000000
H	21.91955280	-9.80360511	0.00000000
H	21.91955321	9.80360556	0.00000000
H	23.13910219	-7.66447123	0.00000000
H	23.13910243	7.66447190	0.00000000
H	24.34993299	-5.51602016	0.00000000
H	24.34993309	5.51602101	0.00000000
H	25.54300435	-3.33731174	0.00000000
H	25.54300437	3.33731272	0.00000000
H	26.72008486	-1.23866118	0.00000000
H	26.72008487	1.23866223	0.00000000

12-HGQR (triplet)

C	-28.23372305	-0.68015806	0.00000000
C	-28.23372347	0.68014645	0.00000000
C	-27.03410550	-2.81603812	0.00000000
C	-27.01830760	-1.42925313	0.00000000
C	-27.01830841	1.42924215	0.00000000
C	-27.03410710	2.81602728	0.00000000
C	-25.85114554	-4.97922560	0.00000000
C	-25.85362906	-3.58159961	0.00000000
C	-25.75877588	-0.72538569	0.00000000
C	-25.75877628	0.72537526	0.00000000
C	-25.85363087	3.58158936	0.00000000
C	-25.85114807	4.97921564	0.00000000
C	-24.64571248	-7.12239134	0.00000000
C	-24.66221483	-5.71971034	0.00000000
C	-24.59185800	-2.87736527	0.00000000
C	-24.59383863	-1.47090297	0.00000000
C	-24.59383932	1.47089310	0.00000000
C	-24.59185932	2.87735547	0.00000000
C	-24.66221738	5.71970100	0.00000000
C	-24.64571563	7.12238247	0.00000000
C	-23.43149120	-9.25811983	0.00000000
C	-23.45559542	-7.85494267	0.00000000
C	-23.39689266	-5.00762265	0.00000000
C	-23.40451008	-3.60975101	0.00000000
C	-23.40451149	3.60974176	0.00000000
C	-23.39689467	5.00761363	0.00000000
C	-23.45559837	7.85493447	0.00000000
C	-23.43149454	9.25811224	0.00000000
C	-22.20842478	-11.38764819	0.00000000
C	-22.23839531	-9.98616403	0.00000000
C	-22.19153149	-7.13628356	0.00000000
C	-22.20484501	-5.74158620	0.00000000
C	-22.20484694	5.74157774	0.00000000
C	-22.19153389	7.13627553	0.00000000

C	-22.23839828	9.98615719	0.00000000
C	-22.20842786	11.38764208	0.00000000
C	-20.97660491	-13.51169684	0.00000000
C	-21.01134187	-12.11259817	0.00000000
C	-20.97645125	-9.26146967	0.00000000
C	-20.99652652	-7.86661704	0.00000000
C	-20.99652870	7.86660963	0.00000000
C	-20.97645371	9.26146287	0.00000000
C	-21.01134448	12.11259286	0.00000000
C	-20.97660735	13.51169231	0.00000000
C	-19.73597070	-15.63075367	0.00000000
C	-19.77510601	-14.23362338	0.00000000
C	-19.75222016	-11.38237623	0.00000000
C	-19.77959571	-9.98581720	0.00000000
C	-19.77959785	9.98581112	0.00000000
C	-19.75222234	11.38237089	0.00000000
C	-19.77510798	14.23361966	0.00000000
C	-19.73597229	15.63075071	0.00000000
C	-18.48617983	-17.74529504	0.00000000
C	-18.53022467	-16.34854601	0.00000000
C	-18.51930951	-13.49829735	0.00000000
C	-18.55367345	-12.09964108	0.00000000
C	-18.55367528	12.09963654	0.00000000
C	-18.51931116	13.49829358	0.00000000
C	-18.53022585	16.34854382	0.00000000
C	-18.48618052	17.74529355	0.00000000
C	-17.22648122	-19.85617487	0.00000000
C	-17.27732781	-18.45654725	0.00000000
C	-17.27842928	-15.60866790	0.00000000
C	-17.31868825	-14.20859410	0.00000000
C	-17.31868956	14.20859117	0.00000000
C	-17.27843025	15.60866566	0.00000000
C	-17.27732817	18.45654644	0.00000000
C	-17.22648109	19.85617464	0.00000000
C	-15.93979215	-21.95807997	0.00000000
C	-16.01539497	-20.55328760	0.00000000
C	-16.03032521	-17.71278928	0.00000000
C	-16.07476328	-16.31342314	0.00000000
C	-16.07476393	16.31342169	0.00000000
C	-16.03032545	17.71278842	0.00000000
C	-16.01539456	20.55328792	0.00000000
C	-15.93979135	21.95808066	0.00000000
C	-14.68672987	-24.06191893	0.00000000
C	-14.72952352	-22.63488379	0.00000000
C	-14.77656539	-19.80932160	0.00000000
C	-14.82136212	-18.41476328	0.00000000
C	-14.82136209	18.41476308	0.00000000
C	-14.77656494	19.80932190	0.00000000
C	-14.72952242	22.63488490	0.00000000
C	-14.68672872	24.06192027	0.00000000
C	-13.50785642	-24.74021697	0.00000000
C	-13.49164428	-21.89324993	0.00000000
C	-13.55776262	-20.51149619	0.00000000
C	-13.55776194	20.51149693	0.00000000
C	-13.49164293	21.89325120	0.00000000
C	-13.50785484	24.74021867	0.00000000
C	-12.25279073	-24.05984419	0.00000000
C	-12.23416454	-22.61681584	0.00000000
C	-12.23416281	22.61681744	0.00000000
C	-12.25278891	24.05984594	0.00000000
C	-11.05930595	-24.76580008	0.00000000
C	-11.00644455	-21.97962315	0.00000000
C	-11.00644207	21.97962516	0.00000000
C	-11.05930379	24.76580202	0.00000000
C	-9.80687201	-24.12511282	0.00000000
C	-9.78668819	-22.68009604	0.00000000
C	-9.78668545	22.68009821	0.00000000
C	-9.80686942	24.12511500	0.00000000
C	-8.59549956	-24.82160472	0.00000000
C	-8.55865129	-22.01775396	0.00000000
C	-8.55864805	22.01775631	0.00000000
C	-8.59549664	24.82160704	0.00000000
C	-7.36010500	-24.16195864	0.00000000
C	-7.34401823	-22.70993790	0.00000000
C	-7.34401463	22.70994035	0.00000000
C	-7.36010156	24.16196112	0.00000000
C	-6.13777813	-24.84958596	0.00000000
C	-6.11183581	-22.04509263	0.00000000
C	-6.11183176	22.04509517	0.00000000
C	-6.13777431	24.84958857	0.00000000
C	-4.90824295	-24.18571897	0.00000000
C	-4.89752570	-22.73149847	0.00000000
C	-4.89752126	22.73150111	0.00000000

C	-4.90823861	24.18572165	0.00000000
C	-3.68208898	-24.86777345	0.00000000
C	-3.66658776	-22.06306217	0.00000000
C	-3.66658289	22.06306483	0.00000000
C	-3.68208423	24.86777624	0.00000000
C	-2.45454066	-24.19985430	0.00000000
C	-2.44919014	-22.74445112	0.00000000
C	-2.44918488	22.74445386	0.00000000
C	-2.45453543	24.19985707	0.00000000
C	-1.22727859	-24.87670765	0.00000000
C	-1.22212056	-22.07215818	0.00000000
C	-1.22211491	22.07216089	0.00000000
C	-1.22727293	24.87671048	0.00000000
C	0.00000136	-24.20452865	0.00000000
C	0.00000122	-22.74880286	0.00000000
C	0.00000724	22.74880560	0.00000000
C	0.00000742	24.20453139	0.00000000
C	1.22728126	-24.87670725	0.00000000
C	1.22212270	-22.07215811	0.00000000
C	1.22212900	22.07216078	0.00000000
C	1.22728773	24.87670996	0.00000000
C	2.45454294	-24.19985364	0.00000000
C	2.44919214	-22.74445085	0.00000000
C	2.44919875	22.74445346	0.00000000
C	2.45454966	24.19985619	0.00000000
C	3.68209081	-24.86777232	0.00000000
C	3.66658910	-22.06306193	0.00000000
C	3.66659585	22.06306444	0.00000000
C	3.68209788	24.86777473	0.00000000
C	4.90824405	-24.18571775	0.00000000
C	4.89752655	-22.73149790	0.00000000
C	4.89753353	22.73150025	0.00000000
C	4.90825124	24.18571998	0.00000000
C	6.13777859	-24.84958429	0.00000000
C	6.11183582	-22.04509211	0.00000000
C	6.11184281	22.04509434	0.00000000
C	6.13778606	24.84958626	0.00000000
C	7.36010460	-24.16195699	0.00000000
C	7.34401760	-22.70993696	0.00000000
C	7.34402475	22.70993892	0.00000000
C	7.36011204	24.16195876	0.00000000
C	8.59549858	-24.82160265	0.00000000
C	8.55864986	-22.01775304	0.00000000
C	8.55865692	22.01775487	0.00000000
C	8.59550629	24.82160402	0.00000000
C	9.80687017	-24.12511075	0.00000000
C	9.78668616	-22.68009464	0.00000000
C	9.78669338	22.68009602	0.00000000
C	9.80687772	24.12511186	0.00000000
C	11.05930372	-24.76579753	0.00000000
C	11.00644181	-21.97962162	0.00000000
C	11.00644877	21.97962279	0.00000000
C	11.05931146	24.76579803	0.00000000
C	12.25278788	-24.05984179	0.00000000
C	12.23416123	-22.61681395	0.00000000
C	12.23416849	22.61681441	0.00000000
C	12.25279540	24.05984205	0.00000000
C	13.50785308	-24.74021454	0.00000000
C	13.49164061	-21.89324816	0.00000000
C	13.55775877	-20.51149502	0.00000000
C	13.55776638	20.51149535	0.00000000
C	13.49164785	21.89324833	0.00000000
C	13.50786050	24.74021454	0.00000000
C	14.68672613	-24.06191670	0.00000000
C	14.72951939	-22.63488200	0.00000000
C	14.77656125	-19.80932086	0.00000000
C	14.82135777	-18.41476309	0.00000000
C	14.82136540	18.41476384	0.00000000
C	14.77656865	19.80932126	0.00000000
C	14.72952642	22.63488192	0.00000000
C	14.68673345	24.06191643	0.00000000
C	15.93978765	-21.95807854	0.00000000
C	16.01539047	-20.55328660	0.00000000
C	16.03032052	-17.71278946	0.00000000
C	16.07475828	-16.31342375	0.00000000
C	16.07476564	16.31342495	0.00000000
C	16.03032777	17.71279020	0.00000000
C	16.01539742	20.55328668	0.00000000
C	15.93979436	21.95807834	0.00000000
C	17.22647644	-19.85617438	0.00000000
C	17.27732284	-18.45654719	0.00000000
C	17.27842398	-15.60866877	0.00000000
C	17.31868263	-14.20859513	0.00000000

C	17.31868950	14.20859700	0.00000000
C	17.27843084	15.60867009	0.00000000
C	17.27732957	18.45654760	0.00000000
C	17.22648298	19.85617447	0.00000000
C	18.48617461	-17.74529537	0.00000000
C	18.53021922	-16.34854669	0.00000000
C	18.51930376	-13.49829840	0.00000000
C	18.55366746	-12.09964185	0.00000000
C	18.55367351	12.09964457	0.00000000
C	18.51931001	13.49830050	0.00000000
C	18.53022553	16.34854761	0.00000000
C	18.48618083	17.74529581	0.00000000
C	19.73596524	-15.63075456	0.00000000
C	19.77510038	-14.23362437	0.00000000
C	19.75221431	-11.38237666	0.00000000
C	19.77958978	-9.98581689	0.00000000
C	19.77959478	9.98582061	0.00000000
C	19.75221973	11.38237974	0.00000000
C	19.77510610	14.23362607	0.00000000
C	19.73597097	15.63075563	0.00000000
C	20.97659958	-13.51169772	0.00000000
C	21.01133650	-12.11259875	0.00000000
C	20.97644576	-9.26146866	0.00000000
C	20.99652108	-7.86661497	0.00000000
C	20.99652489	7.86661972	0.00000000
C	20.97645018	9.26147279	0.00000000
C	21.01134146	12.11260141	0.00000000
C	20.97660473	13.51169970	0.00000000
C	22.20842003	-11.38764825	0.00000000
C	22.23839063	-9.98616334	0.00000000
C	22.19152672	-7.13628057	0.00000000
C	22.20484037	-5.74158207	0.00000000
C	22.20484297	5.74158773	0.00000000
C	22.19153002	7.13628569	0.00000000
C	22.23839471	9.98616705	0.00000000
C	22.20842448	11.38765129	0.00000000
C	23.43148732	-9.25811829	0.00000000
C	23.45559164	-7.85494008	0.00000000
C	23.39688877	-5.00761757	0.00000000
C	23.40450633	-3.60974498	0.00000000
C	23.40450782	3.60975125	0.00000000
C	23.39689094	5.00762347	0.00000000
C	23.45559476	7.85494477	0.00000000
C	23.43149095	9.25812242	0.00000000
C	24.64570950	-7.12238779	0.00000000
C	24.66221193	-5.71970566	0.00000000
C	24.59185500	-2.87735849	0.00000000
C	24.59383583	-1.47089549	0.00000000
C	24.59383637	1.47090199	0.00000000
C	24.59185612	2.87736480	0.00000000
C	24.66221408	5.71971113	0.00000000
C	24.64571222	7.12239282	0.00000000
C	25.85114330	-4.97922014	0.00000000
C	25.85362692	-3.58159315	0.00000000
C	25.75877369	-0.72537749	0.00000000
C	25.75877394	0.72538384	0.00000000
C	25.85362815	3.58159904	0.00000000
C	25.85114510	4.97922573	0.00000000
C	27.03410395	-2.81603121	0.00000000
C	27.01830587	-1.42924545	0.00000000
C	27.01830634	1.42925140	0.00000000
C	27.03410487	2.81603700	0.00000000
C	28.23372152	-0.68014961	0.00000000
C	28.23372173	0.68015530	0.00000000
H	-29.17986123	-1.23881430	0.00000000
H	-29.17986196	1.23880224	0.00000000
H	-28.00341129	-3.33657304	0.00000000
H	-28.00341317	3.33656184	0.00000000
H	-26.81189585	-5.51511948	0.00000000
H	-26.81189868	5.51510927	0.00000000
H	-25.60330897	-7.66396243	0.00000000
H	-25.60331243	7.66395339	0.00000000
H	-24.38666628	-9.80400363	0.00000000
H	-24.38666997	9.80399592	0.00000000
H	-23.61564644	-0.97032708	0.00000000
H	-23.61564685	0.97031774	0.00000000
H	-23.16121640	-11.93771512	0.00000000
H	-23.16121982	11.93770891	0.00000000
H	-22.44463104	-3.07207357	0.00000000
H	-22.44463209	3.07206475	0.00000000
H	-21.92703649	-14.06583076	0.00000000
H	-21.92703923	14.06582617	0.00000000
H	-21.24755710	-5.19953720	0.00000000

H	-21.24755866	5.19952905	0.00000000
H	-20.68404149	-16.18888233	0.00000000
H	-20.68404331	16.18887937	0.00000000
H	-20.04153026	-7.32051061	0.00000000
H	-20.04153212	7.32050336	0.00000000
H	-19.43188725	-18.30736458	0.00000000
H	-19.43188808	18.30736312	0.00000000
H	-18.82687128	-9.43572580	0.00000000
H	-18.82687316	9.43571973	0.00000000
H	-18.16909371	-20.42335985	0.00000000
H	-18.16909365	20.42335966	0.00000000
H	-17.60323106	-11.54561525	0.00000000
H	-17.60323269	11.54561064	0.00000000
H	-16.87398663	-22.53930951	0.00000000
H	-16.87398594	22.53931016	0.00000000
H	-16.37048607	-13.65077174	0.00000000
H	-16.37048724	13.65076872	0.00000000
H	-15.64293775	-24.60312492	0.00000000
H	-15.64293678	24.60312605	0.00000000
H	-15.12876319	-15.75190096	0.00000000
H	-15.12876375	15.75189943	0.00000000
H	-13.87763293	-17.84923932	0.00000000
H	-13.87763287	17.84923904	0.00000000
H	-13.49506618	-25.83887686	0.00000000
H	-13.49506450	25.83887859	0.00000000
H	-12.63643130	-19.91261704	0.00000000
H	-12.63643087	19.91261740	0.00000000
H	-11.09218699	-25.86553670	0.00000000
H	-10.95188716	-20.88215925	0.00000000
H	-10.95188422	20.88216131	0.00000000
H	-11.09218495	25.86553865	0.00000000
H	-8.61185328	-25.92156249	0.00000000
H	-8.54423409	-20.91766585	0.00000000
H	-8.54423076	20.91766821	0.00000000
H	-8.61185053	25.92156479	0.00000000
H	-6.14830047	-25.94964316	0.00000000
H	-6.10191127	-20.94505534	0.00000000
H	-6.10190719	20.94505792	0.00000000
H	-6.14829677	25.94964573	0.00000000
H	-3.68833891	-25.96788902	0.00000000
H	-3.66064731	-20.96298293	0.00000000
H	-3.66064244	20.96298566	0.00000000
H	-3.68833424	25.96789174	0.00000000
H	-1.22934863	-25.97685565	0.00000000
H	-1.22012901	-20.97204997	0.00000000
H	-1.22012341	20.97205279	0.00000000
H	-1.22934299	25.97685839	0.00000000
H	1.22935104	-25.97685510	0.00000000
H	1.22013047	-20.97205006	0.00000000
H	1.22013665	20.97205287	0.00000000
H	1.22935754	25.97685768	0.00000000
H	3.68834065	-25.96788749	0.00000000
H	3.66064814	-20.96298310	0.00000000
H	3.66065473	20.96298580	0.00000000
H	3.68834782	25.96788974	0.00000000
H	6.14830104	-25.94964097	0.00000000
H	6.10191102	-20.94505536	0.00000000
H	6.10191781	20.94505782	0.00000000
H	6.14830870	25.94964276	0.00000000
H	8.61185263	-25.92155990	0.00000000
H	8.54423252	-20.91766549	0.00000000
H	8.54423929	20.91766759	0.00000000
H	8.61186064	25.92156106	0.00000000
H	11.09218502	-25.86553372	0.00000000
H	10.95188437	-20.88215827	0.00000000
H	10.95189068	20.88215980	0.00000000
H	11.09219302	25.86553402	0.00000000
H	12.63642790	-19.91261612	0.00000000
H	12.63643591	19.91261649	0.00000000
H	13.49506276	-25.83887406	0.00000000
H	13.49507006	25.83887387	0.00000000
H	13.87762900	-17.84923931	0.00000000
H	13.87763691	17.84924037	0.00000000
H	15.12875857	-15.75190162	0.00000000
H	15.12876627	15.75190318	0.00000000
H	15.64293385	-24.60312230	0.00000000
H	15.64294117	24.60312153	0.00000000
H	16.37048076	-13.65077256	0.00000000
H	16.37048803	13.65077474	0.00000000
H	16.87398182	-22.53930794	0.00000000
H	16.87398826	22.53930758	0.00000000
H	17.60322517	-11.54561557	0.00000000
H	17.60323165	11.54561854	0.00000000

H	18.16908859	-20.42335920	0.00000000
H	18.16909470	20.42335923	0.00000000
H	18.82686512	-9.43572492	0.00000000
H	18.82687052	9.43572885	0.00000000
H	19.43188189	-18.30736456	0.00000000
H	19.43188767	18.30736483	0.00000000
H	20.04152428	-7.32050803	0.00000000
H	20.04152838	7.32051300	0.00000000
H	20.68403615	-16.18888278	0.00000000
H	20.68404146	16.18888357	0.00000000
H	21.24755170	-5.19953275	0.00000000
H	21.24755446	5.19953862	0.00000000
H	21.92703161	-14.06583121	0.00000000
H	21.92703640	14.06583279	0.00000000
H	22.44462651	-3.07206730	0.00000000
H	22.44462805	3.07207378	0.00000000
H	23.16121235	-11.93771493	0.00000000
H	23.16121652	11.93771754	0.00000000
H	23.61564288	-0.97031957	0.00000000
H	23.61564341	0.97032618	0.00000000
H	24.38666322	-9.80400214	0.00000000
H	24.38666666	9.80400581	0.00000000
H	25.60330675	-7.66395917	0.00000000
H	25.60330938	7.66396377	0.00000000
H	26.81189419	-5.51511453	0.00000000
H	26.81189601	5.51511968	0.00000000
H	28.00341024	-3.33656645	0.00000000
H	28.00341125	3.33657186	0.00000000
H	29.17986033	-1.23880575	0.00000000
H	29.17986072	1.23881107	0.00000000

13-HGQR (triplet)

C	-30.69565596	-0.68010721	0.00000000
C	-30.69565681	0.68006099	0.00000000
C	-29.49638371	-2.81569843	0.00000000
C	-29.48014375	-1.42911842	0.00000000
C	-29.48014560	1.42907391	0.00000000
C	-29.49638674	2.81565455	0.00000000
C	-28.31456376	-4.97902478	0.00000000
C	-28.31608795	-3.58161011	0.00000000
C	-28.22034806	-0.72551243	0.00000000
C	-28.22034892	0.72546944	0.00000000
C	-28.31609143	3.58156828	0.00000000
C	-28.31456786	4.97898406	0.00000000
C	-27.11085888	-7.12275432	0.00000000
C	-27.12603696	-5.72017210	0.00000000
C	-27.05396796	-2.87796420	0.00000000
C	-27.05564428	-1.47128409	0.00000000
C	-27.05564577	1.47124283	0.00000000
C	-27.05397061	2.87792361	0.00000000
C	-27.12604103	5.72013423	0.00000000
C	-27.11086294	7.12271819	0.00000000
C	-25.89887810	-9.25945325	0.00000000
C	-25.92140212	-7.85610019	0.00000000
C	-25.86023824	-5.00904872	0.00000000
C	-25.86705926	-3.61098692	0.00000000
C	-25.86706219	3.61094874	0.00000000
C	-25.86024162	5.00901200	0.00000000
C	-25.92140562	7.85606777	0.00000000
C	-25.89888098	9.25942336	0.00000000
C	-24.67855145	-11.39035825	0.00000000
C	-24.70682231	-9.98827948	0.00000000
C	-24.65676243	-7.13881011	0.00000000
C	-24.66876477	-5.74407276	0.00000000
C	-24.66876799	5.74403906	0.00000000
C	-24.65676530	7.13877878	0.00000000
C	-24.70682419	9.98825415	0.00000000
C	-24.67855216	11.39033623	0.00000000
C	-23.45003418	-13.51611783	0.00000000
C	-23.48302593	-12.11603876	0.00000000
C	-23.44419573	-9.26535418	0.00000000
C	-23.46237642	-7.87078118	0.00000000
C	-23.46237867	7.87075369	0.00000000
C	-23.44419695	9.26532988	0.00000000
C	-23.48302533	12.11602184	0.00000000
C	-23.45003208	13.51610466	0.00000000
C	-22.21345818	-15.63705679	0.00000000
C	-22.25063854	-14.23900124	0.00000000
C	-22.22301301	-11.38792200	0.00000000
C	-22.24793929	-9.99209213	0.00000000
C	-22.24793945	9.99207248	0.00000000
C	-22.22301172	11.38790605	0.00000000
C	-22.25063507	14.23899320	0.00000000
C	-22.21345317	15.63705242	0.00000000

C	-20.96880873	-17.75341907	0.00000000
C	-21.01006515	-16.35685616	0.00000000
C	-20.99347946	-13.50595389	0.00000000
C	-21.02509298	-12.10836285	0.00000000
C	-21.02509038	12.10835202	0.00000000
C	-20.99347535	13.50594680	0.00000000
C	-21.01005901	16.35685640	0.00000000
C	-20.96880130	17.75342248	0.00000000
C	-19.71577241	-19.86559338	0.00000000
C	-19.76165218	-18.46907475	0.00000000
C	-19.75598981	-15.61915559	0.00000000
C	-19.79378868	-14.21983698	0.00000000
C	-19.79378330	14.21983493	0.00000000
C	-19.75598315	15.61915680	0.00000000
C	-19.76164395	18.46908189	0.00000000
C	-19.71576324	19.86560303	0.00000000
C	-18.45360285	-21.97444608	0.00000000
C	-18.50593564	-20.57489149	0.00000000
C	-18.51122954	-17.72721559	0.00000000
C	-18.55417150	-16.32687171	0.00000000
C	-18.55416382	16.32687729	0.00000000
C	-18.51122092	17.72722365	0.00000000
C	-18.50592587	20.57490394	0.00000000
C	-18.45359236	21.97446041	0.00000000
C	-17.16527870	-24.07471704	0.00000000
C	-17.24187345	-22.66996447	0.00000000
C	-17.25994945	-19.82953915	0.00000000
C	-17.30643831	-18.43014976	0.00000000
C	-17.30642905	18.43016123	0.00000000
C	-17.25993946	19.82955228	0.00000000
C	-17.24186239	22.66998072	0.00000000
C	-17.16526699	24.07473450	0.00000000
C	-15.91155309	-26.17755127	0.00000000
C	-15.95469246	-24.75049972	0.00000000
C	-16.00374286	-21.92479705	0.00000000
C	-16.05004314	-20.53030580	0.00000000
C	-16.05003277	20.53032126	0.00000000
C	-16.00373172	21.92481360	0.00000000
C	-15.95468014	24.75051867	0.00000000
C	-15.91154051	26.17757040	0.00000000
C	-14.73256662	-26.85534033	0.00000000
C	-14.71721525	-24.00797202	0.00000000
C	-14.78427768	-22.62630461	0.00000000
C	-14.78426624	22.62632249	0.00000000
C	-14.71720311	24.00799063	0.00000000
C	-14.73255285	26.85535955	0.00000000
C	-13.47780877	-26.17434021	0.00000000
C	-13.45926317	-24.73119621	0.00000000
C	-13.45925051	24.73121473	0.00000000
C	-13.47779555	26.17435848	0.00000000
C	-12.28452961	-26.88021008	0.00000000
C	-12.23160709	-24.09396545	0.00000000
C	-12.23159454	24.09398372	0.00000000
C	-12.28451566	26.88022730	0.00000000
C	-11.03209668	-26.23948368	0.00000000
C	-11.01158193	-24.79445356	0.00000000
C	-11.01156897	24.79447100	0.00000000
C	-11.03208306	26.23950048	0.00000000
C	-9.82126694	-26.93644471	0.00000000
C	-9.78332572	-24.13254933	0.00000000
C	-9.78331335	24.13256669	0.00000000
C	-9.82125334	26.93646078	0.00000000
C	-8.58566525	-26.27721826	0.00000000
C	-8.56875459	-24.82526524	0.00000000
C	-8.56874223	24.82528163	0.00000000
C	-8.58565239	26.27723394	0.00000000
C	-7.36402823	-26.96578445	0.00000000
C	-7.33599653	-24.16128576	0.00000000
C	-7.33598495	24.16130225	0.00000000
C	-7.36401575	26.96579962	0.00000000
C	-6.13424849	-26.30272531	0.00000000
C	-6.12219206	-24.84869148	0.00000000
C	-6.12218090	24.84870716	0.00000000
C	-6.13423701	26.30274027	0.00000000
C	-4.90873887	-26.98618197	0.00000000
C	-4.89014171	-24.18155339	0.00000000
C	-4.89013135	24.18156938	0.00000000
C	-4.90872807	26.98619664	0.00000000
C	-3.68109258	-26.31949832	0.00000000
C	-3.67387632	-24.86439038	0.00000000
C	-3.67386678	24.86440572	0.00000000
C	-3.68108289	26.31951294	0.00000000
C	-2.45424208	-26.99818176	0.00000000

C	-2.44496719	-24.19386998	0.00000000
C	-2.44495862	24.19388576	0.00000000
C	-2.45423334	26.99819625	0.00000000
C	-1.22710849	-26.32777438	0.00000000
C	-1.22470618	-24.87233075	0.00000000
C	-1.22469871	24.87234601	0.00000000
C	-1.22710096	26.32778884	0.00000000
C	-0.00000553	-27.00214369	0.00000000
C	-0.00000590	-24.19800284	0.00000000
C	0.00000040	24.19801860	0.00000000
C	0.00000082	27.00215807	0.00000000
C	1.22709739	-26.32777406	0.00000000
C	1.22469470	-24.87233043	0.00000000
C	1.22469980	24.87234568	0.00000000
C	1.22710250	26.32778846	0.00000000
C	2.45423137	-26.99818113	0.00000000
C	2.44495578	-24.19386935	0.00000000
C	2.44495962	24.19388515	0.00000000
C	2.45423516	26.99819545	0.00000000
C	3.68108213	-26.31949741	0.00000000
C	3.67386553	-24.86438942	0.00000000
C	3.67386823	24.86440472	0.00000000
C	3.68108476	26.31951183	0.00000000
C	4.90872911	-26.98618075	0.00000000
C	4.89013132	-24.18155218	0.00000000
C	4.89013290	24.18156814	0.00000000
C	4.90873036	26.98619509	0.00000000
C	6.13423918	-26.30272385	0.00000000
C	6.12218247	-24.84868998	0.00000000
C	6.12218294	24.84870553	0.00000000
C	6.13423943	26.30273848	0.00000000
C	7.36401973	-26.96578275	0.00000000
C	7.33598754	-24.16128402	0.00000000
C	7.33598719	24.16130043	0.00000000
C	7.36401865	26.96579743	0.00000000
C	8.58565728	-26.27721638	0.00000000
C	8.56874640	-24.82526327	0.00000000
C	8.56874493	24.82527943	0.00000000
C	8.58565540	26.27723156	0.00000000
C	9.82125974	-26.93644264	0.00000000
C	9.78331818	-24.13254718	0.00000000
C	9.78331624	24.13256432	0.00000000
C	9.82125681	26.93645803	0.00000000
C	11.03209003	-26.23948149	0.00000000
C	11.01157518	-24.79445125	0.00000000
C	11.01157227	24.79446826	0.00000000
C	11.03208660	26.23949756	0.00000000
C	12.28452359	-26.88020779	0.00000000
C	12.23160092	-24.09396296	0.00000000
C	12.23159795	24.09398079	0.00000000
C	12.28451954	26.88022399	0.00000000
C	13.47780337	-26.17433778	0.00000000
C	13.45925783	-24.73119353	0.00000000
C	13.45925431	24.73121139	0.00000000
C	13.47779955	26.17435507	0.00000000
C	14.73256150	-26.85533801	0.00000000
C	14.71721034	-24.00796904	0.00000000
C	14.78427324	-22.62630095	0.00000000
C	14.78427048	22.62631889	0.00000000
C	14.71720703	24.00798710	0.00000000
C	14.73255682	26.85535611	0.00000000
C	15.91154854	-26.17754857	0.00000000
C	15.95468787	-24.75049671	0.00000000
C	16.00373884	-21.92479281	0.00000000
C	16.05003952	-20.53030083	0.00000000
C	16.05003738	20.53031759	0.00000000
C	16.00373603	21.92480993	0.00000000
C	15.95468397	24.75051508	0.00000000
C	15.91154458	26.17756676	0.00000000
C	17.16527457	-24.07471347	0.00000000
C	17.24186964	-22.66996032	0.00000000
C	17.25994636	-19.82953348	0.00000000
C	17.30643570	-18.43014329	0.00000000
C	17.30643397	18.43015759	0.00000000
C	17.25994409	19.82954857	0.00000000
C	17.24186646	22.66997695	0.00000000
C	17.16527079	24.07473074	0.00000000
C	18.45359948	-21.97444136	0.00000000
C	18.50593273	-20.57488602	0.00000000
C	18.51122754	-17.72720840	0.00000000
C	18.55417002	-16.32686365	0.00000000
C	18.55416896	16.32687394	0.00000000
C	18.51122579	17.72722011	0.00000000

C	18.50593016	20.57490017	0.00000000
C	18.45359638	21.97445664	0.00000000
C	19.71577006	-19.86558731	0.00000000
C	19.76165038	-18.46906783	0.00000000
C	19.75598897	-15.61914692	0.00000000
C	19.79378832	-14.21982748	0.00000000
C	19.79378843	14.21983227	0.00000000
C	19.75598812	15.61915378	0.00000000
C	19.76164838	18.46907831	0.00000000
C	19.71576743	19.86559931	0.00000000
C	20.96880758	-17.75341156	0.00000000
C	21.01006453	-16.35684783	0.00000000
C	20.99347967	-13.50594399	0.00000000
C	21.02509353	-12.10835229	0.00000000
C	21.02509521	12.10835059	0.00000000
C	20.99348018	13.50594477	0.00000000
C	21.01006350	16.35685335	0.00000000
C	20.96880561	17.75341909	0.00000000
C	22.21345822	-15.63704794	0.00000000
C	22.25063901	-14.23899172	0.00000000
C	22.22301402	-11.38791133	0.00000000
C	22.24794047	-9.99208105	0.00000000
C	22.24794364	9.99207276	0.00000000
C	22.22301612	11.38790552	0.00000000
C	22.25063941	14.23899117	0.00000000
C	22.21345746	15.63704979	0.00000000
C	23.45003525	-13.51610799	0.00000000
C	23.48302722	-12.11602847	0.00000000
C	23.44419721	-9.26534325	0.00000000
C	23.46237789	-7.87077012	0.00000000
C	23.46238201	7.87075597	0.00000000
C	23.44420065	9.26533121	0.00000000
C	23.48302926	12.11602132	0.00000000
C	23.45003612	13.51610331	0.00000000
C	24.67855320	-11.39034789	0.00000000
C	24.70682409	-9.98826889	0.00000000
C	24.65676408	-7.13879936	0.00000000
C	24.66876625	-5.74406213	0.00000000
C	24.66877043	5.74404333	0.00000000
C	24.65676816	7.13878212	0.00000000
C	24.70682750	9.98825551	0.00000000
C	24.67855571	11.39033664	0.00000000
C	25.89888015	-9.25944283	0.00000000
C	25.92140403	-7.85608972	0.00000000
C	25.86023981	-5.00903845	0.00000000
C	25.86706060	-3.61097692	0.00000000
C	25.86706386	3.61095475	0.00000000
C	25.86024366	5.00901722	0.00000000
C	25.92140819	7.85607114	0.00000000
C	25.89888386	9.25942580	0.00000000
C	27.11086091	-7.12274417	0.00000000
C	27.12603875	-5.72016202	0.00000000
C	27.05396929	-2.87795463	0.00000000
C	27.05564544	-1.47127485	0.00000000
C	27.05564690	1.47125021	0.00000000
C	27.05397197	2.87793043	0.00000000
C	27.12604287	5.72013948	0.00000000
C	27.11086510	7.12272264	0.00000000
C	28.31456554	-4.97901510	0.00000000
C	28.31608945	-3.58160059	0.00000000
C	28.22034915	-0.72550378	0.00000000
C	28.22034993	0.72547766	0.00000000
C	28.31609270	3.58157511	0.00000000
C	28.31456937	4.97899024	0.00000000
C	29.49638517	-2.81568931	0.00000000
C	29.48014486	-1.42910963	0.00000000
C	29.48014651	1.42908204	0.00000000
C	29.49638783	2.81566209	0.00000000
C	30.69565688	-0.68009836	0.00000000
C	30.69565759	0.68006927	0.00000000
H	-31.64174736	-1.23887227	0.00000000
H	-31.64174899	1.23882450	0.00000000
H	-30.46581749	-3.33600910	0.00000000
H	-30.46582100	3.33596376	0.00000000
H	-29.27563773	-5.51435321	0.00000000
H	-29.27564221	5.51431076	0.00000000
H	-28.06892067	-7.66351529	0.00000000
H	-28.06892491	7.66347727	0.00000000
H	-26.85467133	-9.80427230	0.00000000
H	-26.85467416	9.80424048	0.00000000
H	-26.07734679	-0.97086131	0.00000000
H	-26.07734819	0.97082049	0.00000000
H	-25.63212855	-11.93909212	0.00000000

H	-25.63212893	11.93906834	0.00000000
H	-24.90685321	-3.07387466	0.00000000
H	-24.90685624	3.07383705	0.00000000
H	-24.40143853	-14.06862734	0.00000000
H	-24.40143583	14.06861271	0.00000000
H	-23.71098860	-5.20286667	0.00000000
H	-23.71099229	5.20283342	0.00000000
H	-23.16270329	-16.19324877	0.00000000
H	-23.16269748	16.19324329	0.00000000
H	-22.50673499	-7.32578029	0.00000000
H	-22.50673792	7.32575338	0.00000000
H	-21.91588495	-18.31324994	0.00000000
H	-21.91587661	18.31325256	0.00000000
H	-21.29441823	-9.44334801	0.00000000
H	-21.29441912	9.44332925	0.00000000
H	-20.66067315	-20.42902918	0.00000000
H	-20.66066308	20.42903831	0.00000000
H	-20.07371585	-11.55589233	0.00000000
H	-20.07371387	11.55588278	0.00000000
H	-19.39559443	-22.54267471	0.00000000
H	-19.39558311	22.54268875	0.00000000
H	-18.84453933	-13.66373470	0.00000000
H	-18.84453441	13.66373424	0.00000000
H	-18.09909964	-24.65655699	0.00000000
H	-18.09908752	24.65657380	0.00000000
H	-17.60698492	-15.76730251	0.00000000
H	-17.60697760	15.76730980	0.00000000
H	-16.86765628	-26.71897031	0.00000000
H	-16.86764340	26.71898866	0.00000000
H	-16.36126148	-17.86722286	0.00000000
H	-16.36125257	17.86723586	0.00000000
H	-15.10693345	-19.96373033	0.00000000
H	-15.10692358	19.96374686	0.00000000
H	-14.71921322	-27.95400755	0.00000000
H	-14.71919784	27.95402615	0.00000000
H	-13.86326938	-22.02688008	0.00000000
H	-13.86325844	22.02689881	0.00000000
H	-12.31732824	-27.97995551	0.00000000
H	-12.17721640	-22.99646657	0.00000000
H	-12.17720450	22.99648562	0.00000000
H	-12.31731326	27.97997217	0.00000000
H	-9.83803949	-28.03640317	0.00000000
H	-9.76848841	-23.03245660	0.00000000
H	-9.76847657	23.03247485	0.00000000
H	-9.83802532	28.03641867	0.00000000
H	-7.37534456	-28.06583964	0.00000000
H	-7.32523751	-23.06124566	0.00000000
H	-7.32522622	23.06126310	0.00000000
H	-7.37533162	28.06585426	0.00000000
H	-4.91617602	-28.08629828	0.00000000
H	-4.88296501	-23.08146889	0.00000000
H	-4.88295475	23.08148584	0.00000000
H	-4.91616495	28.08631245	0.00000000
H	-2.45792006	-28.09833936	0.00000000
H	-2.44134930	-23.09374795	0.00000000
H	-2.44134076	23.09376472	0.00000000
H	-2.45791123	28.09835338	0.00000000
H	-0.00000549	-28.10231626	0.00000000
H	-0.00000615	-23.09786645	0.00000000
H	0.00000010	23.09788322	0.00000000
H	0.00000082	28.10233018	0.00000000
H	2.45790947	-28.09833867	0.00000000
H	2.44133745	-23.09374737	0.00000000
H	2.44134121	23.09376423	0.00000000
H	2.45791313	28.09835246	0.00000000
H	4.91616643	-28.08629697	0.00000000
H	4.88295432	-23.08146776	0.00000000
H	4.88295588	23.08148484	0.00000000
H	4.91616741	28.08631069	0.00000000
H	7.37533627	-28.06583783	0.00000000
H	7.32522835	-23.06124401	0.00000000
H	7.32522820	23.06126157	0.00000000
H	7.37533476	28.06585181	0.00000000
H	9.83803251	-28.03640102	0.00000000
H	9.76848075	-23.03245453	0.00000000
H	9.76847923	23.03247279	0.00000000
H	9.83802909	28.03641565	0.00000000
H	12.31732225	-27.97995318	0.00000000
H	12.17720993	-22.99646417	0.00000000
H	12.17720751	22.99648305	0.00000000
H	12.31731728	27.97996864	0.00000000
H	13.86326515	-22.02687619	0.00000000
H	13.86326308	22.02689522	0.00000000

H	14.71920798	-27.95400521	0.00000000
H	14.71920163	27.95402249	0.00000000
H	15.10692988	-19.96372527	0.00000000
H	15.10692853	19.96374335	0.00000000
H	16.36125890	-17.86721624	0.00000000
H	16.36125787	17.86723237	0.00000000
H	16.86765185	-26.71896740	0.00000000
H	16.86764743	26.71898463	0.00000000
H	17.60698348	-15.76729416	0.00000000
H	17.60698320	15.76730645	0.00000000
H	18.09909558	-24.65655334	0.00000000
H	18.09909108	24.65656991	0.00000000
H	18.84453897	-13.66372477	0.00000000
H	18.84454003	13.66373144	0.00000000
H	19.39559106	-22.54267004	0.00000000
H	19.39558672	22.54268498	0.00000000
H	20.07371633	-11.55588127	0.00000000
H	20.07371914	11.55588111	0.00000000
H	20.66067084	-20.42902317	0.00000000
H	20.66066686	20.42903453	0.00000000
H	21.29441923	-9.44333648	0.00000000
H	21.29442364	9.44332926	0.00000000
H	21.91588391	-18.31324249	0.00000000
H	21.91588053	18.31324906	0.00000000
H	22.50673617	-7.32576892	0.00000000
H	22.50674144	7.32575544	0.00000000
H	23.16270357	-16.19323998	0.00000000
H	23.16270148	16.19324047	0.00000000
H	23.71098974	-5.20285587	0.00000000
H	23.71099478	5.20283752	0.00000000
H	24.40143992	-14.06861758	0.00000000
H	24.40143968	14.06861116	0.00000000
H	24.90685426	-3.07386455	0.00000000
H	24.90685789	3.07384285	0.00000000
H	25.63213065	-11.93908192	0.00000000
H	25.63213237	11.93906862	0.00000000
H	26.07734781	-0.97085179	0.00000000
H	26.07734936	0.97082735	0.00000000
H	26.85467371	-9.80426213	0.00000000
H	26.85467699	9.80424290	0.00000000
H	28.06892296	-7.66350545	0.00000000
H	28.06892705	7.66348179	0.00000000
H	29.27563970	-5.51434385	0.00000000
H	29.27564373	5.51431706	0.00000000
H	30.46581916	-3.33600007	0.00000000
H	30.46582218	3.33597130	0.00000000
H	31.64174858	-1.23886321	0.00000000
H	31.64175001	1.23883259	0.00000000

14-HGQR (triplet)

C	-33.15994342	-0.67991698	0.00000000
C	-33.15994303	0.68016623	0.00000000
C	-31.96096519	-2.81526986	0.00000000
C	-31.94437816	-1.42884071	0.00000000
C	-31.94437786	1.42908938	0.00000000
C	-31.96096459	2.81551636	0.00000000
C	-30.78008617	-4.97869134	0.00000000
C	-30.78082432	-3.58146649	0.00000000
C	-30.68436059	-0.72543577	0.00000000
C	-30.68436061	0.72568536	0.00000000
C	-30.78082543	3.58171105	0.00000000
C	-30.78008810	4.97893149	0.00000000
C	-29.57782565	-7.12285185	0.00000000
C	-29.59187436	-5.72044632	0.00000000
C	-29.51839443	-2.87828090	0.00000000
C	-29.51983281	-1.47143497	0.00000000
C	-29.51983423	1.47168342	0.00000000
C	-29.51839754	2.87852707	0.00000000
C	-29.59187957	5.72068218	0.00000000
C	-29.57783199	7.12308073	0.00000000
C	-28.36772239	-9.26030423	0.00000000
C	-28.38883339	-7.85701127	0.00000000
C	-28.32563258	-5.01007537	0.00000000
C	-28.33185169	-3.61181306	0.00000000
C	-28.33185856	3.61205653	0.00000000
C	-28.32564158	5.01031351	0.00000000
C	-28.38884484	7.85723288	0.00000000
C	-28.36773541	9.26051592	0.00000000
C	-27.14966898	-11.39231319	0.00000000
C	-27.17635053	-9.99002197	0.00000000
C	-27.12364996	-7.14079381	0.00000000
C	-27.13468090	-5.74587774	0.00000000
C	-27.13469543	5.74611070	0.00000000
C	-27.12366718	7.14101813	0.00000000

C	-27.17637066	9.99022348	0.00000000
C	-27.14969127	11.39250181	0.00000000
C	-25.92380869	-13.51952097	0.00000000
C	-25.95516966	-12.11881516	0.00000000
C	-25.91311220	-9.26850775	0.00000000
C	-25.92987686	-7.87389643	0.00000000
C	-25.92990150	7.87411295	0.00000000
C	-25.91314004	9.26871240	0.00000000
C	-25.95520092	12.11899107	0.00000000
C	-25.92384284	13.51968134	0.00000000
C	-24.69034340	-15.64218063	0.00000000
C	-24.72592019	-14.24311571	0.00000000
C	-24.69447506	-11.39244446	0.00000000
C	-24.71747450	-9.99689058	0.00000000
C	-24.71751148	9.99708458	0.00000000
C	-24.69451567	11.39262385	0.00000000
C	-24.72596483	14.24326161	0.00000000
C	-24.69039173	15.64230905	0.00000000
C	-23.44945390	-17.76037162	0.00000000
C	-23.48896404	-16.36285622	0.00000000
C	-23.46790840	-13.51208585	0.00000000
C	-23.49711573	-12.11522012	0.00000000
C	-23.49716683	12.11538629	0.00000000
C	-23.46796348	13.51223548	0.00000000
C	-23.48902395	16.36296940	0.00000000
C	-23.44951828	17.76046632	0.00000000
C	-22.20115405	-19.87424632	0.00000000
C	-22.24451845	-18.47788935	0.00000000
C	-22.23360127	-15.62728229	0.00000000
C	-22.26876715	-14.22902647	0.00000000
C	-22.26883355	14.22916106	0.00000000
C	-22.23367198	15.62739944	0.00000000
C	-22.24459497	18.47796919	0.00000000
C	-22.20123570	19.87430793	0.00000000
C	-20.94514046	-21.98420310	0.00000000
C	-20.99284039	-20.58773653	0.00000000
C	-20.99192766	-17.73799324	0.00000000
C	-21.03259942	-16.33840358	0.00000000
C	-21.03268170	16.33850490	0.00000000
C	-20.99201455	17.73807733	0.00000000
C	-20.99293404	20.58778481	0.00000000
C	-20.94523951	21.98423509	0.00000000
C	-19.68065925	-24.09115475	0.00000000
C	-19.73444997	-22.69162374	0.00000000
C	-19.74358580	-19.84401241	0.00000000
C	-19.78884087	-18.44364634	0.00000000
C	-19.78893892	18.44371510	0.00000000
C	-19.74368864	19.84406522	0.00000000
C	-19.73455997	22.69164474	0.00000000
C	-19.68077364	24.09116297	0.00000000
C	-18.39074561	-26.18989015	0.00000000
C	-18.46832235	-24.78518841	0.00000000
C	-18.48934752	-21.94475447	0.00000000
C	-18.53768513	-20.54543690	0.00000000
C	-18.53779796	20.54547630	0.00000000
C	-18.48946504	21.94477997	0.00000000
C	-18.46844558	24.78518759	0.00000000
C	-18.39087123	26.18988023	0.00000000
C	-17.13625551	-28.29174242	0.00000000
C	-17.17981248	-26.86471380	0.00000000
C	-17.23083119	-24.03886297	0.00000000
C	-17.27855352	-22.64443782	0.00000000
C	-17.27867929	22.64445293	0.00000000
C	-17.23096048	24.03886620	0.00000000
C	-17.17994623	26.86469830	0.00000000
C	-17.13639298	28.29172130	0.00000000
C	-15.95708171	-28.96900269	0.00000000
C	-15.94273452	-26.12130769	0.00000000
C	-16.01075064	-24.73968855	0.00000000
C	-16.01088590	24.73968383	0.00000000
C	-15.94287422	26.12129321	0.00000000
C	-15.95722641	28.96897952	0.00000000
C	-14.70268458	-28.28735753	0.00000000
C	-14.68434836	-26.84410147	0.00000000
C	-14.68449341	26.84408534	0.00000000
C	-14.70283379	28.28733673	0.00000000
C	-13.50948928	-28.99304032	0.00000000
C	-13.45677109	-26.20672431	0.00000000
C	-13.45692312	26.20671086	0.00000000
C	-13.50964552	28.99301927	0.00000000
C	-12.25712562	-28.35217294	0.00000000
C	-12.23646098	-26.90709872	0.00000000
C	-12.23661984	26.90708535	0.00000000

C	-12.25728578	28.35215547	0.00000000
C	-11.04671115	-29.04942108	0.00000000
C	-11.00808274	-26.24546429	0.00000000
C	-11.00824663	26.24545351	0.00000000
C	-11.04687746	29.04940427	0.00000000
C	-9.81093147	-28.39046730	0.00000000
C	-9.79344468	-26.93850604	0.00000000
C	-9.79361387	26.93849488	0.00000000
C	-9.81110162	28.39045306	0.00000000
C	-8.58993505	-29.07972263	0.00000000
C	-8.56035569	-26.27516653	0.00000000
C	-8.56052844	26.27515685	0.00000000
C	-8.59010974	29.07970869	0.00000000
C	-7.35982521	-28.41727037	0.00000000
C	-7.34674878	-26.96330542	0.00000000
C	-7.34692521	26.96329544	0.00000000
C	-7.36000266	28.41725848	0.00000000
C	-6.13506322	-29.10182371	0.00000000
C	-6.11405147	-26.29716849	0.00000000
C	-6.11422977	26.29715944	0.00000000
C	-6.13524331	29.10181253	0.00000000
C	-4.90707838	-28.43608166	0.00000000
C	-4.89838991	-26.98115615	0.00000000
C	-4.89856991	26.98114691	0.00000000
C	-4.90725926	28.43607191	0.00000000
C	-3.68089850	-29.11626266	0.00000000
C	-3.66833207	-26.31201198	0.00000000
C	-3.66851206	26.31200317	0.00000000
C	-3.68107982	29.11625406	0.00000000
C	-2.45360309	-28.44717091	0.00000000
C	-2.44926749	-26.99201866	0.00000000
C	-2.44944706	26.99200983	0.00000000
C	-2.45378318	28.44716292	0.00000000
C	-1.22690877	-29.12340310	0.00000000
C	-1.22272569	-26.31948436	0.00000000
C	-1.22290355	26.31947544	0.00000000
C	-1.22708724	29.12339633	0.00000000
C	0.00008103	-28.45083189	0.00000000
C	0.00008197	-26.99566755	0.00000000
C	-0.00009382	26.99565884	0.00000000
C	-0.00009486	28.45082491	0.00000000
C	1.22706986	-29.12340439	0.00000000
C	1.22289028	-26.31948606	0.00000000
C	1.22271716	26.31947692	0.00000000
C	1.22689681	29.12339838	0.00000000
C	2.45376461	-28.44717363	0.00000000
C	2.44943083	-26.99202184	0.00000000
C	2.44926047	26.99201305	0.00000000
C	2.45359448	28.44716682	0.00000000
C	3.68105853	-29.11626646	0.00000000
C	3.66849540	-26.31201701	0.00000000
C	3.66832781	26.31200765	0.00000000
C	3.68089142	29.11626024	0.00000000
C	4.90723821	-28.43608692	0.00000000
C	4.89855141	-26.98116234	0.00000000
C	4.89838645	26.98115346	0.00000000
C	4.90707362	28.43607978	0.00000000
C	6.13522103	-29.10182973	0.00000000
C	6.11421218	-26.29717661	0.00000000
C	6.11404973	26.29716709	0.00000000
C	6.13505892	29.10182293	0.00000000
C	7.35998206	-28.41727780	0.00000000
C	7.34690706	-26.96331429	0.00000000
C	7.34674687	26.96330553	0.00000000
C	7.35982200	28.41727049	0.00000000
C	8.59008936	-29.07973041	0.00000000
C	8.56051227	-26.27517732	0.00000000
C	8.56035458	26.27516798	0.00000000
C	8.58993112	29.07972351	0.00000000
C	9.81108393	-28.39047631	0.00000000
C	9.79359816	-26.93851704	0.00000000
C	9.79344255	26.93850890	0.00000000
C	9.81092772	28.39046957	0.00000000
C	11.04686072	-29.04942976	0.00000000
C	11.00823359	-26.24547711	0.00000000
C	11.00808073	26.24546860	0.00000000
C	11.04670605	29.04942414	0.00000000
C	12.25727208	-28.35218231	0.00000000
C	12.23660792	-26.90711087	0.00000000
C	12.23645743	26.90710410	0.00000000
C	12.25712039	28.35217748	0.00000000
C	13.50963270	-28.99304810	0.00000000
C	13.45691411	-26.20673781	0.00000000

C	13.45676735	26.20673104	0.00000000
C	13.50948285	28.99304587	0.00000000
C	14.70282369	-28.28736649	0.00000000
C	14.68448585	-26.84411398	0.00000000
C	14.68434300	26.84410916	0.00000000
C	14.70267837	28.28736462	0.00000000
C	15.95721675	-28.96901092	0.00000000
C	15.94286853	-26.12132252	0.00000000
C	16.01088256	-24.73971112	0.00000000
C	16.01074534	24.73969805	0.00000000
C	15.94272911	26.12131641	0.00000000
C	15.95707455	28.96901069	0.00000000
C	17.13638571	-28.29175280	0.00000000
C	17.17994091	-26.86472865	0.00000000
C	17.23095887	-24.03889260	0.00000000
C	17.27867989	-22.64447736	0.00000000
C	17.27854833	22.64444840	0.00000000
C	17.23082572	24.03887322	0.00000000
C	17.17980629	26.86472315	0.00000000
C	17.13624863	28.29175142	0.00000000
C	18.39086779	-26.18990989	0.00000000
C	18.46844404	-24.78521550	0.00000000
C	18.48946703	-21.94480276	0.00000000
C	18.53780202	-20.54549736	0.00000000
C	18.53768075	20.54544808	0.00000000
C	18.48934260	21.94476581	0.00000000
C	18.46831650	24.78519861	0.00000000
C	18.39073935	26.18990004	0.00000000
C	19.68077399	-24.09118941	0.00000000
C	19.73456221	-22.69166859	0.00000000
C	19.74369353	-19.84408381	0.00000000
C	19.78894517	-18.44373204	0.00000000
C	19.78883883	18.44365796	0.00000000
C	19.74358235	19.84402453	0.00000000
C	19.73444451	22.69163531	0.00000000
C	19.68065309	24.09116565	0.00000000
C	20.94524371	-21.98425677	0.00000000
C	20.99293960	-20.58780347	0.00000000
C	20.99202110	-17.73809125	0.00000000
C	21.03268870	-16.33851724	0.00000000
C	21.03260118	16.33841564	0.00000000
C	20.99192703	17.73800593	0.00000000
C	20.99283601	20.58774967	0.00000000
C	20.94513466	21.98421553	0.00000000
C	22.20124265	-19.87432368	0.00000000
C	22.24460246	-18.47798221	0.00000000
C	22.23367894	-15.62740849	0.00000000
C	22.26884033	-14.22916846	0.00000000
C	22.26877347	14.22903930	0.00000000
C	22.23360460	15.62729551	0.00000000
C	22.24451657	18.47790382	0.00000000
C	22.20114987	19.87426052	0.00000000
C	23.44952638	-17.76047606	0.00000000
C	23.48903199	-16.36297698	0.00000000
C	23.46797019	-13.51223950	0.00000000
C	23.49717313	-12.11538850	0.00000000
C	23.49712636	12.11523434	0.00000000
C	23.46791598	13.51209995	0.00000000
C	23.48896583	16.36287154	0.00000000
C	23.44945283	17.76038717	0.00000000
C	24.69039981	-15.64231323	0.00000000
C	24.72597264	-14.24326406	0.00000000
C	24.69452213	-11.39262282	0.00000000
C	24.71751766	-9.99708166	0.00000000
C	24.71748838	9.99690667	0.00000000
C	24.69448632	11.39245993	0.00000000
C	24.72592599	14.24313170	0.00000000
C	24.69034628	15.64219700	0.00000000
C	25.92385046	-13.51968050	0.00000000
C	25.95520837	-12.11898874	0.00000000
C	25.91314667	-9.26870672	0.00000000
C	25.92990818	-7.87410547	0.00000000
C	25.92989257	7.87391435	0.00000000
C	25.91312600	9.26852480	0.00000000
C	25.95517903	12.11883196	0.00000000
C	25.92381544	13.51953799	0.00000000
C	27.14969851	-11.39249652	0.00000000
C	27.17637800	-9.99021693	0.00000000
C	27.12367451	-7.14100859	0.00000000
C	27.13470323	-5.74609962	0.00000000
C	27.13469701	5.74589690	0.00000000
C	27.12366498	7.14081216	0.00000000
C	27.17636255	9.99003965	0.00000000

C	27.14967892	11.39233088	0.00000000
C	28.36774261	-9.26050697	0.00000000
C	28.38885250	-7.85722295	0.00000000
C	28.32565012	-5.01030112	0.00000000
C	28.33186796	-3.61204296	0.00000000
C	28.33186697	3.61183254	0.00000000
C	28.32564760	5.01009433	0.00000000
C	28.38884701	7.85702952	0.00000000
C	28.36773464	9.26032240	0.00000000
C	29.57783963	-7.12306907	0.00000000
C	29.59188802	-5.72066984	0.00000000
C	29.51840762	-2.87851275	0.00000000
C	29.51984523	-1.47166845	0.00000000
C	29.51984612	1.47145358	0.00000000
C	29.51840842	2.87829937	0.00000000
C	29.59188850	5.72046448	0.00000000
C	29.57783920	7.12286996	0.00000000
C	30.78009661	-4.97891801	0.00000000
C	30.78083493	-3.58169714	0.00000000
C	30.68437192	-0.72566956	0.00000000
C	30.68437271	0.72545227	0.00000000
C	30.78083794	3.58148375	0.00000000
C	30.78009997	4.97870863	0.00000000
C	31.96097407	-2.81550184	0.00000000
C	31.94438844	-1.42907446	0.00000000
C	31.94439060	1.42885620	0.00000000
C	31.96097808	2.81528568	0.00000000
C	33.15995406	-0.68015222	0.00000000
C	33.15995535	0.67993174	0.00000000
H	-34.10601033	-1.23875218	0.00000000
H	-34.10600980	1.23900064	0.00000000
H	-32.93050652	-3.33539648	0.00000000
H	-32.93050532	3.33564124	0.00000000
H	-31.74142953	-5.51355350	0.00000000
H	-31.74143006	5.51379113	0.00000000
H	-30.53627067	-7.66294820	0.00000000
H	-30.53627437	7.66317430	0.00000000
H	-29.32401843	-9.80425656	0.00000000
H	-29.32402748	9.80446526	0.00000000
H	-28.54145388	-0.97112410	0.00000000
H	-28.54145663	0.97137100	0.00000000
H	-28.10387501	-11.93997478	0.00000000
H	-28.10389202	11.94016035	0.00000000
H	-27.37138065	-3.07515413	0.00000000
H	-27.37139008	3.07539721	0.00000000
H	-26.87598191	-14.07073904	0.00000000
H	-26.87600968	14.07089638	0.00000000
H	-26.17650663	-5.20535577	0.00000000
H	-26.17652514	5.20558890	0.00000000
H	-25.64051613	-16.19683905	0.00000000
H	-25.64055735	16.19696442	0.00000000
H	-24.97370708	-7.32980099	0.00000000
H	-24.97373696	7.33001828	0.00000000
H	-24.39763084	-18.31840371	0.00000000
H	-24.39768786	18.31849536	0.00000000
H	-23.76329613	-9.44926300	0.00000000
H	-23.76333938	9.44945835	0.00000000
H	-23.14732189	-20.43562647	0.00000000
H	-23.14739638	20.43568519	0.00000000
H	-22.54495888	-11.56405563	0.00000000
H	-22.54501690	11.56422371	0.00000000
H	-21.88929330	-22.54890527	0.00000000
H	-21.88938579	22.54893489	0.00000000
H	-21.31862952	-13.67438780	0.00000000
H	-21.31870306	13.67452479	0.00000000
H	-20.62206304	-24.66037310	0.00000000
H	-20.62217154	24.66038016	0.00000000
H	-20.08444244	-15.78041369	0.00000000
H	-20.08453159	15.78051786	0.00000000
H	-19.32419418	-26.77234085	0.00000000
H	-19.32431530	26.77232995	0.00000000
H	-18.84258331	-17.88248567	0.00000000
H	-18.84268749	17.88255772	0.00000000
H	-18.09221234	-28.83344797	0.00000000
H	-18.09234749	28.83342396	0.00000000
H	-17.59326596	-19.98121888	0.00000000
H	-17.59338380	19.98126196	0.00000000
H	-16.33602294	-22.07687865	0.00000000
H	-16.33615266	22.07689737	0.00000000
H	-15.94316257	-30.06767651	0.00000000
H	-15.94330725	30.06765065	0.00000000
H	-15.09006639	-24.13972145	0.00000000
H	-15.09020348	24.13972148	0.00000000

H	-13.54211869	-30.09279770	0.00000000
H	-13.40263749	-25.10918836	0.00000000
H	-13.40278884	25.10917852	0.00000000
H	-13.54227650	30.09277424	0.00000000
H	-11.06375215	-30.14938252	0.00000000
H	-10.99299244	-25.14536500	0.00000000
H	-10.99315455	25.14535712	0.00000000
H	-11.06391958	30.14936356	0.00000000
H	-8.60184074	-30.17977670	0.00000000
H	-8.54898295	-25.17512348	0.00000000
H	-8.54915412	25.17511592	0.00000000
H	-8.60201557	30.17976105	0.00000000
H	-6.14342035	-30.20193851	0.00000000
H	-6.10590835	-25.19708188	0.00000000
H	-6.10608507	25.19707415	0.00000000
H	-6.14360008	30.20192632	0.00000000
H	-3.68584030	-30.21642261	0.00000000
H	-3.66339760	-25.21188428	0.00000000
H	-3.66357611	25.21187596	0.00000000
H	-3.68602106	30.21641380	0.00000000
H	-1.22854336	-30.22358748	0.00000000
H	-1.22107269	-25.21933299	0.00000000
H	-1.22124950	25.21932380	0.00000000
H	-1.22872134	30.22358121	0.00000000
H	1.22870266	-30.22358858	0.00000000
H	1.22123803	-25.21933494	0.00000000
H	1.22106535	25.21932497	0.00000000
H	1.22852987	30.22358352	0.00000000
H	3.68599863	-30.21642582	0.00000000
H	3.66356162	-25.21189006	0.00000000
H	3.66339409	25.21187953	0.00000000
H	3.68583153	30.21642074	0.00000000
H	6.14357667	-30.20194357	0.00000000
H	6.10606960	-25.19709127	0.00000000
H	6.10590729	25.19708032	0.00000000
H	6.14341437	30.20193791	0.00000000
H	8.60199390	-30.17978317	0.00000000
H	8.54913984	-25.17513610	0.00000000
H	8.54898261	25.17512502	0.00000000
H	8.60183533	30.17977743	0.00000000
H	11.06390120	-30.14938956	0.00000000
H	10.99314316	-25.14538026	0.00000000
H	10.99299128	25.14536959	0.00000000
H	11.06374596	30.14938529	0.00000000
H	13.54226187	-30.09280355	0.00000000
H	13.40278057	-25.10920494	0.00000000
H	13.40263527	25.10919539	0.00000000
H	13.54211132	30.09280297	0.00000000
H	15.09020036	-24.13974756	0.00000000
H	15.09006144	24.13973117	0.00000000
H	15.94329614	-30.06768244	0.00000000
H	15.94315435	30.06768416	0.00000000
H	16.33615311	-22.07692143	0.00000000
H	16.33601837	22.07688883	0.00000000
H	17.59338762	-19.98128320	0.00000000
H	17.59326220	19.98122944	0.00000000
H	18.09234009	-28.83345655	0.00000000
H	18.09220492	28.83345716	0.00000000
H	18.84269338	-17.88257540	0.00000000
H	18.84258203	17.88249638	0.00000000
H	19.32431197	-26.77236033	0.00000000
H	19.32418724	26.77235093	0.00000000
H	20.08453817	-15.78053126	0.00000000
H	20.08444514	15.78042475	0.00000000
H	20.62217218	-24.66040728	0.00000000
H	20.62205594	24.66038430	0.00000000
H	21.31870941	-13.67453331	0.00000000
H	21.31863698	13.67439978	0.00000000
H	21.88939035	-22.54895720	0.00000000
H	21.88928642	22.54891803	0.00000000
H	22.54502280	-11.56422694	0.00000000
H	22.54497085	11.56406925	0.00000000
H	23.14740374	-20.43570116	0.00000000
H	23.14731648	20.43564132	0.00000000
H	23.76334525	-9.44945624	0.00000000
H	23.76331148	9.44927875	0.00000000
H	24.39769639	-18.31850469	0.00000000
H	24.39762838	18.31842034	0.00000000
H	24.97374350	-7.33001132	0.00000000
H	24.97372423	7.32981884	0.00000000
H	25.64056585	-16.19696768	0.00000000
H	25.64051748	16.19685678	0.00000000
H	26.17653302	-5.20557807	0.00000000

H	26.17652396	5.20537516	0.00000000
H	26.87601761	-14.07089441	0.00000000
H	26.87598711	14.07075733	0.00000000
H	27.37139976	-3.07538381	0.00000000
H	27.37139675	3.07517420	0.00000000
H	28.10389938	-11.94015402	0.00000000
H	28.10388359	11.93999328	0.00000000
H	28.54146777	-0.97135707	0.00000000
H	28.54146733	0.97114421	0.00000000
H	29.32403457	-9.80445553	0.00000000
H	29.32402965	9.80427489	0.00000000
H	30.53628169	-7.66316217	0.00000000
H	30.53628359	7.66296586	0.00000000
H	31.74143813	-5.51377741	0.00000000
H	31.74144312	5.51356981	0.00000000
H	32.93051413	-3.33562703	0.00000000
H	32.93051928	3.33541139	0.00000000
H	34.10601973	-1.23898767	0.00000000
H	34.10602189	1.23876668	0.00000000

15-HGQR (triplet)

C	-35.62701384	-0.68001893	0.00000000
C	-35.62701369	0.68003071	0.00000000
C	-34.42821427	-2.81515140	0.00000000
C	-34.41140375	-1.42878925	0.00000000
C	-34.41140352	1.42880078	0.00000000
C	-34.42821385	2.81516261	0.00000000
C	-33.24810737	-4.97864997	0.00000000
C	-33.24826473	-3.58154659	0.00000000
C	-33.15122857	-0.72553850	0.00000000
C	-33.15122848	0.72555000	0.00000000
C	-33.24826447	3.58155739	0.00000000
C	-33.24810712	4.97866014	0.00000000
C	-32.04703924	-7.12312832	0.00000000
C	-32.06019327	-5.72089351	0.00000000
C	-31.98556047	-2.87871199	0.00000000
C	-31.98680649	-1.47179962	0.00000000
C	-31.98680649	1.47181079	0.00000000
C	-31.98556055	2.87872284	0.00000000
C	-32.06019341	5.72090297	0.00000000
C	-32.04703950	7.12313689	0.00000000
C	-30.83852200	-9.26115309	0.00000000
C	-30.85844444	-7.85804544	0.00000000
C	-30.79354529	-5.01111017	0.00000000
C	-30.79931019	-3.61272458	0.00000000
C	-30.79931065	3.61273490	0.00000000
C	-30.79354593	5.01111979	0.00000000
C	-30.85844527	7.85805300	0.00000000
C	-30.83852307	9.26115955	0.00000000
C	-29.62240064	-11.39401211	0.00000000
C	-29.64765822	-9.99180915	0.00000000
C	-29.59273612	-7.14266100	0.00000000
C	-29.60305892	-5.74756663	0.00000000
C	-29.60306012	5.74757544	0.00000000
C	-29.59273761	7.14266878	0.00000000
C	-29.64765998	9.99181443	0.00000000
C	-29.62240270	11.39401622	0.00000000
C	-28.39877847	-13.52237396	0.00000000
C	-28.42859543	-12.12148483	0.00000000
C	-28.38382108	-9.27139813	0.00000000
C	-28.39956892	-7.87660615	0.00000000
C	-28.39957109	7.87661288	0.00000000
C	-28.38382358	9.27140363	0.00000000
C	-28.42859819	12.12148776	0.00000000
C	-28.39878152	13.52237582	0.00000000
C	-27.16784806	-15.64648358	0.00000000
C	-27.20190500	-14.24680435	0.00000000
C	-27.16726968	-11.39650884	0.00000000
C	-27.18885972	-10.00090842	0.00000000
C	-27.18886291	10.00091275	0.00000000
C	-27.16727315	11.39651193	0.00000000
C	-27.20190862	14.24680517	0.00000000
C	-27.16785188	15.64648360	0.00000000
C	-25.92986395	-17.76634614	0.00000000
C	-25.96795085	-16.36781161	0.00000000
C	-25.94322747	-13.51745672	0.00000000
C	-25.97055850	-12.12087452	0.00000000
C	-25.97056255	12.12087644	0.00000000
C	-25.94323168	13.51745759	0.00000000
C	-25.96795502	16.36781082	0.00000000
C	-25.92986818	17.76634490	0.00000000
C	-24.68503864	-19.88194695	0.00000000
C	-24.72690118	-18.48461602	0.00000000
C	-24.71178037	-15.63414089	0.00000000

C	-24.74463205	-14.23664538	0.00000000
C	-24.74463662	14.23664524	0.00000000
C	-24.71178496	15.63414001	0.00000000
C	-24.72690549	18.48461422	0.00000000
C	-24.68504286	19.88194502	0.00000000
C	-23.43339832	-21.99344684	0.00000000
C	-23.47887850	-20.59712789	0.00000000
C	-23.47309056	-17.74667941	0.00000000
C	-23.51126809	-16.34820999	0.00000000
C	-23.51127278	16.34820835	0.00000000
C	-23.47309512	17.74667740	0.00000000
C	-23.47888256	20.59712561	0.00000000
C	-23.43340216	21.99344466	0.00000000
C	-22.17463428	-24.10129366	0.00000000
C	-22.22412428	-22.70485717	0.00000000
C	-22.22754479	-19.85514842	0.00000000
C	-22.27072563	-18.45559886	0.00000000
C	-22.27073005	18.45559633	0.00000000
C	-22.22754897	19.85514582	0.00000000
C	-22.22412780	22.70485477	0.00000000
C	-22.17463749	24.10129146	0.00000000
C	-20.90799212	-26.20645320	0.00000000
C	-20.96317993	-24.80699793	0.00000000
C	-20.97587043	-21.95935434	0.00000000
C	-21.02323631	-20.55910740	0.00000000
C	-21.02324020	20.55910448	0.00000000
C	-20.97587401	21.95935156	0.00000000
C	-20.96318276	24.80699556	0.00000000
C	-20.90799457	26.20645102	0.00000000
C	-19.61651463	-28.30373757	0.00000000
C	-19.69507015	-26.89916798	0.00000000
C	-19.71886575	-24.05867935	0.00000000
C	-19.76896489	-22.65945008	0.00000000
C	-19.76896811	22.65944709	0.00000000
C	-19.71886862	24.05867658	0.00000000
C	-19.69507227	26.89916561	0.00000000
C	-19.61651637	28.30373527	0.00000000
C	-18.36122167	-30.40466162	0.00000000
C	-18.40520189	-28.97770031	0.00000000
C	-18.45816921	-26.15172618	0.00000000
C	-18.50729884	-24.75732217	0.00000000
C	-18.50730140	24.75731923	0.00000000
C	-18.45817142	26.15172344	0.00000000
C	-18.40520338	28.97769785	0.00000000
C	-18.36122304	30.40465917	0.00000000
C	-17.18179494	-31.08137209	0.00000000
C	-17.16854110	-28.23345154	0.00000000
C	-17.23755462	-26.85180917	0.00000000
C	-17.23755661	26.85180630	0.00000000
C	-17.16854253	28.23344893	0.00000000
C	-17.18179625	31.08136959	0.00000000
C	-15.92784384	-30.39901609	0.00000000
C	-15.90984696	-28.95567815	0.00000000
C	-15.90984813	28.95567564	0.00000000
C	-15.92784510	30.39901363	0.00000000
C	-14.73457629	-31.10440636	0.00000000
C	-14.68230863	-28.31806629	0.00000000
C	-14.68230940	28.31806415	0.00000000
C	-14.73457763	31.10440407	0.00000000
C	-13.48239568	-30.46334338	0.00000000
C	-13.46175054	-29.01819753	0.00000000
C	-13.46175140	29.01819567	0.00000000
C	-13.48239685	30.46334145	0.00000000
C	-12.27220477	-31.16068904	0.00000000
C	-12.23326144	-28.35670325	0.00000000
C	-12.23326209	28.35670176	0.00000000
C	-12.27220610	31.16068734	0.00000000
C	-11.03636202	-30.50188656	0.00000000
C	-11.01851573	-29.04985990	0.00000000
C	-11.01851652	29.04985869	0.00000000
C	-11.03636315	30.50188524	0.00000000
C	-9.81584735	-31.19158384	0.00000000
C	-9.78519369	-28.38699577	0.00000000
C	-9.78519429	28.38699497	0.00000000
C	-9.81584867	31.19158272	0.00000000
C	-8.58543838	-30.52960409	0.00000000
C	-8.57161348	-29.07559537	0.00000000
C	-8.57161426	29.07559484	0.00000000
C	-8.58543954	30.52960341	0.00000000
C	-7.36137810	-31.21495052	0.00000000
C	-7.33853108	-28.41025386	0.00000000
C	-7.33853174	28.41025378	0.00000000
C	-7.36137950	31.21494999	0.00000000

C	-6.13293791	-30.54997097	0.00000000
C	-6.12310558	-29.09507388	0.00000000
C	-6.12310653	29.09507404	0.00000000
C	-6.13293925	30.54997091	0.00000000
C	-4.90755820	-31.23130838	0.00000000
C	-4.89238794	-28.42704009	0.00000000
C	-4.89238887	28.42704073	0.00000000
C	-4.90755986	31.23130845	0.00000000
C	-3.67981433	-30.56326686	0.00000000
C	-3.67393274	-29.10826161	0.00000000
C	-3.67393407	29.10826244	0.00000000
C	-3.67981603	30.56326742	0.00000000
C	-2.45381536	-31.24101456	0.00000000
C	-2.44624869	-28.43716469	0.00000000
C	-2.44625012	28.43716601	0.00000000
C	-2.45381749	31.24101522	0.00000000
C	-1.22659247	-30.56982209	0.00000000
C	-1.22463517	-29.11491907	0.00000000
C	-1.22463712	29.11492052	0.00000000
C	-1.22659476	30.56982322	0.00000000
C	0.00000959	-31.24423411	0.00000000
C	0.00000913	-28.44054527	0.00000000
C	0.00000694	28.44054718	0.00000000
C	0.00000680	31.24423530	0.00000000
C	1.22661140	-30.56982167	0.00000000
C	1.22465361	-29.11491869	0.00000000
C	1.22465087	29.11492066	0.00000000
C	1.22660835	30.56982330	0.00000000
C	2.45383442	-31.24101369	0.00000000
C	2.44626679	-28.43716394	0.00000000
C	2.44626372	28.43716632	0.00000000
C	2.45383084	31.24101537	0.00000000
C	3.67983297	-30.56326557	0.00000000
C	3.67395089	-29.10826044	0.00000000
C	3.67394725	29.10826283	0.00000000
C	3.67982910	30.56326764	0.00000000
C	4.90757681	-31.23130659	0.00000000
C	4.89240560	-28.42703857	0.00000000
C	4.89240160	28.42704129	0.00000000
C	4.90757245	31.23130870	0.00000000
C	6.13295596	-30.54996878	0.00000000
C	6.12312313	-29.09507192	0.00000000
C	6.12311865	29.09507459	0.00000000
C	6.13295131	30.54997119	0.00000000
C	7.36139594	-31.21494780	0.00000000
C	7.33854795	-28.41025157	0.00000000
C	7.33854314	28.41025448	0.00000000
C	7.36139091	31.21495022	0.00000000
C	8.58545547	-30.52960098	0.00000000
C	8.57163006	-29.07559259	0.00000000
C	8.57162492	29.07559542	0.00000000
C	8.58545024	30.52960362	0.00000000
C	9.81586407	-31.19158015	0.00000000
C	9.78520938	-28.38699270	0.00000000
C	9.78520401	28.38699565	0.00000000
C	9.81585861	31.19158278	0.00000000
C	11.03637778	-30.50188245	0.00000000
C	11.01853095	-29.04985626	0.00000000
C	11.01852540	29.04985911	0.00000000
C	11.03637220	30.50188519	0.00000000
C	12.27222003	-31.16068424	0.00000000
C	12.23327554	-28.35669934	0.00000000
C	12.23326989	28.35670222	0.00000000
C	12.27221439	31.16068695	0.00000000
C	13.48240967	-30.46333805	0.00000000
C	13.46176390	-29.01819287	0.00000000
C	13.46175824	29.01819567	0.00000000
C	13.48240400	30.46334082	0.00000000
C	14.73458970	-31.10440005	0.00000000
C	14.68232055	-28.31806133	0.00000000
C	14.68231493	28.31806407	0.00000000
C	14.73458410	31.10440278	0.00000000
C	15.92785565	-30.39900944	0.00000000
C	15.90985766	-28.95567242	0.00000000
C	15.90985226	28.95567509	0.00000000
C	15.92785017	30.39901219	0.00000000
C	17.18180593	-31.08136476	0.00000000
C	17.16855058	-28.23344599	0.00000000
C	17.23756298	-26.85180578	0.00000000
C	17.23755806	26.85180797	0.00000000
C	17.16854539	28.23344858	0.00000000
C	17.18180055	31.08136759	0.00000000
C	18.36123093	-30.40465453	0.00000000

C	18.40521012	-28.97769444	0.00000000
C	18.45817621	-26.15172462	0.00000000
C	18.50730488	-24.75732345	0.00000000
C	18.50730037	24.75732474	0.00000000
C	18.45817152	26.15172645	0.00000000
C	18.40520514	28.97769709	0.00000000
C	18.36122582	30.40465734	0.00000000
C	19.61652094	-28.30373295	0.00000000
C	19.69507575	-26.89916549	0.00000000
C	19.71886976	-24.05868308	0.00000000
C	19.76896754	-22.65945719	0.00000000
C	19.76896367	22.65945727	0.00000000
C	19.71886565	24.05868387	0.00000000
C	19.69507129	26.89916751	0.00000000
C	19.61651631	28.30373537	0.00000000
C	20.90799569	-26.20645286	0.00000000
C	20.96318222	-24.80700054	0.00000000
C	20.97587055	-21.95936446	0.00000000
C	21.02323487	-20.55912120	0.00000000
C	21.02323179	20.55911981	0.00000000
C	20.97586722	21.95936389	0.00000000
C	20.96317845	24.80700155	0.00000000
C	20.90799165	26.20645449	0.00000000
C	22.17463411	-24.10129885	0.00000000
C	22.22412247	-22.70486604	0.00000000
C	22.22754065	-19.85516532	0.00000000
C	22.27071987	-18.45561921	0.00000000
C	22.27071766	18.45561627	0.00000000
C	22.22753818	19.85516322	0.00000000
C	22.22411955	22.70486570	0.00000000
C	22.17463088	24.10129932	0.00000000
C	23.43339390	-21.99345865	0.00000000
C	23.47887248	-20.59714357	0.00000000
C	23.47308240	-17.74670239	0.00000000
C	23.51125852	-16.34823559	0.00000000
C	23.51125710	16.34823125	0.00000000
C	23.47308078	17.74669880	0.00000000
C	23.47887046	20.59714171	0.00000000
C	23.43339159	21.99345767	0.00000000
C	24.68503027	-19.88196554	0.00000000
C	24.72689151	-18.48463800	0.00000000
C	24.71176909	-15.63416817	0.00000000
C	24.74461978	-14.23667399	0.00000000
C	24.74461897	14.23666859	0.00000000
C	24.71176818	15.63416334	0.00000000
C	24.72689033	18.48463463	0.00000000
C	24.68502885	19.88196301	0.00000000
C	25.92985259	-17.76637048	0.00000000
C	25.96793869	-16.36783828	0.00000000
C	25.94321445	-13.51748587	0.00000000
C	25.97054502	-12.12090354	0.00000000
C	25.97054463	12.12089749	0.00000000
C	25.94321403	13.51748018	0.00000000
C	25.96793817	16.36783365	0.00000000
C	25.92985193	17.76636653	0.00000000
C	27.16783516	-15.64651165	0.00000000
C	27.20189187	-14.24683334	0.00000000
C	27.16725647	-11.39653729	0.00000000
C	27.18884661	-10.00093545	0.00000000
C	27.18884643	10.00092912	0.00000000
C	27.16725634	11.39653114	0.00000000
C	27.20189177	14.24682781	0.00000000
C	27.16783504	15.64650659	0.00000000
C	28.39876562	-13.52240321	0.00000000
C	28.42858284	-12.12151358	0.00000000
C	28.38380910	-9.27142376	0.00000000
C	28.39955749	-7.87662948	0.00000000
C	28.39955738	7.87662317	0.00000000
C	28.38380908	9.27141749	0.00000000
C	28.42858294	12.12150755	0.00000000
C	28.39876579	13.52239744	0.00000000
C	29.62238920	-11.39404005	0.00000000
C	29.64764738	-9.99183545	0.00000000
C	29.59272638	-7.14268255	0.00000000
C	29.60305001	-5.74758554	0.00000000
C	29.60304991	5.74757938	0.00000000
C	29.59272637	7.14267636	0.00000000
C	29.64764752	9.99182926	0.00000000
C	29.62238945	11.39403396	0.00000000
C	30.83851289	-9.26117784	0.00000000
C	30.85843610	-7.85806789	0.00000000
C	30.79353832	-5.01112728	0.00000000
C	30.79930414	-3.61273918	0.00000000

C	30.79930407	3.61273321	0.00000000
C	30.79353829	5.01112125	0.00000000
C	30.85843618	7.85806176	0.00000000
C	30.83851309	9.26117172	0.00000000
C	32.04703282	-7.12314898	0.00000000
C	32.06018774	-5.72091168	0.00000000
C	31.98555657	-2.87872508	0.00000000
C	31.98680378	-1.47181047	0.00000000
C	31.98680376	1.47180465	0.00000000
C	31.98555654	2.87871923	0.00000000
C	32.06018775	5.72090572	0.00000000
C	32.04703292	7.12314298	0.00000000
C	33.24810374	-4.97866668	0.00000000
C	33.24826223	-3.58156089	0.00000000
C	33.15122797	-0.72554790	0.00000000
C	33.15122796	0.72554215	0.00000000
C	33.24826220	3.58155509	0.00000000
C	33.24810376	4.97866084	0.00000000
C	34.42821378	-2.81516487	0.00000000
C	34.41140411	-1.42880032	0.00000000
C	34.41140408	1.42879462	0.00000000
C	34.42821376	2.81515916	0.00000000
C	35.62701544	-0.68002871	0.00000000
C	35.62701543	0.68002308	0.00000000
H	-36.57295561	-1.23899918	0.00000000
H	-36.57295538	1.23901097	0.00000000
H	-35.39782372	-3.33511114	0.00000000
H	-35.39782317	3.33512225	0.00000000
H	-34.20963026	-5.51314359	0.00000000
H	-34.20962980	5.51315357	0.00000000
H	-33.00577926	-7.66265664	0.00000000
H	-33.00577918	7.66266501	0.00000000
H	-31.79521448	-9.80436249	0.00000000
H	-31.79521509	9.80436878	0.00000000
H	-31.00837484	-0.97161072	0.00000000
H	-31.00837507	0.97162158	0.00000000
H	-30.57710236	-11.94076493	0.00000000
H	-30.57710390	11.94076897	0.00000000
H	-29.83864389	-3.07645376	0.00000000
H	-29.83864469	3.07646393	0.00000000
H	-29.35154913	-14.07252028	0.00000000
H	-29.35155166	14.07252219	0.00000000
H	-28.64459063	-5.20760705	0.00000000
H	-28.64459228	5.20761582	0.00000000
H	-28.11873174	-16.19989663	0.00000000
H	-28.11873510	16.19989681	0.00000000
H	-27.44299974	-7.33325577	0.00000000
H	-27.44300240	7.33326258	0.00000000
H	-26.87888450	-18.32293525	0.00000000
H	-26.87888837	18.32293425	0.00000000
H	-26.23417366	-9.45420822	0.00000000
H	-26.23417733	9.45421268	0.00000000
H	-25.63219926	-20.44165907	0.00000000
H	-25.63220324	20.44165743	0.00000000
H	-25.01778481	-11.57081356	0.00000000
H	-25.01778926	11.57081565	0.00000000
H	-24.37868999	-22.55625727	0.00000000
H	-24.37869369	22.55625538	0.00000000
H	-23.79377355	-13.68326774	0.00000000
H	-23.79377841	13.68326774	0.00000000
H	-23.11805777	-24.66717127	0.00000000
H	-23.11806089	24.66716933	0.00000000
H	-22.56229988	-15.79160744	0.00000000
H	-22.56230475	15.79160585	0.00000000
H	-21.84882582	-26.77657023	0.00000000
H	-21.84882819	26.77656829	0.00000000
H	-21.32359426	-17.89590846	0.00000000
H	-21.32359880	17.89590585	0.00000000
H	-20.54956091	-28.88679621	0.00000000
H	-20.54956259	28.88679408	0.00000000
H	-20.07785476	-19.99651080	0.00000000
H	-20.07785872	19.99650769	0.00000000
H	-19.31702002	-30.94657575	0.00000000
H	-19.31702139	30.94657332	0.00000000
H	-18.82527116	-22.09405634	0.00000000
H	-18.82527447	22.09405309	0.00000000
H	-17.56534740	-24.18883734	0.00000000
H	-17.56535006	24.18883411	0.00000000
H	-17.16716273	-32.18000182	0.00000000
H	-17.16716405	32.17999930	0.00000000
H	-16.31724557	-26.25127770	0.00000000
H	-16.31724787	26.25127437	0.00000000
H	-14.76696899	-32.20415275	0.00000000

H	-14.62845850	-27.22052175	0.00000000
H	-14.62845882	27.22051966	0.00000000
H	-14.76697057	32.20415041	0.00000000
H	-12.28934266	-32.26062809	0.00000000
H	-12.21804685	-27.25662260	0.00000000
H	-12.21804725	27.25662115	0.00000000
H	-12.28934434	32.26062630	0.00000000
H	-9.82816957	-32.29161377	0.00000000
H	-9.77340615	-27.28697421	0.00000000
H	-9.77340649	27.28697349	0.00000000
H	-9.82817124	32.29161254	0.00000000
H	-7.37044860	-32.31504005	0.00000000
H	-7.32966909	-27.31019008	0.00000000
H	-7.32966949	27.31019011	0.00000000
H	-7.37045035	32.31503938	0.00000000
H	-4.91350772	-32.33144462	0.00000000
H	-4.88642216	-27.32693398	0.00000000
H	-4.88642282	27.32693476	0.00000000
H	-4.91350970	32.33144452	0.00000000
H	-2.45675849	-32.34118041	0.00000000
H	-2.44324890	-27.33702989	0.00000000
H	-2.44325007	27.33703138	0.00000000
H	-2.45676090	32.34118088	0.00000000
H	0.00000972	-32.34440986	0.00000000
H	0.00000893	-27.34040066	0.00000000
H	0.00000696	27.34040276	0.00000000
H	0.00000673	32.34441086	0.00000000
H	2.45677786	-32.34117946	0.00000000
H	2.44326655	-27.33702922	0.00000000
H	2.44326369	27.33703178	0.00000000
H	2.45677411	32.34118097	0.00000000
H	4.91352665	-32.33144268	0.00000000
H	4.88643933	-27.32693264	0.00000000
H	4.88643551	27.32693551	0.00000000
H	4.91352217	32.33144463	0.00000000
H	7.37046677	-32.31503710	0.00000000
H	7.32968544	-27.31018808	0.00000000
H	7.32968077	27.31019110	0.00000000
H	7.37046169	32.31503941	0.00000000
H	9.82818664	-32.29160977	0.00000000
H	9.77342129	-27.28697154	0.00000000
H	9.77341599	27.28697456	0.00000000
H	9.82818119	32.29161233	0.00000000
H	12.28935838	-32.26062291	0.00000000
H	12.21806030	-27.25661924	0.00000000
H	12.21805469	27.25662213	0.00000000
H	12.28935279	32.26062559	0.00000000
H	14.76698292	-32.20414599	0.00000000
H	14.62846988	-27.22051755	0.00000000
H	14.62846417	27.22052026	0.00000000
H	14.76697734	32.20414872	0.00000000
H	16.31725423	-26.25127548	0.00000000
H	16.31724936	26.25127745	0.00000000
H	17.16717392	-32.17999397	0.00000000
H	17.16716850	32.17999683	0.00000000
H	17.56535425	-24.18883956	0.00000000
H	17.56534964	24.18884072	0.00000000
H	18.82527488	-22.09406439	0.00000000
H	18.82527085	22.09406434	0.00000000
H	19.31702884	-30.94656802	0.00000000
H	19.31702379	30.94657087	0.00000000
H	20.07785456	-19.99652536	0.00000000
H	20.07785128	19.99652383	0.00000000
H	20.54956628	-28.88679138	0.00000000
H	20.54956178	28.88679380	0.00000000
H	21.32358976	-17.89592926	0.00000000
H	21.32358732	17.89592619	0.00000000
H	21.84882817	-26.77656959	0.00000000
H	21.84882430	26.77657128	0.00000000
H	22.56229136	-15.79163312	0.00000000
H	22.56228970	15.79162867	0.00000000
H	23.11805634	-24.66717582	0.00000000
H	23.11805331	24.66717637	0.00000000
H	23.79376195	-13.68329608	0.00000000
H	23.79376093	13.68329058	0.00000000
H	24.37868438	-22.55626821	0.00000000
H	24.37868229	22.55626734	0.00000000
H	25.01777153	-11.57084203	0.00000000
H	25.01777096	11.57083589	0.00000000
H	25.63218994	-20.44167665	0.00000000
H	25.63218877	20.44167424	0.00000000
H	26.23416029	-9.45423452	0.00000000
H	26.23415996	9.45422814	0.00000000

H	26.87887260	-18.32295859	0.00000000
H	26.87887218	18.32295475	0.00000000
H	27.44298766	-7.33327834	0.00000000
H	27.44298743	7.33327200	0.00000000
H	28.11871879	-16.19992388	0.00000000
H	28.11871888	16.19991892	0.00000000
H	28.64458085	-5.20762522	0.00000000
H	28.64458066	5.20761905	0.00000000
H	29.35153669	-14.07254902	0.00000000
H	29.35153702	14.07254334	0.00000000
H	29.83863690	-3.07646757	0.00000000
H	29.83863678	3.07646159	0.00000000
H	30.57709166	-11.94079274	0.00000000
H	30.57709203	11.94078674	0.00000000
H	31.00837120	-0.97162065	0.00000000
H	31.00837115	0.97161486	0.00000000
H	31.79520625	-9.80438749	0.00000000
H	31.79520652	9.80438146	0.00000000
H	33.00577369	-7.66267790	0.00000000
H	33.00577383	7.66267199	0.00000000
H	34.20962727	-5.51316124	0.00000000
H	34.20962730	5.51315549	0.00000000
H	35.39782378	-3.33512556	0.00000000
H	35.39782378	3.33511990	0.00000000
H	36.57295811	-1.23900917	0.00000000
H	36.57295808	1.23900359	0.00000000

FIGURES

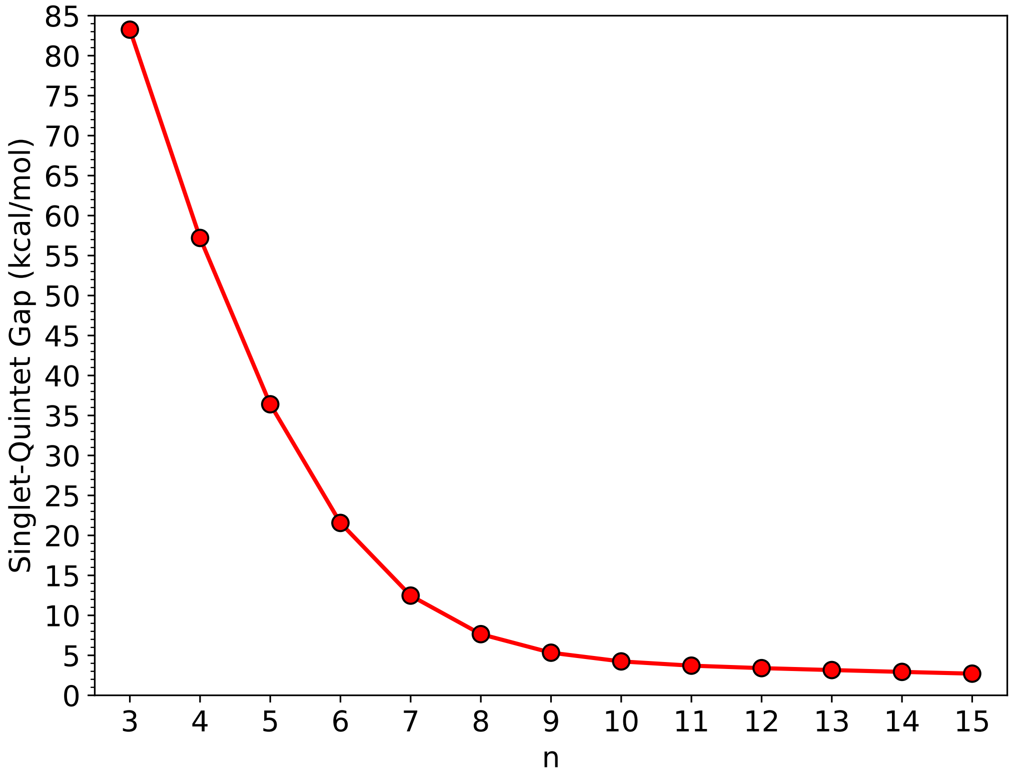

FIG. S1. Singlet-quintet gap of n-HGQR, calculated by spin-unrestricted TAO-LDA.

TABLES

TABLE S1. Singlet-triplet gap E_{ST} (in kcal/mol) of n-HGQR, calculated by spin-unrestricted KS-LDA and TAO-LDA.

n	KS-LDA	TAO-LDA
3	51.14	36.03
4	40.03	23.10
5	31.33	13.18
6	12.63	6.74
7	15.27	3.42
8	10.34	1.96
9	6.51	1.33
10	3.30	1.05
11	0.90	0.91
12	1.12	0.84
13	2.03	0.78
14	2.38	0.73
15		0.68

TABLE S2. Vertical ionization potential IP_v (in eV), vertical electron affinity EA_v (in eV), fundamental gap E_g (in eV), and symmetrized von Neumann entropy S_{vN} of ground-state n-HGQR, calculated by spin-unrestricted TAO-LDA.

n	IP_v	EA_v	E_g	S_{vN}
3	5.95	1.62	4.33	0.09
4	5.40	2.22	3.18	0.33
5	5.01	2.64	2.37	0.95
6	4.74	2.91	1.83	2.14
7	5.58	3.09	1.49	3.79
8	4.48	3.19	1.29	5.59
9	4.41	3.27	1.15	7.18
10	4.36	3.32.	1.04	8.47
11	4.32	3.36	0.96	9.54
12	4.29	3.40	0.89	10.54
13	4.26	3.43	0.83	11.55
14	4.23	3.45	0.78	12.62
15	4.21	3.48	0.73	13.74
